# Electronic Character of α,3‐Dehydrotoluene Intermediates Generated from Isolable Allenyne‐Containing Substrates

**DOI:** 10.1002/anie.202207510

**Published:** 2022-08-29

**Authors:** Qian Xu, Thomas R. Hoye

**Affiliations:** ^1^ Department of Chemistry University of Minnesota 207 Pleasant St., SE Minneapolis MN 55455 USA

**Keywords:** Myers-Saito cyclization, zwitterions, diradicals, reaction mechanisms, reactive intermediates

## Abstract

We report here the generation of α,3‐dehydrotoluenes, a relatively rare subset of reactive intermediates of the dehydroaromatic family, from isolable allenynes. The substructure motif in the allenyne substrates is distinct from, and complementary to, those found in Myers‐Saito/Schmittel‐type cycloisomerizations. The reactions reported here give rise to product profiles that provide insight about the electronic nature (i.e., diradical vs. zwitterion vs. cyclic allene) of the particular isomeric DHT(s) that is(are) produced under different reaction conditions differing most significantly in the polarity of the reaction solvent. One example also revealed previously unobserved carbene‐like reactivity of the DHT.

## Graphical abstract

α,3‐Dehydroluenes (DHT), a relatively rare class of reactive intermediate(s), can be formed by warming isolable allenynes bearing a remote alkyne or nitrile. The product distributions produced in solvents of different polarity give insight to the electronic nature/structure of the DHTs (zwitterion vs. diradical vs. cyclic allene).

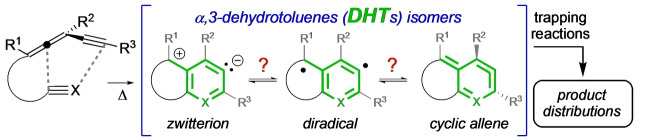

α,3‐Dehydrotoluene (DHT, *m*‐dehydrotoluene), formally a tautomer of 3‐methyl‐*o*‐benzyne, is a reactive intermediate that can be represented as any of the electronically distinct structures **1 a**–**c**. These were first independently described in 1989 by the groups of Myers^[1a]^ and Saito.^[1b]^ The prototypical reaction for their generation (from **2**) and trapping (to give **3** and **4**)^[2]^ is shown in Figure 1a. The parent DHT **1** was soon after characterized by photoelectron spectroscopy^[3a]^ and heat of formation^[3b]^ in the Chen and Wenthold laboratories, respectively. The differences among zwitterionic, diradical, and cyclic allene variants (**1 a**–**c**, respectively) have been studied, and debated, both experimentally and computationally.^[4]^ Because of the formation of both the ether and alcohol products **3** and **4** from polar vs. radical type processes, respectively, Myers and co‐workers offered two possibilities: either the DHT was a hybrid of resonance contributors **1 a/b** having partial dipolar/diradical character or that these two were independent, but interconverting species. Carpenter and co‐workers further studied this reaction both experimentally and computationally and concluded that **1 a** and **1 b** are discrete species, formed from **2** via competitive bifurcation within a common transition structure (TS), and that the branching is dependent upon the polarity of the reaction medium.

**Figure 1:**
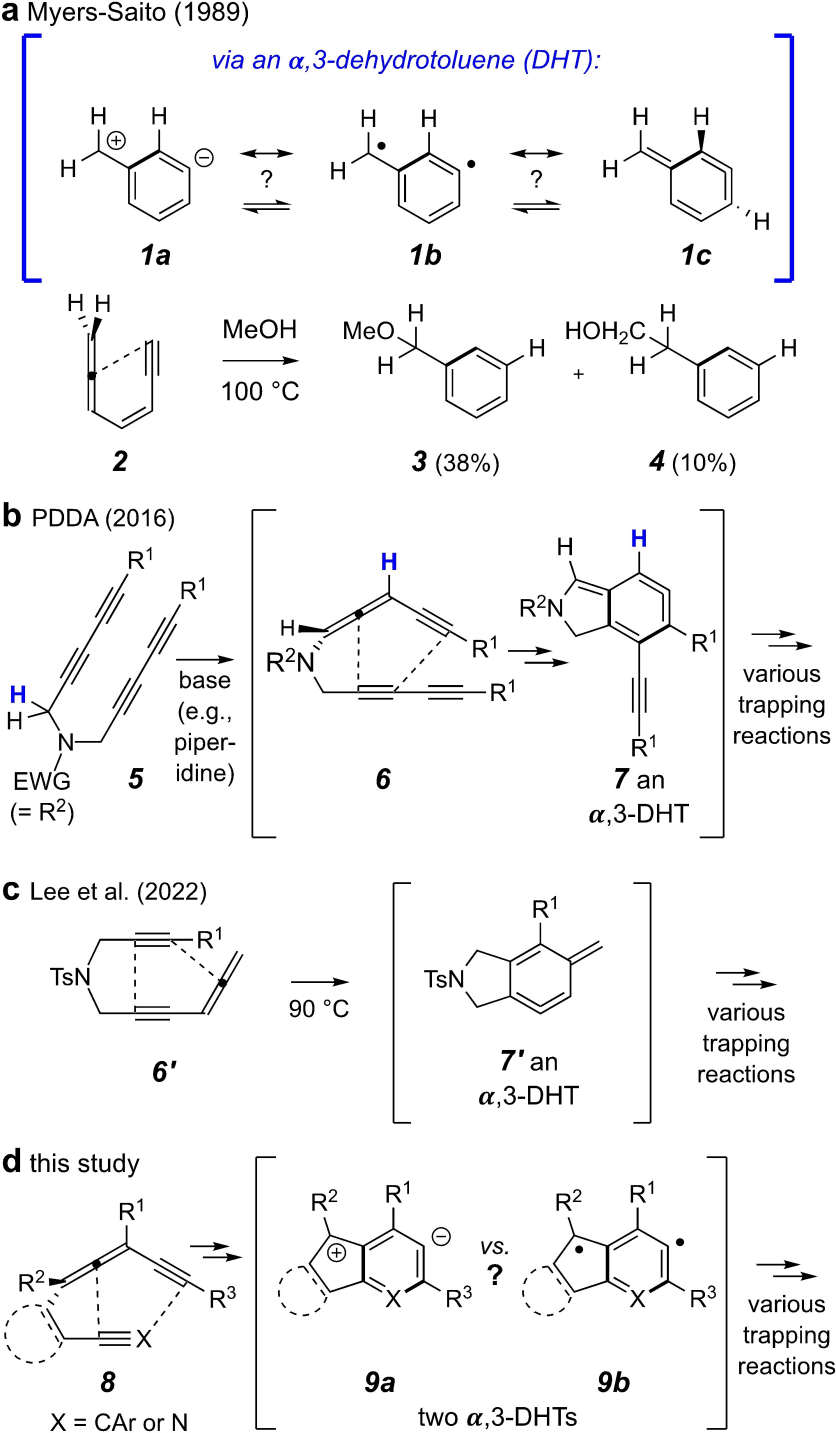
a) Myers‐Saito cyclization: one‐bond formation of DHTs from **2**. b) pentadehydro‐Diels–Alder (PDDA) cyclization: two‐bond formation of DHTs from in situ‐generated allenes **6**. c) two‐bond formation of DHTs from isolable allenes **6′**, topologically complementary to **6** (and **8**). d) this study: two‐bond formation of DHTs from isolable allenes **8**, analogs of **6**.

Previously we have described cyclizations like those shown in Figure 1b, which we established to proceed via a DHT formed from allenynes **6**, generated in situ via tautomerization of **5**.^[5]^ These engage a remotely tethered alkyne that functions as an allenynophile. We called these pentadehydro‐Diels–Alder (PDDA) reactions because five sp‐carbons reside within the reacting framework of **6**; in contrast there are three such carbons in a (fully conjugated) Myers‐Saito substrate. In that earlier study^[5]^ we did not explore the electronic nature of the DHT, portraying it there only as the cyclic allene **7**, simply as a matter of convenience. Very recently, researchers in the Lee laboratory reported the thermal cyclization of **isolable** allenynes^[6]^ **6′** and the subsequent capture of the DHTs **7′**, which revealed interesting modes of reactivity depending on the nature of the heteroatom (O vs. N) in the trapping nucleophile (Figure 1c).^[7]^

In the studies being reported here (Figure 1d), we sought to understand the electronic character of these structurally complex DHT intermediates, as revealed by details of their engagement with trapping agents. As in Lee′s studies,^[7]^ we also have used **isolable** allenynes bearing a tethered arylalkyne or nitrile (cf. **8**). These produce the isomeric DHTs **9 a** and/or **9 b** under various reaction conditions. The complementary topologies of the allenyne substrates **6′** and **8** are notable, the former having the allene distal to the tethered allenynophile and the latter, proximal.

We first established a method for preparing allenynes such as **8** [Figure 2a, see compounds S1–S6 in the Supporting Information].^[8]^ These were sufficiently stable to be easily isolated. However, when warmed, for example to 80 °C for **11 a**, conversion over the course of several hours to various products was observed. When the reaction medium contained acetic acid, **11 a** gave the acetate ester **12 a** as the only observed product (79 % isolated yield). This type of trapping event is most consistent with the depiction of the DHT intermediate as the zwitterionic structure **13**. Protonation of the localized carbanionic carbon would produce the ion pair **14** prior to its collapse to **12 a**.

**Figure 2:**
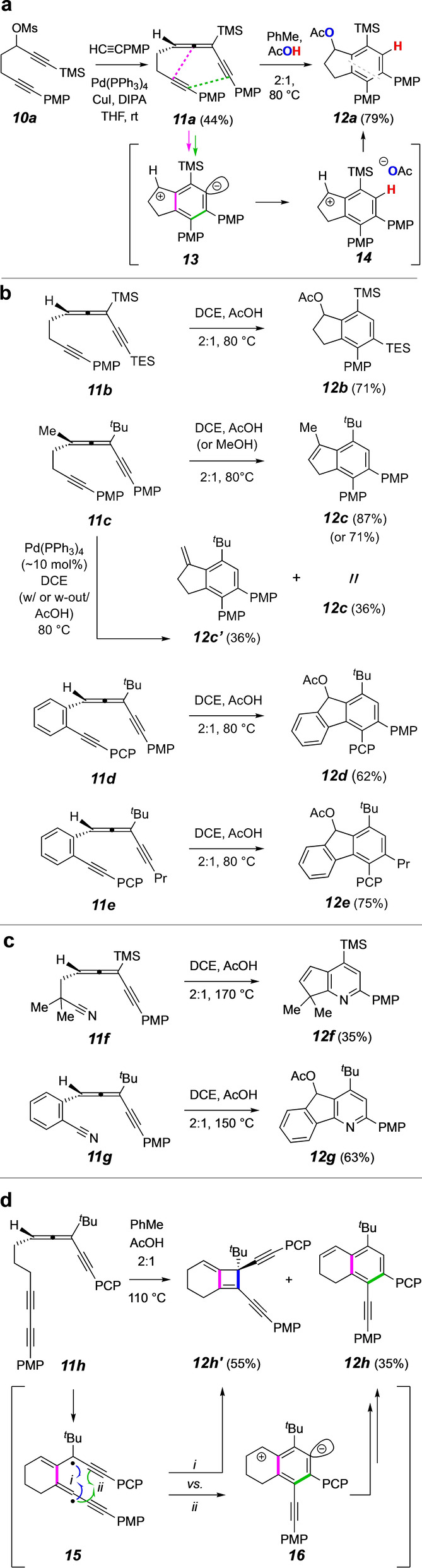
a) First experiment: **11 a** (from **10 a**) to **12 a** via DHT **13** and ion pair **14**. b) Six additional reactions of (four) allenynes having a tethered alkyne (**11 b**–**11 e**). c) Two reactions of allenynes having a tethered cyano group as the allenynophile (**11 f**–**11 g**). d) Formation of a cyclobutene from a substrate (**11 h**) having a one‐carbon longer tether. [TMS: trimethylsilyl; PMP: *p*‐methoxyphenyl; DIPA: diisopropylamine; DCE: 1,2‐dichloroethane; TES: triethylsilyl; PCP: *p*‐chlorophenyl.]

Additional examples of reactions of allenynes containing tethered alkynes are shown in Figure 2b. Substrate **11 b**, a triethylsilyl analog of **11 a**, undergoes a similar transformation to produce **12 b**. However, the tetrasubstituted allene substrate **11 c**, now having a *t*‐butyl in place of TMS substituent on the allene, efficiently gave rise to the indene derivative **12 c**. When this experiment was performed using methanol rather than AcOH as the protic co‐solvent, the same indene product was formed, suggesting that the intermediate benzylic cation (an analog of **14**) proceeded to the alkene directly, since the tertiary methyl ether would likely be stable under the reaction conditions. An interesting impact of a Pd(0) complex was observed; now a 1 : 1 mixture of alkene isomers **12 c** and **12 c’** was formed, regardless of whether AcOH was used as a proton source. This suggests the involvement of an intermediate having Pd at its benzylic carbon from which β‐hydride elimination could occur. Substrates **11 d** and **11 e** contain an arene linker; these fully conjugated alkynyl allenynes gave rise to the fluorenyl acetate products **12 d** and **12 e**, respectively. These conjugated substrates cyclized to the DHT noticeably faster than analogs having the saturated CH_2_CH_2_ linker [e.g., cf. t_1/2_s for **11 a** (∼0.5 h @ 80 °C) vs. **11 d** (∼8 h @ 20 °C); see SI].

Reactions of two nitrile‐containing substrates, one conjugated with the allenyne and one not (**11 g** and **11 f**, respectively), are given in Figure 2c. Each required a significantly higher reaction temperature^[9]^ for conversion to the aza‐arene **12 g** [cf. t_1/2_s for **11 g** (∼3 h @ 145 °C) vs. **11 d** (∼8 h @ 20 °C); see SI] or **12 f**. This reflects the partial reduction in bond order of the inherently stronger C≡N (vs. C≡C) triple bond^[10]^ in the TS leading to formation of the initial diradical (see discussion associated with Figure S1 in the SI).

Finally, we prepared the substrate **11 h** (Figure 2d), now containing a three‐ rather than two‐atom linkage between the proximal allene and tethered alkyne. Interestingly, this gave rise mainly to the novel cyclobutene‐containing product **12 h’** in addition to the dihydronaphthalene isomer **12 h**. This outcome can be rationalized by competitive ring closure within an initial diradical intermediate **15** (cf. blue vs. green arrows), now preferentially giving the cyclobutene. This mode of cyclization was not observed with the earlier substrates, presumably because of the higher strain that would be present in the TS leading to the cyclopentene analog of the cyclohexene **12 h’**.^[11]^ We have supported this rationale with DFT computations using model systems and a variant of the distortion/interaction‐activation strain model^[12]^ applicable to intramolecular reactions (see Figure S5 and associated extensive discussion in the SI).^[13]^

We then designed several experiments in which the trapping group was present within the substrate (Figure 3). These provided additional mechanistic insight bearing on the electronic character of the DHT intermediates. The hydroxyl‐bearing substrate **17** was studied (Figure 3a), first in a solution of 1,2‐dichloroethane (DCE) only. This gave the expected indene derivative **18** as the only isolated product. When the solvent was changed to a 1 : 1 mixture of DCE and 1,4‐cyclohexadiene (1,4‐CHD), the latter a known hydrogen atom donor to the diradical isomers of DHTs,[^1^, ^2^, ^14^] formation of the indene **18** was accompanied by the appearance of a small amount of the saturated indane analog **18‐H_2_**. When a toluene/1,4‐CHD solvent mixture was used, the proportion of **18‐H_2_** grew to ca. 1 : 1 relative to **18**. Changing to the significantly more polar solvent mixture of Et_2_NH/THF (1 : 1), led to the formation of a unique product, the aldehyde **19** (rationalized below); a trace amount of **18** was also detected (GC‐MS).

**Figure 3:**
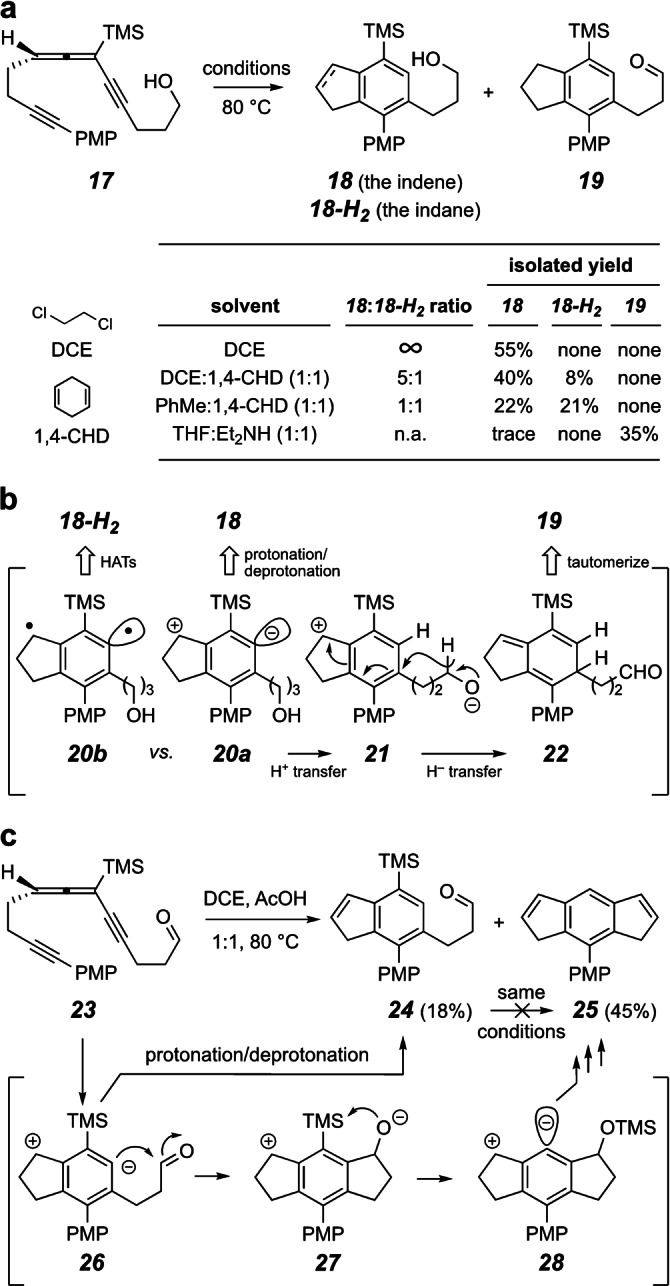
a) Effects of solvent polarity on the ratio of products **18**, **18‐H_2_**, and **19**. b) Rationale for formation of these products. c) The tethered aldehyde in **23** participates in C−C bond formation enroute to **25**.

These results shed light on the preferential formation of the zwitterionic (**20 a**) vs. diradical (**20 b**) DHT (Figure 3b). In DCE alone only **18** is formed, presumably via formation of **20 a** followed by protonation and final indene formation through proton loss. The formation of **18‐H_2_** using the somewhat less polar mixture of DCE/1,4‐CHD suggests the formation (and net reduction by 1,4‐CHD) of the diradical DHT **20 b**. Even more of that pathway was followed in the even less polar toluene/1,4‐CHD solvent. Overall, these three experiments suggested that the solvent polarity influences the relative amounts of **20 a** and **20 b** that are generated, the less polar medium leading to increased amount of the diradical relative to the zwitterion.[^4a^, ^4b^, ^14^]

In contrast, in the most polar medium of THF/Et_2_NH, the formation of **19** can be accounted for by an overall internal redox process. We suggest that this polar environment preferentially gives rise to **20 a**, which, following protonation at its aryl carbon, produces the alkoxide zwitterion, **21**. An intramolecular Cannizzaro‐like hydride transfer would annihilate the charges, giving, ultimately **19**, via the transient, dearomatized tautomer **22**.

Another experiment involving an aldehyde is shown in Figure 3c. Namely, the aldehyde here was built into the substrate allenyne **23** in such a fashion that it might engage the proximal DHT carbanionic carbon atom. Indeed, when **23** was heated in 1 : 1 DCE:AcOH, a small amount of the mono‐indene **24** was isolated, but the major product was the (symmetrical) bis‐indene derivative **25**. Formation of the former presumably follows the same protonation/deprotonation events from the initially formed zwitterionic DHT **26**. Generation of the bis‐indene **25** requires C−C bond formation (**26** to **27**), again providing strong evidence for the intermediacy of the zwitterionic DHT. A Brook rearrangement would account for the conversion of **27** to **28**, from which a proton transfer and AcOH‐promoted net dehydration would account for the generation of the second indenyl ring. It is curious that only indenyl rather than acetoxyindanyl products (cf. **12 a** and **12 b**) were observed in this experiment; the more negative σ_*p*_^+^ value of the alkyl vs. aryl or silyl substituent para to the benzylic cation center^[15]^ could explain this outcome. A control experiment verified that the aldehyde **24** was not a viable precursor to **25** under the same reaction conditions.

Two additional reactions, each proceeding via unusual rearrangement events, shed additional light on the electronic nature of the relevant DHT intermediates and their reactivity (Figure 4). In the first (Figure 4a), when heated in a non‐polar reaction medium (toluene), the *t*‐butyl containing allenic nitrile **11 g** isomerized to the three pyridine derivatives **29**–**31**. When perdeuterotoluene was used as the solvent, none of these products showed evidence of deuterium incorporation, suggesting an intramolecular nature for these transformations. Although isolated in only low yields, the unusual structure of each strongly implicated their formation via the diradical DHT **32**. Specifically, it is proposed that the radical center at the sp^2^‐hybridized arene carbon abstracts a *t*‐butyl methyl hydrogen atom to give the new diradical **33**.^[16]^ This could then, competitively, either collapse to the strained, polycyclic structure **29** or rearrange to the more stable tertiary/benzylic diradical **35**, by way of the closed‐shell, spirocyclic cyclopropane **34**. This would either collapse to the pentacycle **30** or undergo hydrogen‐atom transfer to produce the alkene **31**. A similar set of events (and mechanistic rationale) has been described for a benzo analog of the diradical **33**.^[17]^

**Figure 4:**
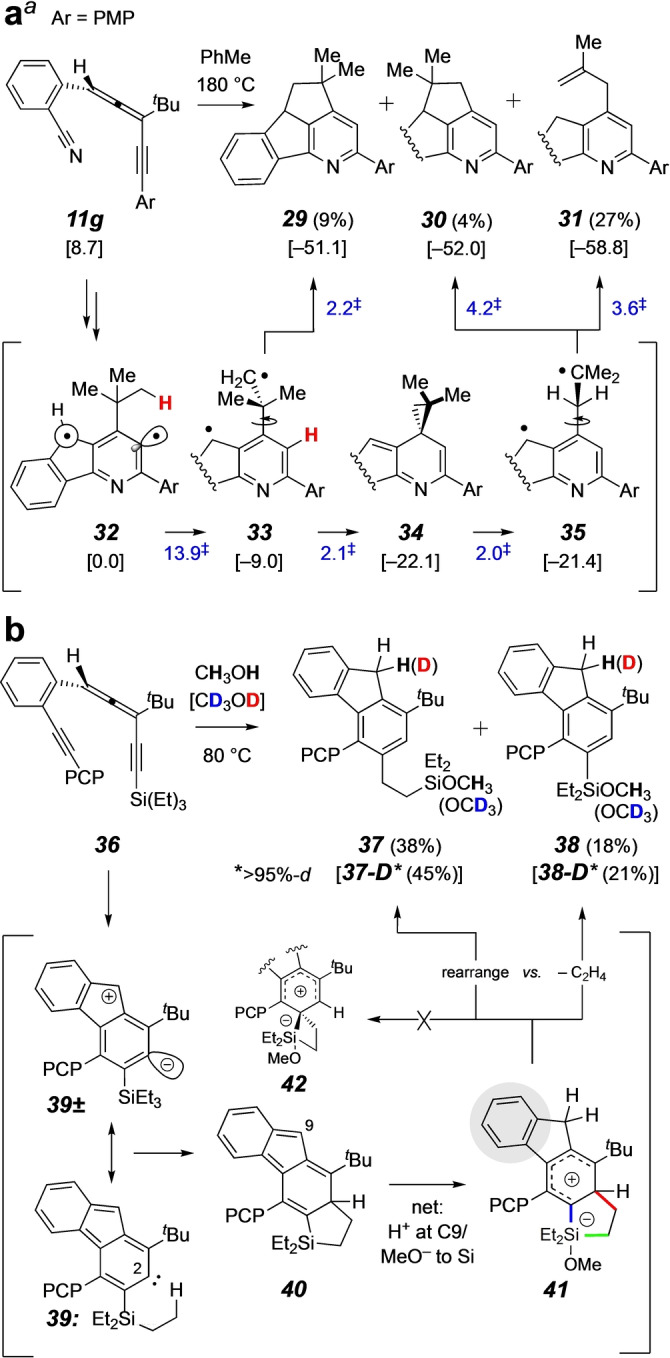
Mechanistic rationales for formation of unusual products a) **29**–**31** provides evidence for the diradical DHT **32** and b) **37** and **38** provides evidence for carbenic character (cf. resonance contributor **39**:) in the zwitterionic DHT **39**. ^*a*^ Numbers in brackets below each structure number are the relative Gibbs energies (kcal mol^−1^) computed [Ar=Ph, DFT: SMD(dichloroethane)/B3LYP‐GD3BJ/6–311++G(d,p)] for each of those species; numbers in blue font are the computed activation barriers (kcal mol^−1^) for each forward step in the reaction pathway.

This reaction manifold was analyzed using DFT (see SI, Figure S3). The results are summarized by i) the relative Gibbs energies shown in brackets directly below each of the optimized structures **29**–**35** and ii) the activation barriers (in blue) for the forward reaction steps that convert the diradical **32** into each of the products **29**–**31**. All barriers for the forward reactions are quite low. Two pairs of competitive events, **33** to **29** vs. **34** and **35** to **30** vs. **31**, have relative activation barriers that are in close alignment with the observed product ratios.

In a second experiment, also resulting in a strange skeletal reorganization, the TES‐containing allenic alkyne **36** was heated in methanol and the diethylmethoxysilanes **37** and **38** were formed, rather cleanly, as the only isolable products. These transformations can be rationalized by initial formation of the zwitterionic DHT **39**, comprised of the principal resonance contributors **39±** and **39**:. The closed‐shell, carbenic character of this DHT is now conferred by conjugation provided through the benzo linker in substrate **36**, absent in DHT derived from the analogous, non‐conjugated substrate **11 b**. In **39** the TES group presents an appetizing C−H bond proximate to C2, and insertion therein would produce **40**. The zwitterion **41** could arise by net addition of methanol, made feasible by the reestablishment of benzenoid aromaticity (gray highlights). Loss of ethylene from **41** and concomitant generation of a second benzo ring would give the fluorene **38**. A low barrier process was identified by DFT for this concerted fragmentation (cleavage of red and green bonds, see Supporting Information for the TS, Figure S4); a related fragmentation to eject ethylene (or propylene) has been described by Xia and Lee.^[18]^ Finally, to account for the formation of **37**, we considered a 1,2‐migration (ring contraction) within **41** to produce the spirocyclic isomer **42**. Extensive attempts using DFT to locate this silacyclobutane were unsuccessful. However, we did uncover a concerted primary event that converts **41** directly to **37** by another low barrier process—namely, a surprising, simultaneous 1,2‐migration of the red C−C bond and breakage of the blue Si−C bond within **41**.

Finally, we explored the potential energy surface for the conversion of **43 d** and **43 g** [the phenyl analogs of substrates **11 d** (pendant alkyne) and **11 g** (pendant nitrile)] to their corresponding DHT intermediates **44 a/b** and **45 a/b**, respectively. A fuller discussion of these results is provided in the Supporting Information (Figures S1 and S2), but the most essential findings (Figure 5) are that: i) the nitrile analog shows a significantly higher activation barrier for its rate‐determining step (ΔΔG^≠^ ca. 9 kcal mol^−1^), consistent with the relative half‐lives for cyclization of substrates **11 g** vs. **11 d** (see above); ii) in each case, the zwitterionic DHT is considerably higher in free energy than the diradical isomer;[^4a^, ^4b^] iii) the small values of the charge distributions of the zwitterionic forms **44 a** and **45 a** (blue font) are consistent with their considerable carbene character (cf. **39**: in Figure 4b); and iv) in the diradical DHTs **44 b** and **45 b**, the radical character (spin population) is more localized on the aromatic C2‐ than on the benzylic C9‐carbon atoms (green font).

**Figure 5:**
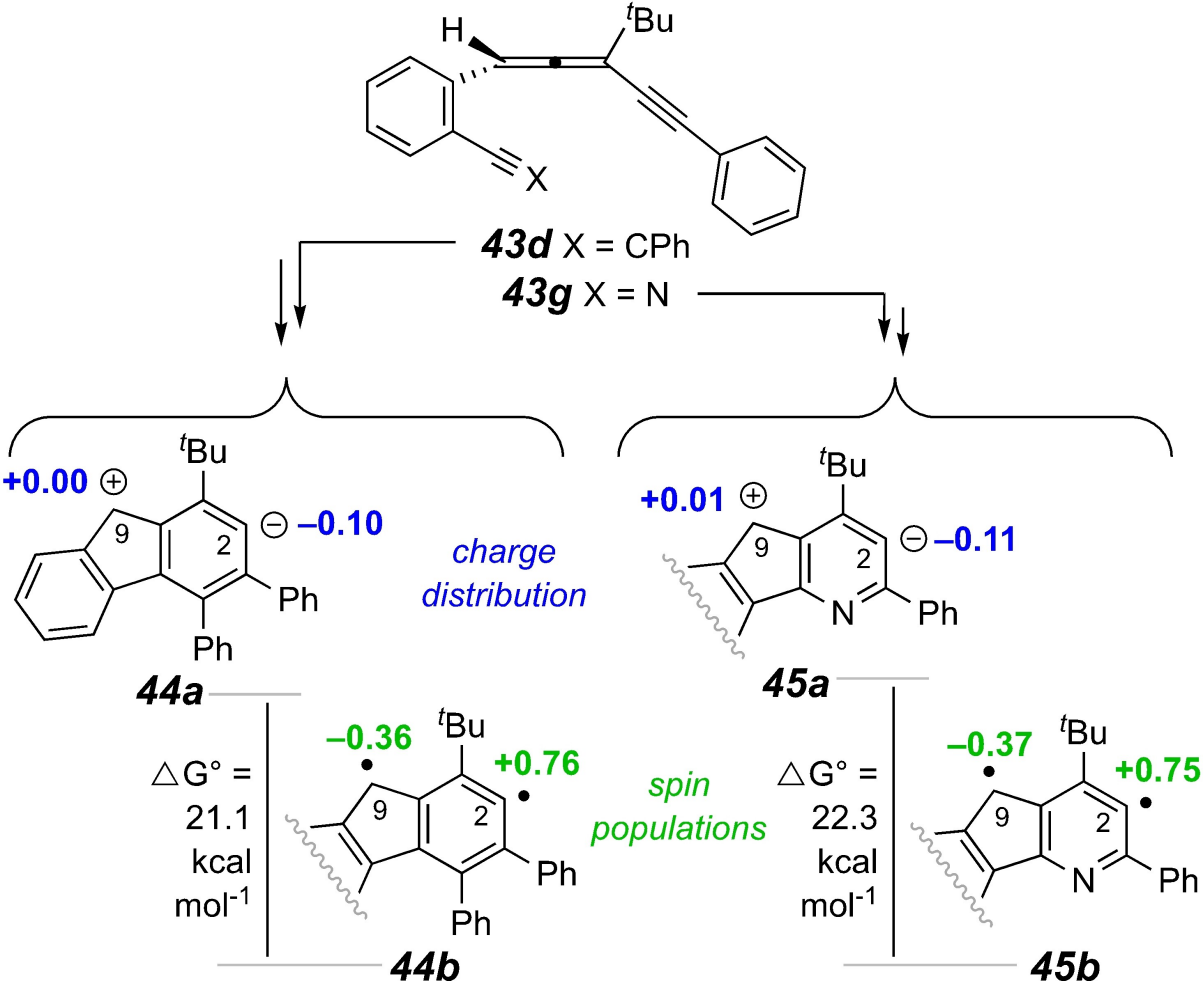
Insights from DFT study [SMD(dichloroethane)/B3LYP‐GD3BJ/6–311++G(d,p)] of the DHT intermediates **44** and **45**. The relatively low charge density (blue) in the fully conjugated zwitterionic DHTs **44 a** and **45 a** suggests that they are carbenic in character. Spin populations in the diradical isomers (green) **44 b** and **45 b** suggest significant delocalization of the C9‐radical character into the fluorene π‐system.

In summary, thermal activation of isolable, conjugated allenynes bearing an alkyne or nitrile tethered to the allene under various conditions has allowed us to identify the electronic nature (zwitterionic vs. diradical vs. allenic,^[19]^ Figure 1a) of the isomeric dehydrotoluene reactive intermediates that are involved. Evidence of diradical DHTs came not only from a net hydrogenation by external 1,4‐cyclohexadiene, but also by unusual translocation of the diradical intermediate produced from **11 g** (Figure 4a). More polar solvent media led to formation of larger proportions of the zwitterionic DHT isomers. Reactions performed in solutions containing AcOH often resulted in respectable isolated yields of trapped, polycyclic acetate ester (Figure 2) or indene products. Finally, we found evidence (Figure 4b) consistent with heretofore unrecognized carbene character of the zwitterionic DHT, a feature further supported by DFT calculations.

## Conflict of interest

The authors declare no conflict of interest.

## Supporting information

As a service to our authors and readers, this journal provides supporting information supplied by the authors. Such materials are peer reviewed and may be re‐organized for online delivery, but are not copy‐edited or typeset. Technical support issues arising from supporting information (other than missing files) should be addressed to the authors.

Supporting Information

Supporting Information

## Data Availability

The data that support the findings of this study are available in the supplementary material of this article.
