# Supplementary material for: Electronic Character of α,3‐Dehydrotoluene Intermediates Generated from Isolable Allenyne‐Containing Substrates
Source: Angew Chem Int Ed Engl. 2022 Aug 29;61(40):e202207510. doi: 10.1002/anie.202207510 (PMC9529817; doi:10.1002/anie.202207510)
Supplement: Supplementary file 1 — Supporting Information [file ANIE-61-0-s002.pdf]

Angewandte Chemie
Eine Zeitschrift der Gesellschaft Deutscher Chemiker

Supporting Information

Electronic Character of α,3-Dehydrotoluene Intermediates Generated from Isolable Allenyne-Containing Substrates

*Q. Xu, T. R. Hoye**

Table of Contents

Contents 2–216

I. General Experimental Protocols 4

II. General Procedures A–B 4

III. Preparation Procedures and Characterization Data for all Compounds 5–38

$t_{1/2}$ for cyclization of substrates **11a** (p S11), **11d** (p S14), **11g** (p S17), and **17** (p S28)

IV. Computational Results 39–123

a) Methods 39

b) Discussion of results 40–44

i/ii) Figures S1/S2. PESs for **43d** and **43g** to products **44a/b** and **45a/b**; and spin populations and charge distributions in DHTs **44a/b** and **45a/b.** 40–41

iii) Figure S3. Expanded version of the PES for the formation of **29–31** from **11g.** 41

iv) Figure S4. Transition structures and energies for the competitive conversions of **S54** to **S55** vs. **S56**. 42

v) Figure S5. DFT analyses to assess the differential strain energies within the tethering atoms in diradicals **S57** *vs.* **S58**. 43–44

c) Co-ordinates and energies for each stationary state 45–123

Figure S6. ChemDraw structures of all stationary states 45

V. References 124

VI. Copies of NMR spectra 124–216

^{1}H and ^{13}C for S1 125–126

^{1}H and ^{13}C for S2 126–128

^{1}H and ^{13}C for S3 129–130

^{1}H and ^{13}C for S4 131–132

^{1}H and ^{13}C for S5 133–134

^{1}H and ^{13}C for S6 135–136

^{1}H and ^{13}C for 10a 137–138

^{1}H and ^{13}C for 11a 139–140

^{1}H and ^{13}C for 11b 141–142

^{1}H and ^{13}C for 11c 143–144

^{1}H for 11d 145

^{1}H and ^{13}C for 11e 146–149

^{1}H and ^{13}C for 11f 148–149

^{1}H and ^{13}C for 11g 150–151

^{1}H and ^{13}C for 11h 152–153

^{1}H and ^{13}C for 12a 154–155

^{1}H and ^{13}C for 12b 156–157

^{1}H and ^{13}C for 12c 158–159

^{1}H and ^{13}C for 12c’ 160–161

^{1}H and ^{13}C for 12d 162–163

^{1}H and ^{13}C for 12e 164–165

^{1}H and ^{13}C for 12f 166–167

^{1}H and ^{13}C for 12g 168–169

**^{1}H and ^{13}C for 12h**	170-171
**^{1}H, ^{13}C, COESY, HSQC and HMBC for 12h’**	172–176
**^{1}H and ^{13}C for 17**	177–178
**^{1}H and ^{13}C for 18**	179–180
**^{1}H and ^{13}C for 18-H_2**	181–182
**^{1}H and ^{13}C for 19**	183–184
**^{1}H and ^{13}C for 23**	185–186
**^{1}H and ^{13}C for 24**	187–188
**^{1}H and ^{13}C for 25**	189–190
**^{1}H, ^{13}C, HSQC and HMBC for 29**	191–194
**^{1}H, ^{13}C, HSQC and HMBC for 30**	195–198
**^{1}H and ^{13}C for 31**	199–200
**^{1}H and ^{13}C for 36**	201–202
**^{1}H, ^{13}C, HSQC and HMBC for 37**	203–206
**^{1}H and ^{13}C for 38**	207–208
**^{1}H, ^{13}C and NOESY for 37-D**	209–211
**^{1}H, ^{13}C, HSQC, HMBC and NOESY for 38-D**	212–216

I. General Experimental Protocols

^{13}C and ^{1}H NMR spectra were recorded on a Bruker HD-500 spectrometer. Proton chemical shifts are referenced to residual $CHCl_3$ in (δ = 7.26 ppm) $CDCl_3$ solutions and to the residual CHD_5 (δ = 7.15 ppm) in benzene-d_6 solutions. A non-first order multiplet or doublet in a ^{1}H NMR spectrum is denoted as a 'nfom' or 'nfod', respectively. For the latter the coupling constant is given as an apparent value (J_{app}); the observed spacing between the two major lines is actually the value of $J_o + J_p$) and not the true doublet coupling. Resonances in the line listings in the ^{1}H NMR spectra are presented in the following format: chemical shift (ppm) [the multiplicity, the coupling constant(s) (in Hz), the integral value (to the nearest integer), and the assignment structure]. First-order coupling constants have been deduced using methods we have reported elsewhere.[1,2] The ^{13}C NMR chemical shifts are mostly taken from the "1D" spectra, although in some instances they were identified from the HSQC and/or HMBC data when the intensity in the 1D spectrum was not sufficiently high to allow a confident identification. Carbon chemical shifts are referenced to the carbon atom in $CDCl_3$ (δ = 77.16 ppm) or in C_6D_6 (δ = 128.06 ppm).

Infrared spectra were recorded in the attenuated total reflectance (ATR) mode using a Bruker Alpha II Spectrometer. Samples measured as thin films were deposited onto the diamond window by evaporation from an organic solvent, often $CDCl_3$. Some crystalline solids were placed directly on the window of the ATR sampling stage. Absorption maxima are given in cm^{-1}.

"The high-resolution **mass spectrometry** (HRMS) measurements were made in the ESI mode using a Thermo Orbitrap Velos instrument (mass accuracy of ≤3 ppm). An external calibrant was used (Pierce™ LTQ) and the samples were directly injected into the ion source.

Melting points are uncorrected and were determined on a Köfler hot-stage affixed to a polarizing microscope.

Medium pressure liquid chromatography (MPLC) was often used to purify newly synthesized materials. Hand-packed silica gel columns (normal-phase, 25-200 psi, 20-40 µm, 60 Å pore size, Teledyne RediSep Rf Gold®) were used. The apparatus consisted of a Waters HPLC pump (model 510), … and a Waters (R401) differential refractive index detector. **Preparative flash column chromatography** was performed on columns packed with Agela silica gel (230-400 mesh). **Thin layer chromatography** (TLC) was performed on silica-gel coated, plastic-backed plates that were visualized by UV light and/or by a solution of potassium permanganate and heating."[3]

Reaction temperatures refer to the temperature of a pre-equilibrated external heating oil bath. Reactions performed at temperatures higher than the that of the solvent's boiling point were done in a thread culture tube that was sealed with an inert, Teflon®-lined screw-cap.

II. General Procedures A–B

A. General procedure for the propargylic ester syntheses

A solution of the terminal alkyne in anhydrous THF (1.1 equiv 0.10 M) was charged to a flame-dried round-bottom flask closed with a septum. The flask was placed into a dry ice/acetone cooling bath and purged with N_2 gas. ^{n}BuLi (1.2 equiv, 2.5 M in hexane) was added dropwise through a syringe. The reaction mixture was stirred at -78 °C for 1 h. A solution of the aldehyde or ketone (1.0 equiv, 1.0 M in THF) was added to the solution. The reaction mixture was warmed to 0 °C over 2 h and recooled to -78 °C. The electrophile [1.4 equiv, MsCl, TFAA, or ClP(O)(OEt)$_2$] was added. After being warmed to room temperature and stirred for 3 h, the reaction mixture was quenched by the addition of sat. aq. NH_4Cl solution, extracted with EtOAc, dried ($MgSO_4$), concentrated, and purified by flash column chromatography.

B. General procedure for the allenyne generation

Under N_2 gas, a round-bottomed flask containing Pd[P(Ph$_3$)]$_4$ (0.05 equiv) and CuI (0.1 equiv) was placed into an ice bath. The propargylic ester (1.0 equiv, 0.05 M in THF) was added to the reaction vessel, followed by the sequential addition of 1.5 equiv of either triethylamine or diisopropylamine and, soon after, the terminal alkyne (1.05 equiv). The flask was capped with a septum and the reaction was stirred at room temperature for 20 h. The mixture was concentrated in vacuum and passed through a short column with silica (same eluant as for the subsequent MPLC purification). The eluted solution was concentrated in vacuum, and the residue was purified by MPLC.

III. Preparation Procedures and Characterization Data for all Compounds

(±)-1-(4-Methoxyphenyl)-5,8,8-trimethylnona-1,6-diyn-5-yl 2,2,2-trifluoroacetate (S1)

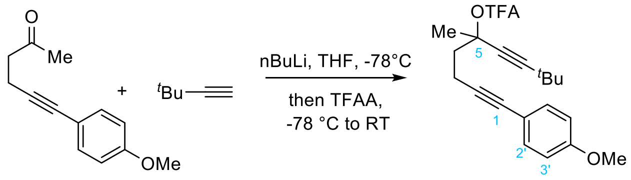

pre-**S1** **S1**

Compound **S1** was prepared following general procedure A and purified by flash column chromatography (15:1 hexanes/EtOAc). Use of ketone **pre–S1**[4] (202 mg, 1.0 mmol) resulted in the isolation of **S1** (317 mg, 83%) as a pale-yellow oil.

Data for S1

^{1}H NMR ($CDCl_3$, 500 MHz): δ 7.33 (nfod, J_{app} = 8.8 Hz, 2H, H2'+H6'), 6.82 (nfod, J_{app} = 8.8 Hz, 2H, H3'+H5'), 3.80 (s, 3H, OCH_3), 2.66–2.55 (m, 2H, C3H_2), 2.32 (ddd, J = 14.0, 9.2, 6.8 Hz, 1H, C4H_aH$_b$), 2.15 (ddd, J = 13.9, 9.2, 7.4 Hz, 1H, C4H$_a$$H_b$), 1.79 (s, 3H, C5Me), and 1.22 [s, 9H, C(CH_3)$_3$].

^{13}C{^{1}H} NMR ($CDCl_3$, 126 MHz): δ 159.3, 155.4 (q, J_{CF} = 42 Hz), 133.0, 115.9, 114.4 (q, J_{CF} = 287 Hz), 114.0, 97.8, 87.1, 80.9, 80.7, 75.6, 55.4, 41.0, 30.8, 27.6, 26.7, and 15.0.

HRMS (ESI) *m/z*: [M - OTFA]$^+$ Calcd for $C_{19}H_{23}O$ 267.1743; Found 267.1731.

IR (thin film): 3093, 2970, 2226, 1788, 1504, 1246, 1219, and 1147 cm^{-1}.

(±)-1-(2-((4-Chlorophenyl)ethynyl)phenyl)-4,4-dimethylpent-2-yn-1-yl Diethyl Phosphate (S2)

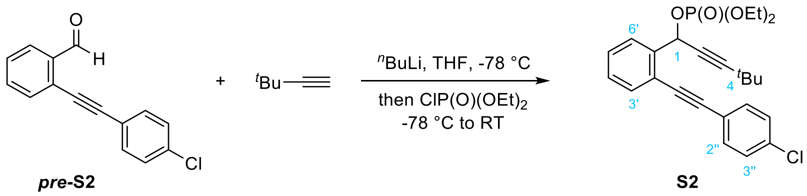

***pre*-S2** **S2**

Compound **S2** was prepared following general procedure A and purified by flash column chromatography (3:2 hexanes/EtOAc). Use of the aldehyde ***pre*-S2** (202 mg, 1.0 mmol) resulted in the isolation of **S2** (1.73 g, 83%) as a clear oil.

Data for S2

^{1}H NMR ($CDCl_3$, 500 MHz): δ 7.79 (dd, J = 7.7, 1.5 Hz, 1H, H6'), 7.63 (nfod, J_{app} = 8.4 Hz, 2H, H2''+H6''), 7.54 (dd, J = 7.7, 1.5 Hz, 1H, H3'), 7.40 (ddd, J = 7.6, 7.6, 1.6 Hz, 1H, H4' or H5'), 7.35 (ddd, J = 7.6, 7.6, 1.6 Hz, 1H, H4' or H5'), 7.33 (nfod, J_{app} = 8.5 Hz, 2H, H3''+H5''), 6.58 (d, $^{3}J_{P\text{–}H}$ = 7.3 Hz, 1H, H1), 4.24–4.08 (m, 2H, $OCH_aH_bCH_3$ and $O'C'H_aH_bC'H_3$), 4.03–3.95 (m, 2H, $OCH_aH_bCH_3$ and $O'C'H_aH_bC'H_3$), 1.33 (td, J = 7.1, 1.1 Hz, 3H, OCH_2CH_3), 1.24 [s, 9H, $C(CH_3)_3$], and 1.16 (td, J = 7.1, 1.1 Hz, 3H, $O'C'H_2C'H_3$).

^{13}C{^{1}H} NMR ($CDCl_3$, 126 MHz): δ 139.6 (d, $^{3}J_{c\text{–}p}$ = 7.3 Hz), 134.7, 133.3, 132.3, 128.98, 128.97, 128.8, 128.0, 122.3, 121.6, 97.7, 94.0, 87.4, 75.5 (d, $^{3}J_{c\text{–}p}$ = 3.6 Hz), 68.2 (d, $^{2}J_{c\text{–}p}$ = 5.1 Hz), 63.80 (d, $^{2}J_{c\text{–}p}$ = 5.8 Hz), 63.78 (d, $^{2}J_{c\text{–}p}$ = 5.8 Hz), 30.8, 27.7, 16.2 (d, $^{3}J_{c\text{–}p}$ = 7.2 Hz), and 16.1 (d, $^{3}J_{c\text{–}p}$ = 7.1 Hz).

HRMS (ESI) m/z: $[M + H]^+$ Calcd for $C_{25}H_{29}{}^{35}ClO_4P$ 459.1487; Found 459.1492 (51%); $[M - (EtO)_2PO_2]^+$ Calcd for $C_{21}H_{18}{}^{35}Cl$ 305.1092; Found 305.1096 (100%).

IR (thin film): 3087, 2973, 2908, 2240, 1492, 1228, and 1035 cm^{-1}.

(±)-5-Cyano-5-methyl-1-(trimethylsilyl)hex-1-yn-3-yl Methanesulfonate (S3)

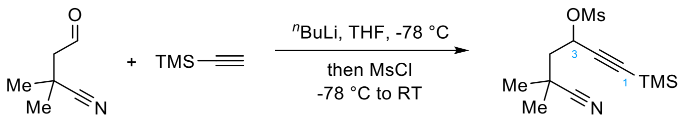

***pre*-S3** **S3**

Compound **S3** was prepared following general procedure A and purified by flash column chromatography (3:1 hexanes/EtOAc). Use of the aldehyde ***pre*-S3** (202 mg, 1.0 mmol) resulted in the isolation of **S3** (1.28 g, 83%) as a clear oil.

Data for S3

^{1}H NMR ($CDCl_3$, 500 MHz): δ 5.35 (dd, *J* = 8.2, 4.8 Hz, 1H, H3), 3.16 (s, 3H, OSO_2CH_3), 2.25 (dd, *J* = 14.8, 8.2 Hz, 1H, C4H_aH_b), 2.10 (dd, *J* = 14.8, 4.8 Hz, 1H, C4H_aH_b), 1.47 [s, 6H, $C(CH_3)_2$], and 0.20 [s, 9H, $Si(CH_3)_3$].

^{13}C{^{1}H} NMR ($CDCl_3$, 126 MHz): δ 123.8, 99.4, 96.3, 68.4, 45.3, 39.5, 30.6, 27.11, 27.07, and -0.5.

HRMS (ESI) *m/z*: $[M + H]^+$ Calcd for $C_{12}H_{22}NO_3SSi$ 288.1084; Found 288.1077 (2%); $[M – OMs]^+$ Calcd for $C_{11h18}NSi$ 192.1203; Found 192.1195 (100%).

IR (thin film): 2963, 2238, 1364, and 1177 cm^{-1}.

(±)-1-(2-Cyanophenyl)-4,4-dimethylpent-2-yn-1-yl Diethyl Phosphate (S4)

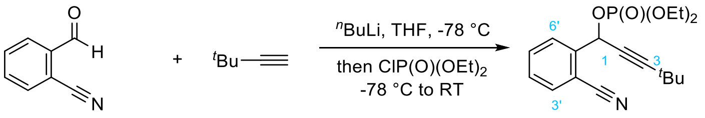

***pre*-S4** **S4**

Compound **S4** was prepared following general procedure A and purified by flash column chromatography (3:1 hexanes/EtOAc). Use of the aldehyde ***pre*-S4** (655 mg, 5.0 mmol) resulted in the isolation of **S4** (1.43 g, 83%) as a clear oil.

Data for S4

^{1}H NMR ($CDCl_3$, 500 MHz): δ 7.74 (dd, *J* = 8.0, 1.4, 1H, H6'), 7.68 (dd, *J* = 7.7, 1.4, H3'), 7.62 (ddd, *J* = 7.7, 7.7, 1.4 Hz, 1H, H5'), 7.45 (ddd, *J* = 7.6, 7.6, 1.3 Hz, 1H, H4'), 6.20 (d, $J_{P\text{-}H}$ = 7.5 Hz, 1H, H1), 4.23–4.05 (m, 4H, CH_2CH$_3$ and CH_2'CH3'), 1.33 (ddd, *J* = 7.0, 7.0, 1.2 Hz, 3H, CH$_2$CH_3), 1.29 (ddd, *J* = 7.1, 7.1, 1.1 Hz, 3H, CH$_2$'CH$_3$'), and 1.23 [s, 9H, C(CH$_3$)$_3$].

^{13}C{^{1}H} NMR ($CDCl_3$, 126 MHz): δ 141.8 (d, $^3J_{c\text{–}p}$ = 6.6 Hz), 133.6, 133.2, 129.3, 128.5, 116.8, 111.4, 98.9, 74.4 (d, $^3J_{c\text{–}p}$ = 5.3 Hz), 67.7 (d, $^2J_{c\text{–}p}$ = 5.0 Hz), 64.3 (d, $^2J_{c\text{–}p}$ = 6.0 Hz), 64.2 (d, $^2J_{c\text{–}p}$ = 5.9 Hz), 30.6, 27.8, 16.170 (d, $^3J_{c\text{–}p}$ = 7.2 Hz), and 16.166 (d, $^3J_{c\text{–}p}$ = 6.9 Hz).

HRMS (ESI) *m/z*: [M + H]$^+$ Calcd for $C_{18}H_{25}NO_4P$ 350.1516; Found 350.1509 (100%).

IR (thin film): 3070, 2973, 2240, 1272, 1029, and 1018 cm^{-1}.

8-(4-Methoxyphenyl)octa-5,7-diynal (S5)

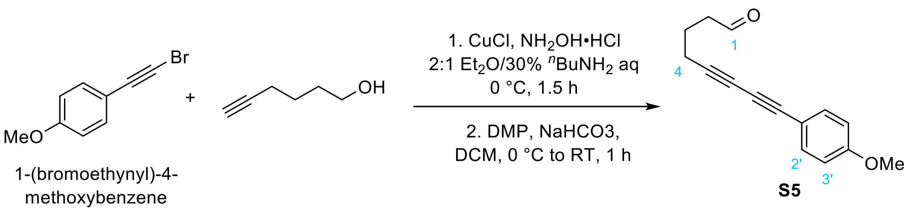

A round-bottomed flask containing CuCl (50 mg, 0.1 equiv) and hydroxylamine hydrochloride (210 mg, 0.3 equiv) was placed into an ice bath. *n*-$BuNH_2$ (4.5 mL) in water (10.5 mL) was added to the flask. Hex-5-yn-1-ol (590 mg, 6.0 mmol, 1.2 equiv) was added followed by the dropwise addition of a solution of 1-(bromoethynyl)-4-methoxybenzene (1.11 g, 5 mmol, 1.0 equiv) in Et_2O[5] (5 mL). This reaction mixture was stirred at 0 ℃ for 1.5 h, quenched by addition of satd aqueous NH_4Cl, extracted with Et_2O, dried ($MgSO_4$), concentrated, passed through a short column with 1:1 hexanes/EtOAc as eluant, concentrated again to produce a diynyl alcohol precursor of **S5**. This crude material was dissolved in DCM (30 mL) at 0 ℃. DMP (2.12 g, 5.0 mmol) and solid $NaHCO_3$ (0.84 g, 10 mmol) were added. After being stirred for 1 h, the reaction mixture was quenched by addition of satd aq Na_2SO_3 solution, extracted with DCM, dried ($MgSO_4$), concentrated, and purified by flash column chromatography (4:1 hexanes/EtOAc) to provide **S5** (517 mg, 46% from the 1-bromoalkyne) as a yellow oil.

Data for S5

^{1}H NMR ($CDCl_3$, 500 MHz): δ 9.81 (t, J = 1.2 Hz, 1H, CHO), 7.41 (nfod, J_{app} = 9.0 Hz, 2H, H2'+H6'), 6.82 (nfod, J_{app} = 8.9 Hz, 2H, H3'+H5'), 3.81 (s, 3H, OCH_3), 2.64 [td, J = 7.2, 1.3 Hz, 2H, $(H2)_2$], 2.45 [t, J = 7.0 Hz, 2H, $(H4)_2$], and 1.90 [tt, J = 7.1, 7.1 Hz, 2H, $(H3)_2$].

^{13}C{^{1}H} NMR ($CDCl_3$, 126 MHz): δ 201.6, 160.3, 134.3, 114.2, 113.9, 82.5, 75.6, 72.9, 66.5, 55.5, 42.7, 20.9, and 19.1.

HRMS (ESI) *m/z*: $[M + H]^+$ Calcd for $C_{15}H_{15}O_2$ 227.1067; Found 227.1062.

IR (thin film): 3007, 2938, 2862, 2741, 2253, 2150, 1722, 1603, 1509, and 1250 cm^{-1}.

(±)-12-(4-Methoxyphenyl)-2,2-dimethyldodeca-3,9,11-triyn-5-yl Methanesulfonate (S6)

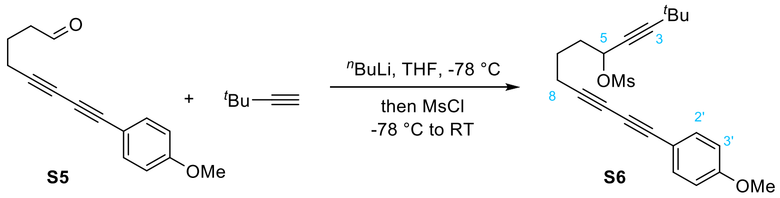

Compound **S6** was prepared following general procedure A and purified by flash column chromatography (3:1 hexanes/EtOAc). Use of the aldehyde **S5** (226 mg, 1.0 mmol) resulted in the isolation of **S4** (323 mg, 84%) as a yellow oil.

Data for S6

^{1}H NMR ($CDCl_3$, 500 MHz): δ 7.42 (nfod, J_{app} = 8.8 Hz, 2H, H2'+H6'), 6.83 (nfod, J_{app} = 8.8 Hz, 2H, H3'+H5'), 5.18 (dd, J = 6.4, 6.4 Hz, 1H, H5), 3.81 (s, 3H, OCH_3), 3.12 (s, 3H, OSO_2CH_3), 2.44 [t, J = 6.7 Hz, 1H, $(H8)_2$], 2.04–1.95 [m, 2H, $(H6)_2$ or $(H7)_2$], and 1.84–1.60 [m, 2H, $(H6)_2$ or $(H7)_2$], and 1.24 [s, 9H, $C(CH_3)_3$].

^{13}C{^{1}H} NMR ($CDCl_3$, 126 MHz): δ 160.3, 134.2, 114.2, 114.0, 98.6, 82.8, 75.4, 74.3, 73.1, 72.4, 66.2, 55.5, 39.3, 35.1, 30.7, 27.7, 23.8, and 19.2.

HRMS (ESI) *m/z*: $[M + H]^+$ Calcd for $C_{22}H_{27}O_4S$ 387.1625; Found 387.1628 (17%).

IR (thin film): 3055, 2972, 2885, 2183, 1604, 1510, 1364, 1250, and 1174 cm^{-1}.

(±)-7-(4-Methoxyphenyl)-1-(trimethylsilyl)hepta-1,6-diyn-3-yl Methanesulfonate (10a)

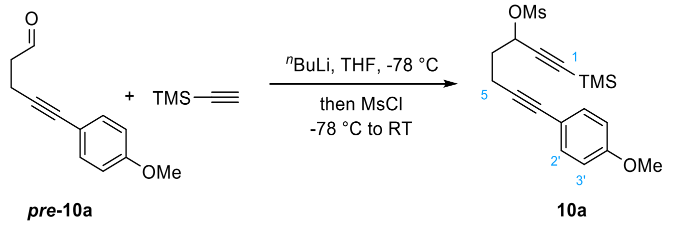

Compound **10a** was prepared following general procedure A and purified by flash column chromatography (3:1 hexanes/EtOAc). Use of the aldehyde ***pre*-10a** (940 mg, 5.0 mmol) resulted in the isolation of **10a** (1.32 g, 72%) as a clear oil.

Data for 10a

^{1}H NMR ($CDCl_3$, 500 MHz): δ 7.34 (nfod, J_{app} = 8.8 Hz, 2H, H2'+H6'), 6.82 (nfod, J_{app} = 8.8 Hz, 2H, H3'+H5'), 5.36 (dd, J = 7.5, 5.6 Hz, 1H, H3), 3.80 (s, 3H, OCH_3), 3.14 (s, 3H, OSO_2CH_3), 2.61 (ddd, J = 18.3, 7.6, 7.6 Hz, 1H, C5H_aH$_b$), 2.60 (ddd, J = 18.1, 7.1, 7.1 Hz, 1H, C5H$_a$$H_b$), 2.20 (dddd, J = 14.0, 7.4, 7.4, 6.6 Hz, 1H, C4H_aH$_b$), 2.15 (dddd, J = 13.9, 7.4, 7.4, 5.5 Hz, 1H, C4H$_a$$H_b$), and 0.20 [s, 9H, $Si(CH_3)_3$].

^{13}C{^{1}H} NMR ($CDCl_3$, 126 MHz): δ 159.4, 133.1, 115.6, 114.0, 100.0, 95.2, 85.8, 81.9, 71.1, 55.4, 39.3, 35.0, 15.5, and -0.3.

HRMS (ESI) *m/z*: $[M + H]^+$ Calcd for $C_{18}H_{25}O_4SSi$ 365.1237; Found 365.1231 (54%).

IR (thin film): 3015, 2960, 2178, 1606, 1362, 1247, and 1174 cm^{-1}.

(±)-(1,9-*bis*(4-Methoxyphenyl)nona-3,4-dien-1,8-diyn-3-yl)trimethylsilane (11a)

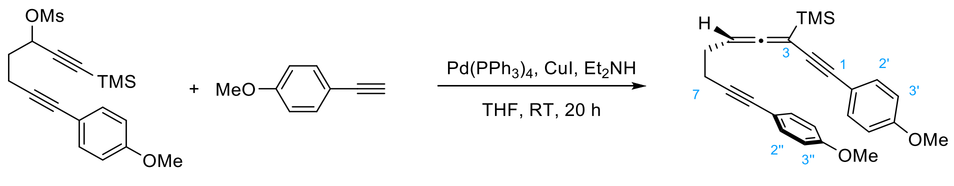

Compound **11a** was prepared following general procedure B and purified by MPLC (15:1 hexanes/EtOAc). Use of the mesylate **10a** (364 mg, 1.0 mmol) resulted in the isolation of **11a** (164 mg, 44%) as a yellow oil. (ca. $t_{1/2}$ = 35 min @80 ℃, see p S19)

Data for 11a

^{1}H NMR ($CDCl_3$, 500 MHz): δ 7.34 (nfod, J_{app} = 8.8 Hz, 2H, H2'+H6' or H2''+H6''), δ 7.33 (nfod, J_{app} = 8.8 Hz, 2H, H2'+H6' or H2''+H6''), 6.82 (nfod, J_{app} = 8.8 Hz, 2H, H3'+H5' or H3''+H5''), 6.77 (nfod, J_{app} = 8.8 Hz, 2H, H3'+H5' or H3''+H5''), 5.27 (dd, J = 6.7, 6.7 Hz, 1H, H5), 3.80 (s, 3H, 4'OCH_3 or 4''OCH_3), 3.78 (s, 3H, 4'OCH_3 or 4''OCH_3), 2.54 (ddd, J = 16.8, 7.9, 7.0 Hz, 1H, C7H_aH$_b$), 2.51 (ddd, J = 16.7, 7.0, 7.0 Hz, 1H, C7H$_a$$H_b$), 2.37 (dddd, J = 14.5, 6.9, 6.9, 6.9 Hz, 1H, C6H_aH$_b$), 2.35 (dddd, J = 14.6, 7.8, 6.9, 6.9 Hz, 1H, C6H$_a$$H_b$), and 0.22 [s, 9H, $Si(CH_3)_3$].

^{13}C{^{1}H} NMR ($CDCl_3$, 126 MHz): δ 212.4, 159.4, 159.2, 133.1, 133.0, 116.5, 116.1, 114.0, 113.9, 91.4, 87.9, 85.8, 85.0, 82.7, 81.1, 55.42, 55.36, 27.7, 19.8, and -1.6.

HRMS (ESI) *m/z*: $[M + H]^+$ Calcd for $C_{26}H_{29}O_2Si$ 401.1931; Found 401.1925.

IR (thin film): 3039, 2956, 2190, 1927, 1605, 1509, and 1247 cm^{-1}.

(±)-Triethyl(9-(4-methoxyphenyl)-3-(trimethylsilyl)nona-3,4-dien-1,8-diyn-1-yl)silane (11b)

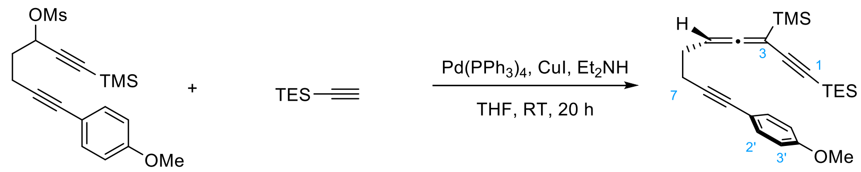

10a **11b**

Compound **11b** was prepared following general procedure B and purified by MPLC (30:1 hexanes/EtOAc). Use of the mesylate **10a** (364 mg, 1.0 mmol) resulted in the isolation of **11b** (273 mg, 67%) as a yellow oil.

Data for 11b

^{1}H NMR ($CDCl_3$, 500 MHz): δ 7.33 (nfod, J_{app} = 8.8 Hz, 2H, H2'+H6'), 6.81 (nfod, J_{app} = 8.8 Hz, 2H, H3'+H5'), 5.21 (dd, J = 6.8, 6.8 Hz, 1H, H5), 3.80 (s, 3H, 4'OCH_3), 2.50 (ddd, J = 16.7, 7.9, 6.9 Hz, 1H, C7$H_a$$H_b$), 2.48 (dddd, J = 14.0, 6.4, 6.4, 6.4 Hz, 1H, $CH_a$$H_b$), 2.39–2.28 (nfom, 2H, C6H_2), 0.99 [t, J = 7.9 Hz, 9H, $Si(CH_2CH_3)_3$], and 0.60 [q, J = 7.9 Hz, 6H, $Si(CH_2CH_3)_3$], and 0.17 [s, 9H, $Si(CH_3)_3$].

^{13}C{^{1}H} NMR ($CDCl_3$, 126 MHz): δ 212.8, 159.2, 133.1, 116.1, 113.9, 100.8, 93.7, 87.8, 85.6, 85.1, 81.1, 55.4, 27.5, 19.8, 7.7, 4.7, and -1.8.

HRMS (ESI) *m/z*: $[M + H]^+$ Calcd for $C_{25}H_{37}O_2Si$ 409.2377; Found 409.2368 (100%).

IR (thin film): 3040, 2999, 2936, 2875, 2126, 2043, 1894, 1610, 1508, and 1246 cm^{-1}.

(±)-4,4'-(3-(*tert*-Butyl)-5-methylnona-3,4-dien-1,8-diyne-1,9-diyl)*bis*(methoxybenzene) (11c)

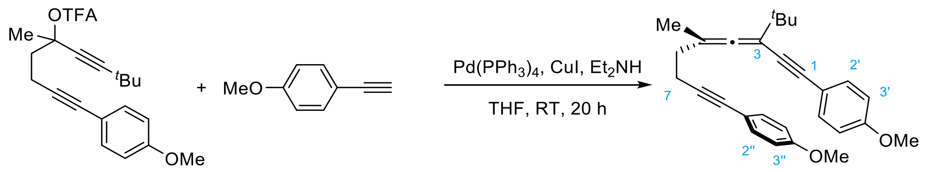

S1 **11c**

Compound **11c** was prepared following general procedure B and purified by MPLC (15:1 hexanes/EtOAc). Use of the trifluoroacetate **S1** (190 mg, 0.50 mmol) resulted in the isolation of **11c** (147 mg, 74%) as a clear oil.

Data for 11c

^{1}H NMR ($CDCl_3$, 500 MHz): δ 7.34 (nfod, J_{app} = 8.8 Hz, 2H, H2'+H6' or H2''+H6''), 7.32 (nfod, J_{app} = 8.8 Hz, 2H, H2'+H6' or H2''+H6''), 6.81 (nfod, J_{app} = 8.8 Hz, 2H, H3'+H5' or H3''+H5''), 6.76 (nfod, J_{app} = 8.8 Hz, 2H, H3'+H5' or H3''+H5''), 3.80 (s, 3H, 4'OCH_3 or 4''OCH_3), 3.77 (s, 3H, 4'OCH_3 or 4''OCH_3), 2.55 (ddd, *J* = 16.9, 8.7, 6.9 Hz, 1H, C7H_aH$_b$), 2.52 (ddd, *J* = 16.4, 8.1, 6.6 Hz, 1H, C7H$_a$$H_b$), 2.36 (ddd, *J* = 15.4, 7.1, 6.9 Hz, 1H, C6H_aH$_b$), 2.31 (ddd, *J* = 15.3, 8.8, 6.9 Hz, 1H, C6H$_a$$H_b$), 1.80 (s, 3H, C5$CH_3$), and 1.17 [s, 9H, $C(CH_3)_3$].

^{13}C{^{1}H} NMR ($CDCl_3$, 126 MHz): δ 204.1, 159.3, 159.1, 133.0, 132.9, 116.4, 116.3, 113.91, 113.89, 101.8, 101.5, 91.3, 88.4, 83.6, 80.7, 55.4, 55.3, 35.2, 33.6, 29.5, 19.4, and 18.1.

HRMS *m/z*: $[M + H]^+$ Calcd for $C_{28}H_{31}O_2$ 399.2319; Found 399.2291 (100%).

IR (thin film): 3038, 2961, 2176, 1907, 1605, 1509, and 1246 cm^{-1}.

(±)-1-(3-(*tert*-Butyl)-5-(4-methoxyphenyl)penta-1,2-dien-4-yn-1-yl)-2-((4-chlorophenyl)ethynyl)benzene (11d)

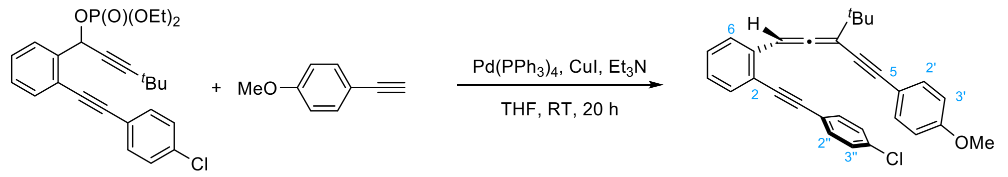

S2 **11d**

Compound **11d** was prepared following general procedure B and purified by MPLC (25:1 hexanes/EtOAc). Use of the phosphate **S2** (459 mg, 1.0 mmol) resulted in the isolation of **11d** (92 mg, 21%) as a marginally stable, orange oil. (ca. $t_{1/2}$ = 8 h @rt, see p S23)

Data for 11d

^{1}H NMR ($CDCl_3$, 500 MHz): δ 7.51 (dd, *J* = 7.4, 1.5 Hz, 1H, H3), 7.49 (nfod, J_{app} = 8.6 Hz, 2H, H2''+H6''), 7.48 (overlapped peaks, 1H, H6), 7.38 (nfod, J_{app} = 8.9 Hz, 2H, H2'+H6'), 7.33 (nfod, J_{app} = 8.6 Hz, 2H, H3''+H5''), 7.31 (dd, *J* = 7.5, 7.5, 1.5 Hz, 1H, H4 or H5), 7.20 (dd, *J* = 7.5, 7.5, 1.5 Hz, 1H, H4 or H5), 7.06 (s, 1H, allene H), 6.84 (nfod, J_{app} = 8.9 Hz, 2H, H3'+5'), 3.81 (s, 3H, OMe), and 1.27 [s, 9H, $C(CH_3)_3$].

^{13}C{^{1}H} NMR The allenyne **11d** underwent full decomposition (tlc analysis) within 48 h when held at ambient temperature as a $CDCl_3$ solution, so carbon NMR data were of low quality.

HRMS (ESI) *m/z*: $[M + H]^+$ Calcd for $C_{30}H_{25}{}^{35}ClO$ 437.1667; Found 437.1646 (28%).

IR (thin film): 3065, 2963, 2208, 1938, 1605, 1492, and 1248 cm^{-1}.

(±)-1-(3-(*tert*-Butyl)octa-1,2-dien-4-yn-1-yl)-2-((4-chlorophenyl)ethynyl)benzene (11e)

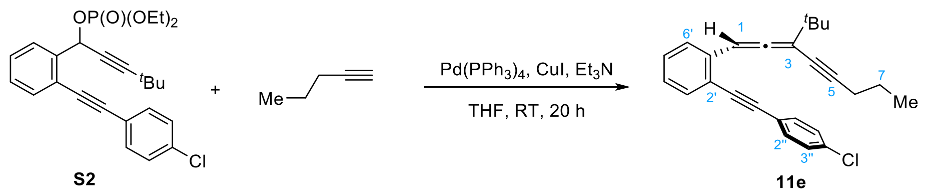

Compound **11e** was prepared following general procedure B and purified by MPLC (hexanes). Use of the phosphate **S2** (459 mg, 1.0 mmol) resulted in the isolation of **11e** (172 mg, 46%) as a yellow oil.

Data for 11e

^{1}H NMR ($CDCl_3$, 500 MHz): δ 7.54 (overlapped peaks, 3H, H3'+H3''+H5''), 7.43 (dd, J = 7.9, 1.3 Hz, 1H, H6'), 7.33 (nfod, J_{app} = 8.5 Hz, 2H, H2''+H6''), 7.28 (ddd, J = 7.5, 7.5 1.5 Hz, 1H, H4' or H5'), 7.18 (ddd, J = 7.6, 7.6 1.4 Hz, 1H, H4' or H5'), 6.98 (t, J = 1.2 Hz, 1H, H1), 2.33 [td, J = 7.0, 1.2 Hz, 2H, $(H6)_2$], 1.50 [qd, J = 7.5, 7.5 Hz, 2H, $(H7)_2$], 1.20 [s, 9H, $C(CH_3)_3$], and 1.01 [t, J = 7.5, 3H, $(H8)_3$].

^{13}C{^{1}H} NMR ($CDCl_3$, 126 MHz): δ 210.4, 136.0, 134.5, 132.9, 132.6, 128.90, 128.86, 127.0, 126.7, 121.9, 121.1, 105.7, 95.2, 94.8, 93.1, 88.7, 73.6, 35.7, 29.3, 22.4, 21.8, and 13.7.

HRMS (ESI) *m/z*: $[M + H]^+$ Calcd for $C_{25}H_{26}{}^{35}Cl$ 373.1718; Found 373.1702 (100%).

IR (thin film): 3066, 2962, 2903, 2214, 1930, 1594, 1490, and 1091 cm^{-1}.

(±)-8-(4-Methoxyphenyl)-2,2-dimethyl-6-(trimethylsilyl)octa-4,5-dien-7-ynenitrile (11f)

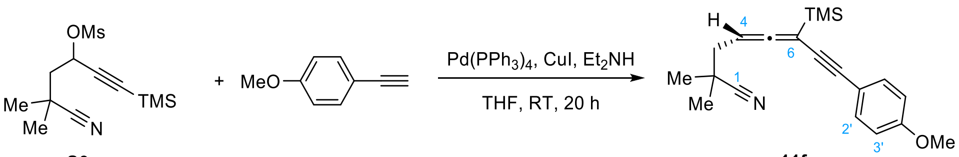

Compound **11f** was prepared following general procedure B and purified by MPLC (4:1 hexanes/EtOAc). Use of the mesylate **S3** (287 mg, 1.0 mmol) resulted in the isolation of **11f** (274 mg, 85%) as a yellow oil.

Data for 11f

^{1}H NMR ($CDCl_3$, 500 MHz): δ 7.35 (nfod, J_{app} = 8.8 Hz, 2H, H2'+H6'), 6.83 (nfod, J_{app} = 8.8 Hz, 2H, H3'+H5'), 5.19 (dd, *J* = 7.8 Hz, 1H, H4), 3.81 (s, 3H, OCH_3), 2.32 (dd, *J* = 14.2, 8.0 Hz, 1H, C3H_aH$_b$), 2.31 (dd, *J* = 14.0, 7.8 Hz, 1H, C3H$_a$$H_b$), 1.41 [s, 3H, C2($CH_3$)$_a$($CH_3$)$_b$], 1.40 [s, 3H, C2($CH_3$)$_a$($CH_3$)$_b$], and 0.24 [s, 9H, Si($CH_3$)$_3$].

^{13}C{^{1}H} NMR ($CDCl_3$, 126 MHz): δ 213.6, 159.5, 133.0, 124.7, 116.3, 114.0, 92.1, 84.9, 81.9, 81.3, 55.4, 40.2, 32.7, 26.6, 26.2, and -1.6.

HRMS (ESI) *m/z*: $[M + H]^+$ Calcd for $C_{20}H_{26}NOSi$ 324.1778; Found 324.1770 (63%).

IR (thin film): 3047, 2963, 2244, 2186, 1907, 1606, and 1238 cm^{-1}.

(±)-2-(3-(*tert*-Butyl)-5-(4-methoxyphenyl)penta-1,2-dien-4-yn-1-yl)benzonitrile (11g)

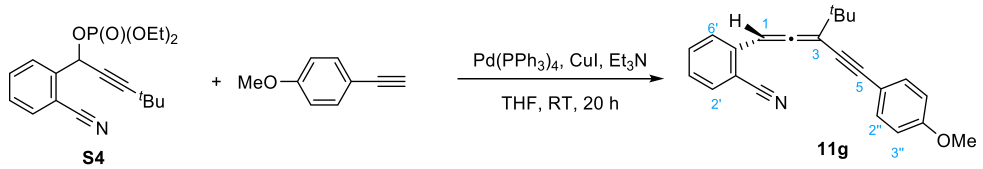

Compound **11g** was prepared following general procedure B and purified by MPLC (4:1 hexanes/EtOAc). Use of the phosphate **S4** (349 mg, 1.0 mmol) resulted in the isolation of **11g** (269 mg, 82%) as an orange oil. (ca. $t_{1/2}$ = 3 h @145 ℃, see p S 26)

Data for 11g

^{1}H NMR ($CDCl_3$, 500 MHz): δ 7.62 (ddd, *J* = 7.9, 1.1, 1.1 Hz, 1H, 1H, H3’), 7.55–7.51 (m, 2H, H6’+H5’), 7.40 (nfod, J_{app} = 8.8 Hz, 2H, H2’’+H6’’), 7.29 (nfom, 1H, H4’), 6.85 (nfod, J_{app} = 8.8 Hz, 2H, H3’’+H5’’), 6.84 (s, 1H, H1), 3.81 (s, 3H, OCH_3), and 1.27 [s, 9H, $C(CH_3)_3$].

^{13}C{^{1}H} NMR ($CDCl_3$, 126 MHz): δ 211.6, 159.8, 138.0, 133.13, 133.08, 132.9, 127.5, 127.4, 117.8, 115.5, 114.1, 110.6, 107.0, 94.7, 94.5, 80.4, 55.4, 36.0, and 29.4.

HRMS (ESI) *m/z*: $[M + H]^+$ Calcd for $C_{23}H_{22}NO$ 328.1696; Found 328.1688 (100%).

IR (thin film): 3066, 2965, 2223, 1898, 1604, 1509, and 1285 cm^{-1}.

(±)-1-(10-(*tert*-Butyl)-12-(4-chlorophenyl)dodeca-8,9-dien-1,3,11-triyn-1-yl)-4-methoxybenzene (11h)

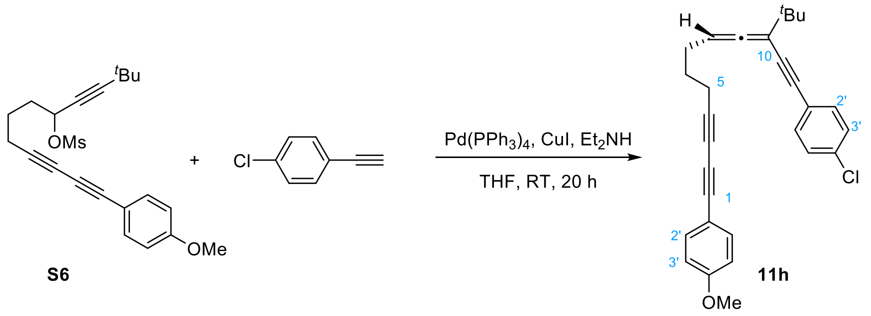

S6 **11h**

Compound **11g** was prepared following general procedure B and purified by MPLC (25:1 hexanes/EtOAc). Use of the mesylate **S6** (386 mg, 1.0 mmol) resulted in the isolation of **11h** (260 mg, 61%) as an amorphous white solid.

Data for 11h

^{1}H NMR ($CDCl_3$, 500 MHz): δ 7.40 (nfod, J_{app} = 8.9 Hz, 2H, H2'+H6'), 7.36 (nfod, J_{app} = 8.5 Hz, 2H, H3''+H5''), 7.27 (nfod, J_{app} = 8.5 Hz, 2H, H2''+H6''), 6.81 (nfod, J_{app} = 8.8 Hz, 2H, H3'+H5'), 5.45 (dd, *J* = 6.6, 6.6 Hz, 1H, H8), 3.79 (s, 3H, 4'OCH_3), 2.45 [t, *J* = 6.9 Hz, 2H, $(H5)_2$], 2.27–2.20 [m, 2H, $(H7)_2$], 1.73 [tt, *J* = 7.0, 7.0 Hz, 2H, $(H6)_2$], and 1.18 [s, 9H, $C(CH_3)_3$].

^{13}C{^{1}H} NMR ($CDCl_3$, 126 MHz): δ 207.5, 160.2, 134.2, 133.9, 132.7, 128.7, 122.4, 114.2, 114.1, 101.5, 93.4, 91.2, 85.5, 83.5, 75.2, 73.2, 65.9, 55.4, 34.6, 29.4, 27.9, 27.5, and 19.2.

HRMS (ESI) *m/z*: $[M + H]^+$ Calcd for $C_{29}H_{28}{}^{35}ClO$ 427.1823; Found 427.1828 (41%).

IR (thin film): 3071, 2988, 2930, 2255, 2199, 1942, 1600, 1488, and 1207 cm^{-1}.

(±)-4,5-*bis*(4-Methoxyphenyl)-7-(trimethylsilyl)-2,3-dihydro-1*H*-inden-1-yl Acetate (12a)

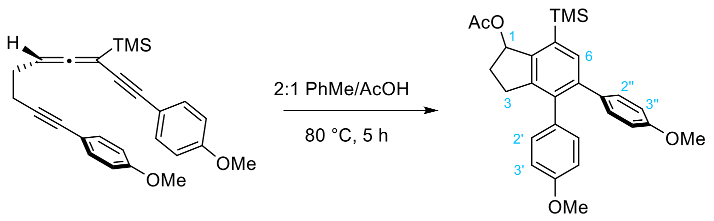

11a **12a**

Allenyne **11a** (10.0 mg, 0.025 mmol) was dissolved in a 2:1 mixture (vol ratio) of PhMe/AcOH (1.5 mL) in a threaded culture tube. The tube was capped and heated at 80 ℃ for 5 h. After the reaction solution was concentrated, the residue was passed through a pipet column of silica gel (elution with 3:1 hexanes/EtOAc) and the filtrate was reconcentrated. The residue was purified by MPLC (12:1 hexanes/EtOAc) to provide **12a** (9.1 mg, 79%) as a clear oil.

Data for 12a

^{1}H NMR ($CDCl_3$, 500 MHz): δ 7.48 (s, 1H, H6), 7.02 (nfod, J_{app} = 8.8 Hz, 2H, H2''+H6''), 7.03–6.97 (b, 2H, H2'+H6'), 6.77 (nfod, J_{app} = 8.1 Hz, 2H, H3'+H5'), 6.73 (nfod, J_{app} = 8.8 Hz, 2H, H3''+H5''), 6.24 (dd, *J* = 6.3, 2.0 Hz, 1H, H1), 3.78 (s, 3H, 4'OCH_3 or 4''OCH_3), 3.76 (s, 3H, 4'OCH_3 or 4''OCH_3), 3.02 (ddd, *J* = 16.2, 8.8, 7.9 Hz, 1H, C3H_aH$_b$), 2.66 (ddd, *J* = 16.4, 8.8, 2.8 Hz, 1H, C3H$_a$$H_b$), 2.42 (dddd, *J* = 14.4, 8.8, 8.4, 6.4 Hz, 1H, C2H_aH$_b$), 2.11 (dddd, *J* = 14.4, 8.2, 2.8, 2.0 Hz, 1H, C2H$_a$$H_b$), 2.10 (s, 3H, $OCOCH_3$), and 0.33 [s, 9H, $Si(CH_3)_3$].

^{13}C{^{1}H} NMR ($CDCl_3$, 126 MHz): δ 171.2, 158.28, 158.27, 144.9, 144.1, 140.8, 138.0, 136.4, 135.8, 134.1, 132.0, 131.2, 131.1, 113.5, 113.4, 80.0, 55.30, 55.27, 33.0, 30.4, 21.8, and 0.0.

HRMS (ESI) *m/z*: [M - OAc]$^+$ Calcd for $C_{26}H_{29}O_2Si$ 401.1931; Found 401.1912 (100%).

IR (thin film): 3035, 2952, 1733, 1609, 1515, and 1245 cm^{-1}.

Half-life measurement: In another experiment, **11a** was heated under the same conditions as the procedure described above. After 60 minutes, the reaction was cooled and concentrated. The ^{1}H NMR spectrum of the crude residue showed an ca. 3:1 ratio of **12a**:**11a**, indicating an approximate half-life for the cyclization of $t_{1/2}$ = 30 min.

(±)-4-(4-Methoxyphenyl)-5-(triethylsilyl)-7-(trimethylsilyl)-2,3-dihydro-1*H*-inden-1-yl Acetate (12b)

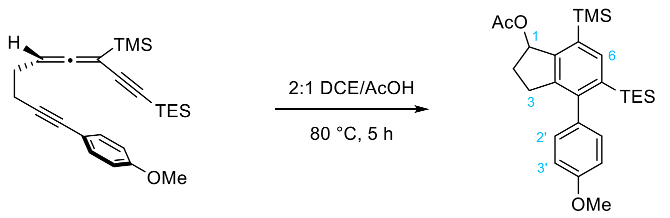

11b **12b**

Allenyne **11b** (10.0 mg, 0.025 mmol) was dissolved in a 2:1 mixture (vol ratio) of DCE/AcOH (1.5 mL) in a threaded culture tube. The tube was capped and heated at 80 ℃ for 5 h. After the reaction solution was concentrated, the residue was passed through a pipet column of silica gel (elution with 5:1 hexanes/EtOAc) and the filtrate was reconcentrated. The residue was purified by MPLC (16:1 hexanes/EtOAc) to provide **12b** (8.0 mg, 71%) as a clear oil.

Data for 12b

^{1}H NMR ($CDCl_3$, 500 MHz): δ 7.66 (s, 1H, H6), 7.14 (nfom, 1H, H2' or H6'), 7.08 (nfom, 1H, H2' or H6'), 6.92–6.89 (overlapping nfom's, 2H, H3'+H5'), 6.20 (dd, *J* = 6.6, 2.5 Hz, 1H, H1) 3.86 (s, 3H, 4'OCH_3), 2.76 (ddd, *J* = 16.2, 7.9, 7.9 Hz, 1H, C3H_aH_b), 2.49 (ddd, *J* = 16.4, 8.9, 3.4 Hz, 1H, C3H_aH_b), 2.39 (dddd, *J* = 14.4 8.9, 7.4, 6.7 Hz, 1H, C2H_aH_b), 2.08 (s, 3H, $OCOCH_3$) 1.98 (dddd, *J* = 14.0, 8.2, 5.8, 2.4 Hz, 1H, C2H_aH_b), 0.82 [t, *J* = 7.9 Hz, 9H, $Si(CH_2CH_3)_3$], and 0.45 [q, *J* = 7.9 Hz, 6H, $Si(CH_2CH_3)_3$], and 0.32 [s, 9H, $Si(CH_3)_3$].

^{13}C{^{1}H} NMR ($CDCl_3$, 126 MHz): δ 171.1, 158.9, 146.7, 145.4, 143.8, 141.0, 135.8, 134.6, 130.3, 130.1, 128.5(v weak), 113.4, 113.3, 80.2, 55.4, 32.3, 29.9, 21.8, 7.8, 4.5, and 0.0.

(both the proton and carbon NMR data show evidence for slow rotation about the biaryl bond in this compound.)

HRMS (ESI) *m/z*: $[M - OAc]^+$ Calcd for $C_{25}H_{37}OSi_2$ 409.2377; Found 409.2366 (100%).

IR (thin film): 3031, 2952, 2874, 1735, 1610, 1512, and 1234 cm^{-1}.

4-(*tert*-Butyl)-6,7-*bis*(4-methoxyphenyl)-3-methyl-1*H*-indene (12c)

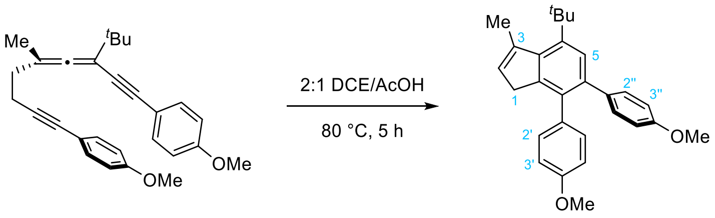

11c **12c**

Allenyne **11c** (10.0 mg, 0.025 mmol) was dissolved in a 2:1 mixture (vol ratio) of DCE/AcOH (1.5 mL) in a threaded culture tube. The tube was capped and heated at 80 ℃ for 5 h. After the reaction solution was concentrated, the residue was passed through a pipet column of silica gel (elution with 5:1 hexanes/EtOAc) and the filtrate was reconcentrated. The residue was purified by MPLC (16:1 hexanes/EtOAc) to provide **12c** (8.7 mg, 87%) as a clear oil.

Data for 12c

^{1}H NMR ($CDCl_3$, 500 MHz): δ 7.43 (s, 1H, H5), 7.06 (nfod, J_{app} = 8.8 Hz, 4H, H2'+H6'+H2''+H6''), 6.80 (nfod, J_{app} = 8.8 Hz, 2H, H3'+H5' or H3''+H5''), 6.72 (nfod, J_{app} = 8.8 Hz, 2H, H3'+H5' or H3''+H5''), 6.38 (tq, J = 2.4, 1.2 Hz, 1H, H2), 3.79 (s, 3H, 4'OCH_3 or 4''OCH_3), 3.76 (s, 3H, 4'OCH_3 or 4''OCH_3), 3.09 (qd, J = 2.1, 2.1 Hz, 2H, C1H_2), 2.58 (td, J = 2.1, 1.3 Hz, 3H, C3CH_3), and 1.56 [s, 9H, $C(CH_3)_3$].

^{13}C{^{1}H} NMR ($CDCl_3$, 126 MHz): δ 158.109, 157.9, 147.6, 144.2, 141.6, 140.5, 136.4, 135.02, 134.7, 134.2, 132.6, 131.3, 131.2, 126.7, 113.5, 113.2, 55.280, 55.269, 38.1, 35.1, 33.4, and 21.6.

HRMS (ESI) *m/z*: $[M + H]^+$ Calcd for $C_{28}H_{31}O_2$ 399.2319; Found 399.2290 (99%).

IR (thin film): 3036, 2999, 2954, 1606, 1509, and 1247 cm^{-1}.

7-(*tert*-Butyl)-4,5-*bis*(4-methoxyphenyl)-1-methylene-2,3-dihydro-1*H*-indene (12c')

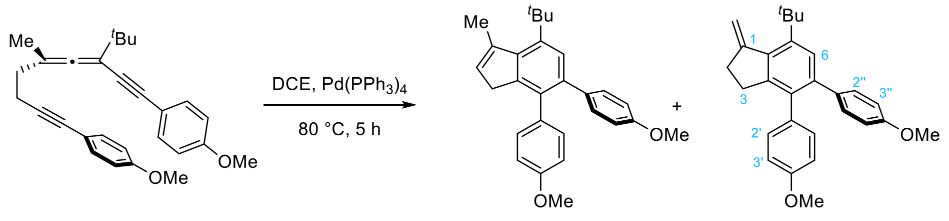

Allenyne **11c** (10.0 mg, 0.025 mmol) and) of $Pd(PPh_3)_4$ (3.0 mg, 0.0026 mmol, 0.1 equiv) was dissolved in of DCE (1.5 mL) in a threaded culture tube. The tube was capped and heated at 80 ℃ for 5 h. After the reaction solution was concentrated, the residue was passed through a pipet column of silica gel (elution with 5:1 hexanes/EtOAc) and the filtrate was reconcentrated. The residue was purified by MPLC (25:1 hexanes/EtOAc) to provide the mixture of **12c** and **12c'** (coeluted, 7.2 mg, 72% in total, 1:1 ratio determined by ^{1}H NMR analysis) as a clear oil.

Data for 12c' (extracted from the ca. 1:1 mixture of **12c** and **12c'**, the endo- and exo-cyclic alkenes)

^{1}H NMR ($CDCl_3$, 500 MHz): δ 7.39 (s, 1H, H6), 7.04 (nfod, J_{app} = 8.8 Hz, 2H, H2'+H6' or H2''+H6''), 6.99 (nfod, J_{app} = 8.8 Hz, 2H, H2'+H6'or H2''+H6''), 6.77 (nfod, J_{app} = 8.8 Hz, 2H, H3'+H5' or H3''+H5''), 6.72 (nfod, J_{app} = 8.8 Hz, 2H, H3'+H5' or H3''+H5''), 5.55 (t, *J* = 1.9 Hz, 1H, C1H_aH$_b$), 5.44 (t, *J* = 2.0 Hz, 1H, C1H$_a$$H_b$), 3.78 (s, 3H, 4'$OCH_3$ or 4''OCH_3), 3.76 (s, 3H, 4'OCH_3 or 4''OCH_3), 2.75–2.67 (m, 4H, C2H_2+C3H_2), and 1.56 [s, 9H, C(CH_3)$_3$].

^{13}C{^{1}H} NMR ($CDCl_3$, 126 MHz): δ 158.2, 158.113, 151.7, 149.3, 146.6, 137.1, 135.05, 134.4, 133.2, 132.3, 131.4, 131.1, 127.4, 113.4, 113.3, 111.2, 55.287, 55.263, 37.1, 35.5, 30.9, and 30.8.

HRMS (ESI) *m/z*: [M + H]$^+$ Calcd for $C_{28}H_{31}O_2$ 399.2319; Found 399.2290 (99%).

IR (thin film, of the mixture of **12c** and **12c'**): 3032, 2999, 2957, 1610, 1512, and 1246 cm^{-1}.

(±)-1-(*tert*-Butyl)-4-(4-chlorophenyl)-3-(4-methoxyphenyl)-9*H*-fluoren-9-yl Acetate (12d)

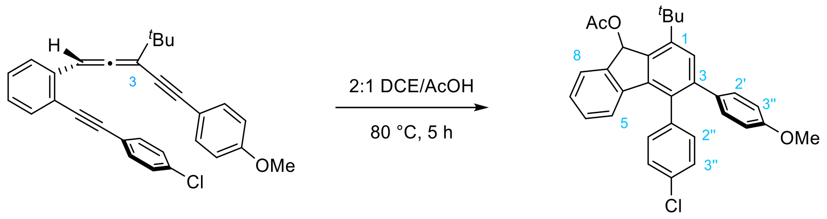

11d **12d**

Allenyne **11d** (10.0 mg, 0.023 mmol) was dissolved in a 2:1 mixture (vol ratio) of DCE/AcOH (1.5 mL) in a threaded culture tube. The tube was capped and heated at 80 ℃ for 5 h. After the reaction solution was concentrated, the residue was passed through a pipet column of silica gel (elution with 5:1 hexanes/EtOAc) and the filtrate was reconcentrated. The residue was purified by MPLC (16:1 hexanes/EtOAc) to provide **12d** (6.9 mg, 62%) as a yellow oil.

Data for 12d

^{1}H NMR ($CDCl_3$, 500 MHz): δ 7.63 (dd, J = 7.5, 1.1 Hz, 1H, H8), 7.41 (s, 1H, H2), 7.32 (dd, J = 8.1, 2.4 Hz, 1H, H3'' or H5''), 7.28 (dd, J = 8.1, 2.2 Hz, 1H, H3'' or H5''), 7.21 (dd, J = 8.1, 2.1 Hz, 1H, H6'' or H2''), 7.16 (s, 1H, H9), 7.14 (ddd, J = 7.5, 7.5, 1.2 Hz, 1H, H6 or H7), 7.04 (dd, J = 8.2, 2.3 Hz, 1H, H6'' or H2''), 6.99 (ddd, J = 7.5, 7.5, 1.3 Hz, 1H, H6 or H7), 6.99 (nfod, J_{app} = 8.8 Hz, 2H, H2'+H6'), 6.73 (nfod, J_{app} = 8.9 Hz, 2H, H3'+H5'), 6.27 (ddd, J = 7.8, 0.9, 0.9 Hz, 1H, H5),), 3.77 (s, 3H, OCH_3), 2.18 [s, 1H, C(O)CH_3], and 1.50 [s, 9H, $C(CH_3)_3$].

^{13}C{^{1}H} NMR ($CDCl_3$, 126 MHz): δ 171.7, 158.4, 148.4, 143.3, 143.2, 140.7, 140.5, 137.8, 137.0, 133.5, 133.3, 133.1, 132.1, 131.8, 131.0, 129.2, 129.0, 128.9, 128.8, 127.7, 126.0, 123.1, 113.3, 75.6, 55.3, 36.5, 31.8, and 21.9.

HRMS (ESI) *m/z*: [M - OAc]$^+$ Calcd for $C_{32}H_{27}{}^{35}ClO_3$ 437.1667; Found 437.1658 (100%).

IR (thin film): 3057, 2960, 1733, 1607, 1483, 1377, and 1224 cm^{-1}.

Half-life measurement: In a separate experiment, **11d** was dissolved in $CDCl_3$ and its decomposition/conversion was monitored at ambient temperature by ^{1}H NMR analysis. From periodic monitoring over time, it was estimated that the half-life for cyclization was ca. 8 h.

(±)-1-(*tert*-Butyl)-4-(4-chlorophenyl)-3-propyl-9*H*-fluoren-9-yl Acetate (12e)

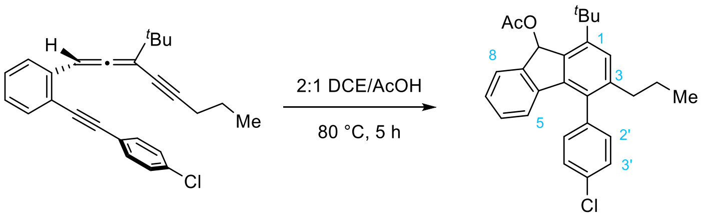

11e **12e**

Allenyne **11e** (10.0 mg, 0.027 mmol) was dissolved in a 2:1 mixture (vol ratio) of DCE/AcOH (1.5 mL) in a threaded culture tube. The tube was capped and heated at 80 ℃ for 5 h. After the reaction solution was concentrated, the residue was passed through a pipet column of silica gel (elution with 10:1 hexanes/EtOAc) and the filtrate was reconcentrated. The residue was purified by MPLC (30:1 hexanes/EtOAc) to provide **12e** (8.7 mg, 75%) as a yellow oil.

Data for 12e

^{1}H NMR ($CDCl_3$, 500 MHz): δ 7.59 (dd, *J* = 7.5, 1.1 Hz, 1H, H8), 7.49 (dd, *J* = 8.4, 2.1 Hz, 1H, H3' or H5'), 7.47 (dd, *J* = 8.4, 2.1 Hz, 1H, H3' or H5'), 7.31 (s, 1H, H2), 7.27 (dd, *J* = 8.5, 2.6 Hz, 1H, H2' or H6'), 7.18 (dd, *J* = 8.8, 2.5 Hz, 1H, H2' or H6'), 7.104 (ddd, *J* = 7.6, 7.6, 1.0 Hz, 1H, H6 or H7), 7.096 (s, 1H, H9), 6.98 (ddd, *J* = 7.7, 7.7, 1.1 Hz, 1H, H6 or H7), 6.09 (ddd, *J* = 7.9, 0.9, 0.9 Hz, 1H, H5), 2.34 (nfom, 2H, $CH_2CH_2CH_3$), 2.15 [s, 1H, C(O)CH_3], 1.48 [s, 9H, C(CH_3)$_3$], 1.46 (qd, *J* = 7.4, 7.4 Hz, 2H, $CH_2CH_2CH_3$), and 0.83 (t, *J* = 7.3 Hz, 2H,$CH_2CH_2CH_3$).

^{13}C{^{1}H} NMR ($CDCl_3$, 126 MHz): δ 171.7, 148.3, 143.3, 142.8, 140.7, 140.5, 138.1, 135.5, 133.8, 133.6, 131.2, 131.1, 129.32, 129.26, 129.1, 127.8, 127.4, 125.9, 122.8, 75.7, 36.4, 35.4, 31.8, 24.7, 21.9, and 14.2.

HRMS (ESI) *m/z*: [M - OAc]$^+$ Calcd for $C_{26}H_{26}{}^{35}Cl$ 373.1718; Found 373.1722 (100%).

IR (thin film): 3054, 2960, 2870, 1735, 1606, 1484, 1367, and 1232 cm^{-1}.

2-(4-Methoxyphenyl)-7,7-dimethyl-4-(trimethylsilyl)-7*H*-cyclopenta[b]pyridine (12f)

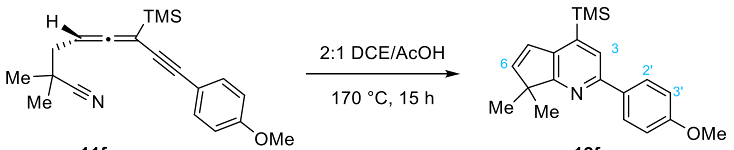

Allenyne **11f** (10.0 mg, 0.031 mmol) was dissolved in a 2:1 mixture (vol ratio) of DCE/AcOH (1.5 mL) in a threaded culture tube. The tube was capped and heated at 170 ℃ for 15 h. After the reaction solution was concentrated, the residue was passed through a pipet column of silica gel (elution with 3:1 hexanes/EtOAc) and the filtrate was reconcentrated. The residue was purified by MPLC (5:1 hexanes/EtOAc) to provide **12f** (3.5 mg, 35%) as a clear oil.

Data for 12f

^{1}H NMR ($CDCl_3$, 500 MHz): δ 7.99 (nfod, J_{app} = 8.8 Hz, 2H, H2'+H6'), 7.55 (s, 1H, H3), 6.99 (nfod, J_{app} = 8.8 Hz, 2H, H3'+H5'), 6.83 (d, J = 5.8 Hz, 1H, H5), 6.54 (d, J = 5.7 Hz, 1H, H6), 3.86 (s, 3H, 4'OCH_3), 1.39 [s, 6H, C7$(Me)_2$], and 0.38 [s, 9H, Si$(CH_3)_3$].

^{13}C{^{1}H} NMR ($CDCl_3$, 126 MHz): δ 171.3, 160.0, 151.7, 147.5, 141.5, 138.1, 133.6, 128.3, 127.1, 122.3, 114.2, 55.5, 49.0, 23.0, and -0.7.

HRMS (ESI) *m/z*: $[M + H]^+$ Calcd for $C_{20}H_{26}NO_2Si$ 324.1778; Found 324.1764 (100%).

IR (thin film): 3089, 2960, 1608, 1533, and 1249 cm^{-1}.

(±)-4-(*tert*-Butyl)-2-(4-methoxyphenyl)-5*H*-indeno[1,2-b]pyridin-5-yl Acetate (12g)

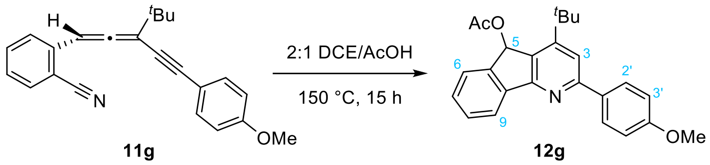

Allenyne **11g** (10.0 mg, 0.030 mmol) was dissolved in a 2:1 mixture (vol ratio) of DCE/AcOH (1.5 mL) in a threaded culture tube. The tube was capped and heated at 150 ℃ for 15 h. After the reaction solution was concentrated, the residue was passed through a pipet column of silica gel (elution with 2:1 hexanes/EtOAc) and the filtrate was reconcentrated. The residue was purified by MPLC (4:1 hexanes/EtOAc) to provide **12g** (7.4 mg, 63%) as a clear oil.

Data for 12g

^{1}H NMR ($CDCl_3$, 500 MHz): δ 8.06 (nfod, J_{app} = 8.8 Hz, 2H, H2'+H6'), 8.06 (overlapping m, 1H, H9), 7.65 (ddd, J = 7.6, 1.2, 0.6, 0.6 Hz, 1H, H6), 7.60 (s, 1H, H3), 7.47 (ddd, J = 7.5, 7.5, 1.2 Hz, 1H, H7), 7.37 (ddd, J = 7.5, 7.5, 1.2 Hz, 1H, H8), 7.12 (d, J = 0.6 Hz, 1H, H5), 7.03 (nfod, J_{app} = 8.8 Hz, 2H, H3'+H5'), 3.89 (s, 3H, 4'OCH_3), 2.18 (s, 3H, $OCOCH_3$), and 1.48 [s, 9H, $C(CH_3)_3$].

^{13}C{^{1}H} NMR ($CDCl_3$, 126 MHz): δ 171.6, 161.7, 160.7, 158.6, 158.2, 143.4, 140.7, 134.2, 132.5, 129.9, 129.8, 128.6, 126.0, 121.1, 116.8, 114.2, 74.5, 55.6, 36.5, 30.8, and 21.7.

HRMS (ESI) *m/z*: $[M + H]^+$ Calcd for $C_{25}H_{26}NO_3$ 388.1907; Found 388.1890 (100%).

IR (thin film): 3030, 2999, 2937, 1736, 1608, 1514, 1248, and 1230 cm^{-1}.

Half-life measurement: In another experiment, **11g** was heated under the same conditions as the procedure described above, but at 145 °C. After 90 minutes, the reaction was cooled and concentrated. The ^{1}H NMR spectrum of the crude residue showed an ca. 1:3 ratio of **12g**:**11g**, indicating an approximate half-life for the cyclization of $t_{1/2}$ = 3 h.

5-(*tert*-Butyl)-7-(4-chlorophenyl)-8-((4-methoxyphenyl)ethynyl)-1,2-dihydronaphthalene (12h) and (±)-8-(*tert*-Butyl)-8-((4-chlorophenyl)ethynyl)-7-((4-methoxyphenyl)ethynyl)bicyclo[4.2.0]octa-1,6-diene (12h')

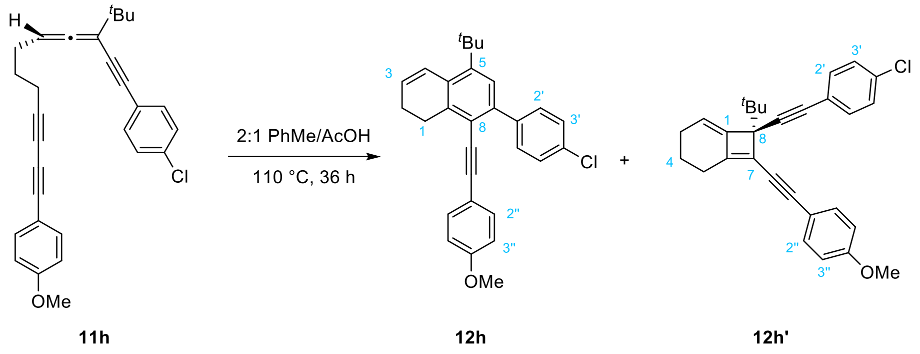

Allenyne **11h** (20.0 mg, 0.047 mmol) was dissolved in a 2:1 mixture (vol ratio) of PhMe/AcOH (3.0 mL) in a threaded culture tube. The tube was capped and ` heated at 110 ℃ for 36 h. After the reaction solution was concentrated, the residue was passed through a pipet column of silica gel (elution with 5:1 hexanes/EtOAc) and the filtrate was reconcentrated. The residue was purified by MPLC (35:1 hexanes/EtOAc) to provide, in order of elution, **12h** (7.0 mg, 35%) and **12h'** (10.9 mg, 55%), each as a clear oil.

Data for 12h

^{1}H NMR ($CDCl_3$, 500 MHz): δ 7.59 (nfod, J_{app} = 8.8 Hz, 2H, H2''+H6''), 7.40 (nfod, J_{app} = 8.4 Hz, 2H, H3'+H5'), 7.25 (nfod, J_{app} = 8.2 Hz, 2H, H2'+H6'), 7.25 (s, 1H, H6), 7.11 (dt, J = 10.1, 1.8 Hz, 1H, H4), 6.83 (nfod, J_{app} = 8.8 Hz, 2H, H3''+H5''), 6.18 (dt, J = 9.8, 4.5 Hz, 1H, H3), 3.81 (s, 3H, 4'OCH_3), 3.16 (t, J = 7.9 Hz, 2H, C1H_2), 2.30 (tdd, J = 8.2, 4.6, 1.8 Hz, 2H, C2H_2), and 1.46 [s, 9H, $C(CH_3)_3$].

^{13}C{^{1}H} NMR ($CDCl_3$, 126 MHz): δ 159.6, 145.4, 140.6, 140.4, 139.9, 133.2, 132.7, 131.7, 131.0, 128.2, 128.0, 126.9, 125.4, 118.8, 115.9, 114.1, 96.8, 86.5, 55.5, 35.7, 31.7, 27.3, and 22.2.

HRMS (ESI) *m/z*: [M + H]$^+$ Calcd for $C_{29}H_{28}{}^{35}ClO$ 427.1823; Found 427.1806 (1%).

IR (thin film): 3043, 2957, 2148, 1605, 1510, and 1249 cm^{-1}.

Data for 12h'

^{1}H NMR ($CDCl_3$, 500 MHz): δ 7.39 (nfod, J_{app} = 8.8 Hz, 2H, H2''+H6''), 7.37 (nfod, J_{app} = 8.4 Hz, 2H, H2'+H6'), 7.23 (nfod, J_{app} = 8.6 Hz, 2H, H3'+H5'), 6.84 (nfod, J_{app} = 8.8 Hz, 2H, H3''+H5''), 5.47 (tdd, J = 4.2, 0.8, 0.8 Hz, 1H, H2), 3.81 (s, 3H, 4'OCH_3), 2.42 (ddd, J = 17.1, 6.1, 6.1, 0.8 Hz, 1H, C5H_aH_b), 2.40 (ddd, J = 17.1, 7.2, 5.3 Hz, 1H, C5H_aH_b]), 2.21 (ddd, J = 5.9, 5.9, 4.1 Hz, 2H, C3H_aH_b), 1.83 (ddddd, J = 13.1, 6.8, 5.5, 5.5, 5.5 Hz, 1H, C4H_aH_b), 1.75 (ddddd, J = 13.6, 7.5, 6.0, 6.0, 6.0 Hz, 1H, C4H_aH_b), and 1.17 [s, 9H, $C(CH_3)_3$].

^{13}C{^{1}H} NMR ($CDCl_3$, 126 MHz): δ 159.8 (C4''), 152.5 (C1), 143.7 (C6), 133.5 (C4'), 133.2 (C2'+C6' or C2''+C6''), 133.0 (C2'+C6' or C2''+C6''), 128.5 (C3'+C5'), 124.3 (C7), 122.8 (C1'), 115.7 (C1''), 114.1 (C3''+C5''), 112.3 (C2), 99.2, 92.1, 82.5, 82.3, 63.5 (C8), 55.5 (OCH_3), 36.2 ($C(CH_3)_3$), 26.7 ($C(CH_3)_3$), 24.7 (C3), 23.0 (C5), and 22.9 (C4). (carbon assignments based on HSQC and HMBC analyses)

HRMS (ESI) *m/z*: [M + H]$^+$ Calcd for $C_{29}H_{28}{}^{35}ClO$ 427.1823; Found 427.1806 (6%).
IR (thin film): 3037, 3001, 2954, 2144, 1602, 1509, and 1250 cm^{-1}.

(±)-12-(4-Methoxyphenyl)-6-(trimethylsilyl)dodeca-6,7-dien-4,11-diyn-1-ol (17)

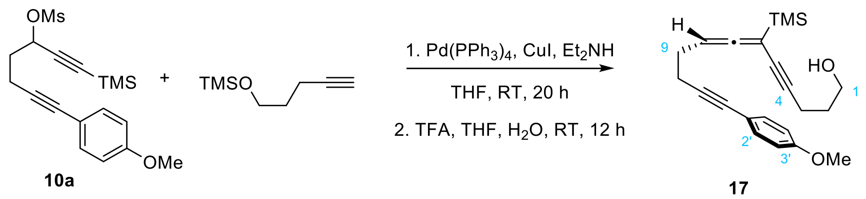

Compound **17** was prepared following general procedure B followed by further desilylation. The mesylate 10a (1.82 g, 5.0 mmol) was used and the crude product from the first reaction was dissolved in 4:1 THF/H2O (vol ratio, 20 mL) and TFA (2 mL) was added. This reaction solution was stirred for 12 h at room temperature and then quenched by the addition of satd aq Na2CO3. The resulting mixture was extracted with EtOAc, dried (MgSO4), concentrated, and purified by flash column chromatography (1:1 hexanes/EtOAc) to give **17** (1.36 g, 77%) as an orange oil. (ca. $t_{1/2}$ = 1 h @80 ℃, see p S29)

Data for 17

^{1}H NMR ($CDCl_3$, 500 MHz): δ 7.33 (nfod, J_{app} = 8.8 Hz, 2H, H2'+H6'), 6.80 (nfod, J_{app} = 8.8 Hz, 2H, H3'+H5'), 5.19 (ddt, J = 6.6, 6.6 1.1 Hz, 1H, H8), 3.81 (s, 3H, 4'OCH_3), 3.77–3.74 (m, 2H, C1H_2), 2.54–2.45 (m, 2H, C10H_2), 2.43 (td, J = 6.9, 1.2 Hz, 2H, C3H_2), 2.33–2.28 (m, 2H, C9H_2), 1.77 (tt, J = 6.9, 6.9 Hz, 1H, C2H_2), 1.46 (bs, 1H, OH) and 0.16 [s, 9H, $Si(CH_3)_3$].

^{13}C{^{1}H} NMR ($CDCl_3$, 126 MHz): δ 212.4, 159.2, 133.1, 116.2, 113.9, 91.1, 87.9, 85.6, 84.8, 81.0, 75.3, 62.1, 55.4, 31.8, 27.7, 19.8, 16.5, -1.7.

HRMS (ESI) *m/z*: $[M + H]^+$ Calcd for $C_{22}H_{29}O_2Si$ 353.1931; Found 351.1934 (100%).

IR (thin film): 3600–3200, 3029, 2953, 2198, 1929, 1606, 1509, and 1245 cm^{-1}.

3-(7-(4-Methoxyphenyl)-4-(trimethylsilyl)-1*H*-inden-6-yl)propan-1-ol (18)

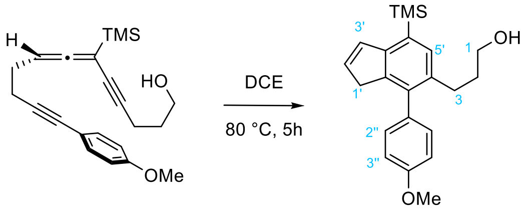

17 **18**

Allenyne **17** (10.0 mg, 0.028 mmol) was dissolved in DCE (1.5 mL) in a threaded culture tube. The tube was capped and heated at 80 ℃ for 5 h. After the reaction solution was concentrated, the residue was passed through a pipet column of silica gel (elution with 1:1 hexanes/EtOAc) and the filtrate was reconcentrated. The residue was purified by MPLC (2:1 hexanes/EtOAc) to provide **18** (5.5 mg, 55%) as a clear oil.

Data for 18

^{1}H NMR ($CDCl_3$, 500 MHz): δ 7.43 (s, 1H, H5'), 7.20 (nfod, J_{app} = 8.8 Hz, 2H, H2''+H6''), 7.08 (dt, J = 5.9, 2.0 Hz, 1H, H3'), 6.98 (nfod, J_{app} = 8.8 Hz, 2H, H3''+H5''), 6.53 (dt, J = 5.7, 2.1 Hz, 1H, H2'), 3.87 (s, 3H, 4''OCH_3), 3.51 (t, J = 6.6 Hz, 2H, C1H_2), 3.08 (dd, J = 1.8, 1.8 Hz, 2H, C1'H_2), 2.62–2.58 (nfom, 2H, C3H_2), 1.61 (tt, J = 7.8, 6.3 Hz, 2H, C2H_2), 1.01 (bs, 1H, OH), and 0.38 [s, 9H, $Si(CH_3)_3$].

^{13}C{^{1}H} NMR ($CDCl_3$, 126 MHz): δ 158.7, 147.4, 143.2, 138.3, 135.5, 134.5, 133.3, 132.8, 132.3, 131.4, 130.2, 114.0, 62.7, 55.4, 38.9, 34.9, 29.3, and -0.0.

HRMS (ESI) *m/z*: $[M + H]^+$ Calcd for $C_{22}H_{29}O_2Si$ 353.1931; Found 353.1934 (100%).

IR (thin film): 3600–3200, 3047, 2952, 1610, 1513, 1246, and 1035 cm^{-1}.

Half-life measurement: In another experiment, **17** was heated under the same conditions as the procedure described above. After 60 minutes, the reaction was cooled and concentrated. The ^{1}H NMR spectrum of the crude residue showed an ca. 1:1 ratio of **18**:**17**, indicating an approximate half-life for the cyclization of $t_{1/2}$ = 1 h.

3-(4-(4-Methoxyphenyl)-7-(trimethylsilyl)-2,3-dihydro-1*H*-inden-5-yl)propan-1-ol (18-H2)

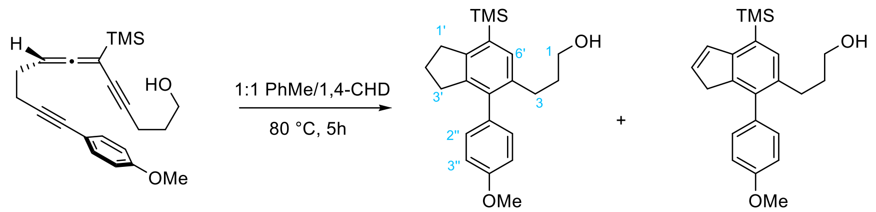

17 **18-H2** **18**

Allenyne **17** (10.0 mg, 0.028 mmol) was dissolved in a mixture of 1:1 (vol ratio) of PhMe/1,4-cyclohexadiene (1.5 mL) in a threaded culture tube. The tube was capped and heated at 80 ℃ for 5 h. After the reaction solution was concentrated, the residue was passed through a pipet column of silica gel (elution with 1:1 hexanes/EtOAc) and the filtrate was reconcentrated. The residue was purified by MPLC (3:1 hexanes/EtOAc) to provide, in order of elution, **18-H2** (2.1 mg, 21%) and **18** (2.2 mg, 22%), each as a clear oil.

Data for 18-H2

^{1}H NMR ($CDCl_3$, 500 MHz): δ 7.23 (s, 1H, H6'), 7.14 (nfod, J_{app} = 8.8 Hz, 2H, H2''+H6''), 6.94 (nfod, J_{app} = 8.8 Hz, 2H, H3''+H5''), 3.85 (s, 3H, 4''OCH_3), 3.50 (t, J = 6.4 Hz, 2H, C1H_2), 3.00 (t, J = 7.4 Hz, 2H, C1'H_2 or C3'H_2), 2.58 (t, J = 7.5 Hz, 2H, C1'H_2 or C3'H_2), 2.55–2.52 (nfom, 2H, C3H_2), 1.98 (tt, J = 7.5, 7.5 Hz, 2H, C2H_2), 1.68 (tt, J = 7.8, 6.4 Hz, 2H, C2H_2), 1.53 (bs, 1H, OH), and 0.32 [s, 9H, $Si(CH_3)_3$].

^{13}C{^{1}H} NMR ($CDCl_3$, 126 MHz): δ 158.5, 147.2, 143.0, 138.9, 136.9, 134.1, 133.1, 132.7, 130.2, 113.8, 62.8, 55.4, 34.8, 34.3, 32.4, 29.3, 25.4, and -0.4.

HRMS (ESI) *m/z*: $[M + H]^+$ Calcd for $C_{22}H_{31}O_2Si$ 355.2088; Found 355.2072 (34%).

IR (thin film): 3600–3200, 3043, 2952, 1610, 1514, and 1245 cm^{-1}.

3-(4-(4-Methoxyphenyl)-7-(trimethylsilyl)-2,3-dihydro-1*H*-inden-5-yl)propanal (19)

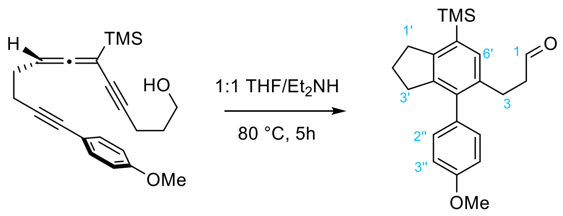

17 **19**

Allenyne **17** (10.0 mg, 0.028 mmol) was dissolved in a mixture of 1:1 (vol ratio) of THF/Et_2NH (1.5 mL) in a threaded culture tube. The tube was capped and heated at 80 ℃ for 5 h. After the reaction solution was concentrated, the residue was passed through a pipet column of silica gel (elution with 3:1 hexanes/EtOAc) and the filtrate was reconcentrated. The residue was purified by MPLC (10:1 hexanes/EtOAc) to provide **19** (3.5 mg, 35%) as a clear oil.

Data for 19

^{1}H NMR ($CDCl_3$, 500 MHz): δ 9.62 (t, *J* = 1.7, 1H, H1), 7.19 (s, 1H, H6'), 7.13 (nfod, J_{app} = 8.8 Hz, 2H, H2''+H6''), 6.94 (nfod, J_{app} = 8.8 Hz, 2H, H3''+H5''), 3.85 (s, 3H, 4''OCH_3), 3.00 (t, *J* = 7.4 Hz, 2H, C1'H_2 or C3'H_2), 2.81 (t, *J* = 7.5 Hz, 2H, C1'H_2 or C3'H_2), 2.58 (t, *J* = 7.8 Hz, 2H, C3H_2), 2.50 (td, *J* = 7.9, 1.7 Hz, 2H, C2H_2), 1.98 (tt, *J* = 7.5, 7.5 Hz, 2H, C2'H_2), and 0.32 [s, 9H, Si$(CH_3)_3$].

^{13}C{^{1}H} NMR ($CDCl_3$, 126 MHz): δ 202.2, 158.6, 147.8, 143.2, 138.9, 135.4, 134.4, 133.0, 132.3, 130.0, 114.0, 55.4, 45.6, 34.3, 32.4, 25.9, 25.4, and -0.5.

HRMS (ESI) *m/z*: $[M + H]^+$ Calcd for $C_{22}H_{29}O_2Si$ 353.1931; Found 353.1934 (100%).

IR (thin film): 3030, 2953, 2839, 2744, 1724, 1610, 1514, and 1246 cm^{-1}.

(±)-12-(4-Methoxyphenyl)-6-(trimethylsilyl)dodeca-6,7-dien-4,11-diynal (23)

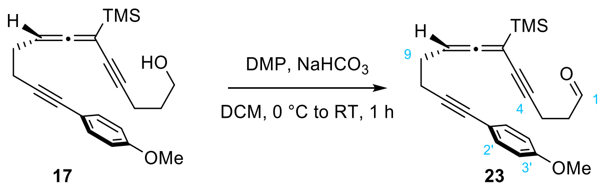

Allenyne **17** (352 mg, 1.0 mmol) was dissolved in DCM (10 mL) at 0 ℃ in a round-bottom flask. DMP (464 mg, 1.1 mmol) and solid $NaHCO_3$ (168 mg, 2 mmol) were added. After being stirred for 1 h, the reaction mixture was quenched by addition of satd aq Na_2SO_3 solution, extracted with DCM, dried ($MgSO_4$), concentrated, and purified by flash column chromatography (5:1 hexanes/EtOAc) to provide **23** (270 mg, 77%) as a yellow oil.

Data for 23

^{1}H NMR ($CDCl_3$, 500 MHz): δ 9.79 (t, J = 1.2, 1H, H1), 7.33 (nfod, J_{app} = 8.8 Hz, 2H, H2'+H6'), 6.80 (nfod, J_{app} = 8.8 Hz, 2H, H3'+H5'), 5.19 (tt, J = 6.6, 1.1 Hz, 1H, H8), 3.79 (s, 3H, 4'OCH_3), 2.63 (m, 4H, C2H_2+C3H_2), 2.49 (ddd, J = 16.8, 7.9, 6.9 Hz, 1H, C10$H_a$$H_b$), 2.47 (ddd, J =16.7, 7.0, 7.0 Hz, 1H, C10$H_a$$H_b$), 2.33 (ddd, J =15.7, 6.9, 6.9 Hz, 1H, C9$H_a$$H_b$), 2.30 (ddd, J =15.5, 7.7, 6.7 Hz, 1H, C9$H_a$$H_b$), and 0.15 [s, 9H, Si($CH_3$)$_3$].

^{13}C{^{1}H} NMR ($CDCl_3$, 126 MHz): δ 212.5, 200.9, 159.2, 133.1, 116.2, 114.0, 89.5, 87.8, 85.7, 84.6, 81.1, 75.9, 55.4, 43.0, 27.6, 19.8, 13.2, and -1.7.

HRMS (ESI) *m/z*: $[M + H]^+$ Calcd for $C_{22}H_{27}O_2Si$ 355.1775; Found 355.1778 (100%).

IR (thin film): 3029, 2956, 2751, 1716, 1606, 1511, and 1248 cm^{-1}.

3-(7-(4-Methoxyphenyl)-4-(trimethylsilyl)-1*H*-inden-6-yl)propanal (24) and 8-(4-Methoxyphenyl)-1,7-dihydro-*s*-indacene (25)

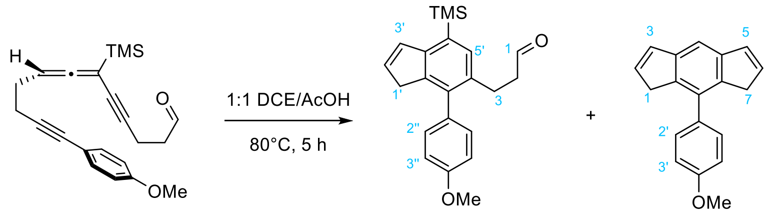

23 **24** **25**

Allenyne **23** (10.0 mg, 0.028 mmol) was dissolved in a mixture of 1:1 (vol ratio) of DCE/AcOH (1.5 mL) in a threaded culture tube. The tube was capped and heated at 80 ℃ for 5 h. After the reaction solution was concentrated, the residue was passed through a pipet column of silica gel (elution with 3:1 hexanes/EtOAc) and the filtrate was reconcentrated. The residue was purified by MPLC (10:1 hexanes/EtOAc), to provide, in order of elution, **25** (3.4 mg, 45%) and **24** (1.8 mg, 18%), each as a clear oil.

Data for 24

^{1}H NMR ($CDCl_3$, 500 MHz): δ 9.63 (t, *J* = 1.7, 1H, H1), 7.31 (s, 1H, H5'), 7.19 (nfod, J_{app} = 8.8 Hz, 2H, H2''+H6''), 7.08 (dt, *J* = 5.7, 1.9 Hz, 1H, H3'), 6.98 (nfod, J_{app} = 8.8 Hz, 2H, H3''+H5''), 6.56 (dt, *J* = 5.7, 2.0 Hz, 1H, H2'), 3.87 (s, 3H, OCH_3), 3.08 (dd, *J* = 1.9, 1.9 Hz, 2H, C1'H_2), 2.87 (t, *J* = 7.6 Hz, 2H, C3H_2), 2.53 (td, *J* = 7.3, 1.7 Hz, 2H, C2H_2), and 0.38 [s, 9H, $Si(CH_3)_3$].

^{13}C{^{1}H} NMR ($CDCl_3$, 126 MHz): δ 202.2, 158.8, 147.9, 143.4, 138.3, 134.9, 133.9, 133.2, 132.7, 131.9, 131.7, 130.0, 114.1, 55.4, 45.7, 38.8, 25.9, and -0.1.

HRMS (ESI) *m/z*: $[M + H]^+$ Calcd for $C_{22}H_{27}O_2Si$ 351.1775; Found 351.1778 (20%).

IR (thin film): 3072, 2955, 2878, 2751, 1723, 1608, 1512, and 1248 cm^{-1}.

Data for 25

^{1}H NMR ($CDCl_3$, 500 MHz): δ 7.43 (s, 1H, H4), 7.39 (nfod, J_{app} = 8.8 Hz, 2H, H2'+H6'), 7.00 (nfod, J_{app} = 8.8 Hz, 2H, H3'+H5'), 6.95 (dt, *J* = 5.7, 1.9 Hz, 2H, H3+H5), 6.52 (dt, *J* = 5.7, 2.0 Hz, 2H, H2+H6), 3.88 (s, 3H, OCH_3), and 3.30 [dd *J* = 1.7, 1.7 Hz, 4H, CH_2].

^{13}C{^{1}H} NMR ($CDCl_3$, 126 MHz): δ 158.8, 144.2, 139.6, 134.1, 132.4, 129.9, 114.0, 112.9, 55.4, and 38.5. (the two biaryl-linked carbons were not identifiable in either the 1D ^{13}C nor HMBC spectra)

HRMS (ESI) *m/z*: $[M + H]^+$ Calcd for $C_{19}H_{17}O$ 261.1274; Found 261.1278 (47%).

IR (thin film): 3057, 2954, 1607, 1513, and 1249 cm^{-1}.

(±)-2-(4-Methoxyphenyl)-4,4-dimethyl-5,5a-dihydro-4*H*-benzo[2,3]pentaleno[1,6-*bc*]pyridine (29), (±)-2-(4-Methoxyphenyl)-5,5-dimethyl-5,5a-dihydro-4*H*-benzo[2,3]pentaleno[1,6-*bc*]pyridine (30), and 2-(4-Methoxyphenyl)-4-(2-methylallyl)-5*H*-indeno[1,2-b]pyridine (31)

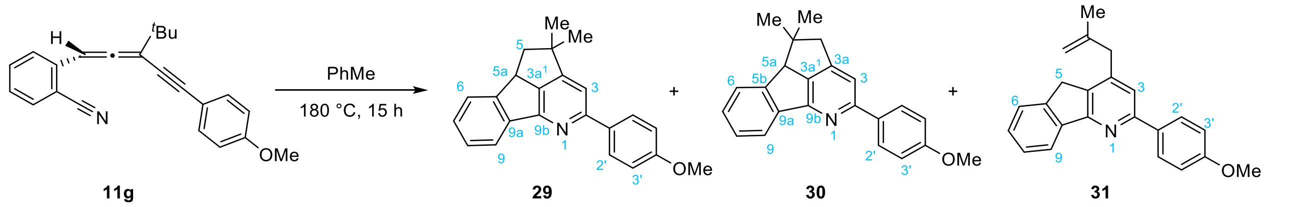

Allenyne **11g** (200 mg, 0.61 mmol) was dissolved in PhMe (20 mL) in a threaded culture tube. The tube was capped and heated at 180 ℃ for 15 h. After the reaction solution was concentrated, the residue was passed through a short column of silica gel (elution with 3:1 hexanes/EtOAc) and the filtrate was reconcentrated. The residue was purified by MPLC (12:1 hexanes/EtOAc), in order of elution, to provide **31** (54 mg, 27%), **29** (18 mg, 9%), and **30** (8.0 mg, 4%) as a yellow oil, a white crystalline solid, and a clear oil, respectively.

Data for 29

^{1}H NMR ($CDCl_3$, 500 MHz): δ 8.04 (nfod, J_{app} = 8.9 Hz, H2'+H6', 2H), 8.03 (overlapped, H9, 1H), 7.51 (ddd, *J* = 7.5, 1.1, 1.1 Hz, H6), 7.42 (dd, *J* = 7.5, 7.5 Hz, 1H, H8), 7.31 (ddd, *J* = 7.5, 7.5, 1.1 Hz, H7), 7.10 (s, 1H, H3), 7.01 (nfod, J_{app} = 8.8 Hz, 2H, H3'+H5'), 4.46 (dd, *J* = 9.9, 7.2 Hz, 1H, H5a), 3.88 (s, 3H, OCH_3), 2.60 (dd, *J* = 11.6, 7.4 Hz, 1H, C5H_aH_b), 1.70 (dd, *J* = 11.4, 9.9 Hz, 1H, C5H_aH_b), 1.67 (s, 3H, C4CH_3CH_3'), and 1.30 (s, 3H, C4CH_3CH_3').

^{13}C{^{1}H} NMR ($CDCl_3$, 126 MHz): δ 160.8 (C3a), 160.3 (C4'), 159.9 (C2), 158.6 (C9b), 152.6 (C3a^1), 152.0 (C5b), 144.2 (C9a), 133.6 (C1'), 128.9 (C2'+C6'), 127.8 (C7), 127.6 (C8), 126.1 (C6), 122.0 (C9), 114.1 (C3'+C5'), 110.5 (C3), 55.5 (OCH_3), 52.0 (C4), 50.9 (C5), 45.2 (C5a), 27.0 (Me_a), and 26.6 (Me_b). [Assignments from analysis of the HSQC and HMBC data]

HRMS (ESI) *m/z*: $[M + H]^+$ Calcd for $C_{23}H_{22}NO$ 328.1696; Found 328.1681 (100%).

IR (thin film): 3080, 2968, 1609, 1574, 1249, and 1172 cm^{-1}.

mp: 59.1–63.7 ℃

Data for 30

^{1}H NMR ($CDCl_3$, 500 MHz): δ 8.05 (nfod, J_{app} = 8.9 Hz, H2'+H6', 2H), 8.04 (overlapped, H9, 1H), 7.51 (ddd, *J* = 7.5, 1.1, 1.1 Hz, H6), 7.44 (dd, *J* = 7.5, 7.5 Hz, 1H, H7), 7.33 (ddd, *J* = 7.5, 7.5, 1.1 Hz, H8), 7.22 (s, 1H, H3), 7.01 (nfod, J_{app} = 8.8 Hz, 2H, H3'+H5'), 4.08 (s, 1H, H5a), 3.88 (s, 3H, OCH_3), 3.60 (d, *J* = 15.0 Hz, 1H, C4H_aH_b), 2.63 (d, *J* = 15.0, 1H, C4H_aH_b), 1.65 (s, 3H, C5CH_3CH_3'), and 0.30 (s, 3H, C5CH_3CH_3').

^{13}C{^{1}H} NMR ($CDCl_3$, 126 MHz): δ 160.4 (C4'), 159.5 (C9b), 158.9 (C3a), 152.2 (C3a^1), 149.4 (C5b), 144.7 (C9a), 133.4 (C1'), 128.9 (C2'+C6'), 127.8 (C7 or C8), 127.7 (C7 or C8), 126.5 (C6), 122.1 (C9), 114.2 (C3'+C5'), 113.7 (C3), 57.4 (C5a), 55.5 (OMe), 52.6 (C5), 52.2 (C4), 29.1 (Me_a), and 21.8 (Me_b). [Assignments from analysis of the HSQC and HMBC data, C2 was not identifiable]

HRMS (ESI) *m/z*: $[M + H]^+$ Calcd for $C_{23}H_{22}NO$ 328.1696; Found 328.1679 (100%).

IR (thin film): 3054, 2954, 1606, 1514, and 1250 cm^{-1}.

Data for 31

^{1}H NMR (C_6D_6, 500 MHz): δ 8.50 (ddd, *J* = 7.5, 1.1, 1.1 Hz, H9), 8.35 (nfod, J_{app} = 8.8 Hz, 2H, H2'+H6'), 7.39 (s, 1H, H3), 7.33 (ddd, *J* = 7.5, 7.5, 1.1 Hz, H7 or H8), 7.31 (ddd, *J* = 7.6, 1.1, 1.1 Hz, 1H, H6), 7.25 (ddd, *J* = 7.5, 7.5, 1.2 Hz, H7 or H8), 6.98 (nfod, J_{app} = 8.8 Hz, 2H, H3'+H5'), 4.79 (qd, *J* = 1.2, 0.8 Hz, 1H, C3''H_aH_b), 4.69 (qd, *J* = 1.0, 1.0 Hz, 1H, C3''H_aH_b), 3.38 (s, 2H, C5H_2), 3.33 (s, 3H, OCH_3), 3.11 (s, 2H, C1''H_2), and 1.52 (dd, *J* = 1.5, 0.9 Hz, C2''CH_3).

^{13}C{^{1}H} NMR (C_6D_6, 126 MHz): δ 161.0, 160.9, 157.0, 144.6, 144.3, 142.7, 142.5, 134.3, 133.2, 128.9, 128.6, 127.6, 125.3, 121.8, 118.3, 114.4, 113.1, 54.9, 42.0, 33.1, and 22.3.

HRMS (ESI) *m/z*: $[M + H]^+$ Calcd for $C_{23}H_{22}NO$ 328.1696; Found 328.1677 (100%).
IR (thin film): 3071, 2967, 2993, 1606, 1573, 1362, 1240, and 1172 cm^{-1}.

(±)-(3-(*tert*-Butyl)-5-(2-((4-chlorophenyl)ethynyl)phenyl)penta-3,4-dien-1-yn-1-yl)triethylsilane (36)

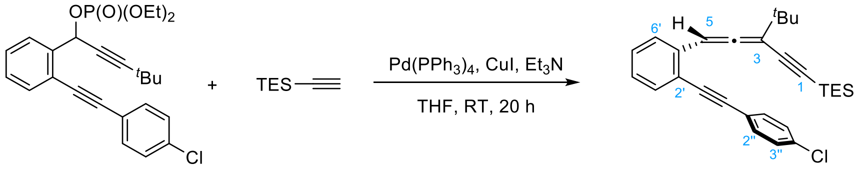

S2 36

Data for 36

Compound **36** was prepared following general procedure B and purified by MPLC (hexanes). Use of the phosphate **S2** (459 mg, 1.0 mmol) resulted in the isolation of **11e** (249 mg, 56%) as an orange crystalline solid.

^{1}H NMR ($CDCl_3$, 500 MHz): δ 7.49 (overlapped peaks, 3H, H3'+H2''+H6''), 7.44 (dd, *J* = 7.9, 1.3 Hz, 1H, H6'), 7.34 (nfod, J_{app} = 8.5 Hz, 2H, H3''+H5''), 7.30 (ddd, *J* = 7.5, 7.5 1.5 Hz, 1H, H4' or H5'), 7.20 (ddd, *J* = 7.6, 7.6 1.4 Hz, 1H, H4' or H5'), 7.02 (s, 1H, H5), 1.22 [s, 9H, $C(CH_3)_3$], 1.01 [t, *J* = 8.0 Hz, 9H, $Si(CH_2CH_3)_3$], and 0.63 [q, *J* = 8.0 Hz, 6H, $Si(CH_2CH_3)_3$].

^{13}C{^{1}H} NMR ($CDCl_3$, 126 MHz): δ 211.1, 135.6, 134.6, 133.0, 132.6, 128.93, 128.88, 127.1, 126.9, 121.8, 121.2, 105.5, 99.3, 96.6, 95.6, 93.3, 88.6, 35.7, 29.3, 7.7, and 4.6.

HRMS (ESI) *m/z*: $[M + H]^+$ Calcd for $C_{29}H_{34}{}^{35}ClSi$ 445.2113 ; Found 445.2121 (5%).

IR (thin film): 3080, 2956, 2874, 2137, 1931, 1601, 1479, 1209, and 1098 cm^{-1}.

mp: 99.4–104.8 ℃

(2-(1-(*tert*-Butyl)-4-(4-chlorophenyl)-9*H*-fluoren-3-yl)ethyl)diethyl(methoxy)silane (37) and (2-(1-(*tert*-Butyl)-4-(4-chlorophenyl)-9*H*-fluoren-3-yl)diethyl(methoxy)silane (38)

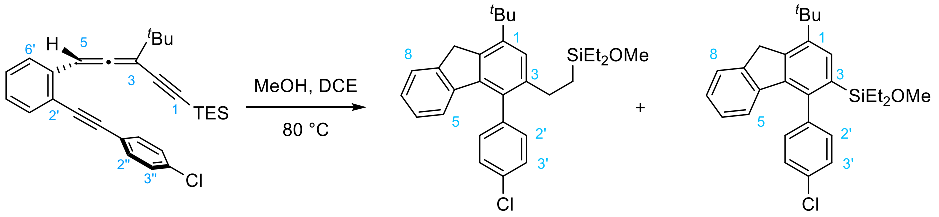

36 **37** **38**

Allenyne **36** (20 mg, 0.050 mmol) was dissolved in MeOH (5 mL) containing ca. 5% (by vol) dichloroethane in a threaded culture tube. The tube was capped and heated at 80 ℃ for 5 h. After the reaction solution was concentrated, the residue was passed through a pipet column of silica gel (elution with 3:1 hexanes/EtOAc) and the filtrate was reconcentrated. The residue was purified by MPLC (15:1 hexanes/EtOAc), to provide, in order of elution, **38** (3.6 mg, 18%) and **37** (8.1 mg, 38%), each as a yellow oil.

Data for 37

^{1}H NMR (C_6D_6, 500 MHz): δ 7.48 (s, 1H, H2), 7.33 (ddd, *J* = 7.9, 1.0, 1.0 Hz, 1H, H8), 7.19 (nfod, J_{app} = 8.5 Hz, 2H, H3'+H5'), 7.09 (ddd, *J* = 7.5, 7.5 1.2 Hz, 1H, H6 or H7), 7.05 (nfod, J_{app} = 8.5 Hz, 2H, H2'+H6'), 6.94 (ddd, *J* = 7.5, 7.5, 1.0 Hz, 1H, H6 or H7), 6.62 (ddd, *J* = 7.5, 0.9, 0.9 Hz, 1H, H5), 3.94 (s, 2H, C9H_2), 3.21 (s, 3H, OCH_3), 2.64 (nfom, 2H, CH_2CH$_2$SiEt$_2$OMe), 1.41 [s, 9H, C(CH_3)$_3$], 0.93 (nfom, 2H, CH$_2$CH_2SiEt$_2$OMe), 0.89 [t, *J* = 8.1 Hz, 6H, Si(CH$_2$CH_3)$_2$OMe], and 0.48 [q, *J* = 8.1 Hz, 4H, Si(CH_2CH$_3$)$_2$OMe].

^{13}C{^{1}H} NMR (C_6D_6, 126 MHz): δ 146.5, 144.2, 142.8, 142.0, 141.2, 139.3, 138.6, 133.6, 133.4, 131.7, 129.4, 126.7, 126.5, 125.2, 124.6, 123.2, 50.4, 38.6, 35.9, 30.9, 27.5, 16.4, 6.9, and 4.8.

HRMS (ESI) *m/z*: [M + H]$^+$ Calcd for $C_{30}H_{38}{}^{35}ClOSi$ 477.2375; Found 477.2381 (2%); [M - OMe]$^+$ Calcd for $C_{29}H_{34}{}^{35}ClSi$ 445.2113; Found 445.2119 (44%); [M + H – MeOH - isobutylene]$^+$ Calcd for $C_{25}H_{26}{}^{35}ClSi$ 389.1487; Found 389.1492 (100%)

IR (thin film): 3071, 2954, 2876, 1603, 1482, 1204, and 1085 cm^{-1}.

Data for 38

^{1}H NMR ($CDCl_3$, 500 MHz): δ 7.68 (s, 1H, H2), 7.48 (ddd, *J* = 7.9, 1.0, 1.0 Hz, 1H, H8), 7.46 (nfod, J_{app} = 8.5 Hz, 2H, H3'+H5'), 7.28 (nfod, J_{app} = 8.5 Hz, 2H, H2'+H6'), 7.17 (ddd, *J* = 7.5, 7.5 1.2 Hz, 1H, H6 or H7), 6.98 (ddd, *J* = 7.5, 7.5 1.0 Hz, 1H, H6 or H7), 6.21 (ddd, *J* = 7.5, 0.9, 0.9 Hz, 1H, H5), 4.17 (s, 2H, C9H_2), 1.55 [s, 9H, C(CH_3)$_3$], 0.86 [t, *J* = 8.0 Hz, 6H, Si(CH$_2$CH_3)$_2$], 0.49 [dq, *J* = 15.4, 7.7 Hz, 2H, Si(CH_aH$_b$CH3)$_2$], and 0.43 [dq, *J* = 15.4, 7.7 Hz, 2H, Si(CH$_a$$H_b$CH3)$_2$].

^{13}C{^{1}H} NMR ($CDCl_3$, 126 MHz): δ 145.1, 143.5, 142.4, 141.3, 140.5, 140.3, 140.1, 133.8, 133.7, 131.45, 131.35, 128.7, 126.32, 126.31, 124.3, 123.0, 50.9, 38.7, 36.2, 31.0, 7.0, and 5.7.

HRMS (ESI) *m/z*: [M + H]$^+$ Calcd for $C_{28}H_{34}{}^{35}ClOSi$ 449.2062; Found 449.2047 (7%). [M + H – $HOCH_3$]$^+$ Calcd for $C_{27}H_{30}{}^{35}ClSi$ 417.1800; Found 417.1788 (100 %).

IR (thin film): 3070, 2956, 2875, 1494, 1604, 1364, and 1158 cm^{-1}.

(2-(1-(*tert*-Butyl)-4-(4-chlorophenyl)-9*H*-fluoren-3-yl-9-*d*)ethyl)diethyl(methoxy-*d*$_3$)silane (37-D) and (2-(1-(*tert*-Butyl)-4-(4-chlorophenyl)-9*H*-fluoren-3-yl-9-*d*)diethyl(methoxy-*d*$_3$)silane (38-D)

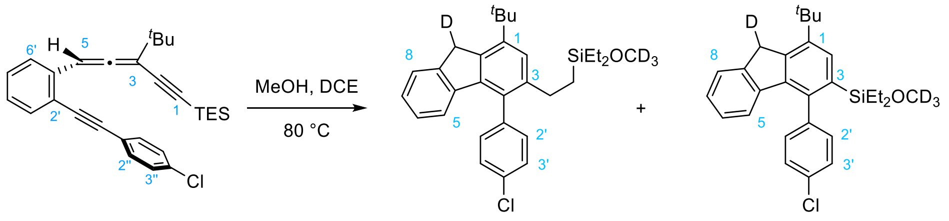

36 **37-D** **38-D**

Allenyne **36** (20 mg, 0.050 mmol) was dissolved in CD_3OD (5 mL) containing ca. 5% of 1,2-dchloroethane in a threaded culture tube. The tube was capped and heated at 80 ℃ for 5 h. After the reaction solution was concentrated, the residue was passed through a pipet column of silica gel (elution with 3:1 hexanes/EtOAc) and the filtrate was reconcentrated. The residue was purified by MPLC (15:1 hexanes/EtOAc), to provide, in order of elution, **38-D** (4.3 mg, 21%) and **37-D** (9.7 mg, 38%), each as a yellow oil.

Data for 37-D

^{1}H NMR (C_6D_6, 500 MHz): δ 7.48 (s, 1H, H2), 7.33 (ddd, *J* = 7.9, 1.0, 1.0 Hz, 1H, H8), 7.19 (nfod, J_{app} = 8.5 Hz, 2H, H3'+H5'), 7.09 (ddd, *J* = 7.5, 7.5 1.2 Hz, 1H, H6 or H7), 7.05 (nfod, J_{app} = 8.5 Hz, 2H, H2'+H6'), 6.94 (ddd, *J* = 7.5, 7.5, 1.0 Hz, 1H, H6 or H7), 6.62 (ddd, *J* = 7.5, 0.9, 0.9 Hz, 1H, H5), 3.91 (br s, 1H, H9), 2.64 (nfom, 2H, C*H*$_2$CH$_2$SiEt$_2$OMe), 1.41 [s, 9H, C(CH$_3$)$_3$], 0.93 (nfom, 2H, CH$_2$C*H*$_2$SiEt$_2$OMe), 0.89 [t, *J* = 8.1 Hz, 6H, Si(CH$_2$C*H*$_3$)$_2$OMe], and 0.48 [q, *J* = 8.1 Hz, 4H, Si(C*H*$_2$CH$_3$)$_2$OMe].

^{13}C{^{1}H} NMR (C_6D_6, 126 MHz): δ 146.6, 144.2, 142.8, 142.0, 141.2, 139.3, 138.5, 133.6, 133.4, 131.7, 129.4, 126.7, 126.4, 125.2, 124.6, 123.2, 38.1 (v weak), 35.9, 30.9, 30.1, 27.5, 16.4, 6.9, and 4.8. [resonance for O*C*D$_3$ not observed above the noise.]

HRMS (ESI) *m/z*: [M + H]$^+$ Calcd for $C_{30}H_{34}D_4{}^{35}ClOSi$ 481.2626; Found 481.2632 (2%). [M + H – HOCD$_3$]$^+$ Calcd for $C_{29}H_{33}D^{35}ClSi$ 446.2176; Found 446.2180 (40%).

IR (thin film): 3070, 2954, 2875, 2221, 2062, 1607, 1483, 1123, and 1077 cm^{-1}.

Data for 38-D

^{1}H NMR ($CDCl_3$, 500 MHz): δ 7.68 (s, 1H, H2), 7.48 (ddd, *J* = 7.9, 1.0, 1.0 Hz, 1H, H8), 7.46 (nfod, J_{app} = 8.5 Hz, 2H, H3'+H5'), 7.28 (nfod, J_{app} = 8.5 Hz, 2H, H2'+H6'), 7.17 (ddd, *J* = 7.5, 7.5 1.2 Hz, 1H, H6 or H7), 6.98 (ddd, *J* = 7.5, 7.5 1.0 Hz, 1H, H6 or H7), 6.21 (ddd, *J* = 7.5, 0.9, 0.9 Hz, 1H, H5), 4.15 (br s, 1H, H9), 1.55 [s, 9H, C(CH$_3$)$_3$], 0.86 [t, *J* = 8.0 Hz, 6H, Si(CH$_2$C*H*$_3$)$_2$], 0.49 [dq, *J* = 15.4, 7.7 Hz, 2H, Si(C*H*$_a$H$_b$CH3)$_2$], and 0.43 [dq, *J* = 15.4, 7.7 Hz, 2H, Si(CH$_a$*H*$_b$CH3)$_2$].

^{13}C{^{1}H} NMR ($CDCl_3$, 126 MHz): δ 145.1, 143.5, 142.4, 141.3, 140.5, 140.3, 140.1, 133.77, 133.75, 131.45, 131.35, 128.7, 126.33, 126.30, 124.3, 123.0, 50.1 (weak), 38.4 (C9, t, $^1J_{CD}$ = 20 Hz), 36.2, 31.0, 7.0, and 5.7.

HRMS (ESI) *m/z*: [M + H]$^+$ Calcd for $C_{28}H_{30}D_4{}^{35}ClOSi$ 453.2313; Found 453.2321 (1%); [M + H – HOCD$_3$]$^+$ Calcd for $C_{27}H_{29}D^{35}ClSi$ 418.1863; Found 418.1868 (20%).

IR (thin film): 3070, 2956, 2875, 2213, 2062, 1605, 1431, 1246, and 1122 cm^{-1}.

IV. Computational Results

a. **Methods**

b. **Discussion of results**

- **i/ii) Figures S1/S2.** PESs for **43d** and **43g** to products **44a/b** and **45a/b**; and spin populations and charge distributions in DHTs **44a/b** and **45a/b.**
- **iii) Figure S3.** Expanded version of the PES for the formation of **29–31** from **11g.**
- **iv) Figure S4.** Transition structures and energies for the competitive conversions of **S54** to **S55** vs. **S56**.
- **v) Figure S5.** DFT analyses to assess the differential strain energies within the tethering atoms in diradicals **S57** *vs.* **S58**.

c. **Co-ordinates and energies for each stationary state**

IVa. Methods

The DFT calculations were carried out with Gaussian 16.[6] The geometry of each stationary point was first optimized in the gas phase at the B3LYP-GD3BJ[7]/6-31G level of theory. The nature of each optimized structure was verified by frequency calculation (298 K and at the same level of theory). Those obtained geometries were further optimized at the same level of theory, using the SMD(dichloroethane) solvation model.[8] Single point calculations were then carried out for each of the optimized geometries using SMD(dichloroethane)/B3LYP15-GD3BJ/6-311++G(d,p). The thermal correction for the Gibbs energy (298 K) from the initial optimization calculations were then applied.

More explicitly, each closed-shell structure was subjected to an initial conformational search in Maestro (Version 10.1.013, MMshare Version 2.9.013, Release 2015-1, Platform Linux-x86_64) in the Schrödinger software package. These were then ported to DFT geometry optimizations and frequency calculations by the above protocol. One of the duplicate conformers from any pairs of optimized geometries having virtually identical energies was discarded. The lowest energy of each of these initial DFT-optimized geometries was then subjected to the single-point calculation for each structure. Finally, the energies of the resulting ensemble of conformers were Boltzmann averaged and those energies are given in the PES diagrams.

For all open-shell calculations (i.e., diradicals and the stepwise transition state structures leading to or from a diradical), spin symmetry was broken during the optimization using the keyword “guess=(mix, always).” For each open-shell singlet diradical structure, the initial geometry was generated by initial optimization of the triplet diradical structure; the resulting geometry was then further optimization as the singlet. A "stabilization” calculation (keyword "stable=opt") was used to reoptimize the wavefunction of the triplet/singlet diradical structure.

To search for each transition structure, a “Scan” calculation was used. The structure at the maximum from the scan was used as the initial guess of the geometry of the transition structure.

IVb. Discussion of results

i/ii) **Figures S1/S2.** PESs for **43d** and **43g** to products **44a/b** and **45a/b**; and spin populations and charge distributions in DHTs **44a/b** and **45a/b**.

DFT methods were used to gain insights about the potential energy surfaces of the overall transformations of the model allenynes **43d** and **43g** to DHT **44a/45a** (zwitterion) or **44b/45b** (diradical), respectively. The results are summarized in Figure S1. Different internal allenynophiles [X = CPh (**43d**) or X = N (**43g**)] have an impact on the relative free energies (ΔG), but they do not alter the fundamental nature of the mechanism for each. The first elementary step is a *5-exo-dig* cyclization between the tethered allenyne and the proximal allenynophile carbon atom (alkyne/nitrile) in **43d/43g**. This generates the diradical intermediate **S46/S47**, which subsequently undergoes *6-endo-dig* cyclization to produce the distinct DHTs **44a/45a** (zwitterion) or **44b/45b** (diradical). The rate-determining step is the initial *5-exo-dig* cyclization for both substrates; the nitrile analog has a significantly higher activation barrier [35.0 vs. 24.7 kcal mol^{-1}] for that event. This result parallels our experimental observation for the cyclization temperature (cf. Figures 2b and 2c in the manuscript, 80 °C for **11d** and 150 °C for **11g**). This outcome is in accordance with a bond energy argument: a C≡C bond is ca. 9 kcal mol^{-1} weaker than a C≡N bond.[9] As a result, there is a greater energetic penalty for breaking a C≡N to generate a C=N· compared to forming a C=C· from a C≡C. Larger ΔGs (vs. the starting substrate) for all the species in the nitrogen analog can be rationalized by analogous arguments.

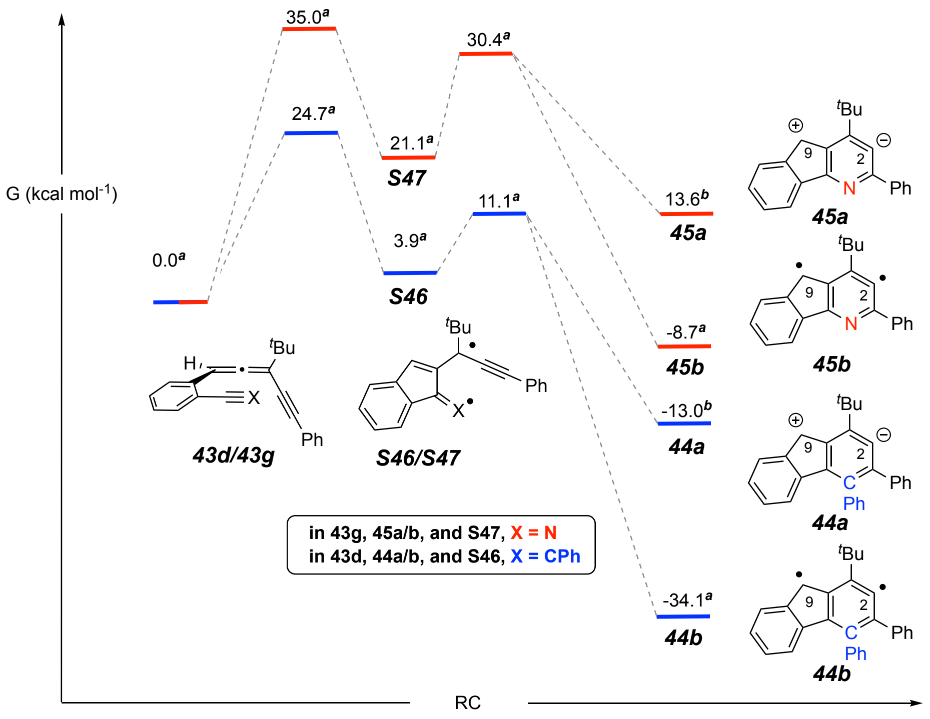

Figure S1. DFT calculations of the free energies (G) of species on the potential energy surface for the cyclization of allenynes **43d** (alkyne) and **43g** (nitrile) to produce the discrete DHTs **44a/b** and **45a/b** via diradical intermediates **S46/S47**. Energies are normalized to G = 0 for both **43d** and **43g**. Red bars: X = N. Blue bars: X = C-Ph.

[a] SMD(dichloroethane)/UB3LYP-GD3BJ/6-311++G(d,p)//SMD(dichloroethane)/UB3LYP-GD3BJ/6-31G

[b] SMD(dichloroethane)/RB3LYP-GD3BJ/6-311++G(d,p)//SMD(dichloroethane)/RB3LYP-GD3BJ/6-31G

We also examined the nature of the electron distributions in these isomeric DHTs. The zwitterionic DHTs **44a/45a** are always of considerably higher free energy than their diradical counterparts **44b/45b** [ΔG° = 21.1 kcal mol^{-1} when X = CPh and ΔG° = 22.3 kcal mol^{-1} when X = N; Figure S1]. We used Hirshfield[10] charge density and spin population analyses to evaluate the electronic structures of the four species. No significant charges are found in diradical DHTs **44b** or **45b** using an unrestricted B3LYP functional, but considerable spin population is detected on both carbons C2 and C9. More spin population on C2 is observed, indicating that the radical on C2 is more localized compared to the benzylic radical on C9 (+0.76 vs -0.36 in **44b**, and +0.75 vs -0.37 in **45b**). To capture the zwitterionic DHTs, a restricted functional (RB3LYP) was used for the computation. A single point computation was performed

using the optimized geometries for the diradical DHTs **44b/45b**. For the zwitterionic DHT **44a**, only a small amount of negative charge (-0.10) is found at C2 while no net charge is seen at C9. This result is consistent with this DHT possessing much of the character represented by the neutral carbene resonance contributor **44a:**. For the nitrogen analog **45a**, a similar result is observed at both C2 and C9 as for **44a**, again implying considerable contribution to the hybrid from the carbene contributor **45a:**.

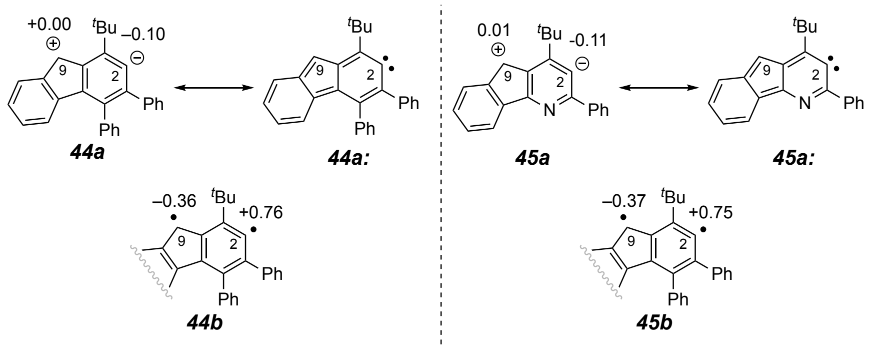

Figure S2. Charge distribution in zwitterionic DHTs **44a** and **45a** and spin population in diradical DHTs **44b** and **45b**.

IVb. Discussion of results

iii) **Figure S3.** Expanded version of the PES for the formation of **29–31** from **11g.**

A detailed potential energy surface diagram of the transformation of the model diradical **45b** (the PMP in **11g** has been replaced by a phenyl group) leading to products **S48–S50** is shown in Figure S3. The rate limiting event is the internal hydrogen atom abstraction that converts **45b** to the isomeric diradical **S51**. The activation barriers for the forward reaction steps that further convert **S51** into each of the products **S48**–**S50** are all quite low. Collectively, two pairs of competitive events, i) **S51** to product **S48** vs. spirocyclopropane **S52** and ii) **S53** to products **S49** vs. **S50**, have relative bifurcation activation barriers that are in close alignment with the observed product ratios.

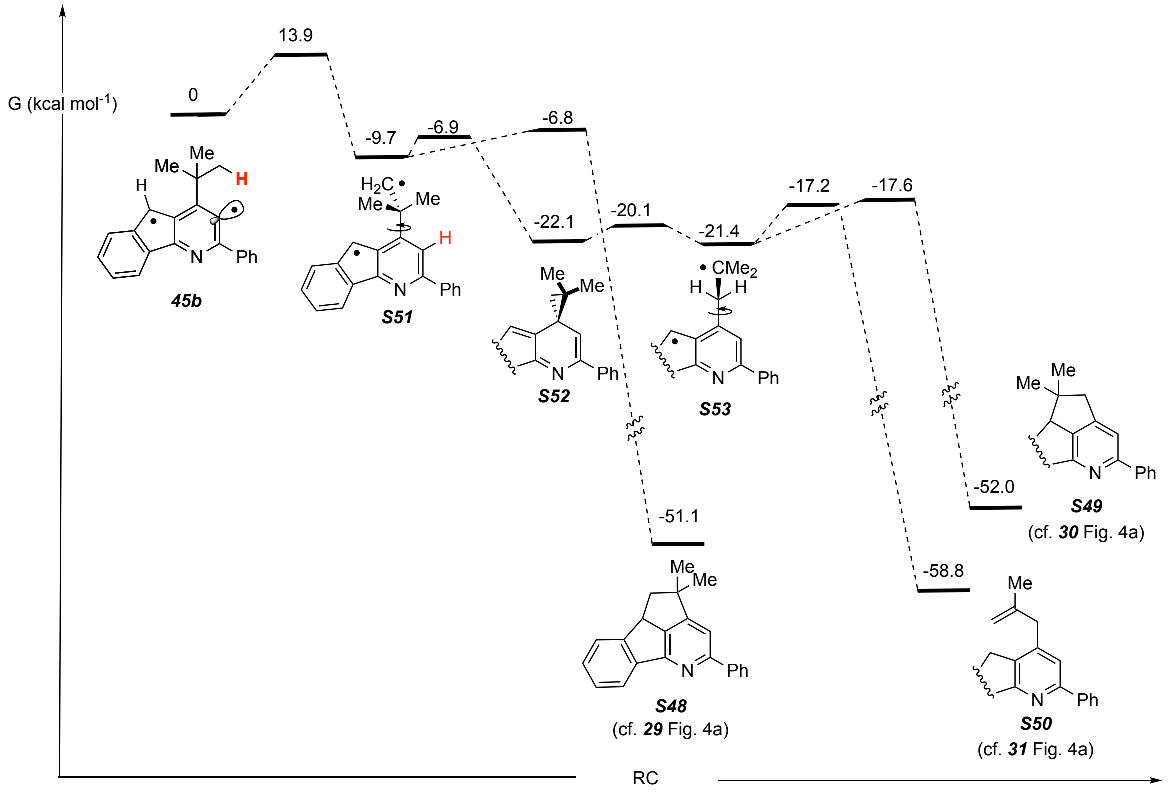

Figure S3. Potential energy surface diagram for the conversion of the diradical **45b** to products **S48**, **S49,** and **S50**. SMD(dichloroethane)/UB3LYP-GD3BJ/6-311++G(d,p)//SMD(dichloroethane)/UB3LYP-GD3BJ/6-31G

IVb. Discussion of results

iv) Figure S4. Transition structures and energies for the competitive conversions of **S54** to **S55** vs. **S56**.

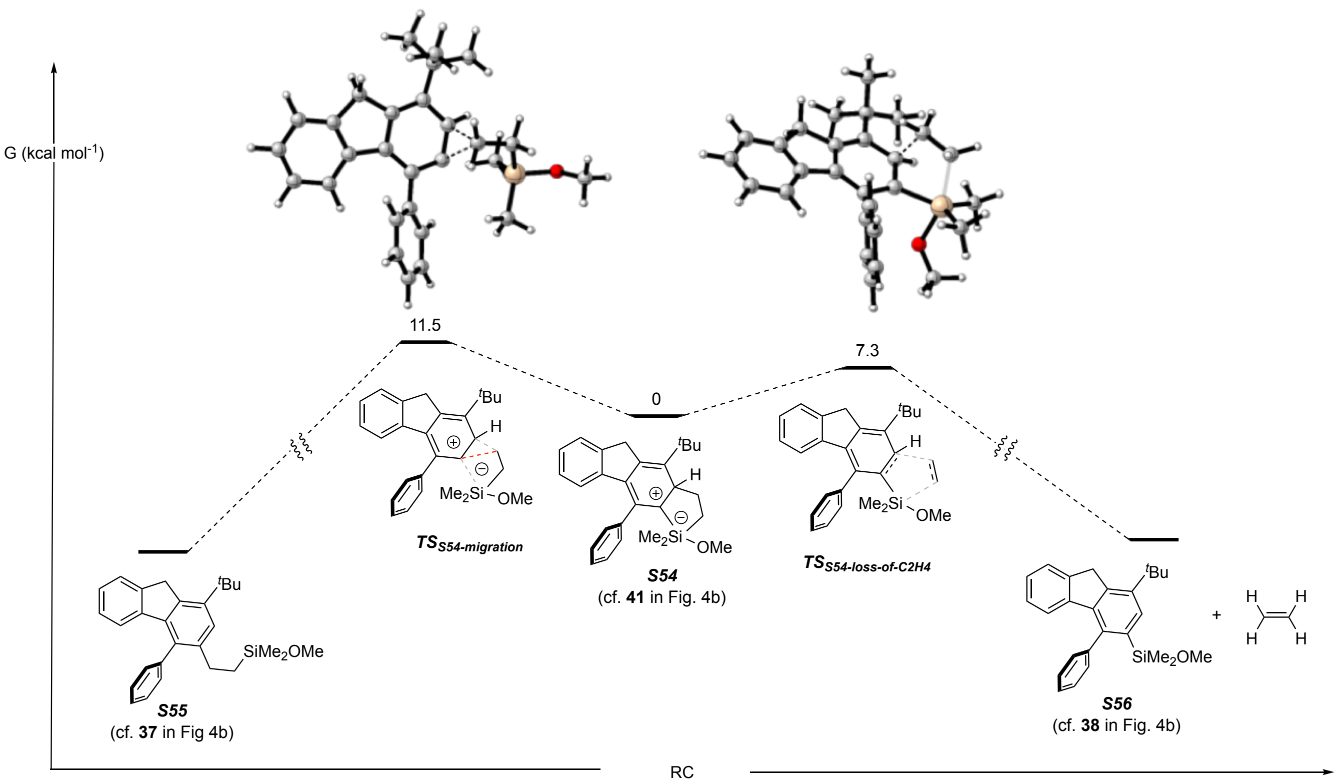

Figure S4. Activation barriers for the two reaction pathways of **S54** (an analog of the species studied experimentally: the diethylsilyl moiety is replaced by dimethylsilyl and the PCP by Ph) leading to rearrangement product **S55** vs. fragmentation product **S56**. SMD(dichloroethane)/UB3LYP-GD3BJ/6-311++G(d,p)//SMD(dichloroethane)/UB3LYP-GD3BJ/6-31G

IVb. Discussion of results

v) **Figure S5.** DFT analyses to assess the differential strain energies within the tethering atoms in diradicals **S57** *vs.* **S58**.

To gain more insights on the reason why the cyclobutene product **12h'** was only observed from substrate **11h**, we used significantly simplified model structures **S57** and **S58** (Figure S5) of the actual diradical intermediates arising from **10a** and **11h**. The activation barriers for the two competing cyclization pathways (*4-exo* vs. *6-endo*) were explored. Note that **S57** has a 2-atom-linker between the allenyne motif and the pedant alkyne whereas **S58** has a 3-atom-linker between those two motifs. For diradical **S57**, the *6-endo* cyclization has a 4.5 kcal/mol lower barrier compared with the *4-exo* process (5.9 vs. 10.4). However, the *6-endo* cyclization is less favorable than the *4-exo* in diradical **S58** (6.1 vs. 1.6). Formation of cyclobutene **S60** from **S58** is more exergonic than the formation of **S59** from **S57** ($\Delta G°$ of –28.9 vs. –13.5 kcal mol^{-1}). Since the bond types are the same in structures **S59** and **S60**, this energy difference might arise principally from the greater amount of strain in **S59** (cyclobutene fused to cyclopentene) than in **S60** (cyclobutene fused to cyclohexene). This would likely translate to a larger energy penalty due to strain in $\mathbf{TS}_{\mathbf{S57\text{-}4\text{-}exo}}$ than that in $\mathbf{TS}_{\mathbf{S58\text{-}\mathit{4\text{-}exo}}}$.

We then used the distortion/interaction-activation strain model[11] to evaluate the stain energy present in the four TSs shown in the PES. Guided by protocols advanced for applying this model to *intramolecular* cycloadditions,[12] we first disassembled the two diradicals **S57** and **S58** as well as the four TSs given in the PES (left of Figure S5) into 3 pieces, shown as **A**, **B**, and **C1** (right in Figure S5). The geometry for **A** was generated by: 1. extracting the indicated atoms from the optimized geometry of each diradical and each TS; 2. adding a **H** atom (purple font) to the "broken" C-C bond (and two **H** atoms to the "broken" C=C bond) that were present in each diradical and each TS; and 3. holding the backbone of each of these fragments frozen, the positions of the newly added **H** atoms were DFT-optimized. The same operation was repeated to generate the geometries of **B** and **C1** for each diradical and each TS. The "strain energies" tabulated to the right of the PES in Figure S5 were obtained by subtracting the the Gibbs energy of each fragment (**A**, **B**, and **C1**) in the appropriate diradical from the Gibbs energy of the fragment in each of the four corresponding transition states.

Using this approach, we found there was almost no strain built up in piece **C1** and that a negative total strain energy (**A**+**B**+**C**1) was seen for both of the *4-exo* cyclizations. This indicated that this strategy was not suitable for the task at hand. We hypothesized that because we were studying an intramolecular diradical cyclization in which the tethering moiety <u>also included a fused ring</u>, that the methods described for intramolecular pericyclic reactions tethered by a simple chain[12] are not applicable here. We conjectured that most of the strain in the possible TSs was originating from <u>within the cyclopenteno or cyclohexeno moieties</u> and its attachments. We used several complementary approaches to assess this.

Clearly, the simple **C1** fragment had not revealed any significant differences in strain. We then used, sequentially, fragments **C2**–**C6**, each of which contains the two directly bound carbon atoms present in the diradicals **S57** and **S58**. In **C2**, fixed as a singlet diradical, three hydrogen atoms (**H**) were added. The substantially negative strain energy for both 4-exo TSs again indicated that this was a flawed approach. The bonding interaction in **C2** likely masks any differences in strain energy we were attempting to assess. Next we completely saturated the attached carbon atoms by adding a total of 5 hydrogens (**H**) to generate **C3**, removing all bonding interaction from the diradical. Significant strain energy was then observed for the *4-exo* process and the value in $\mathbf{TS}_{\mathbf{S57\text{-}\mathit{4\text{-}exo}}}$ was 15.7 kcal/mol larger than that in $\mathbf{TS}_{\mathbf{S58\text{-}\mathit{4\text{-}exo}}}$! (27.6 vs 11.9 kcal/mol). We observed similar results when we used fragments **C4** or **C5** (each with four attached **H**s). Little strain was present in either *6-endo* cyclization. Significant strain was seen for the *4-exo* cyclizations and, again, a greater amount was present in the 4-exo cyclization when the linker was cyclopenteno (i.e., in $\mathbf{TS}_{\mathbf{S57\text{-}\mathit{4\text{-}exo}}}$) than when cyclohexeno (i.e., in $\mathbf{TS}_{\mathbf{S58\text{-}\mathit{4\text{-}exo}}}$).

Finally, we used fragment **C6** (a triplet diradical) as the model. The benefit was that the diradical structure was maintained so it was the closest analog to the diradical motif in **S57** and **S58**. The triplet nature obliterated the bonding interaction present in **C2**, as evidenced by both the geometries and energies of **C6** vs. **C2**. Significantly more strain energy was found again in $\mathbf{TS}_{\mathbf{S57\text{-}\mathit{4\text{-}exo}}}$ vs. $\mathbf{TS}_{\mathbf{S58\text{-}\mathit{4\text{-}exo}}}$. (25.1 vs. 11.9 kcal/mol).

As a conclusion, although we were able to identify trends in the differential strain energies that paralleled the experimental observations, clearly the fragments used here were not well-suited to match the results in a quantitative manner. This likely originates from the unorthodox nature of the tethering moities themselves and the complexities introduced by the delocalized diradicals present in these processes.

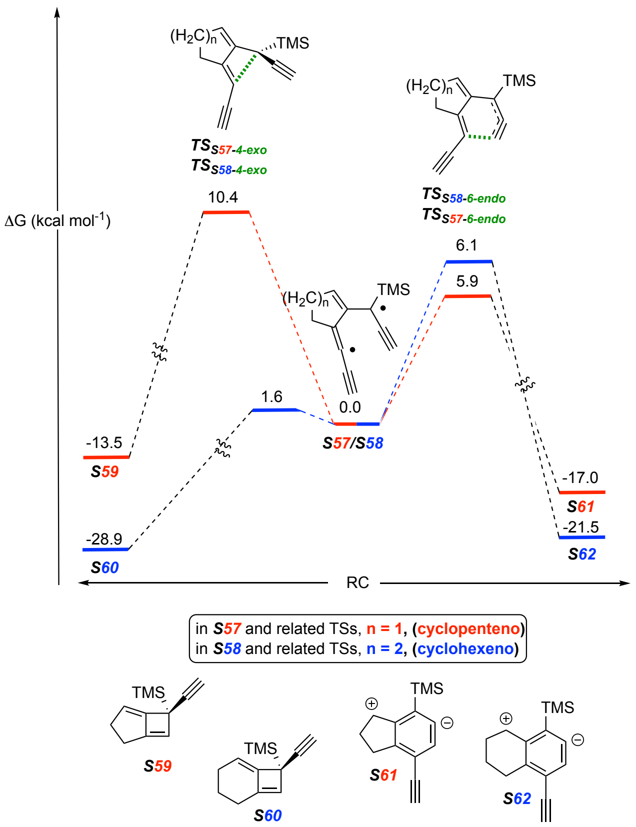

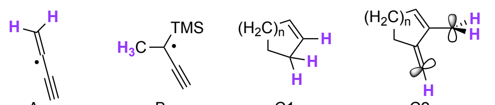

		A	B	C1	C2
Strain Energy (kcal mol-1) = $G_{fragment\_in\_TS} - G_{fragment\_in\_diradical}$					
n = 1	*TS*$_{S57\text{-}4\text{-}exo}$	1.4	-3.3	0.6	-12.8
n = 1	*TS*$_{S57\text{-}6\text{-}endo}$	2.4	4.8	-0.1	0.8
n = 2	*TS*$_{S58\text{-}4\text{-}exo}$	0.8	-2.1	0.1	-10.1
n = 2	*TS*$_{S58\text{-}6\text{-}endo}$	2.2	3.1	-0.4	3.6

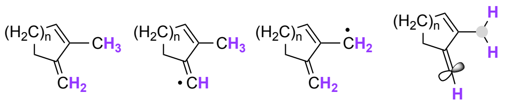

		C3	C4	C5	C6
Strain Energy (kcal mol-1) = $G_{fragment\_in\_TS} - G_{fragment\_in\_diradical}$					
n = 1	*TS*$_{S57\text{-}4\text{-}exo}$	27.6	16.4	34.3	25.1
n = 1	*TS*$_{S57\text{-}6\text{-}endo}$	-0.5	-0.1	-0.2	0.2
n = 2	*TS*$_{S58\text{-}4\text{-}exo}$	11.9	5.6	22.0	11.9
n = 2	*TS*$_{S58\text{-}6\text{-}endo}$	-0.3	0.1	0.5	0.6

Figure S5. DFT analyses to assess the differential strain energies imposed by the tethering atoms in **S57** and **S58**. These support the observation that *4-exo* cyclization is less favorable in the cyclopenteno diradical **S57** while *6-endo* cyclization is less favorable in the cyclohexeno diradical **S58**. That is, there is little difference in the strain energy for the 6-endo cyclizations, regardless of the ring size fused within the tether, so that the greater stain localized within the entire set of tethering atoms (i.e., including those in the fused carbocycle) of **TS**$_{S57\text{-}4\text{-}exo}$ compared to that in **TS**$_{S58\text{-}4\text{-}exo}$ is principally responsible for the observed selectivity.

For the zwitterions **S61** and **S62**: SMD(dichloroethane)/RB3LYP-GD3BJ/6-311++G(d,p)//SMD(dichloroethane)/RB3LYP-GD3BJ/6-31G. For all other structures: SMD(dichloroethane)/UB3LYP-GD3BJ/6-311++G(d,p)//SMD(dichloroethane)/UB3LYP-GD3BJ/6-31G

.

IVc. Co-ordinates and energies for each stationary state

The following information is given for each of the calculated stationery states for the structures shown in Figure S6.

(1) a CYLview20[13]-generated three-dimensional image of the optimized structure
(2) the sum of the electronic and thermal free energies in Hartree
(3) the imaginary frequency for each transition state structure
(4) the Cartesian coordinates obtained from the geometry optimization calculations

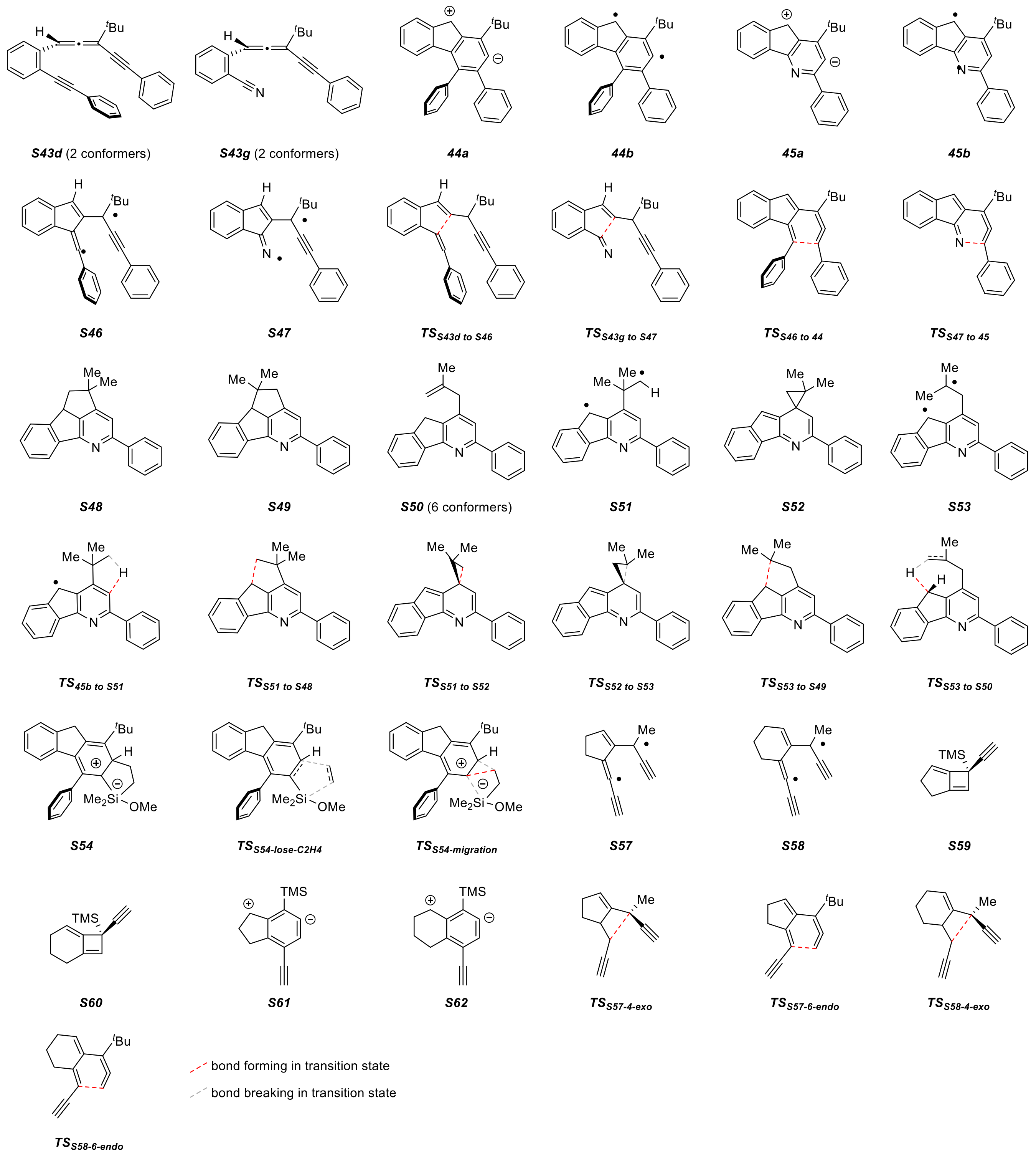

Figure S6. Structure and number for each of the stationery states provided on the following series of pages.

Geometry and free energy for 43d (conformer 1)

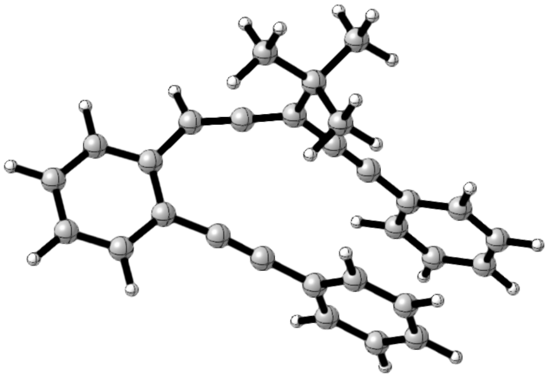

Sum of electronic and thermal Free Energies= -1119.456708

Center Number	Atomic Number	Atomic Type	Coordinates (Angstroms) X	Y	Z
1	6	0	-4.057062	-2.416827	0.434968
2	6	0	-3.224204	-1.372210	-0.025056
3	6	0	-3.769809	-0.382220	-0.891230
4	6	0	-5.116354	-0.501500	-1.284945
5	6	0	-5.924945	-1.539922	-0.822861
6	6	0	-5.393825	-2.500032	0.047595
7	6	0	-1.848002	-1.389146	0.358634
8	6	0	-3.006212	0.766031	-1.413670
9	6	0	-1.916344	1.298905	-0.904721
10	6	0	-0.859911	1.892612	-0.375321
11	6	0	-0.988166	3.026772	0.677365
12	6	0	0.456903	1.453699	-0.705616
13	6	0	1.594267	1.064455	-0.916472
14	6	0	2.912292	0.562470	-1.113267
15	6	0	3.975897	1.013869	-0.300270
16	6	0	5.257718	0.486665	-0.461263
17	6	0	5.501814	-0.493764	-1.432266
18	6	0	4.454546	-0.942895	-2.247404
19	6	0	3.168755	-0.422601	-2.092729
20	6	0	-0.372780	2.531131	2.006160
21	6	0	-2.465007	3.394351	0.901643
22	6	0	-0.214522	4.264814	0.171126
23	1	0	-3.631712	-3.166438	1.093187
24	1	0	-5.527082	0.243978	-1.958746
25	1	0	-6.960717	-1.600944	-1.139495
26	1	0	-6.012894	-3.312127	0.413276
27	1	0	-3.440576	1.230916	-2.300658
28	1	0	3.780898	1.764585	0.457540
29	1	0	6.065814	0.836702	0.172660
30	1	0	6.499291	-0.903345	-1.552917
31	1	0	4.639110	-1.702017	-3.000442
32	1	0	2.353473	-0.776186	-2.714106
33	1	0	0.677822	2.252343	1.876027

34	1	0	-0.427846	3.323252	2.762410
35	1	0	-0.917735	1.657340	2.380228
36	1	0	-2.932592	3.747115	-0.024270
37	1	0	-3.037576	2.535191	1.267747
38	1	0	-2.535109	4.194748	1.646672
39	1	0	-0.640476	4.633480	-0.769166
40	1	0	-0.270312	5.069949	0.913265
41	1	0	0.841345	4.028802	0.000754
42	6	0	-0.682947	-1.518506	0.700139
43	6	0	0.690149	-1.662258	1.069240
44	6	0	1.532060	-2.536447	0.348523
45	6	0	1.230461	-0.923005	2.144146
46	6	0	2.879552	-2.661895	0.693405
47	1	0	1.123466	-3.101076	-0.482268
48	6	0	2.578604	-1.053345	2.480100
49	1	0	0.589681	-0.246256	2.696898
50	6	0	3.407541	-1.921703	1.757210
51	1	0	3.519281	-3.327283	0.123869
52	1	0	2.984467	-0.474731	3.303486
53	1	0	4.457166	-2.012989	2.015089

Geometry and free energy for 43d (conformer 2)

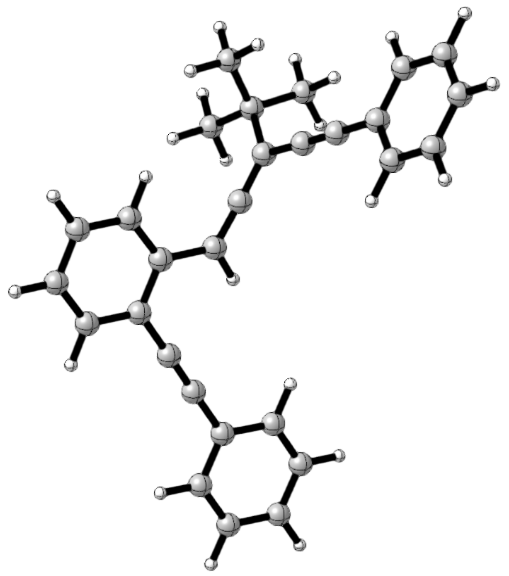

Sum of electronic and thermal Free Energies= -1119.455178

Center Number	Atomic Number	Atomic Type	Coordinates (Angstroms) X	Y	Z
1	6	0	3.032378	2.642657	-2.151032
2	6	0	2.561544	1.669077	-1.240619
3	6	0	1.215943	1.738119	-0.777458
4	6	0	0.394594	2.779780	-1.245680
5	6	0	0.873190	3.732234	-2.143638
6	6	0	2.198651	3.664257	-2.598986
7	6	0	3.443544	0.639320	-0.804412
8	6	0	0.714014	0.733349	0.176451
9	6	0	-0.502533	0.700718	0.677240
10	6	0	-1.724768	0.699348	1.184253
11	6	0	-2.084766	1.467288	2.485293
12	6	0	-2.776740	-0.020597	0.537988
13	6	0	-3.705923	-0.619143	0.019234
14	6	0	-4.784814	-1.319377	-0.595529
15	6	0	-6.078204	-1.283092	-0.027088
16	6	0	-7.132676	-1.968155	-0.632871
17	6	0	-6.918421	-2.698193	-1.809517
18	6	0	-5.639360	-2.740285	-2.380049
19	6	0	-4.578172	-2.058568	-1.782154
20	6	0	-3.190332	2.497022	2.158320
21	6	0	-0.849334	2.196499	3.041012
22	6	0	-2.606031	0.454852	3.530307
23	1	0	4.058342	2.580370	-2.496629
24	1	0	-0.630770	2.832898	-0.895115
25	1	0	0.218234	4.525103	-2.489019
26	1	0	2.576041	4.403096	-3.297616
27	1	0	1.428789	-0.030920	0.479269
28	1	0	-6.241265	-0.717281	0.883815
29	1	0	-8.121620	-1.932893	-0.187813
30	1	0	-7.740628	-3.229134	-2.277668
31	1	0	-5.468970	-3.304340	-3.291174
32	1	0	-3.587262	-2.089355	-2.221857

33	1	0	-4.081688	2.006692	1.752796
34	1	0	-3.479576	3.035342	3.068582
35	1	0	-2.835791	3.229243	1.423739
36	1	0	-0.047366	1.491727	3.286313
37	1	0	-0.457192	2.921976	2.319650
38	1	0	-1.121613	2.736084	3.954885
39	1	0	-1.832740	-0.280941	3.779010
40	1	0	-2.890364	0.980076	4.449744
41	1	0	-3.484671	-0.082129	3.157076
42	6	0	4.213531	-0.236131	-0.441043
43	6	0	5.107527	-1.260373	-0.013973
44	6	0	6.445756	-1.279701	-0.468119
45	6	0	4.668845	-2.272244	0.869970
46	6	0	7.317056	-2.285399	-0.046986
47	1	0	6.786973	-0.505202	-1.146290
48	6	0	5.548101	-3.273439	1.284961
49	1	0	3.642743	-2.262001	1.221126
50	6	0	6.873134	-3.284509	0.829359
51	1	0	8.342284	-2.290312	-0.402057
52	1	0	5.200240	-4.045337	1.963491
53	1	0	7.553453	-4.064642	1.154341

Geometry and free energy for 43g (conformer 1)

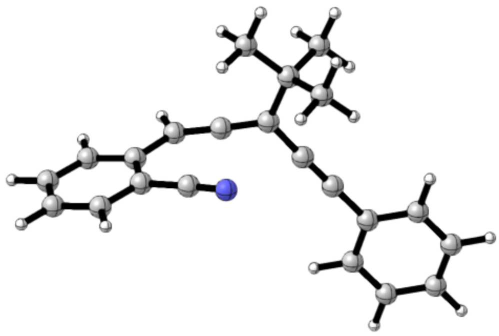

Sum of electronic and thermal Free Energies= -904.486571

Center Number	Atomic Number	Atomic Type	Coordinates (Angstroms) X	Y	Z
1	6	0	-4.372613	-1.533872	1.321405
2	6	0	-3.314047	-0.814425	0.729359
3	6	0	-3.209676	-0.720200	-0.685133
4	6	0	-4.199851	-1.359263	-1.455408
5	6	0	-5.244999	-2.069172	-0.864210
6	6	0	-5.333729	-2.160514	0.530303
7	6	0	-2.381707	-0.185937	1.608729
8	6	0	-2.146740	-0.002168	-1.406578
9	6	0	-1.077983	0.593137	-0.919421
10	6	0	0.015429	1.211903	-0.506959
11	6	0	0.027270	2.718029	-0.128259
12	6	0	1.245032	0.496888	-0.385302
13	7	0	-1.663176	0.313159	2.392506
14	6	0	2.318276	-0.072573	-0.265587
15	6	0	3.569840	-0.740174	-0.126352
16	6	0	4.715153	-0.029590	0.298758
17	6	0	5.939786	-0.685193	0.434235
18	6	0	6.044430	-2.053332	0.150289
19	6	0	4.914194	-2.766054	-0.271387
20	6	0	3.684921	-2.119669	-0.410362
21	6	0	0.554253	2.864346	1.317299
22	6	0	-1.389794	3.309931	-0.222918
23	6	0	0.968374	3.456843	-1.107357
24	1	0	-4.429255	-1.590538	2.402430
25	1	0	-4.136360	-1.292439	-2.536269
26	1	0	-5.988978	-2.550164	-1.489837
27	1	0	-6.142985	-2.710547	0.996349
28	1	0	-2.286379	0.013982	-2.489077
29	1	0	4.630656	1.029167	0.518549
30	1	0	6.812574	-0.129754	0.761413
31	1	0	6.998034	-2.559604	0.257248
32	1	0	4.991041	-3.825720	-0.491680
33	1	0	2.808317	-2.669023	-0.735910
34	1	0	1.574192	2.475674	1.406983
35	1	0	0.565497	3.923478	1.600580
36	1	0	-0.084211	2.320838	2.020925

37	1	0	-1.795565	3.216168	-1.236243
38	1	0	-2.075043	2.809439	0.469516
39	1	0	-1.359939	4.374297	0.035159
40	1	0	0.602330	3.375695	-2.137357
41	1	0	1.021273	4.519620	-0.842934
42	1	0	1.982122	3.043677	-1.068260

Geometry and free energy for 43g (conformer 2)

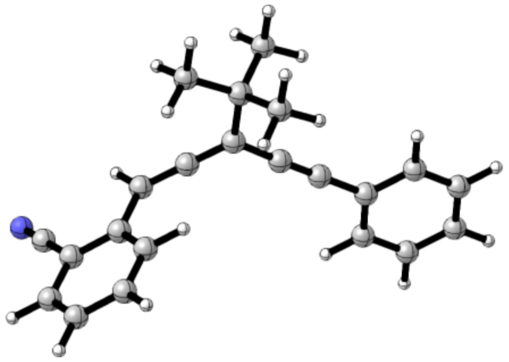

Sum of electronic and thermal Free Energies= -904.488454

Center Number	Atomic Number	Atomic Type	Coordinates (Angstroms) X	Y	Z
1	6	0	-5.106672	-1.704949	0.538931
2	6	0	-4.217364	-1.127747	-0.390865
3	6	0	-3.003592	-0.529543	0.041613
4	6	0	-2.723330	-0.534198	1.420326
5	6	0	-3.605613	-1.105727	2.335253
6	6	0	-4.801950	-1.693822	1.897544
7	6	0	-4.562574	-1.155309	-1.775879
8	6	0	-2.079237	0.076025	-0.932550
9	6	0	-0.940916	0.661306	-0.625126
10	6	0	0.189490	1.262411	-0.294253
11	6	0	0.252883	2.776747	0.045021
12	6	0	1.412074	0.525601	-0.236240
13	7	0	-4.854902	-1.181944	-2.913617
14	6	0	2.480220	-0.062008	-0.173539
15	6	0	3.724711	-0.753691	-0.102951
16	6	0	4.906046	-0.064859	0.253580
17	6	0	6.123307	-0.744394	0.320917
18	6	0	6.184498	-2.114898	0.035794
19	6	0	5.018226	-2.806012	-0.318299
20	6	0	3.795888	-2.135778	-0.388506
21	6	0	0.795222	2.934790	1.483824
22	6	0	-1.145294	3.411259	-0.052554
23	6	0	1.209341	3.466517	-0.953989
24	1	0	-6.025862	-2.155283	0.182590
25	1	0	-1.799927	-0.083093	1.765821
26	1	0	-3.362824	-1.094704	3.392289
27	1	0	-5.487740	-2.137986	2.609686
28	1	0	-2.379075	0.029736	-1.979022
29	1	0	4.855362	0.996070	0.473313
30	1	0	7.024224	-0.205548	0.595314
31	1	0	7.132543	-2.639708	0.089012
32	1	0	5.061589	-3.867274	-0.539845
33	1	0	2.891724	-2.668320	-0.662556
34	1	0	1.788364	2.484715	1.585402
35	1	0	0.873640	3.998695	1.736369
36	1	0	0.124829	2.457989	2.208152
37	1	0	-1.555762	3.317667	-1.063857
38	1	0	-1.846139	2.941067	0.646208

39	1	0	-1.081850	4.477076	0.192847
40	1	0	0.838387	3.367279	-1.980415
41	1	0	1.287647	4.534162	-0.717605
42	1	0	2.213002	3.030744	-0.906691

Geometry and free energy for 44a

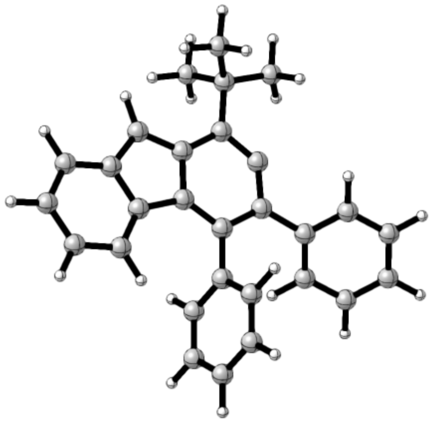

Sum of electronic and thermal Free Energies= -1119.47713

Center Number	Atomic Number	Atomic Type	Coordinates (Angstroms) X	Y	Z
1	6	0	1.060047	-0.411358	0.023652
2	6	0	1.862243	0.799365	0.065610
3	6	0	3.242329	0.427464	0.060591
4	6	0	3.332485	-1.004108	-0.012668
5	6	0	2.002370	-1.551775	-0.049819
6	6	0	-0.337472	-0.382871	0.024617
7	6	0	-0.984320	0.900249	0.033584
8	6	0	-0.145828	2.010773	0.035071
9	6	0	1.231258	2.079557	0.066158
10	6	0	-2.453060	1.111390	0.035760
11	6	0	1.971730	3.422345	0.086670
12	6	0	0.951352	4.581446	0.081034
13	6	0	2.867526	3.557426	-1.170097
14	6	0	2.831878	3.536444	1.369777
15	6	0	-2.984717	2.178496	-0.719085
16	6	0	-4.356916	2.439062	-0.727334
17	6	0	-5.228467	1.641164	0.024561
18	6	0	-4.712212	0.586930	0.788425
19	6	0	-3.339745	0.323627	0.797102
20	6	0	4.455674	-1.843118	-0.070660
21	6	0	4.263421	-3.226025	-0.180188
22	6	0	2.967053	-3.759837	-0.237218
23	6	0	1.830915	-2.928747	-0.173854
24	6	0	-1.135090	-1.641833	-0.006161
25	6	0	-1.179474	-2.482999	1.120110
26	6	0	-1.936261	-3.658519	1.097883
27	6	0	-2.655850	-4.009087	-0.051999
28	6	0	-2.614703	-3.177487	-1.177796
29	6	0	-1.861013	-1.999267	-1.154268
30	1	0	4.090016	1.094871	0.092451
31	1	0	0.293288	4.534543	0.956068
32	1	0	1.479890	5.541334	0.100807
33	1	0	0.325934	4.554190	-0.818529
34	1	0	3.632761	2.778132	-1.216784

35	1	0	2.262608	3.494579	-2.082022
36	1	0	3.372927	4.530569	-1.160837
37	1	0	3.593669	2.754209	1.426121
38	1	0	3.339998	4.508021	1.389290
39	1	0	2.201478	3.461865	2.263332
40	1	0	-2.311206	2.791665	-1.308977
41	1	0	-4.745257	3.259807	-1.321887
42	1	0	-6.295201	1.840119	0.017903
43	1	0	-5.377709	-0.028893	1.384920
44	1	0	-2.957057	-0.484623	1.405964
45	1	0	5.456842	-1.424731	-0.038201
46	1	0	5.121416	-3.888591	-0.227602
47	1	0	2.831963	-4.832330	-0.332997
48	1	0	0.846036	-3.372269	-0.224201
49	1	0	-0.620101	-2.210529	2.009086
50	1	0	-1.965018	-4.297906	1.974311
51	1	0	-3.243845	-4.920951	-0.069417
52	1	0	-3.169386	-3.444147	-2.071638
53	1	0	-1.834953	-1.351168	-2.023908

Geometry and free energy for 44b

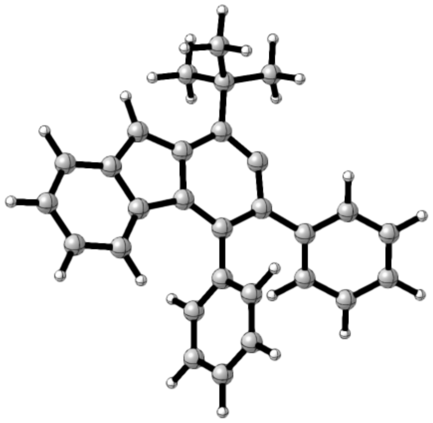

Sum of electronic and thermal Free Energies= -1119.510829

Center Number	Atomic Number	Atomic Type	Coordinates (Angstroms) X	Y	Z
1	6	0	1.060047	-0.411358	0.023652
2	6	0	1.862243	0.799365	0.065610
3	6	0	3.242329	0.427464	0.060591
4	6	0	3.332485	-1.004108	-0.012668
5	6	0	2.002370	-1.551775	-0.049819
6	6	0	-0.337472	-0.382871	0.024617
7	6	0	-0.984320	0.900249	0.033584
8	6	0	-0.145828	2.010773	0.035071
9	6	0	1.231258	2.079557	0.066158
10	6	0	-2.453060	1.111390	0.035760
11	6	0	1.971730	3.422345	0.086670
12	6	0	0.951352	4.581446	0.081034
13	6	0	2.867526	3.557426	-1.170097
14	6	0	2.831878	3.536444	1.369777
15	6	0	-2.984717	2.178496	-0.719085
16	6	0	-4.356916	2.439062	-0.727334
17	6	0	-5.228467	1.641164	0.024561
18	6	0	-4.712212	0.586930	0.788425
19	6	0	-3.339745	0.323627	0.797102
20	6	0	4.455674	-1.843118	-0.070660
21	6	0	4.263421	-3.226025	-0.180188
22	6	0	2.967053	-3.759837	-0.237218
23	6	0	1.830915	-2.928747	-0.173854
24	6	0	-1.135090	-1.641833	-0.006161
25	6	0	-1.179474	-2.482999	1.120110
26	6	0	-1.936261	-3.658519	1.097883
27	6	0	-2.655850	-4.009087	-0.051999
28	6	0	-2.614703	-3.177487	-1.177796
29	6	0	-1.861013	-1.999267	-1.154268
30	1	0	4.090016	1.094871	0.092451
31	1	0	0.293288	4.534543	0.956068
32	1	0	1.479890	5.541334	0.100807
33	1	0	0.325934	4.554190	-0.818529
34	1	0	3.632761	2.778132	-1.216784

35	1	0	2.262608	3.494579	-2.082022
36	1	0	3.372927	4.530569	-1.160837
37	1	0	3.593669	2.754209	1.426121
38	1	0	3.339998	4.508021	1.389290
39	1	0	2.201478	3.461865	2.263332
40	1	0	-2.311206	2.791665	-1.308977
41	1	0	-4.745257	3.259807	-1.321887
42	1	0	-6.295201	1.840119	0.017903
43	1	0	-5.377709	-0.028893	1.384920
44	1	0	-2.957057	-0.484623	1.405964
45	1	0	5.456842	-1.424731	-0.038201
46	1	0	5.121416	-3.888591	-0.227602
47	1	0	2.831963	-4.832330	-0.332997
48	1	0	0.846036	-3.372269	-0.224201
49	1	0	-0.620101	-2.210529	2.009086
50	1	0	-1.965018	-4.297906	1.974311
51	1	0	-3.243845	-4.920951	-0.069417
52	1	0	-3.169386	-3.444147	-2.071638
53	1	0	-1.834953	-1.351168	-2.023908

Geometry and free energy for 45a

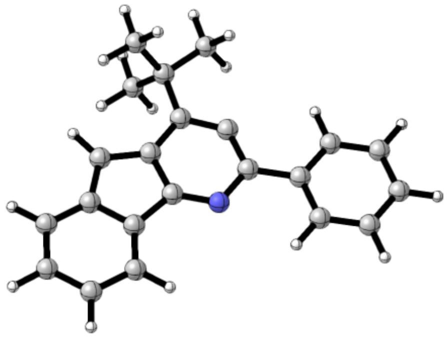

Sum of electronic and thermal Free Energies= -904.466498

Center Number	Atomic Number	Atomic Type	Coordinates (Angstroms) X	Y	Z
1	6	0	-0.873793	-0.709160	0.000001
2	6	0	-1.247248	0.691856	0.000002
3	6	0	-2.673812	0.766315	0.000005
4	6	0	-3.201303	-0.579141	0.000005
5	6	0	-2.106795	-1.505547	0.000002
6	7	0	0.374647	-1.173701	-0.000002
7	6	0	1.403307	-0.256349	-0.000003
8	6	0	1.067041	1.097774	-0.000001
9	6	0	-0.195616	1.657846	0.000001
10	6	0	2.783703	-0.752636	-0.000005
11	6	0	-0.410775	3.174108	0.000003
12	6	0	0.951415	3.899407	0.000000
13	6	0	-1.194292	3.591107	1.269470
14	6	0	-1.194297	3.591109	-1.269461
15	6	0	3.864032	0.152829	-0.000006
16	6	0	5.179199	-0.313543	-0.000009
17	6	0	5.437587	-1.691484	-0.000010
18	6	0	4.368854	-2.598570	-0.000008
19	6	0	3.052037	-2.135451	-0.000006
20	6	0	-4.519467	-1.056596	0.000007
21	6	0	-4.734601	-2.443265	0.000006
22	6	0	-3.656010	-3.342290	0.000003
23	6	0	-2.326403	-2.875074	0.000001
24	1	0	-3.275867	1.662766	0.000007
25	1	0	1.538429	3.638916	-0.888015
26	1	0	0.794188	4.983816	0.000002
27	1	0	1.538433	3.638914	0.888012
28	1	0	-2.181342	3.122594	1.314418
29	1	0	-0.642012	3.310943	2.173645
30	1	0	-1.332527	4.678638	1.275011
31	1	0	-2.181348	3.122596	-1.314404
32	1	0	-1.332532	4.678640	-1.274999
33	1	0	-0.642022	3.310946	-2.173638
34	1	0	3.662515	1.219563	-0.000006
35	1	0	6.002101	0.393816	-0.000010
36	1	0	6.460396	-2.053798	-0.000012

37	1	0	4.563565	-3.666191	-0.000009
38	1	0	2.219614	-2.828719	-0.000005
39	1	0	-5.358905	-0.368699	0.000008
40	1	0	-5.749481	-2.826925	0.000007
41	1	0	-3.847970	-4.410030	0.000003
42	1	0	-1.494351	-3.571351	-0.000001

Geometry and free energy for 45b

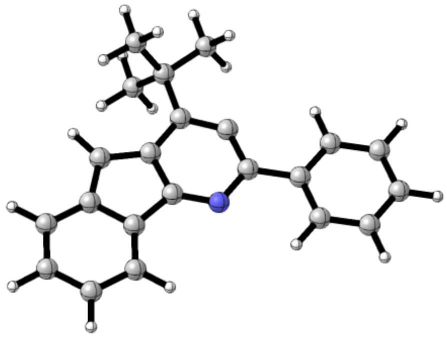

Sum of electronic and thermal Free Energies= -904.502072

Center Number	Atomic Number	Atomic Type	Coordinates (Angstroms) X	Y	Z
1	6	0	-0.873793	-0.709160	0.000001
2	6	0	-1.247248	0.691856	0.000002
3	6	0	-2.673812	0.766315	0.000005
4	6	0	-3.201303	-0.579141	0.000005
5	6	0	-2.106795	-1.505547	0.000002
6	7	0	0.374647	-1.173701	-0.000002
7	6	0	1.403307	-0.256349	-0.000003
8	6	0	1.067041	1.097774	-0.000001
9	6	0	-0.195616	1.657846	0.000001
10	6	0	2.783703	-0.752636	-0.000005
11	6	0	-0.410775	3.174108	0.000003
12	6	0	0.951415	3.899407	0.000000
13	6	0	-1.194292	3.591107	1.269470
14	6	0	-1.194297	3.591109	-1.269461
15	6	0	3.864032	0.152829	-0.000006
16	6	0	5.179199	-0.313543	-0.000009
17	6	0	5.437587	-1.691484	-0.000010
18	6	0	4.368854	-2.598570	-0.000008
19	6	0	3.052037	-2.135451	-0.000006
20	6	0	-4.519467	-1.056596	0.000007
21	6	0	-4.734601	-2.443265	0.000006
22	6	0	-3.656010	-3.342290	0.000003
23	6	0	-2.326403	-2.875074	0.000001
24	1	0	-3.275867	1.662766	0.000007
25	1	0	1.538429	3.638916	-0.888015
26	1	0	0.794188	4.983816	0.000002
27	1	0	1.538433	3.638914	0.888012
28	1	0	-2.181342	3.122594	1.314418
29	1	0	-0.642012	3.310943	2.173645
30	1	0	-1.332527	4.678638	1.275011
31	1	0	-2.181348	3.122596	-1.314404
32	1	0	-1.332532	4.678640	-1.274999
33	1	0	-0.642022	3.310946	-2.173638
34	1	0	3.662515	1.219563	-0.000006
35	1	0	6.002101	0.393816	-0.000010

36	1	0	6.460396	-2.053798	-0.000012
37	1	0	4.563565	-3.666191	-0.000009
38	1	0	2.219614	-2.828719	-0.000005
39	1	0	-5.358905	-0.368699	0.000008
40	1	0	-5.749481	-2.826925	0.000007
41	1	0	-3.847970	-4.410030	0.000003
42	1	0	-1.494351	-3.571351	-0.000001

Geometry and free energy for S46

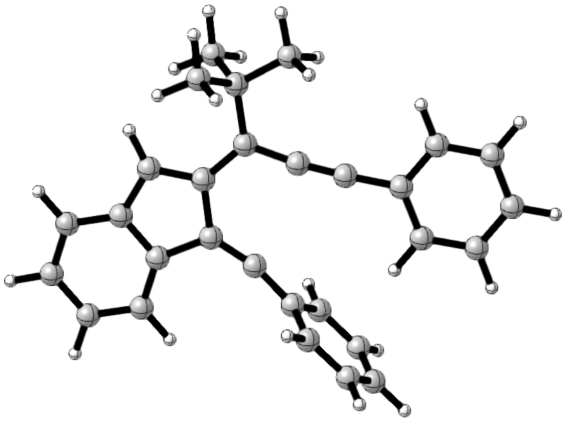

Sum of electronic and thermal Free Energies= -1119.450170

Center Number	Atomic Number	Atomic Type	Coordinates (Angstroms) X	Y	Z
1	6	0	3.349528	-2.458187	-0.000096
2	6	0	2.957245	-1.125159	-0.006014
3	6	0	3.918291	-0.073546	-0.019182
4	6	0	5.288657	-0.377415	-0.026969
5	6	0	5.679901	-1.722208	-0.020778
6	6	0	4.724311	-2.753328	-0.007362
7	6	0	3.224145	1.192511	-0.020762
8	6	0	1.831746	0.990756	-0.007431
9	6	0	1.606015	-0.501746	-0.000859
10	6	0	0.785452	1.947412	0.002059
11	6	0	-0.544857	1.520499	-0.000263
12	6	0	-1.746678	1.250977	-0.004230
13	6	0	-3.135725	0.969186	-0.010679
14	6	0	-4.075432	2.030993	-0.016373
15	6	0	-5.443564	1.761961	-0.024421
16	6	0	-5.905794	0.437899	-0.026486
17	6	0	-4.985619	-0.619623	-0.020257
18	6	0	-3.615000	-0.363682	-0.012513
19	6	0	1.043548	3.477043	0.017219
20	6	0	1.807264	3.903422	-1.263569
21	6	0	1.836747	3.872036	1.290350
22	6	0	-0.285288	4.266342	0.041983
23	1	0	2.612825	-3.255127	0.010197
24	1	0	6.031007	0.414401	-0.036937
25	1	0	6.735768	-1.973157	-0.026106
26	1	0	5.050882	-3.787998	-0.002535
27	1	0	3.732590	2.143306	-0.033616
28	1	0	-3.716509	3.054454	-0.014781
29	1	0	-6.151754	2.584214	-0.029191
30	1	0	-6.971337	0.233519	-0.032846
31	1	0	-5.335492	-1.646830	-0.021578
32	1	0	-2.907799	-1.181048	-0.007722
33	1	0	1.222445	3.651437	-2.155711
34	1	0	1.963305	4.988869	-1.253420
35	1	0	2.783292	3.422754	-1.354262
36	1	0	1.271325	3.600051	2.189101

37	1	0	2.813494	3.388087	1.347925
38	1	0	1.994291	4.957246	1.302032
39	1	0	-0.877118	4.029660	0.932029
40	1	0	-0.063142	5.339146	0.054953
41	1	0	-0.894878	4.055057	-0.842407
42	6	0	0.458926	-1.152000	0.008361
43	6	0	-0.458047	-2.188606	0.013514
44	6	0	-0.981796	-2.702141	1.244915
45	6	0	-0.976262	-2.718583	-1.213343
46	6	0	-1.963949	-3.685255	1.236033
47	1	0	-0.605115	-2.303224	2.180004
48	6	0	-1.958533	-3.701502	-1.195680
49	1	0	-0.595181	-2.332359	-2.151964
50	6	0	-2.463192	-4.190584	0.022379
51	1	0	-2.350644	-4.062153	2.177369
52	1	0	-2.340917	-4.091105	-2.133596
53	1	0	-3.233588	-4.954022	0.025789

Geometry and free energy for S47

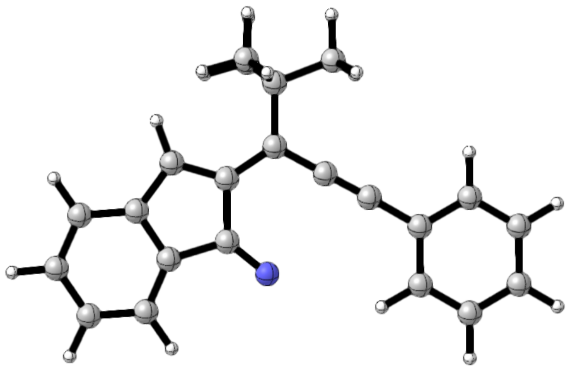

Sum of electronic and thermal Free Energies= -904.454669

Center Number	Atomic Number	Atomic Type	Coordinates (Angstroms) X	Y	Z
1	6	0	-3.217682	-2.731155	0.000019
2	6	0	-2.689097	-1.449676	0.000011
3	6	0	-3.521568	-0.296277	-0.000015
4	6	0	-4.914513	-0.445895	-0.000036
5	6	0	-5.451107	-1.742128	-0.000029
6	6	0	-4.618930	-2.872298	-0.000002
7	6	0	-2.692365	0.889256	-0.000015
8	6	0	-1.329690	0.541418	0.000013
9	6	0	-1.270632	-0.978348	0.000027
10	6	0	-0.174770	1.352176	0.000024
11	6	0	1.075278	0.731126	0.000026
12	6	0	2.173924	0.178887	0.000020
13	6	0	3.407078	-0.516964	0.000008
14	6	0	4.637893	0.182713	-0.000015
15	6	0	5.843260	-0.518867	-0.000031
16	6	0	5.846009	-1.920923	-0.000025
17	6	0	4.631742	-2.622795	-0.000002
18	6	0	3.419879	-1.933816	0.000014
19	6	0	-0.230011	2.898377	0.000005
20	6	0	-0.951067	3.405002	1.275977
21	6	0	-0.950861	3.404951	-1.276103
22	6	0	1.195351	3.494805	0.000112
23	7	0	-0.244919	-1.734540	0.000046
24	1	0	-2.572984	-3.603281	0.000039
25	1	0	-5.565194	0.422182	-0.000057
26	1	0	-6.528115	-1.873569	-0.000044
27	1	0	-5.057476	-3.864157	0.000004
28	1	0	-3.089847	1.891392	-0.000036
29	1	0	4.630931	1.267292	-0.000020
30	1	0	6.781589	0.025860	-0.000048
31	1	0	6.786211	-2.462284	-0.000039
32	1	0	4.632377	-3.707860	0.000003
33	1	0	2.475107	-2.465703	0.000034
34	1	0	-0.420022	3.067614	2.173308
35	1	0	-0.961968	4.501345	1.277475
36	1	0	-1.984064	3.056736	1.343714

37	1	0	-0.419646	3.067568	-2.173334
38	1	0	-1.983831	3.056633	-1.344011
39	1	0	-0.961811	4.501294	-1.277626
40	1	0	1.758630	3.187580	-0.887182
41	1	0	1.127318	4.588117	0.000097
42	1	0	1.758489	3.187595	0.887502

Geometry, imaginary frequency, and free energy for TS$_{S43d\ to\ S46}$

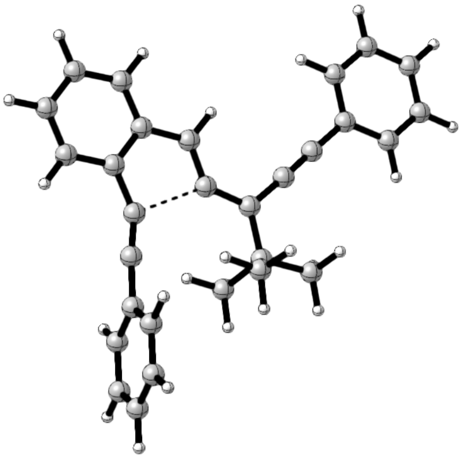

Imaginary frequency= -415.56 cm^{-1}
Sum of electronic and thermal Free Energies= -1119.417017

Center Number	Atomic Number	Atomic Type	Coordinates (Angstroms) X	Y	Z
1	6	0	4.408846	-0.948002	-1.497303
2	6	0	3.322183	-0.927993	-0.617026
3	6	0	3.117235	-1.987979	0.296016
4	6	0	4.002964	-3.069898	0.330942
5	6	0	5.092230	-3.086996	-0.550169
6	6	0	5.294220	-2.033392	-1.456314
7	6	0	2.301092	0.106483	-0.512406
8	6	0	1.929921	-1.751453	1.106104
9	6	0	1.254579	-0.612966	0.841637
10	6	0	0.072539	-0.015253	1.276555
11	6	0	0.066896	1.128206	2.314827
12	6	0	-1.152268	-0.508052	0.813073
13	6	0	-2.236335	-0.934505	0.420567
14	6	0	-3.482784	-1.412609	-0.052428
15	6	0	-4.692476	-0.800360	0.359490
16	6	0	-5.916662	-1.269878	-0.115192
17	6	0	-5.963675	-2.350850	-1.006897
18	6	0	-4.773068	-2.963741	-1.422311
19	6	0	-3.542725	-2.505219	-0.952631
20	6	0	-0.951518	2.225653	1.942672
21	6	0	1.464828	1.755983	2.470066
22	6	0	-0.350709	0.482788	3.667028
23	1	0	4.561133	-0.130933	-2.194280
24	1	0	3.845619	-3.885434	1.029693
25	1	0	5.786253	-3.921031	-0.531910
26	1	0	6.144679	-2.056889	-2.129749
27	1	0	1.627598	-2.432013	1.894968
28	1	0	-4.652969	0.037225	1.047507
29	1	0	-6.836021	-0.792767	0.208191
30	1	0	-6.918435	-2.711960	-1.374268
31	1	0	-4.805571	-3.800602	-2.112285
32	1	0	-2.620368	-2.978331	-1.271462
33	1	0	-1.953455	1.807932	1.799801
34	1	0	-1.003199	2.969494	2.746503

35	1	0	-0.656488	2.733766	1.021342
36	1	0	2.214614	1.005031	2.739161
37	1	0	1.785592	2.245463	1.546211
38	1	0	1.436765	2.510450	3.264495
39	1	0	0.360181	-0.296597	3.963725
40	1	0	-0.371536	1.248579	4.451898
41	1	0	-1.347283	0.033747	3.594786
42	6	0	1.966211	1.190489	-1.096697
43	6	0	0.980824	2.173341	-1.288370
44	6	0	-0.331443	1.776040	-1.672656
45	6	0	1.235155	3.552165	-1.054862
46	6	0	-1.341388	2.727232	-1.814785
47	1	0	-0.532421	0.726329	-1.847095
48	6	0	0.214724	4.488194	-1.196065
49	1	0	2.233408	3.861735	-0.766131
50	6	0	-1.075044	4.082479	-1.579192
51	1	0	-2.338571	2.410950	-2.102252
52	1	0	0.418362	5.537649	-1.010842
53	1	0	-1.863997	4.818752	-1.690034

Geometry, imaginary frequency, and free energy for $TS_{S43g\ to\ S47}$

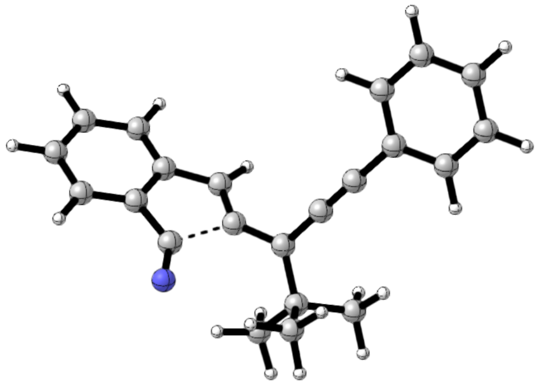

Imaginary frequency= -56.59 cm^{-1}
Sum of electronic and thermal Free Energies= -904.432426

Center Number	Atomic Number		Forces (Hartrees/Bohr) X	Y	Z
1	6	0	4.550169	-1.048684	0.915761
2	6	0	3.361379	-0.601233	0.349836
3	6	0	2.652579	-1.387399	-0.584988
4	6	0	3.153010	-2.633944	-0.982378
5	6	0	4.359519	-3.079470	-0.427789
6	6	0	5.054419	-2.294326	0.507579
7	6	0	2.661439	0.690167	0.619383
8	6	0	1.439761	-0.702000	-0.981283
9	6	0	1.241085	0.477608	-0.314402
10	6	0	0.088833	1.270259	-0.269741
11	6	0	0.095901	2.804570	-0.248765
12	6	0	-1.137956	0.609728	-0.144535
13	7	0	2.951627	1.645863	1.344303
14	6	0	-2.213125	0.025013	-0.037901
15	6	0	-3.449442	-0.653929	0.075128
16	6	0	-4.666254	0.070712	0.100220
17	6	0	-5.880333	-0.604598	0.211449
18	6	0	-5.904191	-2.003808	0.300641
19	6	0	-4.705254	-2.730612	0.277401
20	6	0	-3.484757	-2.067157	0.164888
21	6	0	1.310939	3.361134	-1.021898
22	6	0	0.150513	3.247484	1.241022
23	6	0	-1.195853	3.352582	-0.894674
24	1	0	5.083204	-0.436932	1.635311
25	1	0	2.606117	-3.249792	-1.688708
26	1	0	4.763156	-4.042140	-0.723169
27	1	0	5.998989	-2.647012	0.907861
28	1	0	0.805176	-1.043820	-1.791385
29	1	0	-4.640203	1.152551	0.032248
30	1	0	-6.808355	-0.043513	0.229651
31	1	0	-6.851654	-2.524801	0.388014
32	1	0	-4.725091	-3.812830	0.346839
33	1	0	-2.553537	-2.622249	0.145645

34	1	0	1.314527	2.997780	-2.056337
35	1	0	1.252313	4.455336	-1.045926
36	1	0	2.252867	3.080113	-0.548450
37	1	0	-0.752000	2.920880	1.768580
38	1	0	1.034268	2.838846	1.741220
39	1	0	0.206865	4.341734	1.287918
40	1	0	-2.089128	3.026212	-0.353761
41	1	0	-1.167860	4.447689	-0.877331
42	1	0	-1.285339	3.028317	-1.938074

Geometry, imaginary frequency, and free energy for $TS_{S46\ to\ 44}$

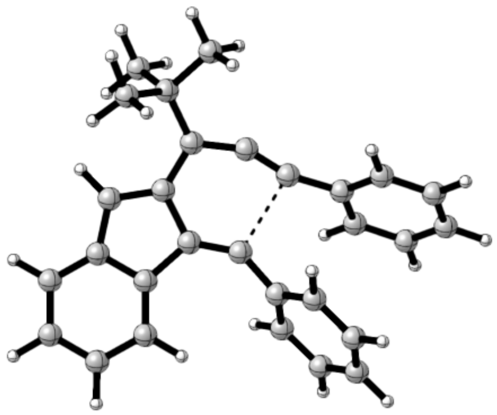

Imaginary frequency= -296.11 cm^{-1}
Sum of electronic and thermal Free Energies= -1119.438769

Center Number	Atomic Number	Atomic Type	Coordinates (Angstroms) X	Y	Z
1	6	0	-1.715068	-3.159055	0.181055
2	6	0	-2.003523	-1.809453	-0.002645
3	6	0	-3.346202	-1.374435	-0.223657
4	6	0	-4.391077	-2.310187	-0.267833
5	6	0	-4.090876	-3.666005	-0.089535
6	6	0	-2.768665	-4.087917	0.133904
7	6	0	-3.351238	0.062253	-0.373503
8	6	0	-2.051115	0.565141	-0.237796
9	6	0	-1.127073	-0.608469	-0.018440
10	6	0	-1.593683	1.909680	-0.135506
11	6	0	-0.214315	2.059113	-0.205505
12	6	0	0.962394	1.632633	-0.176946
13	6	0	2.384128	1.560884	-0.297931
14	6	0	3.206241	2.285624	0.595092
15	6	0	4.595871	2.186024	0.512032
16	6	0	5.187970	1.361011	-0.453613
17	6	0	4.381661	0.643075	-1.347019
18	6	0	2.992131	0.741727	-1.275632
19	6	0	-2.497873	3.120996	0.149050
20	6	0	-3.421317	2.852479	1.361792
21	6	0	-3.350923	3.442321	-1.106207
22	6	0	-1.626937	4.355258	0.471602
23	1	0	-0.698412	-3.493741	0.354088
24	1	0	-5.415009	-1.987413	-0.428327
25	1	0	-4.888795	-4.401159	-0.120376
26	1	0	-2.557618	-5.143573	0.270070
27	1	0	-4.236477	0.642990	-0.584602
28	1	0	2.743068	2.910871	1.350608
29	1	0	5.216838	2.745128	1.204187
30	1	0	6.268358	1.278423	-0.509533
31	1	0	4.836015	0.004544	-2.097218
32	1	0	2.367222	0.186542	-1.963574
33	1	0	-2.827394	2.629921	2.255841

34	1	0	-4.028373	3.742270	1.566700
35	1	0	-4.098485	2.012979	1.186695
36	1	0	-2.705703	3.655627	-1.966084
37	1	0	-4.013258	2.614553	-1.374887
38	1	0	-3.971772	4.326240	-0.915054
39	1	0	-0.957445	4.598356	-0.361078
40	1	0	-2.270029	5.222468	0.659165
41	1	0	-1.013951	4.185055	1.363966
42	6	0	0.187806	-0.578727	0.212785
43	6	0	1.310862	-1.440899	0.318393
44	6	0	1.660806	-2.312254	-0.750024
45	6	0	2.173157	-1.373159	1.444367
46	6	0	2.816904	-3.087727	-0.680284
47	1	0	1.017591	-2.358994	-1.621991
48	6	0	3.321953	-2.157927	1.504844
49	1	0	1.928887	-0.690721	2.250808
50	6	0	3.652754	-3.015494	0.443943
51	1	0	3.071204	-3.747436	-1.503651
52	1	0	3.969950	-2.096049	2.373005
53	1	0	4.555073	-3.615952	0.490552

Geometry, imaginary frequency, and free energy for $TS_{S47\ to\ 45}$

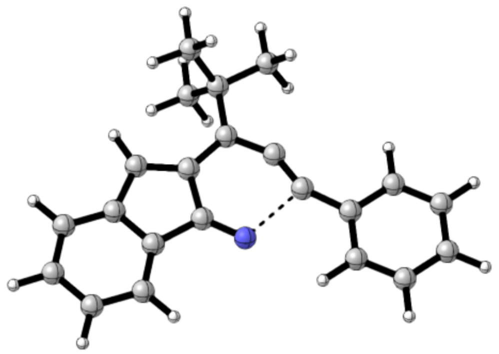

Imaginary frequency= -337.80 cm^{-1}
Sum of electronic and thermal Free Energies= -904.439814

Center Number	Atomic Number	Atomic Type	Coordinates (Angstroms) X	Y	Z
1	6	0	0.926642	-0.827709	-0.005875
2	6	0	1.280067	0.642244	0.007780
3	6	0	2.683397	0.736689	0.019655
4	6	0	3.268628	-0.587889	0.011277
5	6	0	2.227439	-1.560017	-0.004492
6	7	0	-0.225714	-1.394617	-0.018057
7	6	0	-1.791210	0.097410	-0.004043
8	6	0	-1.035155	1.108222	-0.000449
9	6	0	0.262559	1.622037	0.001445
10	6	0	-3.054011	-0.568457	-0.000730
11	6	0	0.508270	3.138405	-0.004203
12	6	0	-0.841976	3.886690	-0.033166
13	6	0	1.317211	3.544049	-1.261999
14	6	0	1.270259	3.556828	1.278704
15	6	0	-4.234235	0.218328	0.000722
16	6	0	-5.487231	-0.391747	0.005247
17	6	0	-5.593610	-1.789975	0.007971
18	6	0	-4.432064	-2.574170	0.006163
19	6	0	-3.171683	-1.976328	0.001955
20	6	0	4.607100	-1.002889	0.016191
21	6	0	4.885280	-2.377685	0.005184
22	6	0	3.850923	-3.326743	-0.010376
23	6	0	2.503207	-2.918760	-0.015348
24	1	0	3.262141	1.647612	0.034479
25	1	0	-1.450145	3.641726	0.844728
26	1	0	-0.664284	4.967817	-0.035491
27	1	0	-1.417111	3.632478	-0.930502
28	1	0	2.302403	3.071539	-1.289693
29	1	0	0.778267	3.262716	-2.173805
30	1	0	1.461122	4.630861	-1.270316
31	1	0	2.250596	3.078330	1.350617
32	1	0	1.420634	4.642836	1.278531
33	1	0	0.694790	3.290524	2.172541
34	1	0	-4.147850	1.299387	-0.001661
35	1	0	-6.381845	0.222208	0.006531

36	1	0	-6.570571	-2.261690	0.011421
37	1	0	-4.509237	-3.656525	0.008217
38	1	0	-2.270896	-2.574401	0.000561
39	1	0	5.412864	-0.276309	0.027768
40	1	0	5.917043	-2.713503	0.008604
41	1	0	4.091116	-4.384404	-0.018487
42	1	0	1.702114	-3.650078	-0.027310

Geometry and free energy for S48

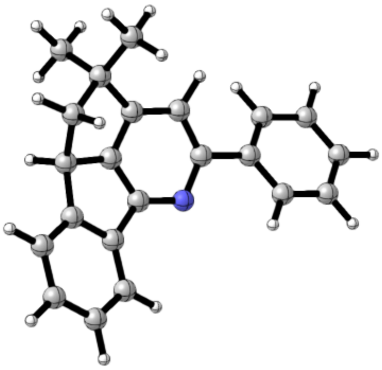

Sum of electronic and thermal Free Energies= -904.583636

Center Number	Atomic Number	Atomic Type	Coordinates (Angstroms) X	Y	Z
1	6	0	-1.972112	-2.951238	-0.054310
2	6	0	-1.826014	-1.579998	-0.254221
3	6	0	-2.951268	-0.700420	-0.155368
4	6	0	-4.211856	-1.219371	0.119606
5	6	0	-4.352840	-2.602303	0.324807
6	6	0	-3.246159	-3.457703	0.236774
7	6	0	-2.525198	0.736927	-0.482177
8	6	0	-1.047889	0.553615	-0.609612
9	6	0	-0.616392	-0.761222	-0.485970
10	7	0	0.686500	-1.055229	-0.312512
11	6	0	1.548256	-0.001380	-0.175344
12	6	0	1.099538	1.342594	-0.030405
13	6	0	-0.260197	1.614479	-0.209667
14	6	0	-2.540041	1.944363	0.519429
15	6	0	-1.202545	2.760114	0.206300
16	6	0	-0.702356	3.516045	1.444899
17	6	0	-1.427211	3.740139	-0.967908
18	6	0	2.984871	-0.344259	-0.062596
19	6	0	3.989539	0.617510	-0.275138
20	6	0	5.339242	0.277560	-0.155015
21	6	0	5.708396	-1.031134	0.173814
22	6	0	4.716043	-1.999313	0.373368
23	6	0	3.368011	-1.661280	0.253799
24	1	0	-1.110015	-3.605681	-0.120777
25	1	0	-5.076623	-0.566570	0.187668
26	1	0	-5.330415	-3.012321	0.557009
27	1	0	-3.377817	-4.522866	0.396463
28	1	0	-3.004017	1.043577	-1.424070
29	1	0	1.790655	2.101026	0.316431
30	1	0	-3.427831	2.578218	0.430775
31	1	0	-2.485574	1.562593	1.545561
32	1	0	-1.451494	4.243576	1.778567
33	1	0	-0.500680	2.825299	2.270505
34	1	0	0.221367	4.064257	1.224485
35	1	0	-2.153847	4.512028	-0.688556
36	1	0	-0.488663	4.235859	-1.240102

37	1	0	-1.801644	3.218600	-1.855282
38	1	0	3.718945	1.627618	-0.562507
39	1	0	6.101091	1.030819	-0.328118
40	1	0	6.756812	-1.295432	0.266407
41	1	0	4.994783	-3.017947	0.623575
42	1	0	2.588548	-2.398411	0.399162

Geometry and free energy for S49

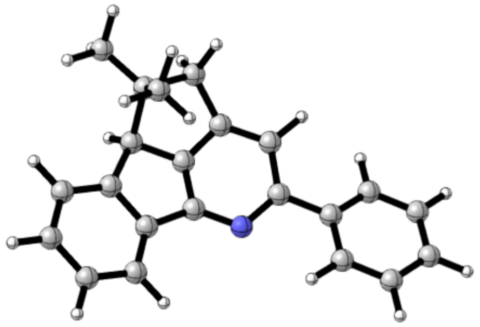

Sum of electronic and thermal Free Energies= -904.585107

Center Number	Atomic Number	Atomic Type	Coordinates (Angstroms) X	Y	Z
1	6	0	1.590384	2.891073	-0.087148
2	6	0	1.512652	1.523534	-0.342955
3	6	0	2.688779	0.707132	-0.331473
4	6	0	3.929759	1.286809	-0.088201
5	6	0	4.001959	2.665239	0.173938
6	6	0	2.845729	3.457493	0.173147
7	6	0	2.327475	-0.738113	-0.690449
8	6	0	0.843367	-0.632384	-0.774020
9	6	0	0.342996	0.647176	-0.569489
10	7	0	-0.967554	0.856986	-0.340860
11	6	0	-1.763390	-0.250855	-0.231163
12	6	0	-1.236759	-1.572962	-0.174034
13	6	0	0.128431	-1.758942	-0.414300
14	6	0	2.457850	-1.990509	0.283408
15	6	0	1.153480	-2.838349	-0.089510
16	6	0	-3.211840	0.003319	-0.052403
17	6	0	-4.169528	-1.000686	-0.285477
18	6	0	-5.530604	-0.744342	-0.102407
19	6	0	-5.958565	0.521710	0.311141
20	6	0	-5.013971	1.532094	0.532071
21	6	0	-3.654676	1.277569	0.349676
22	1	0	0.691136	3.497085	-0.089165
23	1	0	4.832470	0.683700	-0.089979
24	1	0	4.964519	3.121450	0.381212
25	1	0	2.924401	4.520638	0.375401
26	1	0	2.797367	-1.006854	-1.648982
27	1	0	-1.872928	-2.387360	0.150381
28	1	0	-3.854793	-1.976864	-0.637762
29	1	0	-6.255944	-1.528892	-0.292486
30	1	0	-7.015850	0.721052	0.452618
31	1	0	-5.338679	2.518355	0.847945
32	1	0	-2.911923	2.048667	0.510246
33	1	0	0.852412	-3.493115	0.735008
34	1	0	1.361861	-3.473663	-0.961735
35	6	0	2.363025	-1.534508	1.749718
36	1	0	2.369674	-2.401371	2.420751

37	1	0	3.209160	-0.890688	2.010404
38	1	0	1.442869	-0.968626	1.930035
39	6	0	3.745170	-2.786992	0.047061
40	1	0	3.772165	-3.683274	0.678294
41	1	0	3.828132	-3.104603	-0.999271
42	1	0	4.626496	-2.181582	0.290324

Geometry and free energy for S50 (Conformer 1)

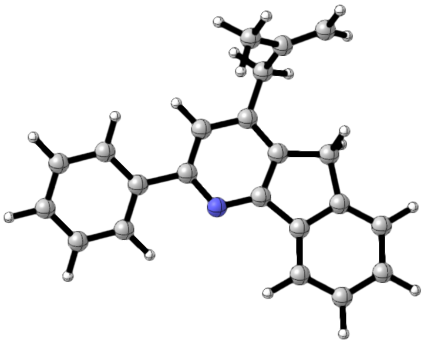

Sum of electronic and thermal Free Energies= -904.595984

Center Number	Atomic Number	Atomic Type	Coordinates (Angstroms) X	Y	Z
1	6	0	1.769799	-3.077115	0.332382
2	6	0	1.782596	-1.714358	0.034284
3	6	0	2.996856	-1.017386	-0.162845
4	6	0	4.211550	-1.689600	-0.061443
5	6	0	4.202783	-3.061042	0.237520
6	6	0	2.994096	-3.747680	0.432594
7	6	0	2.717295	0.448440	-0.466996
8	6	0	1.204073	0.510326	-0.429789
9	6	0	0.676701	-0.766767	-0.131117
10	7	0	-0.630169	-1.040909	-0.018987
11	6	0	-1.505367	-0.014479	-0.210850
12	6	0	-1.052148	1.278609	-0.531688
13	6	0	0.319072	1.566502	-0.644380
14	6	0	-2.944945	-0.343522	-0.077815
15	6	0	-3.346111	-1.692701	-0.071179
16	6	0	-4.693826	-2.034201	0.043582
17	6	0	-5.668580	-1.035791	0.159910
18	6	0	-5.280435	0.308320	0.166105
19	6	0	-3.931448	0.651526	0.050048
20	6	0	0.807712	2.966846	-0.956389
21	6	0	1.473607	3.633425	0.240708
22	6	0	2.747393	4.049152	0.190873
23	6	0	0.609978	3.794876	1.467780
24	1	0	0.827392	-3.591983	0.481405
25	1	0	5.152127	-1.167870	-0.209548
26	1	0	5.142699	-3.597308	0.319414
27	1	0	3.011422	-4.807737	0.663562
28	1	0	3.169851	1.122110	0.271742
29	1	0	3.118314	0.749945	-1.445120
30	1	0	-1.765524	2.072219	-0.717763
31	1	0	-2.579797	-2.452745	-0.154907
32	1	0	-4.985341	-3.079610	0.041701
33	1	0	-6.716792	-1.301686	0.249612
34	1	0	-6.026579	1.089694	0.268501
35	1	0	-3.651029	1.698383	0.081711
36	1	0	1.516167	2.935796	-1.792706

37	1	0	-0.047401	3.576448	-1.279238
38	1	0	3.222838	4.534926	1.037666
39	1	0	3.354845	3.924926	-0.701074
40	1	0	1.147462	4.303121	2.273043
41	1	0	-0.293150	4.376513	1.236713
42	1	0	0.269780	2.820932	1.840428

Geometry and free energy for S50 (Conformer 2)

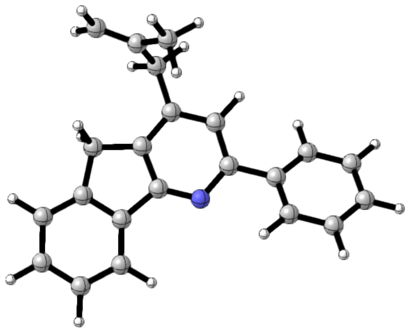

Sum of electronic and thermal Free Energies= -904.595794

Center Number	Atomic Number	Atomic Type	Coordinates (Angstroms) X	Y	Z
1	6	0	-1.758262	-3.083666	0.241124
2	6	0	-1.773666	-1.714816	-0.027503
3	6	0	-2.988847	-1.017801	-0.218496
4	6	0	-4.201983	-1.695763	-0.138837
5	6	0	-4.190669	-3.073022	0.131929
6	6	0	-2.980990	-3.759919	0.319881
7	6	0	-2.712087	0.454600	-0.491642
8	6	0	-1.199317	0.520863	-0.441651
9	6	0	-0.669835	-0.760585	-0.166188
10	7	0	0.637283	-1.034081	-0.055781
11	6	0	1.510937	-0.002238	-0.224007
12	6	0	1.054230	1.302417	-0.488094
13	6	0	-0.316894	1.588180	-0.606934
14	6	0	2.950954	-0.331876	-0.095607
15	6	0	3.962082	0.549447	-0.520537
16	6	0	5.309833	0.211839	-0.376920
17	6	0	5.672579	-1.014106	0.191218
18	6	0	4.674134	-1.902712	0.608289
19	6	0	3.327756	-1.567142	0.464335
20	6	0	-0.808399	2.999994	-0.857160
21	6	0	-1.500731	3.604847	0.357539
22	6	0	-2.771875	4.027650	0.299716
23	6	0	-0.665777	3.699070	1.611145
24	1	0	-0.815006	-3.599203	0.382262
25	1	0	-5.143263	-1.174087	-0.282598
26	1	0	-5.129338	-3.613809	0.196617
27	1	0	-2.996291	-4.824808	0.527594
28	1	0	-3.106886	0.773653	-1.466739
29	1	0	-3.172413	1.112232	0.256606
30	1	0	1.763157	2.115604	-0.585344
31	1	0	3.704149	1.494272	-0.985545
32	1	0	6.075478	0.902637	-0.715072
33	1	0	6.719821	-1.275506	0.302806
34	1	0	4.946042	-2.857627	1.046557
35	1	0	2.543899	-2.245857	0.775468
36	1	0	0.048965	3.629088	-1.133312

37	1	0	-1.500354	3.006785	-1.707807
38	1	0	-3.265493	4.471235	1.159171
39	1	0	-3.358780	3.951842	-0.611233
40	1	0	-1.219919	4.169356	2.428100
41	1	0	-0.339458	2.705510	1.942044
42	1	0	0.245671	4.285678	1.431297

Geometry and free energy for S50 (Conformer 3)

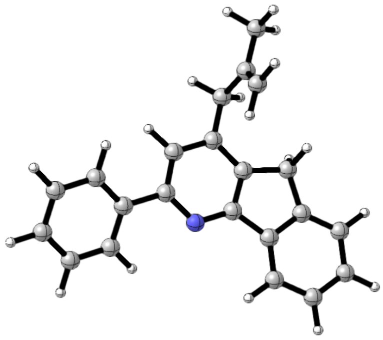

Sum of electronic and thermal Free Energies= -904.595822

Center Number	Atomic Number	Atomic Type	Coordinates (Angstroms) X	Y	Z
1	6	0	1.691696	-3.171901	0.259141
2	6	0	1.736692	-1.801791	0.001064
3	6	0	2.967818	-1.127369	-0.170250
4	6	0	4.166270	-1.829934	-0.084158
5	6	0	4.124878	-3.209070	0.174388
6	6	0	2.899994	-3.873070	0.344409
7	6	0	2.722794	0.353048	-0.434308
8	6	0	1.211817	0.447423	-0.399860
9	6	0	0.653466	-0.823775	-0.138320
10	7	0	-0.659924	-1.066445	-0.031066
11	6	0	-1.507686	-0.010255	-0.183771
12	6	0	-1.021450	1.280966	-0.460976
13	6	0	0.356520	1.534715	-0.572019
14	6	0	-2.955178	-0.305407	-0.056922
15	6	0	-3.913632	0.710159	0.115183
16	6	0	-5.271027	0.399139	0.223996
17	6	0	-5.695714	-0.932631	0.166147
18	6	0	-4.748968	-1.951656	0.005634
19	6	0	-3.392875	-1.642425	-0.101839
20	6	0	0.881649	2.921077	-0.845673
21	6	0	1.453900	3.653403	0.364484
22	6	0	1.401288	3.168581	1.612824
23	6	0	2.079204	4.989748	0.040775
24	1	0	0.737151	-3.669128	0.389661
25	1	0	5.119228	-1.325856	-0.213043
26	1	0	5.051944	-3.768871	0.244289
27	1	0	2.891975	-4.939534	0.544310
28	1	0	3.183355	0.993964	0.329064
29	1	0	3.136014	0.670800	-1.402076
30	1	0	-1.713530	2.100473	-0.611198
31	1	0	-3.604429	1.746777	0.187971
32	1	0	-5.995139	1.195725	0.361412
33	1	0	-6.750450	-1.173417	0.250308
34	1	0	-5.068850	-2.987908	-0.036287
35	1	0	-2.647873	-2.419077	-0.218965
36	1	0	1.667327	2.870236	-1.615091

37	1	0	0.082762	3.539875	-1.279490
38	1	0	1.811971	3.726179	2.448876
39	1	0	0.946113	2.211595	1.841825
40	1	0	1.351104	5.660706	-0.435846
41	1	0	2.460032	5.484004	0.938848
42	1	0	2.912042	4.874728	-0.666794

Geometry and free energy for S50 (Conformer 4)

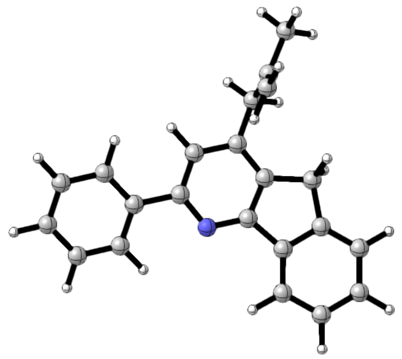

Sum of electronic and thermal Free Energies= -904.595822

Center Number	Atomic Number	Atomic Type	Coordinates (Angstroms) X	Y	Z
1	6	0	1.691696	-3.171901	0.259141
2	6	0	1.736693	-1.801791	0.001064
3	6	0	2.967818	-1.127369	-0.170250
4	6	0	4.166270	-1.829933	-0.084159
5	6	0	4.124879	-3.209069	0.174388
6	6	0	2.899995	-3.873069	0.344408
7	6	0	2.722794	0.353048	-0.434309
8	6	0	1.211817	0.447424	-0.399860
9	6	0	0.653467	-0.823775	-0.138320
10	7	0	-0.659923	-1.066446	-0.031066
11	6	0	-1.507686	-0.010256	-0.183771
12	6	0	-1.021450	1.280966	-0.460976
13	6	0	0.356520	1.534715	-0.572019
14	6	0	-2.955177	-0.305407	-0.056922
15	6	0	-3.392874	-1.642426	-0.101836
16	6	0	-4.748967	-1.951658	0.005637
17	6	0	-5.695713	-0.932633	0.166147
18	6	0	-5.271027	0.399138	0.223993
19	6	0	-3.913633	0.710158	0.115180
20	6	0	0.881648	2.921077	-0.845673
21	6	0	1.453898	3.653404	0.364485
22	6	0	1.401285	3.168582	1.612825
23	6	0	2.079202	4.989749	0.040776
24	1	0	0.737152	-3.669128	0.389660
25	1	0	5.119228	-1.325855	-0.213043
26	1	0	5.051945	-3.768870	0.244289
27	1	0	2.891976	-4.939533	0.544310
28	1	0	3.183355	0.993965	0.329064
29	1	0	3.136014	0.670801	-1.402076
30	1	0	-1.713530	2.100473	-0.611197
31	1	0	-2.647872	-2.419078	-0.218959
32	1	0	-5.068849	-2.987910	-0.036281
33	1	0	-6.750449	-1.173419	0.250308
34	1	0	-5.995140	1.195723	0.361406
35	1	0	-3.604430	1.746777	0.187964
36	1	0	1.667327	2.870236	-1.615090

37	1	0	0.082762	3.539875	-1.279491
38	1	0	1.811967	3.726181	2.448877
39	1	0	0.946110	2.211596	1.841825
40	1	0	2.460028	5.484005	0.938849
41	1	0	2.912040	4.874729	-0.666792
42	1	0	1.351102	5.660707	-0.435846

Geometry and free energy for S50 (Conformer 5)

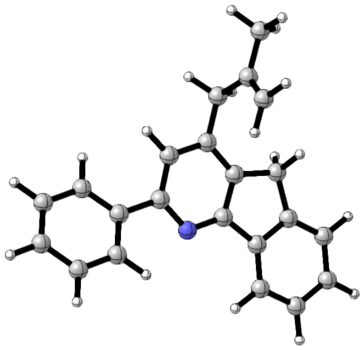

Sum of electronic and thermal Free Energies= -904.595918

Center Number	Atomic Number	Atomic Type	Coordinates (Angstroms) X	Y	Z
1	6	0	1.693452	-3.170256	0.174750
2	6	0	1.736181	-1.795684	-0.058815
3	6	0	2.965837	-1.117731	-0.226611
4	6	0	4.165171	-1.820911	-0.159964
5	6	0	4.126120	-3.204266	0.075348
6	6	0	2.902633	-3.871972	0.240858
7	6	0	2.718404	0.366625	-0.465087
8	6	0	1.207623	0.459755	-0.417980
9	6	0	0.651624	-0.816074	-0.174698
10	7	0	-0.661070	-1.061978	-0.067164
11	6	0	-1.510896	-0.004641	-0.199077
12	6	0	-1.025268	1.296941	-0.424256
13	6	0	0.351579	1.552827	-0.543858
14	6	0	-2.957691	-0.304956	-0.074391
15	6	0	-3.361014	-1.549309	0.445565
16	6	0	-4.714249	-1.858332	0.584511
17	6	0	-5.693405	-0.933326	0.202323
18	6	0	-5.304410	0.302403	-0.325800
19	6	0	-3.949746	0.613360	-0.464446
20	6	0	0.876165	2.949700	-0.759597
21	6	0	1.459913	3.627498	0.476598
22	6	0	1.416791	3.088753	1.702978
23	6	0	2.084221	4.976167	0.206413
24	1	0	0.739865	-3.670826	0.299361
25	1	0	5.117025	-1.314157	-0.286485
26	1	0	5.053896	-3.764598	0.129885
27	1	0	2.896360	-4.941905	0.421347
28	1	0	3.183868	0.995432	0.305309
29	1	0	3.124704	0.699665	-1.430642
30	1	0	-1.714437	2.130117	-0.487407
31	1	0	-2.591995	-2.256015	0.730234
32	1	0	-5.006544	-2.820900	0.991855
33	1	0	-6.746010	-1.173983	0.310163
34	1	0	-6.055053	1.022012	-0.636394
35	1	0	-3.671740	1.567451	-0.897776
36	1	0	1.654962	2.931739	-1.537448

37	1	0	0.074739	3.588138	-1.158806
38	1	0	1.834962	3.608968	2.559165
39	1	0	0.961674	2.123420	1.893801
40	1	0	1.352800	5.668154	-0.233654
41	1	0	2.473568	5.430062	1.121965
42	1	0	2.910632	4.891649	-0.512899

Geometry and free energy for S50 (Conformer 6)

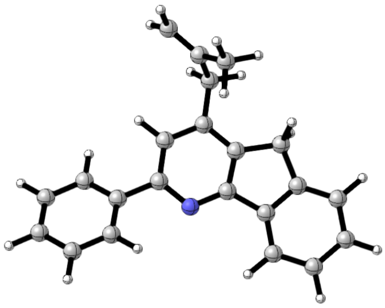

Sum of electronic and thermal Free Energies= -904.594931

Center Number	Atomic Number	Atomic Type	Coordinates (Angstroms) X	Y	Z
1	6	0	2.888057	-2.402004	0.171590
2	6	0	2.478588	-1.082165	-0.020028
3	6	0	3.418620	-0.034104	-0.145629
4	6	0	4.782057	-0.306239	-0.078612
5	6	0	5.197754	-1.632884	0.114678
6	6	0	4.259956	-2.670093	0.238348
7	6	0	2.700273	1.294589	-0.346822
8	6	0	1.239080	0.887935	-0.318031
9	6	0	1.133376	-0.510256	-0.127474
10	7	0	-0.022939	-1.180289	-0.056428
11	6	0	-1.174724	-0.458988	-0.176178
12	6	0	-1.146079	0.935101	-0.351463
13	6	0	0.070725	1.636766	-0.431490
14	6	0	-2.441448	-1.225568	-0.091277
15	6	0	-3.682260	-0.662048	-0.441689
16	6	0	-4.858455	-1.408740	-0.342679
17	6	0	-4.817051	-2.733545	0.105233
18	6	0	-3.586496	-3.306715	0.447992
19	6	0	-2.410843	-2.562274	0.348843
20	6	0	0.084514	3.142838	-0.636413
21	6	0	-0.913808	3.875701	0.241887
22	6	0	-1.943567	4.555467	-0.282760
23	6	0	-0.672594	3.776684	1.728805
24	1	0	2.150679	-3.191357	0.263912
25	1	0	5.515584	0.488627	-0.173528
26	1	0	6.257628	-1.859533	0.168879
27	1	0	4.604113	-3.688391	0.386541
28	1	0	2.945017	2.014941	0.446248
29	1	0	2.980375	1.768308	-1.297697
30	1	0	-2.068156	1.500293	-0.400467
31	1	0	-3.736296	0.355809	-0.810646
32	1	0	-5.805523	-0.957840	-0.621121
33	1	0	-5.731452	-3.312801	0.181998
34	1	0	-3.544004	-4.334751	0.793369
35	1	0	-1.451017	-2.992585	0.604476
36	1	0	1.095416	3.519426	-0.431188

37	1	0	-0.125094	3.364638	-1.691089
38	1	0	-2.658621	5.081133	0.342879
39	1	0	-2.102303	4.614601	-1.355692
40	1	0	0.316277	4.176963	1.991672
41	1	0	-1.426896	4.327570	2.297395
42	1	0	-0.689205	2.730223	2.058064

Geometry and free energy for S51

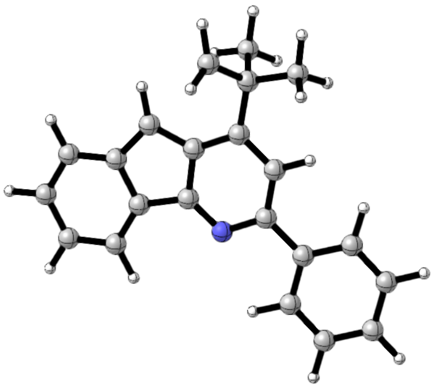

Sum of electronic and thermal Free Energies= -904.517659

Center Number	Atomic Number	Atomic Type	Coordinates (Angstroms) X	Y	Z
1	6	0	0.830866	-0.700754	0.004931
2	6	0	1.243849	0.679601	-0.017615
3	6	0	2.675197	0.714238	-0.013729
4	6	0	3.161743	-0.644136	0.004655
5	6	0	2.037165	-1.534770	0.018887
6	7	0	-0.433041	-1.112827	0.010308
7	6	0	-1.407718	-0.152509	-0.006650
8	6	0	-1.082976	1.222005	-0.047228
9	6	0	0.240701	1.675137	-0.052447
10	6	0	-2.802326	-0.640130	0.005068
11	6	0	0.612762	3.162759	-0.068641
12	6	0	-0.620968	4.074741	-0.330788
13	6	0	1.632817	3.429811	-1.149573
14	6	0	1.196578	3.543771	1.324516
15	6	0	-3.901027	0.221134	0.196812
16	6	0	-5.206038	-0.274408	0.195236
17	6	0	-5.442368	-1.640580	0.004679
18	6	0	-4.358296	-2.507953	-0.178581
19	6	0	-3.053897	-2.015532	-0.176135
20	6	0	4.462880	-1.167353	0.013650
21	6	0	4.631053	-2.558419	0.036716
22	6	0	3.522467	-3.420615	0.051572
23	6	0	2.210686	-2.909506	0.043185
24	1	0	3.291488	1.601277	-0.032111
25	1	0	-1.358850	3.983872	0.475083
26	1	0	-0.297008	5.119179	-0.372933
27	1	0	-1.103144	3.827430	-1.281672
28	1	0	1.638404	2.847048	-2.064098
29	1	0	2.227640	4.337943	-1.109129
30	1	0	2.069056	2.932719	1.569487
31	1	0	1.495852	4.598202	1.329695
32	1	0	0.442612	3.392932	2.104354
33	1	0	-3.744643	1.280112	0.366552
34	1	0	-6.038167	0.405154	0.348392
35	1	0	-6.457400	-2.024121	0.003178

36	1	0	-4.531318	-3.569436	-0.323929
37	1	0	-2.205515	-2.673719	-0.311659
38	1	0	5.325739	-0.508920	0.002637
39	1	0	5.632233	-2.976575	0.043444
40	1	0	3.679731	-4.493905	0.070160
41	1	0	1.351163	-3.570520	0.056067
42	1	0	-1.885471	1.941924	-0.096793

Geometry and free energy for S52

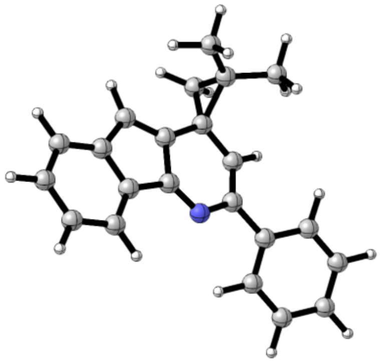

Sum of electronic and thermal Free Energies= -904.537418

Center Number	Atomic Number	Atomic Type	Coordinates (Angstroms) X	Y	Z
1	6	0	2.364342	-2.709236	-0.417733
2	6	0	2.141920	-1.376495	-0.100960
3	6	0	3.215665	-0.500915	0.227355
4	6	0	4.523750	-0.979343	0.233149
5	6	0	4.749536	-2.328421	-0.095538
6	6	0	3.687818	-3.184070	-0.417312
7	6	0	2.657117	0.830801	0.524372
8	6	0	1.297446	0.795538	0.373900
9	6	0	0.907108	-0.591737	0.011264
10	7	0	-0.312695	-1.024943	-0.100547
11	6	0	-1.371516	-0.115997	0.153034
12	6	0	-1.130920	1.183845	0.487411
13	6	0	0.215579	1.781978	0.500584
14	6	0	-2.727874	-0.702258	0.063866
15	6	0	-3.878856	0.100300	-0.054788
16	6	0	-5.148830	-0.475504	-0.114349
17	6	0	-5.295431	-1.867097	-0.067891
18	6	0	-4.157429	-2.674890	0.031313
19	6	0	-2.886179	-2.100386	0.093085
20	6	0	0.343955	3.225935	-0.222781
21	6	0	0.462647	3.096234	1.262375
22	6	0	1.567140	3.489826	-1.074414
23	6	0	-0.921332	3.831836	-0.809918
24	1	0	1.532149	-3.361330	-0.659607
25	1	0	5.356414	-0.330197	0.485047
26	1	0	5.764722	-2.712415	-0.098942
27	1	0	3.888887	-4.220331	-0.667202
28	1	0	3.258299	1.674582	0.835756
29	1	0	-1.963940	1.826140	0.740407
30	1	0	-3.783490	1.178549	-0.126701
31	1	0	-6.023162	0.160525	-0.209604
32	1	0	-6.282822	-2.314307	-0.118007
33	1	0	-4.259491	-3.755079	0.060336
34	1	0	-1.998793	-2.716343	0.160418
35	1	0	-0.350123	3.463824	1.881962
36	1	0	1.443277	3.189781	1.720078

37	1	0	1.824889	4.555928	-1.029844
38	1	0	2.438963	2.916374	-0.757219
39	1	0	1.366176	3.234843	-2.121358
40	1	0	-0.743708	4.890980	-1.035199
41	1	0	-1.199882	3.331963	-1.744575
42	1	0	-1.773434	3.781453	-0.128063

Geometry and free energy for S53

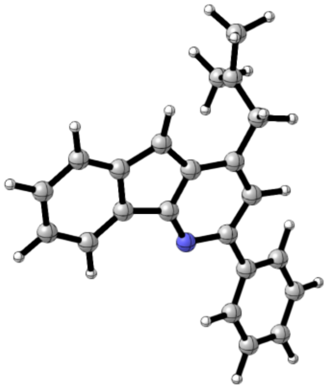

Sum of electronic and thermal Free Energies= -904.536299

Center Number	Atomic Number	Atomic Type	Coordinates (Angstroms) X	Y	Z
1	6	0	1.533776	-3.247332	0.183116
2	6	0	1.590608	-1.882336	-0.050377
3	6	0	2.847885	-1.211159	-0.230672
4	6	0	4.041283	-1.945129	-0.169367
5	6	0	3.975361	-3.324575	0.068417
6	6	0	2.740308	-3.970127	0.242590
7	6	0	2.595685	0.192143	-0.458598
8	6	0	1.182965	0.399698	-0.414529
9	6	0	0.541126	-0.863710	-0.165814
10	7	0	-0.774234	-1.031187	-0.059865
11	6	0	-1.568656	0.076937	-0.196312
12	6	0	-1.008498	1.355033	-0.422600
13	6	0	0.371394	1.543714	-0.537658
14	6	0	-3.023625	-0.147184	-0.074802
15	6	0	-3.968054	0.853519	-0.378444
16	6	0	-5.335950	0.608642	-0.246204
17	6	0	-5.790662	-0.641034	0.189871
18	6	0	-4.862093	-1.646226	0.487440
19	6	0	-3.495389	-1.404371	0.355096
20	6	0	1.948472	3.330309	0.328474
21	6	0	0.985181	2.907474	-0.759083
22	6	0	1.546799	3.162301	1.762447
23	6	0	3.014157	4.326235	-0.016905
24	1	0	0.577524	-3.741404	0.315170
25	1	0	5.000718	-1.455334	-0.302725
26	1	0	4.891678	-3.903466	0.118785
27	1	0	2.716489	-5.039409	0.424546
28	1	0	3.345850	0.950529	-0.632669
29	1	0	-1.653793	2.222121	-0.489829
30	1	0	-3.642736	1.823559	-0.735842
31	1	0	-6.047087	1.391709	-0.488698
32	1	0	-6.854279	-0.829692	0.292552
33	1	0	-5.205146	-2.619489	0.823454
34	1	0	-2.765563	-2.172151	0.576803
35	1	0	0.170337	3.649111	-0.859823

36	1	0	1.514157	2.914005	-1.723874
37	1	0	0.865220	3.968913	2.093292
38	1	0	1.020493	2.216399	1.928782
39	1	0	2.418219	3.194585	2.427070
40	1	0	2.628099	5.363490	-0.020530
41	1	0	3.834345	4.303188	0.711135
42	1	0	3.432682	4.144699	-1.015060

Geometry, imaginary frequency, and free energy for $TS_{45b\ to\ S51}$

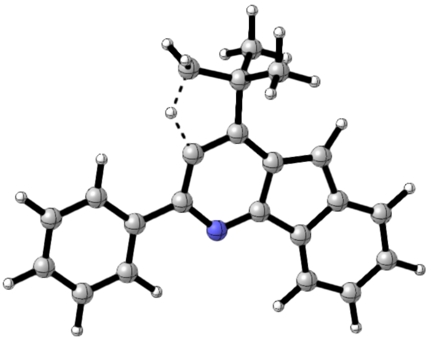

Imaginary frequency= -1819.65 cm^{-1}
Sum of electronic and thermal Free Energies= -904.479936

Center Number	Atomic Number	Atomic Type	Coordinates (Angstroms) X	Y	Z
1	6	0	-0.711626	0.887688	0.000000
2	6	0	0.668994	1.328612	0.000000
3	6	0	0.682224	2.755363	0.000000
4	6	0	-0.685471	3.221609	0.000000
5	6	0	-1.560469	2.084618	0.000000
6	7	0	-1.112123	-0.380587	0.000000
7	6	0	-0.153829	-1.372248	0.000000
8	6	0	1.196654	-0.999790	0.000000
9	6	0	1.647961	0.316078	0.000000
10	6	0	-0.624763	-2.763347	0.000000
11	6	0	3.172926	0.438362	0.000000
12	6	0	3.659840	-1.034385	0.000000
13	6	0	3.659840	1.178130	1.267944
14	6	0	3.659840	1.178130	-1.267944
15	6	0	0.285165	-3.838569	0.000000
16	6	0	-0.173118	-5.156104	0.000000
17	6	0	-1.547878	-5.423796	0.000000
18	6	0	-2.460237	-4.360838	0.000000
19	6	0	-2.005525	-3.042503	0.000000
20	6	0	-1.226344	4.514705	0.000000
21	6	0	-2.620262	4.664421	0.000000
22	6	0	-3.467068	3.544868	0.000000
23	6	0	-2.937459	2.240153	0.000000
24	1	0	1.556526	3.391208	0.000000
25	1	0	4.159795	-1.373406	-0.908754
26	1	0	4.159795	-1.373406	0.908754
27	1	0	3.266512	2.199555	1.304016
28	1	0	3.327312	0.655938	2.171359
29	1	0	4.754476	1.231060	1.279887
30	1	0	3.266512	2.199555	-1.304016
31	1	0	4.754476	1.231060	-1.279887
32	1	0	3.327312	0.655938	-2.171359
33	1	0	1.350619	-3.638118	0.000000
34	1	0	0.539711	-5.974360	0.000000
35	1	0	-1.903503	-6.449016	0.000000
36	1	0	-3.526697	-4.561883	0.000000

37	1	0	-2.696310	-2.208937	0.000000
38	1	0	-0.579859	5.386628	0.000000
39	1	0	-3.051461	5.660047	0.000000
40	1	0	-4.542579	3.687004	0.000000
41	1	0	-3.586961	1.371820	0.000000
42	1	0	2.452822	-1.593529	0.000000

Geometry, imaginary frequency, and free energy for $TS_{S51\ to\ S48}$

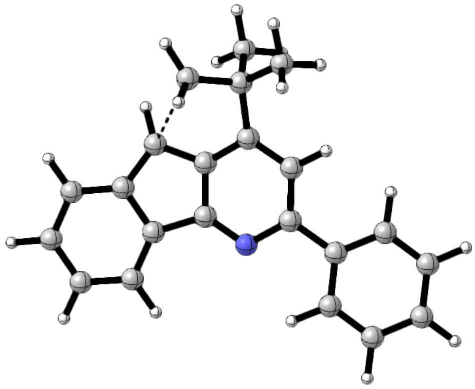

Imaginary frequency= -234.38 cm^{-1}
Sum of electronic and thermal Free Energies= -904.512983

Center Number	Atomic Number	Atomic Type	Coordinates (Angstroms) X	Y	Z
1	6	0	-0.764465	-0.727401	-0.159081
2	6	0	-1.189323	0.627648	-0.253214
3	6	0	-2.632202	0.683971	-0.275490
4	6	0	-3.103380	-0.680241	-0.112196
5	6	0	-1.968459	-1.562303	-0.067331
6	7	0	0.518216	-1.097211	-0.086774
7	6	0	1.458694	-0.106791	-0.081377
8	6	0	1.096204	1.263598	-0.041090
9	6	0	-0.244863	1.646582	-0.091211
10	6	0	2.870831	-0.543764	-0.047825
11	6	0	-0.813024	3.047664	0.156035
12	6	0	0.209540	3.968604	0.872526
13	6	0	-2.031520	2.861173	1.049910
14	6	0	-1.218452	3.698873	-1.192796
15	6	0	3.930516	0.337010	-0.337902
16	6	0	5.255861	-0.100652	-0.291325
17	6	0	5.548597	-1.427860	0.041373
18	6	0	4.501782	-2.315862	0.319636
19	6	0	3.177659	-1.880756	0.273347
20	6	0	-4.398477	-1.213790	-0.060983
21	6	0	-4.556160	-2.600554	0.054931
22	6	0	-3.440171	-3.452852	0.105968
23	6	0	-2.134895	-2.936388	0.039270
24	1	0	-3.236761	1.486798	-0.668682
25	1	0	1.081877	4.153037	0.234156
26	1	0	-0.250764	4.935831	1.099138
27	1	0	0.552919	3.519552	1.809746
28	1	0	-1.905014	2.348793	1.998668
29	1	0	-2.874190	3.542080	0.958472
30	1	0	-1.933093	3.075507	-1.738047
31	1	0	-1.673957	4.680881	-1.019731
32	1	0	-0.336722	3.834757	-1.827711
33	1	0	3.723122	1.361477	-0.626173
34	1	0	6.058742	0.591809	-0.523115
35	1	0	6.578669	-1.767242	0.077365

36	1	0	4.719573	-3.348266	0.573929
37	1	0	2.356997	-2.555752	0.480103
38	1	0	-5.266563	-0.563125	-0.098441
39	1	0	-5.553844	-3.023982	0.106443
40	1	0	-3.589461	-4.524064	0.192291
41	1	0	-1.270921	-3.591287	0.062682
42	1	0	1.866131	2.008200	0.109285

Geometry, imaginary frequency, and free energy for $TS_{S51\ to\ 52}$

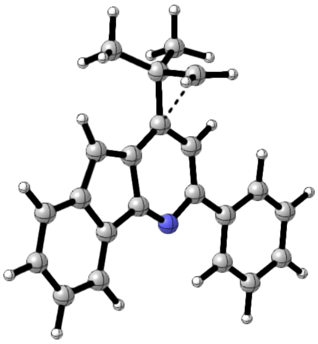

Imaginary frequency= -590.98 cm^{-1}
Sum of electronic and thermal Free Energies= -904.513084

Center Number	Atomic Number	Atomic Type	Coordinates (Angstroms) X	Y	Z
1	6	0	-0.880043	-0.656014	0.014685
2	6	0	-1.297518	0.760006	-0.005188
3	6	0	-2.695393	0.781138	-0.013022
4	6	0	-3.198627	-0.585724	-0.005590
5	6	0	-2.088633	-1.480392	0.012776
6	7	0	0.357751	-1.087673	0.024208
7	6	0	1.377991	-0.142214	0.017467
8	6	0	1.094725	1.214152	0.009396
9	6	0	-0.238062	1.739961	0.002073
10	6	0	2.752520	-0.687064	0.000514
11	6	0	-0.451466	3.264136	-0.018354
12	6	0	0.721976	4.073803	-0.594279
13	6	0	-0.450664	3.171755	1.474373
14	6	0	-1.761453	3.752389	-0.655272
15	6	0	3.890848	0.128353	0.159178
16	6	0	5.173831	-0.419895	0.131040
17	6	0	5.350505	-1.796369	-0.052938
18	6	0	4.227748	-2.618059	-0.203756
19	6	0	2.944058	-2.072042	-0.175600
20	6	0	-4.500941	-1.094462	-0.014991
21	6	0	-4.680249	-2.486351	-0.007403
22	6	0	-3.581234	-3.359297	0.008750
23	6	0	-2.268534	-2.856856	0.017848
24	1	0	-3.340482	1.644699	-0.005071
25	1	0	0.857210	3.852672	-1.658455
26	1	0	0.507741	5.141852	-0.485502
27	1	0	1.665612	3.879450	-0.078559
28	1	0	0.469903	3.288108	2.034121
29	1	0	-1.353753	2.900950	2.008025
30	1	0	-2.638711	3.455040	-0.080484
31	1	0	-1.756871	4.846351	-0.696025
32	1	0	-1.865143	3.369111	-1.675321
33	1	0	3.783664	1.195285	0.319813
34	1	0	6.036262	0.226716	0.257898
35	1	0	6.348697	-2.221260	-0.074252
36	1	0	4.352411	-3.687125	-0.343628

37	1	0	2.068042	-2.697671	-0.285597
38	1	0	-5.359477	-0.430262	-0.029071
39	1	0	-5.685733	-2.894842	-0.014173
40	1	0	-3.747687	-4.431270	0.015831
41	1	0	-1.411504	-3.521263	0.031313
42	1	0	1.917269	1.911446	-0.020557

Geometry, imaginary frequency, and free energy for TS$_{S52\ to\ S53}$

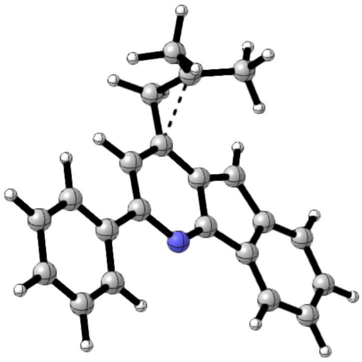

Imaginary frequency= -238.49 cm^{-1}
Sum of electronic and thermal Free Energies= -904.534183

Center Number	Atomic Number	Atomic Type	Coordinates (Angstroms) X	Y	Z
1	6	0	2.351145	-2.733494	0.317493
2	6	0	2.123700	-1.397167	-0.003582
3	6	0	3.206971	-0.507065	-0.298397
4	6	0	4.521675	-0.987353	-0.259709
5	6	0	4.743247	-2.333057	0.070674
6	6	0	3.674097	-3.200381	0.357180
7	6	0	2.663065	0.815305	-0.607709
8	6	0	1.282063	0.753088	-0.517632
9	6	0	0.893658	-0.621658	-0.144042
10	7	0	-0.343115	-1.037953	-0.006432
11	6	0	-1.381901	-0.132902	-0.234574
12	6	0	-1.119458	1.191710	-0.538084
13	6	0	0.213394	1.704601	-0.647430
14	6	0	-2.751664	-0.681470	-0.115461
15	6	0	-3.878542	0.009558	-0.607182
16	6	0	-5.162692	-0.522797	-0.468239
17	6	0	-5.353013	-1.761692	0.157295
18	6	0	-4.240913	-2.463513	0.638875
19	6	0	-2.956215	-1.931904	0.503032
20	6	0	0.424966	3.326020	0.411170
21	6	0	0.471581	3.150565	-1.056108
22	6	0	-0.832820	3.783487	1.091909
23	6	0	1.679628	3.333477	1.218279
24	1	0	1.519954	-3.397609	0.533427
25	1	0	5.359076	-0.332612	-0.481037
26	1	0	5.760137	-2.712197	0.105230
27	1	0	3.874399	-4.236375	0.610130
28	1	0	3.259604	1.671712	-0.890715
29	1	0	-1.942482	1.879790	-0.679510
30	1	0	-3.755471	0.958026	-1.118290
31	1	0	-6.014463	0.026027	-0.857776
32	1	0	-6.350963	-2.174930	0.261978
33	1	0	-4.373873	-3.426205	1.122837
34	1	0	-2.095190	-2.473836	0.873928
35	1	0	-0.338631	3.608778	-1.620581

36	1	0	1.439926	3.299537	-1.532799
37	1	0	-0.671369	4.811648	1.450388
38	1	0	-1.699142	3.794708	0.428340
39	1	0	-1.064183	3.173476	1.972621
40	1	0	2.284960	4.214465	0.957245
41	1	0	1.464097	3.363944	2.289952
42	1	0	2.313590	2.457174	1.013105

Geometry, imaginary frequency, and free energy for $TS_{S53\ to\ S49}$

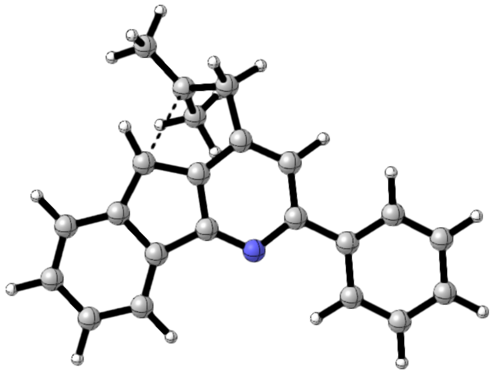

Imaginary frequency= -156.93 cm^{-1}

Sum of electronic and thermal Free Energies= -904.529682

Center Number	Atomic Number	Atomic Type	Coordinates (Angstroms) X	Y	Z
1	6	0	1.550306	-3.078764	0.160558
2	6	0	1.547155	-1.723793	-0.145056
3	6	0	2.779319	-1.007419	-0.350387
4	6	0	4.000064	-1.690831	-0.255166
5	6	0	3.991946	-3.055182	0.058684
6	6	0	2.783535	-3.742739	0.266143
7	6	0	2.473642	0.372397	-0.683602
8	6	0	1.041370	0.496365	-0.627615
9	6	0	0.452697	-0.762892	-0.320250
10	7	0	-0.861872	-0.937674	-0.144270
11	6	0	-1.665764	0.163374	-0.236847
12	6	0	-1.127088	1.465526	-0.403538
13	6	0	0.245320	1.643199	-0.573947
14	6	0	-3.117029	-0.068941	-0.076869
15	6	0	-4.071015	0.900328	-0.441899
16	6	0	-5.436019	0.658281	-0.272572
17	6	0	-5.875399	-0.558793	0.259835
18	6	0	-4.935802	-1.535234	0.614394
19	6	0	-3.572387	-1.295261	0.446340
20	6	0	2.194349	2.779371	0.440029
21	6	0	1.012727	2.944751	-0.512093
22	6	0	1.888554	2.441740	1.867386
23	6	0	3.464516	3.511075	0.132198
24	1	0	0.614859	-3.607741	0.306363
25	1	0	4.938977	-1.168908	-0.412309
26	1	0	4.931762	-3.591013	0.143737
27	1	0	2.804957	-4.800845	0.505441
28	1	0	3.155910	1.043709	-1.183437
29	1	0	-1.773982	2.330455	-0.318212
30	1	0	-3.751757	1.837541	-0.883955
31	1	0	-6.156236	1.415816	-0.564762
32	1	0	-6.936171	-0.746409	0.390758
33	1	0	-5.267942	-2.484136	1.023447
34	1	0	-2.833739	-2.041432	0.710059
35	1	0	0.357254	3.768111	-0.182212

36	1	0	1.388352	3.221724	-1.507472
37	1	0	1.524356	3.330983	2.414040
38	1	0	1.105901	1.679444	1.938816
39	1	0	2.778393	2.080862	2.392960
40	1	0	3.380684	4.584456	0.382824
41	1	0	4.304480	3.115286	0.713390
42	1	0	3.717060	3.456471	-0.933922

Geometry, imaginary frequency, and free energy for $TS_{S53\ to\ S50}$

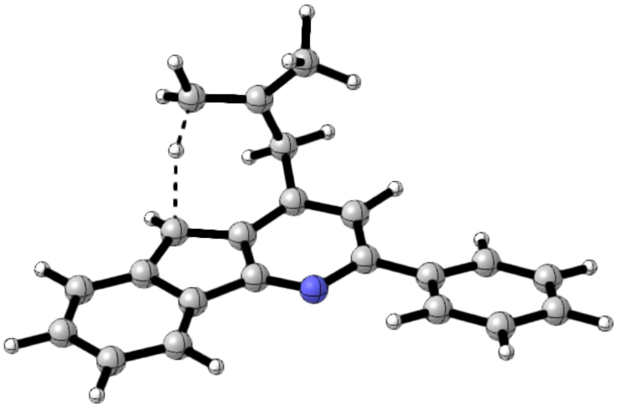

Imaginary frequency= -381.80 cm^{-1}
Sum of electronic and thermal Free Energies= -904.530622

Center Number	Atomic Number	Atomic Type	Coordinates (Angstroms) X	Y	Z
1	6	0	-2.054989	-2.828589	0.325878
2	6	0	-1.923429	-1.497066	-0.056340
3	6	0	-3.066739	-0.692845	-0.351199
4	6	0	-4.344949	-1.251392	-0.261209
5	6	0	-4.472192	-2.592749	0.127457
6	6	0	-3.342995	-3.374842	0.421236
7	6	0	-2.619852	0.658831	-0.715190
8	6	0	-1.194321	0.647317	-0.697121
9	6	0	-0.741752	-0.661559	-0.262372
10	7	0	0.525320	-0.990498	-0.069483
11	6	0	1.484313	-0.022072	-0.277074
12	6	0	1.125749	1.294003	-0.578711
13	6	0	-0.222669	1.668148	-0.790752
14	6	0	2.890152	-0.455156	-0.124501
15	6	0	3.965581	0.315621	-0.605934
16	6	0	5.284116	-0.109420	-0.430344
17	6	0	5.554778	-1.316916	0.223168
18	6	0	4.492221	-2.098703	0.692512
19	6	0	3.174267	-1.675307	0.518872
20	6	0	-1.074850	3.301011	0.529467
21	6	0	-0.605574	3.135843	-0.892345
22	6	0	-2.449127	3.023576	0.817619
23	6	0	-0.077684	3.519536	1.628249
24	1	0	-1.173562	-3.423827	0.538640
25	1	0	-5.227232	-0.659408	-0.484633
26	1	0	-5.460877	-3.034702	0.202314
27	1	0	-3.470033	-4.409836	0.720801
28	1	0	-3.232648	1.343074	-1.290577
29	1	0	1.892422	2.057400	-0.645134
30	1	0	3.775595	1.237894	-1.144028
31	1	0	6.099247	0.496320	-0.813355
32	1	0	6.579545	-1.647574	0.357519
33	1	0	4.691880	-3.039891	1.194893
34	1	0	2.342452	-2.272022	0.870826
35	1	0	0.259406	3.757405	-1.138559

36	1	0	-1.402009	3.318229	-1.620360
37	1	0	-3.163338	3.503673	0.137576
38	1	0	-2.656995	1.866035	0.507229
39	1	0	-2.741479	3.143623	1.862161
40	1	0	-0.128945	4.563173	1.977350
41	1	0	-0.289105	2.885513	2.497692
42	1	0	0.948116	3.324775	1.307826

Geometry and free energy for S54

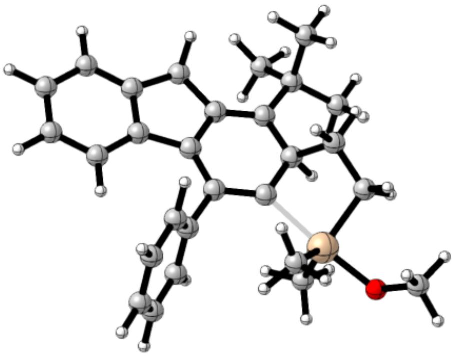

Sum of electronic and thermal Free Energies= -1452.154843

Center Number	Atomic Number	Atomic Type	Coordinates (Angstroms) X	Y	Z
1	6	0	-3.068548	-1.556583	1.456480
2	6	0	-2.713838	-0.332470	0.864150
3	6	0	-3.515301	0.816121	1.073464
4	6	0	-4.669882	0.747846	1.849261
5	6	0	-5.020976	-0.476796	2.435937
6	6	0	-4.221689	-1.615932	2.243494
7	6	0	-2.924200	2.007017	0.347732
8	6	0	-1.571865	1.480720	-0.144723
9	6	0	-1.571497	0.034778	0.024758
10	6	0	-0.573758	-0.781615	-0.576885
11	6	0	0.665605	-0.162754	-0.661867
12	6	0	0.802010	1.330496	-0.667240
13	6	0	-0.479288	2.147323	-0.637984
14	6	0	-0.489572	3.608989	-1.151454
15	6	0	0.793882	3.953058	-1.953930
16	6	0	-0.579277	4.599663	0.047653
17	6	0	-1.690082	3.859783	-2.106350
18	6	0	-0.774888	-2.203579	-0.957112
19	6	0	0.269641	-3.140291	-0.836375
20	6	0	0.094760	-4.463204	-1.252493
21	6	0	-1.125109	-4.876364	-1.801770
22	6	0	-2.168823	-3.952024	-1.936613
23	6	0	-1.994524	-2.629080	-1.521426
24	6	0	1.772761	1.737592	0.510398
25	6	0	3.243302	1.326940	0.299416
26	14	0	3.773918	-0.523080	0.450607
27	8	0	5.474328	-0.457113	0.918516
28	1	0	-2.456849	-2.438637	1.311985
29	1	0	-5.287306	1.627372	2.006900
30	1	0	-5.914902	-0.544240	3.047882
31	1	0	-4.502191	-2.553247	2.713082
32	1	0	-3.569863	2.301116	-0.490901
33	1	0	-2.832865	2.883282	0.998213
34	1	0	1.358240	1.566030	-1.590213
35	1	0	0.885469	3.331929	-2.852614

36	1	0	0.741167	4.997711	-2.280530
37	1	0	1.705776	3.840372	-1.360021
38	1	0	-1.488008	4.446972	0.636660
39	1	0	0.279946	4.493608	0.717699
40	1	0	-0.592464	5.630592	-0.327099
41	1	0	-2.652169	3.788703	-1.593865
42	1	0	-1.619586	4.867570	-2.533581
43	1	0	-1.687771	3.139601	-2.932204
44	1	0	1.211703	-2.815649	-0.412236
45	1	0	0.910135	-5.172228	-1.145554
46	1	0	-1.260106	-5.903971	-2.124672
47	1	0	-3.114475	-4.259126	-2.373059
48	1	0	-2.801987	-1.914324	-1.644284
49	1	0	1.723684	2.827302	0.623464
50	1	0	1.373825	1.312297	1.439872
51	1	0	3.595657	1.690481	-0.677101
52	1	0	3.848097	1.862059	1.047574
53	6	0	2.838767	-1.432813	1.849158
54	1	0	2.942885	-0.905357	2.804916
55	1	0	1.774755	-1.508772	1.612007
56	1	0	3.243856	-2.442918	1.977777
57	6	0	3.909893	-1.468721	-1.189398
58	1	0	2.941333	-1.507345	-1.691927
59	1	0	4.637461	-0.966411	-1.835985
60	1	0	4.269829	-2.487415	-1.009664
61	6	0	5.980434	0.008624	2.182129
62	1	0	5.612523	-0.598536	3.022499
63	1	0	7.070896	-0.069123	2.151161
64	1	0	5.716182	1.059998	2.376716

Geometry, imaginary frequency, and free energy for $TS_{S54\text{-}lose\text{-}C2H4}$

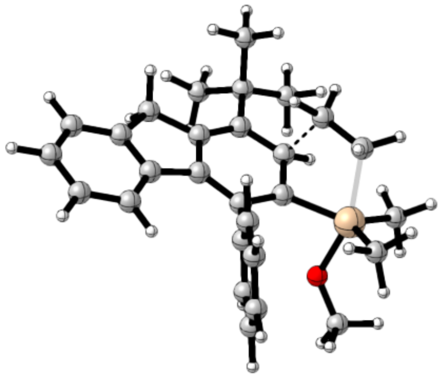

Imaginary frequency= -338.73 cm^{-1}
Sum of electronic and thermal Free Energies= -1452.143231

Center Number	Atomic Number	Atomic Type	Coordinates (Angstroms) X	Y	Z
1	6	0	-1.970210	3.033060	-0.237735
2	6	0	-2.165927	1.648709	-0.075203
3	6	0	-3.483412	1.133710	-0.000496
4	6	0	-4.591740	1.972460	-0.084548
5	6	0	-4.387675	3.348627	-0.251762
6	6	0	-3.086399	3.869272	-0.327312
7	6	0	-3.452919	-0.362048	0.196952
8	6	0	-1.966250	-0.697973	0.135321
9	6	0	-1.215256	0.531136	0.037654
10	6	0	0.205819	0.560189	0.051022
11	6	0	0.866081	-0.661187	-0.039764
12	6	0	0.107762	-1.910788	-0.095140
13	6	0	-1.331665	-1.930412	0.162906
14	6	0	-2.051327	-3.284785	0.388293
15	6	0	-1.049970	-4.435068	0.677914
16	6	0	-2.853971	-3.662956	-0.892278
17	6	0	-3.019076	-3.230410	1.603738
18	6	0	0.949380	1.860646	0.076022
19	6	0	1.392358	2.455337	-1.116976
20	6	0	2.055463	3.689122	-1.096077
21	6	0	2.281458	4.342185	0.120107
22	6	0	1.849339	3.751303	1.315085
23	6	0	1.190131	2.519389	1.293179
24	6	0	0.499739	-2.310751	-1.791759
25	6	0	1.948261	-2.087513	-2.036463
26	14	0	2.807019	-1.114633	-0.338611
27	1	0	-0.977083	3.454124	-0.297124
28	1	0	-5.598555	1.570065	-0.023629
29	1	0	-5.240616	4.016175	-0.322698
30	1	0	-2.942318	4.936985	-0.456393
31	1	0	-3.889117	-0.623973	1.168639
32	1	0	-4.040545	-0.890933	-0.563239
33	1	0	0.641263	-2.736413	0.375144
34	1	0	-0.471427	-4.246405	1.589531
35	1	0	-1.610639	-5.364005	0.828255
36	1	0	-0.352445	-4.607400	-0.147159

37	1	0	-3.612815	-2.913050	-1.134122
38	1	0	-2.193338	-3.768180	-1.759219
39	1	0	-3.364954	-4.620987	-0.737743
40	1	0	-3.903576	-2.620436	1.412130
41	1	0	-3.369372	-4.243561	1.833077
42	1	0	-2.512396	-2.838571	2.492703
43	1	0	1.211669	1.953783	-2.063095
44	1	0	2.393016	4.135934	-2.026259
45	1	0	2.795320	5.298309	0.138826
46	1	0	2.033994	4.246691	2.263347
47	1	0	0.878991	2.048475	2.218626
48	1	0	0.157192	-3.345417	-1.854703
49	1	0	-0.182441	-1.656202	-2.339008
50	1	0	2.501734	-3.009102	-2.234604
51	1	0	2.118527	-1.364916	-2.841707
52	6	0	4.121725	-0.134462	-1.351654
53	1	0	4.555489	-0.766630	-2.134526
54	1	0	3.660577	0.738702	-1.826030
55	1	0	4.922287	0.228833	-0.698948
56	6	0	3.510183	-2.770971	0.395716
57	1	0	4.011598	-3.342153	-0.393562
58	1	0	4.224344	-2.587999	1.204780
59	1	0	2.711025	-3.404105	0.804399
60	8	0	2.897415	-0.113520	1.229409
61	6	0	3.994509	-0.072578	2.144857
62	1	0	4.973401	-0.037204	1.635810
63	1	0	3.906588	0.833501	2.758910
64	1	0	4.008564	-0.940370	2.825298

Geometry, imaginary frequency, and free energy for $TS_{S54\text{-}migration}$

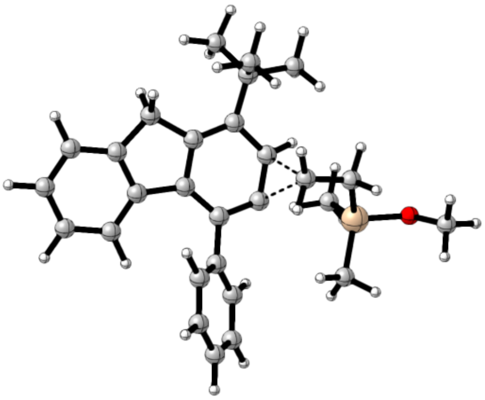

Imaginary frequency= -447.94 cm^{-1}
Sum of electronic and thermal Free Energies= -1452.136442

Center Number	Atomic Number	Atomic Type	Coordinates (Angstroms) X	Y	Z
1	6	0	3.858230	1.578696	-0.527368
2	6	0	3.235041	0.344879	-0.277243
3	6	0	4.023082	-0.818744	-0.129985
4	6	0	5.413594	-0.759400	-0.199712
5	6	0	6.030377	0.477517	-0.432197
6	6	0	5.253567	1.633860	-0.602923
7	6	0	3.143264	-2.035951	0.051242
8	6	0	1.723732	-1.479547	-0.058343
9	6	0	1.810154	-0.021617	-0.173936
10	6	0	0.661437	0.768204	-0.159949
11	6	0	-0.574986	0.122623	-0.504275
12	6	0	-0.654682	-1.294594	-0.339309
13	6	0	0.520403	-2.145798	-0.055826
14	6	0	0.332729	-3.650636	0.263854
15	6	0	-1.133902	-4.107258	0.046753
16	6	0	0.689210	-3.915461	1.755148
17	6	0	1.215638	-4.552670	-0.643480
18	6	0	0.649484	2.229175	0.110260
19	6	0	1.337714	2.770955	1.215747
20	6	0	1.274767	4.138242	1.499232
21	6	0	0.522476	4.993455	0.683547
22	6	0	-0.171489	4.467985	-0.413255
23	6	0	-0.115167	3.098726	-0.692808
24	6	0	-1.659472	-0.635230	1.104118
25	6	0	-3.144033	-0.836227	0.846464
26	14	0	-4.005039	0.284623	-0.471322
27	8	0	-5.705010	-0.147413	-0.364428
28	1	0	3.272438	2.478489	-0.664482
29	1	0	6.013736	-1.657699	-0.085092
30	1	0	7.112560	0.540102	-0.489351
31	1	0	5.740660	2.584059	-0.798318
32	1	0	3.353332	-2.784163	-0.720843
33	1	0	3.317954	-2.526957	1.018326
34	1	0	-1.480933	-1.796032	-0.836421
35	1	0	-1.442586	-4.004968	-0.999796
36	1	0	-1.222421	-5.165880	0.315256

37	1	0	-1.839161	-3.550369	0.671819
38	1	0	1.725457	-3.644737	1.978880
39	1	0	0.038606	-3.339436	2.423913
40	1	0	0.558506	-4.978696	1.992471
41	1	0	2.279058	-4.470836	-0.410329
42	1	0	0.932579	-5.602614	-0.500655
43	1	0	1.072618	-4.303702	-1.701018
44	1	0	1.917118	2.111636	1.854434
45	1	0	1.806201	4.535062	2.359017
46	1	0	0.475656	6.055689	0.902581
47	1	0	-0.755729	5.124134	-1.051317
48	1	0	-0.652240	2.682071	-1.537188
49	1	0	-1.242380	-1.345841	1.823623
50	1	0	-1.429034	0.368394	1.464189
51	1	0	-3.360117	-1.878440	0.580076
52	1	0	-3.674099	-0.656855	1.796544
53	6	0	-3.805152	2.130910	-0.036683
54	1	0	-4.185083	2.353162	0.967264
55	1	0	-2.749630	2.414113	-0.076550
56	1	0	-4.357790	2.749792	-0.752373
57	6	0	-3.594653	-0.138770	-2.273004
58	1	0	-2.533025	0.038346	-2.463492
59	1	0	-3.837996	-1.184800	-2.485849
60	1	0	-4.193740	0.487646	-2.942398
61	6	0	-6.583742	0.131512	0.742753
62	1	0	-6.650401	1.208600	0.953649
63	1	0	-7.579004	-0.230949	0.471963
64	1	0	-6.266215	-0.382751	1.662473

Geometry and free energy for S57

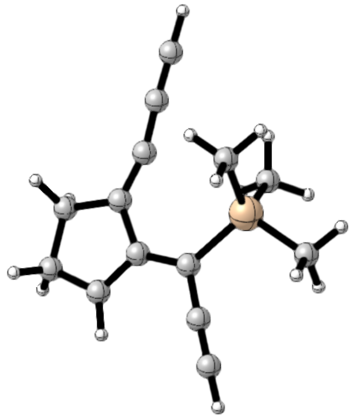

Sum of electronic and thermal Free Energies= -832.458793

Center Number	Atomic Number	Atomic Type	Coordinates (Angstroms) X	Y	Z
1	6	0	-1.755390	-0.081261	-0.011964
2	6	0	-0.752385	-1.173005	0.002893
3	6	0	-1.421135	-2.388140	0.007172
4	6	0	-2.915553	-2.249021	-0.003094
5	6	0	-3.157327	-0.715500	-0.082780
6	6	0	-1.548635	1.226490	0.033011
7	6	0	0.671499	-1.018514	0.015235
8	6	0	1.467225	-2.170048	0.062317
9	6	0	2.192497	-3.157645	0.103760
10	6	0	-1.712266	2.540273	0.070014
11	6	0	-1.792124	3.779505	0.108420
12	1	0	-0.915111	-3.345231	0.025965
13	1	0	-3.369558	-2.780531	-0.851114
14	1	0	-3.364250	-2.684580	0.901965
15	1	0	-3.788122	-0.349931	0.733543
16	1	0	-3.648337	-0.432085	-1.020488
17	1	0	2.830344	-4.008176	0.139316
18	1	0	-1.860947	4.840645	0.143893
19	14	0	1.644767	0.644189	-0.035817
20	6	0	1.424365	1.600100	1.609405
21	1	0	2.014249	2.523973	1.576781
22	1	0	0.382063	1.863398	1.797954
23	1	0	1.789512	0.989089	2.442021
24	6	0	3.494687	0.189373	-0.184105
25	1	0	3.684184	-0.392757	-1.091751
26	1	0	4.096560	1.104686	-0.227708
27	1	0	3.826726	-0.404384	0.673011
28	6	0	1.156052	1.681962	-1.577312
29	1	0	0.876839	2.699269	-1.287993
30	1	0	2.004441	1.730611	-2.268726
31	1	0	0.305605	1.239943	-2.103063

Geometry and free energy for S58

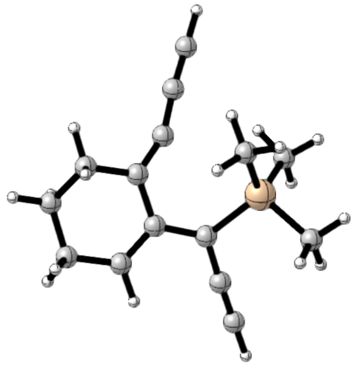

Sum of electronic and thermal Free Energies= -871.762737

Center Number	Atomic Number	Atomic Type	Coordinates (Angstroms) X	Y	Z
1	6	0	-2.790900	0.798009	0.654877
2	6	0	-1.391967	0.605104	0.072766
3	6	0	-0.914866	-0.789582	-0.048283
4	6	0	-1.845205	-1.810696	-0.000672
5	6	0	-3.323714	-1.623421	0.189299
6	6	0	-3.775258	-0.169728	-0.026598
7	6	0	0.496245	-1.071714	-0.216734
8	6	0	0.886320	-2.331666	-0.683104
9	6	0	1.270604	-3.419031	-1.101831
10	6	0	-0.690669	1.648536	-0.361156
11	6	0	-0.410155	2.923854	-0.615563
12	6	0	-0.057836	4.083421	-0.880712
13	1	0	-2.761166	0.588595	1.734993
14	1	0	-3.107011	1.837120	0.529697
15	1	0	-1.485019	-2.831591	-0.087277
16	1	0	-3.871496	-2.298309	-0.483376
17	1	0	-3.596927	-1.945182	1.209721
18	1	0	-4.788182	-0.021248	0.365241
19	1	0	-3.804929	0.052910	-1.100854
20	1	0	1.608294	-4.357841	-1.470784
21	1	0	0.239503	5.079418	-1.108320
22	14	0	1.975521	0.052361	0.312287
23	6	0	3.353041	-1.153386	0.874117
24	1	0	3.638906	-1.831219	0.064808
25	1	0	4.237621	-0.585069	1.184568
26	1	0	3.018241	-1.760499	1.722222
27	6	0	2.666084	1.056645	-1.163313
28	1	0	1.956292	1.811874	-1.507244
29	1	0	3.594346	1.558223	-0.864906
30	1	0	2.893556	0.382936	-1.996565
31	6	0	1.551572	1.162672	1.816507
32	1	0	1.186985	2.146120	1.513459
33	1	0	0.787962	0.690609	2.443412
34	1	0	2.455958	1.296005	2.421577

Geometry and free energy for S59

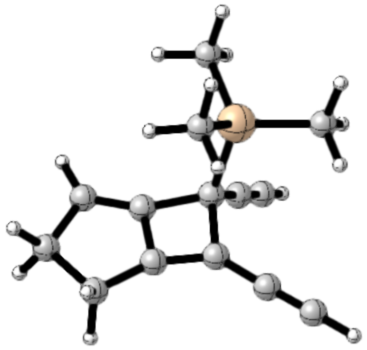

Sum of electronic and thermal Free Energies= -832.480263

Center Number	Atomic Number	Atomic Type	Coordinates (Angstroms) X	Y	Z
1	6	0	-1.734496	0.615424	-0.071857
2	6	0	-1.223091	-0.628981	0.462681
3	6	0	-2.143255	-1.608390	0.481812
4	6	0	-3.460074	-1.049578	-0.085660
5	6	0	-3.168078	0.457413	-0.488744
6	6	0	0.189580	0.006209	0.643650
7	6	0	-0.554433	1.305223	0.073232
8	6	0	0.728109	0.085581	1.984676
9	14	0	1.513973	-0.704128	-0.603232
10	6	0	0.744899	-0.706508	-2.345698
11	6	0	3.039903	0.435661	-0.525866
12	6	0	1.947515	-2.473047	-0.039286
13	6	0	1.224767	0.153902	3.093585
14	6	0	-0.057094	2.585929	-0.193981
15	6	0	0.409448	3.690417	-0.421147
16	1	0	-2.045670	-2.630903	0.825326
17	1	0	-3.791102	-1.632752	-0.954503
18	1	0	-4.265352	-1.109925	0.658019
19	1	0	-3.839008	1.150153	0.030995
20	1	0	-3.311205	0.617344	-1.563643
21	1	0	1.442840	-1.155425	-3.062843
22	1	0	-0.183607	-1.287624	-2.363846
23	1	0	0.522301	0.312882	-2.678225
24	1	0	3.827478	0.072487	-1.197009
25	1	0	2.775686	1.456308	-0.824378
26	1	0	3.444196	0.470172	0.492041
27	1	0	2.723134	-2.904999	-0.683169
28	1	0	2.319940	-2.469753	0.991418
29	1	0	1.065156	-3.122085	-0.084383
30	1	0	1.645179	0.217385	4.071968
31	1	0	0.812214	4.658993	-0.616900

Geometry and free energy for S60

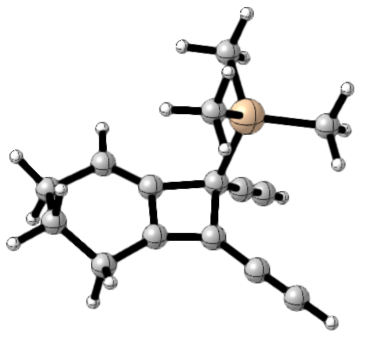

Sum of electronic and thermal Free Energies= -871.808748

Center Number	Atomic Number	Atomic Type	Coordinates (Angstroms) X	Y	Z
1	6	0	-1.429146	0.867098	0.077063
2	6	0	-1.032668	-0.463307	0.528908
3	6	0	0.448274	0.005967	0.652422
4	6	0	-0.165793	1.393853	0.188329
5	6	0	1.058874	-0.041106	1.964966
6	14	0	1.624635	-0.785819	-0.689173
7	6	0	0.789998	-0.605225	-2.392152
8	6	0	3.279004	0.160564	-0.634483
9	6	0	1.870775	-2.621483	-0.235306
10	6	0	1.614570	-0.089439	3.046799
11	6	0	-1.850133	-1.512788	0.681085
12	6	0	-3.316239	-1.297814	0.332481
13	6	0	-3.535683	-0.149035	-0.690408
14	6	0	-2.832708	1.185569	-0.307784
15	6	0	0.456288	2.625763	-0.050046
16	6	0	1.028663	3.683284	-0.255869
17	1	0	1.405461	-1.082558	-3.164380
18	1	0	-0.195644	-1.083154	-2.398287
19	1	0	0.664871	0.449604	-2.658877
20	1	0	3.984815	-0.251059	-1.365949
21	1	0	3.125158	1.221280	-0.862574
22	1	0	3.732371	0.086509	0.360410
23	1	0	2.582752	-3.097705	-0.920196
24	1	0	2.259558	-2.718195	0.784849
25	1	0	0.921380	-3.165937	-0.295515
26	1	0	2.088140	-0.125798	4.001926
27	1	0	-1.522203	-2.480157	1.050565
28	1	0	-3.739239	-2.223315	-0.077316
29	1	0	-3.884280	-1.087132	1.253019
30	1	0	-3.146370	-0.477293	-1.663363
31	1	0	-4.609874	0.026251	-0.814135
32	1	0	-3.352939	1.642854	0.547908
33	1	0	-2.890164	1.905664	-1.132083
34	1	0	1.526859	4.610200	-0.433612

Geometry and free energy for S61

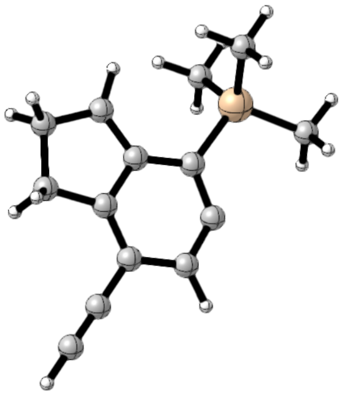

Sum of electronic and thermal Free Energies= -832.485976

Center Number	Atomic Number	Atomic Type	X	Y	Z
1	6	0	-1.728202	0.392225	-0.000000
2	6	0	-0.312997	0.633015	-0.000001
3	6	0	-2.252183	-0.904102	-0.000000
4	6	0	-1.342040	-2.009287	-0.000001
5	6	0	0.008231	-1.709164	-0.000002
6	6	0	0.611970	-0.470888	-0.000000
7	14	0	2.512759	-0.256145	0.000000
8	6	0	3.032347	0.707778	-1.566268
9	6	0	3.032351	0.707824	1.566237
10	6	0	3.311224	-1.985963	0.000029
11	6	0	-3.662913	-1.126037	0.000001
12	6	0	-4.866401	-1.305382	0.000003
13	6	0	-0.072815	2.024433	-0.000001
14	6	0	-1.367187	2.795570	0.000005
15	6	0	-2.483631	1.702417	-0.000002
16	1	0	-1.720959	-3.024851	-0.000001
17	1	0	4.117674	0.866690	-1.571954
18	1	0	2.762989	0.147875	-2.469484
19	1	0	2.541355	1.685993	-1.612436
20	1	0	4.117683	0.866703	1.571934
21	1	0	2.541390	1.686057	1.612361
22	1	0	2.762963	0.147965	2.469471
23	1	0	4.404597	-1.899462	0.000024
24	1	0	3.014042	-2.556064	0.887742
25	1	0	3.014036	-2.556099	-0.887660
26	1	0	-5.921392	-1.466474	0.000004
27	1	0	0.902167	2.494130	-0.000006
28	1	0	-1.445460	3.453818	0.877318
29	1	0	-1.445462	3.453831	-0.877298
30	1	0	-3.135379	1.785643	-0.878292
31	1	0	-3.135390	1.785642	0.878280

Geometry and free energy for S62

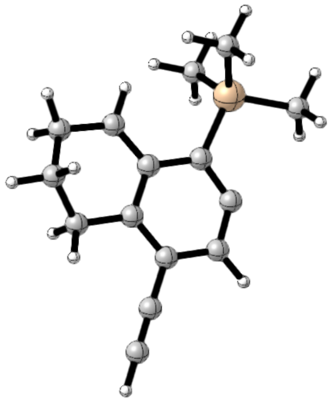

Sum of electronic and thermal Free Energies= -871.797038

Center Number	Atomic Number	Atomic Type	Coordinates (Angstroms) X	Y	Z
1	6	0	-1.665757	0.111438	-0.072070
2	6	0	-0.272795	0.483396	-0.077018
3	6	0	0.088470	1.848303	-0.159061
4	6	0	-0.914007	2.961733	-0.160236
5	6	0	-2.246420	2.517400	0.463836
6	6	0	-2.716684	1.198869	-0.168970
7	6	0	-2.043916	-1.245126	-0.008733
8	6	0	-1.053781	-2.267905	0.059529
9	6	0	0.260912	-1.846963	0.055450
10	6	0	0.735034	-0.561607	-0.007719
11	14	0	2.633814	-0.268394	0.012550
12	6	0	3.194569	0.568852	-1.612830
13	6	0	3.121823	0.797690	1.523024
14	6	0	3.472818	-1.974350	0.153816
15	6	0	-3.422234	-1.627859	-0.018748
16	6	0	-4.588571	-1.974275	-0.028074
17	1	0	1.136353	2.114461	-0.230369
18	1	0	-0.505718	3.832591	0.369432
19	1	0	-1.094525	3.301712	-1.196201
20	1	0	-2.111571	2.373338	1.544103
21	1	0	-3.009929	3.291237	0.329254
22	1	0	-2.954394	1.376552	-1.230303
23	1	0	-3.645781	0.859666	0.299452
24	1	0	-1.347483	-3.309774	0.112183
25	1	0	4.285108	0.688429	-1.612464
26	1	0	2.919169	-0.050643	-2.474463
27	1	0	2.741559	1.556837	-1.744995
28	1	0	4.211565	0.919839	1.556046
29	1	0	2.665828	1.792429	1.487519
30	1	0	2.804453	0.309673	2.452055
31	1	0	4.563234	-1.855698	0.169274
32	1	0	3.172266	-2.487859	1.074152
33	1	0	3.211279	-2.614838	-0.696155
34	1	0	-5.611572	-2.278015	-0.037401

Geometry, imaginary frequency, and free energy for TS$_{S57\text{-}4\text{-}exo}$

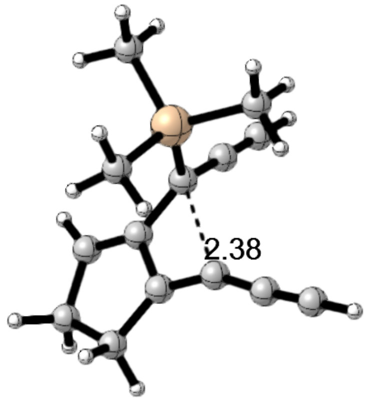

Imaginary frequency= -439.31 cm^{-1}
Sum of electronic and thermal Free Energies= -832.442199

Center Number	Atomic Number	Atomic Type	Coordinates (Angstroms) X	Y	Z
1	6	0	-1.732717	0.404583	0.011680
2	6	0	-1.106424	-0.875519	0.240143
3	6	0	-1.964094	-1.899298	0.049245
4	6	0	-3.326496	-1.367953	-0.389734
5	6	0	-3.151791	0.196473	-0.481942
6	6	0	-0.899462	1.409257	0.328184
7	6	0	0.307865	-0.620490	0.644033
8	6	0	0.758787	-0.944368	1.922424
9	6	0	1.202021	-1.209596	3.035087
10	6	0	-0.610331	2.724290	0.428246
11	6	0	-0.248987	3.897405	0.540957
12	1	0	-1.764397	-2.954148	0.192369
13	1	0	-3.636628	-1.792558	-1.353448
14	1	0	-4.107824	-1.639422	0.333506
15	1	0	-3.887467	0.729125	0.129385
16	1	0	-3.271228	0.550116	-1.512878
17	1	0	1.567800	-1.433171	4.008652
18	1	0	0.052772	4.912452	0.642957
19	14	0	1.637341	-0.266186	-0.696364
20	6	0	2.804916	1.085570	-0.030652
21	1	0	3.655682	1.234324	-0.704745
22	1	0	2.266479	2.032500	0.072924
23	1	0	3.187867	0.798729	0.954394
24	6	0	2.610568	-1.894972	-0.961287
25	1	0	1.955161	-2.679318	-1.355745
26	1	0	3.431757	-1.743593	-1.671521
27	1	0	3.032061	-2.244761	-0.012946
28	6	0	0.803116	0.247428	-2.333064
29	1	0	0.405360	1.263466	-2.267743
30	1	0	1.530386	0.206923	-3.151779
31	1	0	-0.022366	-0.430935	-2.574208

Geometry, imaginary frequency, and free energy for TS$_{S57\text{-}6\text{-}endo}$

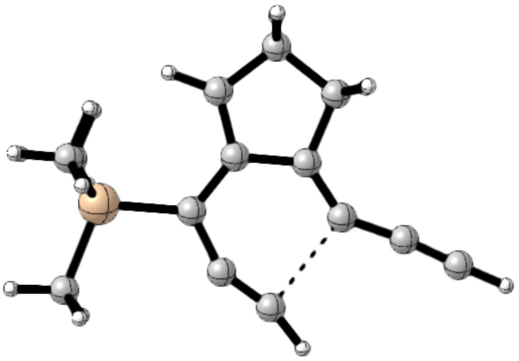

Imaginary frequency= -363.02 cm^{-1}
Sum of electronic and thermal Free Energies= -832.449321

Center Number	Atomic Number	Atomic Type	Coordinates (Angstroms) X	Y	Z
1	6	0	1.795836	0.549822	-0.000012
2	6	0	0.320238	0.675237	0.000006
3	6	0	-0.021451	2.020092	0.000031
4	6	0	1.175581	2.930210	0.000034
5	6	0	2.396561	1.963530	0.000005
6	6	0	2.523772	-0.574273	-0.000038
7	6	0	-0.574680	-0.442428	-0.000003
8	6	0	0.043808	-1.691295	-0.000032
9	6	0	1.055275	-2.419850	-0.000055
10	6	0	3.803262	-1.026445	-0.000059
11	6	0	4.918841	-1.550949	-0.000079
12	1	0	-1.036829	2.395464	0.000048
13	1	0	1.178935	3.592544	-0.877662
14	1	0	1.178954	3.592515	0.877752
15	1	0	3.032246	2.109437	0.880034
16	1	0	3.032228	2.109466	-0.880032
17	1	0	1.710490	-3.261034	-0.000079
18	1	0	5.894220	-1.975375	-0.000097
19	14	0	-2.479160	-0.294413	0.000024
20	6	0	-3.058448	0.639019	1.568356
21	1	0	-2.622201	1.641260	1.624177
22	1	0	-4.150065	0.737169	1.571222
23	1	0	-2.759960	0.089802	2.467644
24	6	0	-3.058489	0.639069	-1.568262
25	1	0	-4.150106	0.737220	-1.571097
26	1	0	-2.622242	1.641311	-1.624064
27	1	0	-2.760025	0.089880	-2.467575
28	6	0	-3.189994	-2.063268	0.000005
29	1	0	-2.856337	-2.614431	0.885501
30	1	0	-4.285383	-2.039869	0.000019
31	1	0	-2.856358	-2.614403	-0.885516

Geometry, imaginary frequency, and free energy for $TS_{S58\text{-}4\text{-}exo}$

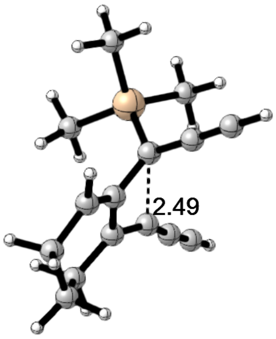

Imaginary frequency= -226.12 cm^{-1}
Sum of electronic and thermal Free Energies= -871.760227

Center Number	Atomic Number	Atomic Type	Coordinates (Angstroms) X	Y	Z
1	6	0	2.774010	0.971150	-0.528608
2	6	0	1.400983	0.681148	0.035342
3	6	0	0.968723	-0.702122	0.164207
4	6	0	1.802958	-1.749778	-0.022162
5	6	0	3.246365	-1.544032	-0.421726
6	6	0	3.757063	-0.129877	-0.069082
7	6	0	-0.468514	-0.746652	0.557162
8	6	0	-0.863885	-1.410089	1.712741
9	6	0	-1.262012	-1.980094	2.725900
10	6	0	0.481507	1.553001	0.475760
11	6	0	0.046869	2.805676	0.699414
12	6	0	-0.444773	3.915218	0.928450
13	1	0	2.717488	0.974098	-1.628433
14	1	0	3.121047	1.962600	-0.220899
15	1	0	1.441217	-2.765686	0.112462
16	1	0	3.879002	-2.296507	0.066897
17	1	0	3.358747	-1.717924	-1.505066
18	1	0	4.742180	0.036575	-0.519580
19	1	0	3.879904	-0.058464	1.019369
20	1	0	-1.590787	-2.465652	3.613486
21	1	0	-0.862417	4.872787	1.130077
22	14	0	-1.854524	-0.277845	-0.688104
23	6	0	-2.688310	-1.924919	-1.202045
24	1	0	-3.040503	-2.464493	-0.316529
25	1	0	-3.545475	-1.736975	-1.858990
26	1	0	-1.979844	-2.567990	-1.735910
27	6	0	-3.133984	0.831371	0.190020
28	1	0	-2.693010	1.802026	0.435146
29	1	0	-4.011016	0.992360	-0.446799
30	1	0	-3.463746	0.357486	1.120711
31	6	0	-1.124715	0.561716	-2.239542
32	1	0	-0.862742	1.603838	-2.040082
33	1	0	-0.222716	0.037528	-2.573560
34	1	0	-1.857706	0.533932	-3.053839

Geometry, imaginary frequency, and free energy for TS$_{S58\text{-}6\text{-}exo}$

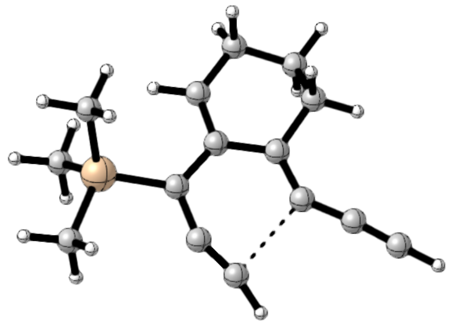

Imaginary frequency= -331.90 cm^{-1}
Sum of electronic and thermal Free Energies= -871.797038

Center Number	Atomic Number	Atomic Type	Coordinates (Angstroms) X	Y	Z
1	6	0	2.629720	1.389854	0.440300
2	6	0	1.718420	0.214511	0.065089
3	6	0	0.286311	0.550361	-0.161861
4	6	0	-0.094980	1.877437	-0.219539
5	6	0	0.834715	3.051467	-0.117744
6	6	0	2.292483	2.645297	-0.376396
7	6	0	-0.707844	-0.505574	-0.246872
8	6	0	-0.224712	-1.776583	-0.494496
9	6	0	0.635161	-2.669932	-0.583923
10	6	0	2.255757	-1.015689	0.027760
11	6	0	3.442549	-1.659387	0.173012
12	6	0	4.466152	-2.338296	0.271094
13	1	0	2.498237	1.612512	1.510805
14	1	0	3.673608	1.095995	0.300394
15	1	0	-1.144332	2.104373	-0.375784
16	1	0	0.518646	3.836151	-0.818540
17	1	0	0.753381	3.502244	0.887257
18	1	0	2.972778	3.463871	-0.114663
19	1	0	2.431659	2.431760	-1.444043
20	1	0	1.198949	-3.530039	-0.863850
21	1	0	5.360238	-2.906954	0.364730
22	14	0	-2.573719	-0.278573	0.109672
23	6	0	-3.445438	0.627797	-1.337641
24	1	0	-4.524271	0.678630	-1.149589
25	1	0	-3.073236	1.648600	-1.469947
26	1	0	-3.284962	0.083844	-2.274548
27	6	0	-2.819851	0.694431	1.738955
28	1	0	-2.352512	1.682583	1.692440
29	1	0	-3.888307	0.826043	1.944488
30	1	0	-2.373369	0.147249	2.576075
31	6	0	-3.321433	-2.025329	0.280961
32	1	0	-4.397970	-1.964754	0.475819
33	1	0	-3.167659	-2.604820	-0.635193
34	1	0	-2.850863	-2.569447	1.106556

V. References

[1] T. R. Hoye, P. R. Hanson, J. R. Vyvyan, *J. Org. Chem.* **1994**, *59*, 4096–4103.

[2] T. R. Hoye, H. Zhao, *J. Org. Chem.* **2002**, *67*, 4014–4016.

[3] Supporting Information for S. Arora, D. S. Sneddon, T. R. Hoye, *Eur. J. Org. Chem.* **2020**, *2020*, 2379–2383.

[4] S. Cacchi, G. Fabrizi, L. Moro, *J. Org. Chem.* **1997**, *62*, 5327–5332.

[5] N. V. S. D. K. Bhupathiraju, M. Sayeedi, W. Rizvi, S. Singh, J. D. Batteas, C. Michael Drain, *Tetrahedron Lett.* **2018**, *59*, 3629–3631.

[6] M. J. Frisch, G. W. Trucks, H. B. Schlegel, G. E. Scuseria, M. A. Robb, J. R. Cheeseman, G. Scalmani, V. Barone, G. A. Petersson, H. Nakatsuji, X. Li, M. Caricato, A. V. Marenich, J. Bloino, B. G. Janesko, R. Gomperts, B. Mennucci, H. P. Hratchian, J. V. Ortiz, A. F. Izmaylov, J. L. Sonnenberg, Williams, F. Ding, F. Lipparini, F. Egidi, J. Goings, B. Peng, A. Petrone, T. Henderson, D. Ranasinghe, V. G. Zakrzewski, J. Gao, N. Rega, G. Zheng, W. Liang, M. Hada, M. Ehara, K. Toyota, R. Fukuda, J. Hasegawa, M. Ishida, T. Nakajima, Y. Honda, O. Kitao, H. Nakai, T. Vreven, K. Throssell, J. A. Montgomery Jr., J. E. Peralta, F. Ogliaro, M. J. Bearpark, J. J. Heyd, E. N. Brothers, K. N. Kudin, V. N. Staroverov, T. A. Keith, R. Kobayashi, J. Normand, K. Raghavachari, A. P. Rendell, J. C. Burant, S. S. Iyengar, J. Tomasi, M. Cossi, J. M. Millam, M. Klene, C. Adamo, R. Cammi, J. W. Ochterski, R. L. Martin, K. Morokuma, O. Farkas, J. B. Foresman, D. J. Fox, Wallingford, CT, **2016**.

[7] P. J. Stephens, F. J. Devlin, C. F. Chabalowski, M. J. Frisch, *J. Phys. Chem.* **1994**, *98*, 11623–11627.

[8] A. V. Marenich, C. J. Cramer, D. G. Truhlar, *J. Phys. Chem. B* **2009**, *113*, 6378–6396.

[9] W. T. Borden, *J. Phys. Chem. A* **2017**, *121*, 1140–1144.

[10] F. L. Hirshfeld, *Theor. Chim. Acta* **1977**, *44*, 129–138.

[11] F. M. Bickelhaupt, K. N. Houk, *Angew. Chem. Int. Ed.* **2017**, *56*, 10070–10086.

[12] a) I. Fernández, F. M. Bickelhaupt, F. P. Cossío, *Chem. Eur. J.* **2014**, *20*, 10791–10801. (b) E. Velasco-Juárez, E. M. Arpa, *Theor. Chem. Acc.* **2021**, *140*, 107.

[13] *CYLview20*; C.Y. Legault, Université de Sherbrooke, 2020 (http://www.cylview.org). [accessed April 2, 2022]

VI. Copies of NMR Spectra

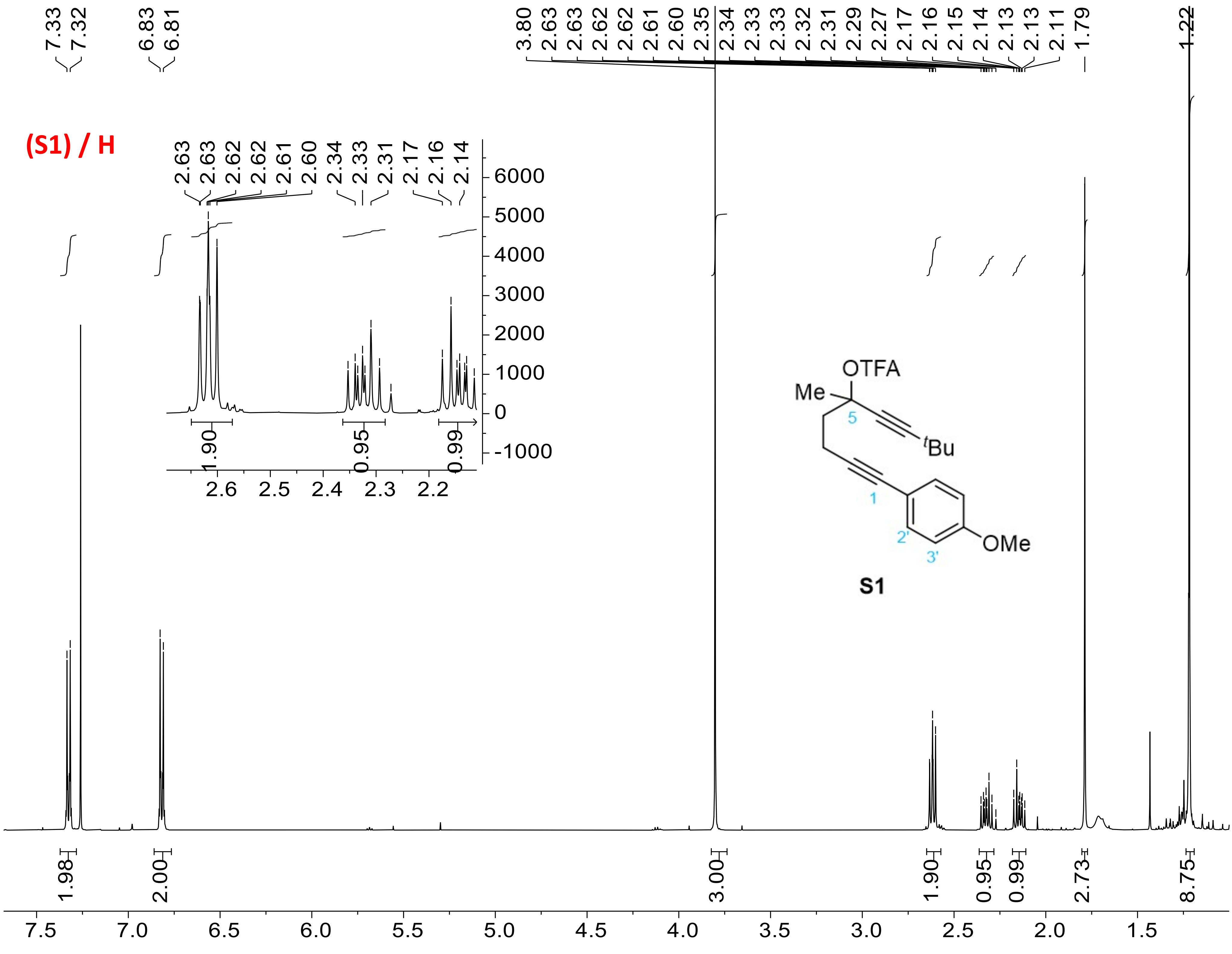
(S1) / H
7.33
7.32
6.83
6.81
3.80
2.63
2.63
2.62
2.62
2.61
2.60
2.35
2.34
2.33
2.33
2.32
2.31
2.29
2.27
2.17
2.16
2.15
2.14
2.13
2.13
2.11
1.79
1.22
1.98
2.00
3.00
1.90
0.95
0.99
2.73
8.75
OTFA
Me
5
tBu
1
2'
3'
OMe
S1

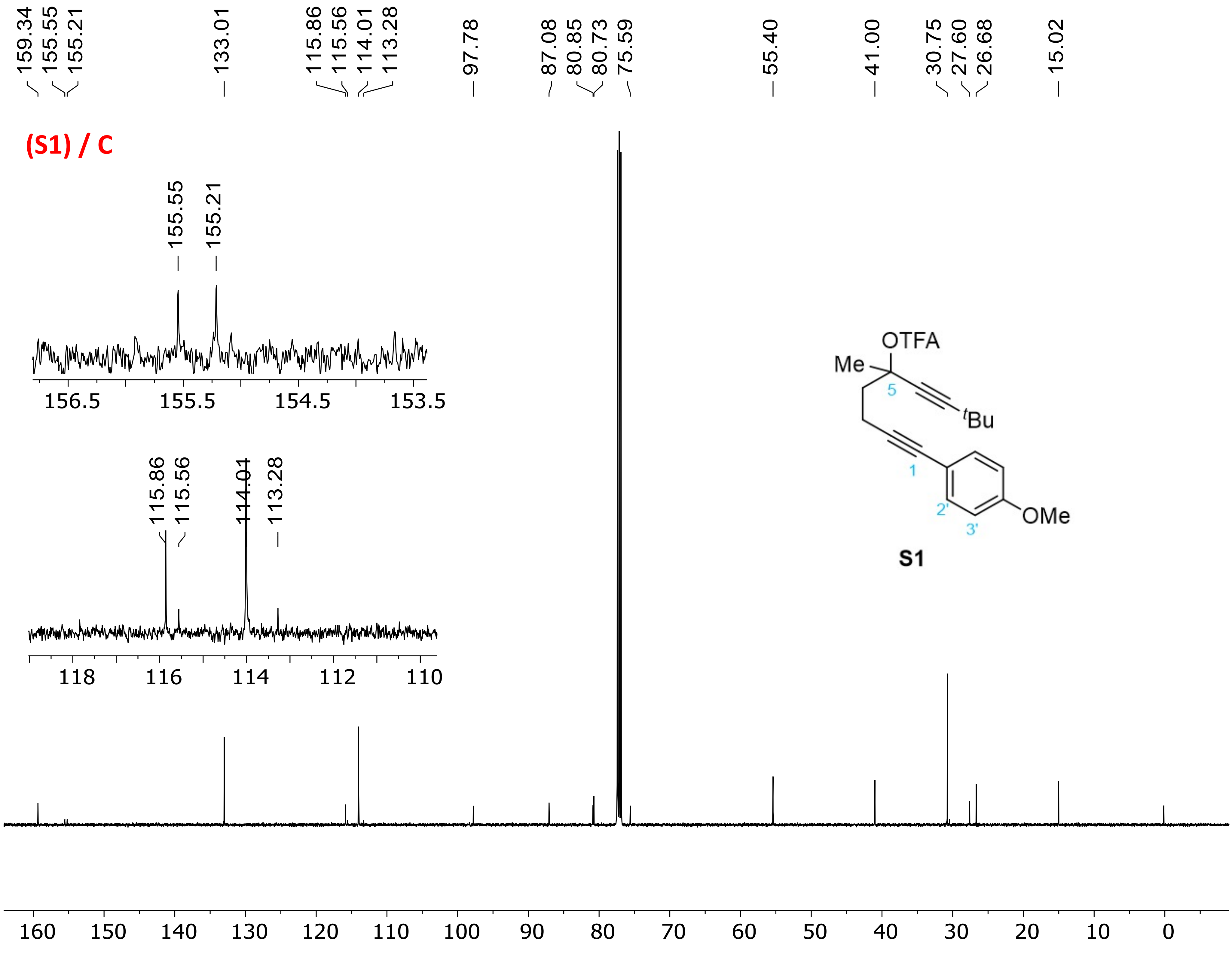
159.34
155.55
155.21
133.01
115.86
115.56
114.01
113.28
97.78
87.08
80.85
80.73
75.59
55.40
41.00
30.75
27.60
26.68
15.02
(S1) / C
155.55
155.21
156.5
155.5
154.5
153.5
115.86
115.56
114.01
113.28
118
116
114
112
110
OTFA
Me
5
^{t}Bu
1
2'
3'
OMe
S1
160
150
140
130
120
110
100
90
80
70
60
50
40
30
20
10
0

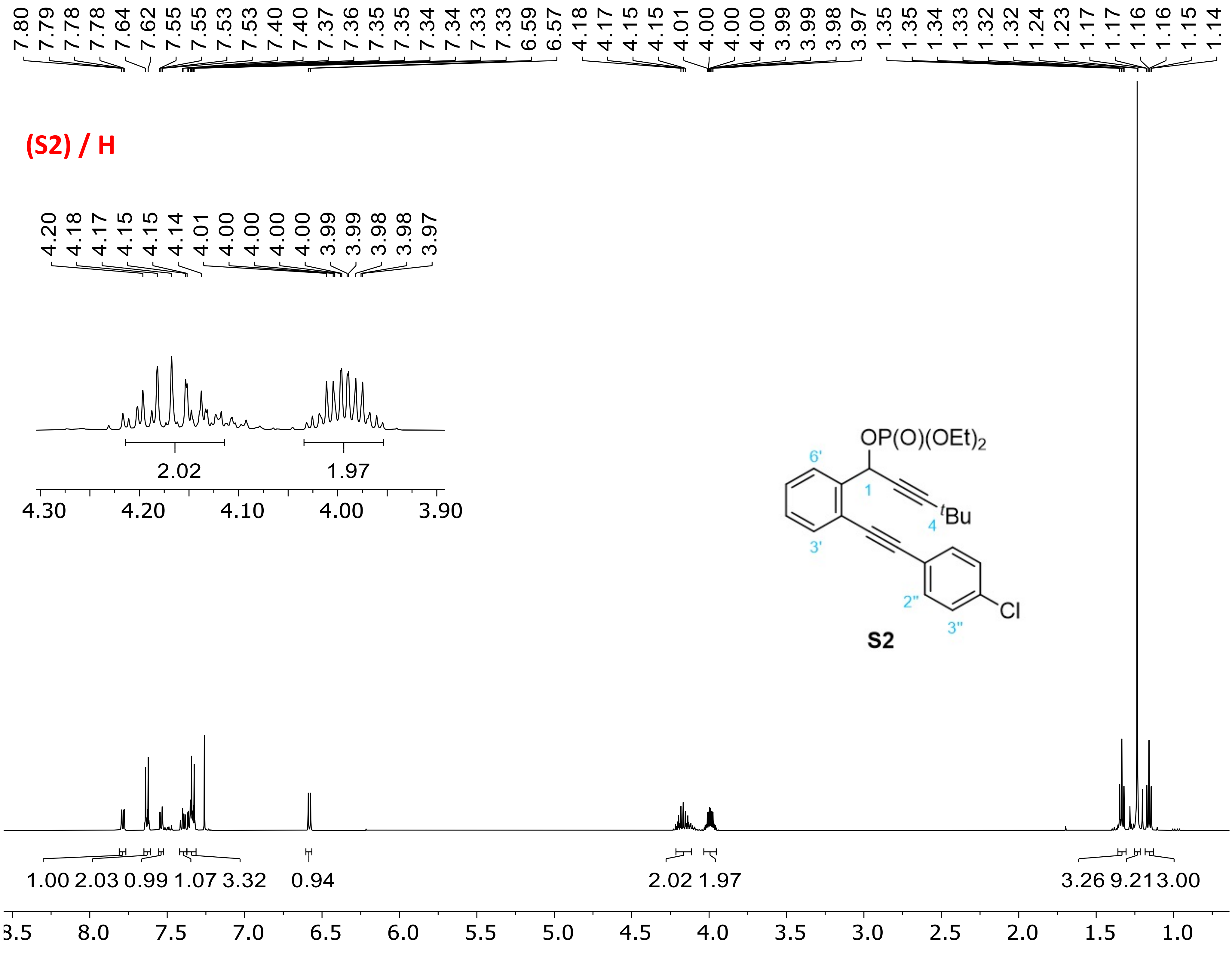

7.80 7.79 7.78 7.78 7.64 7.62 7.55 7.55 7.53 7.53 7.40 7.40 7.37 7.36 7.35 7.35 7.34 7.34 7.33 7.33 6.59 6.57 4.18 4.17 4.15 4.15 4.01 4.00 4.00 4.00 3.99 3.99 3.98 3.97 1.35 1.35 1.34 1.33 1.32 1.32 1.24 1.23 1.17 1.17 1.16 1.16 1.15 1.14
(S2) / H
4.20 4.18 4.17 4.15 4.15 4.14 4.01 4.00 4.00 4.00 4.00 3.99 3.99 3.98 3.98 3.97
2.02
1.97
4.30 4.20 4.10 4.00 3.90
OP(O)(OEt)2
6'
1
tBu
4
3'
2''
3''
Cl
S2
1.00 2.03 0.99 1.07 3.32 0.94 2.02 1.97 3.26 9.21 3.00
8.5 8.0 7.5 7.0 6.5 6.0 5.5 5.0 4.5 4.0 3.5 3.0 2.5 2.0 1.5 1.0

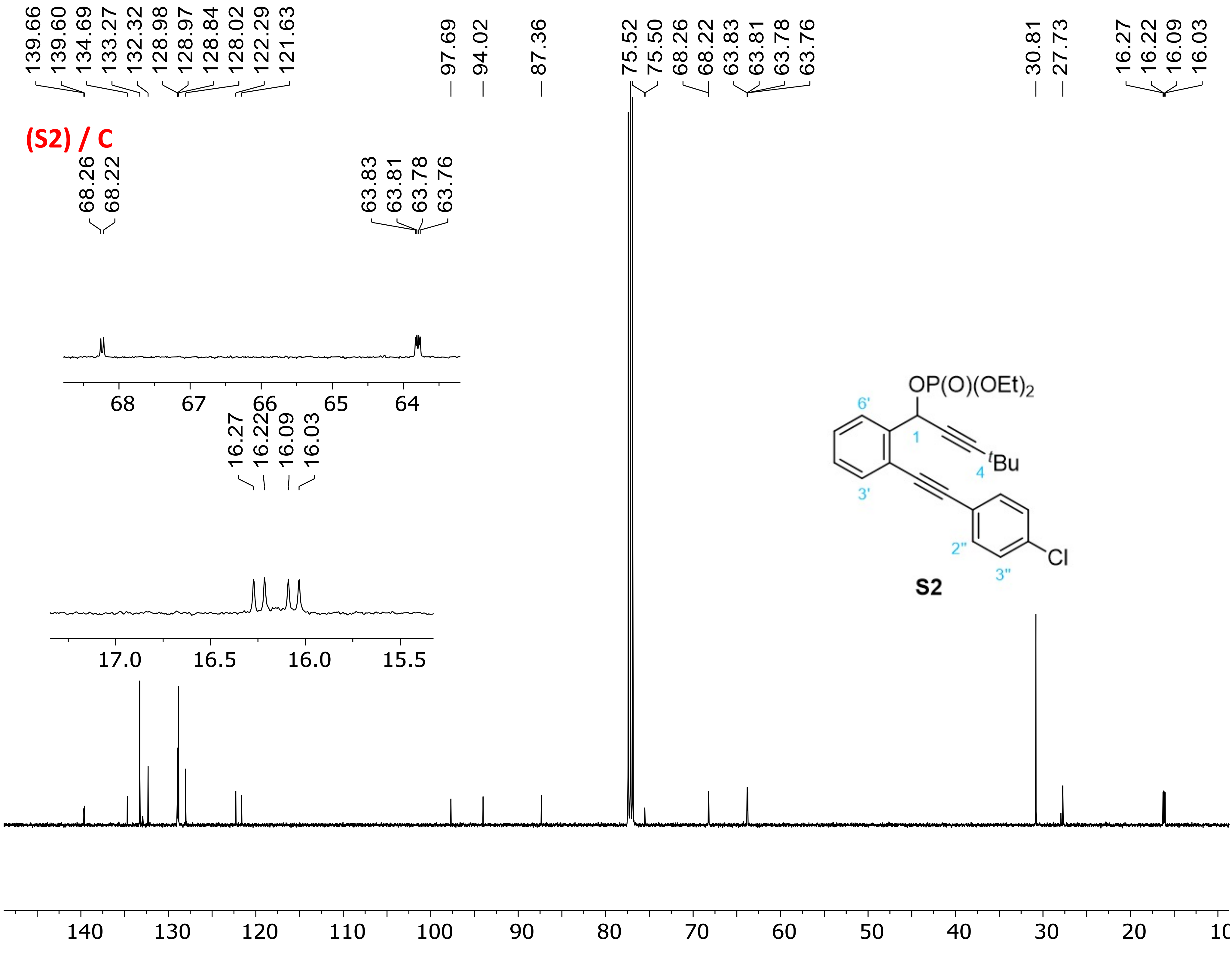
139.66
139.60
134.69
133.27
132.32
128.98
128.97
128.84
128.02
122.29
121.63
97.69
94.02
87.36
75.52
75.50
68.26
68.22
63.83
63.81
63.78
63.76
30.81
27.73
16.27
16.22
16.09
16.03
(S2) / C
OP(O)(OEt)2
6'
1
tBu
4
3'
2''
3''
Cl
S2
68
67
66
65
64
17.0
16.5
16.0
15.5
140
130
120
110
100
90
80
70
60
50
40
30
20
10

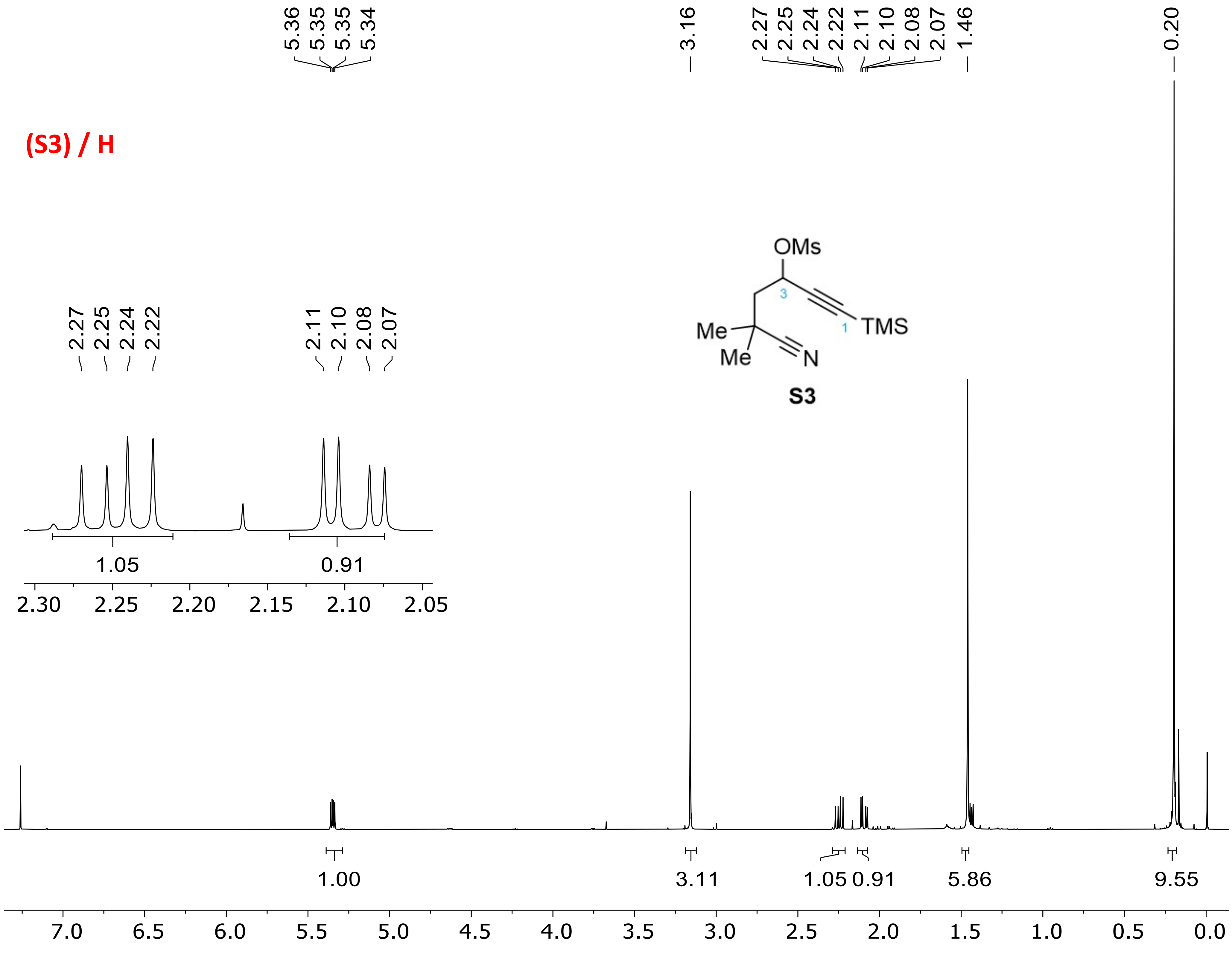
5.36
5.35
5.35
5.34
3.16
2.27
2.25
2.24
2.22
2.11
2.10
2.08
2.07
1.46
0.20
(S3) / H
OMs
3
1
TMS
Me
Me
N
S3
1.05
0.91
2.30 2.25 2.20 2.15 2.10 2.05
1.00
3.11
1.05 0.91
5.86
9.55
7.0 6.5 6.0 5.5 5.0 4.5 4.0 3.5 3.0 2.5 2.0 1.5 1.0 0.5 0.0

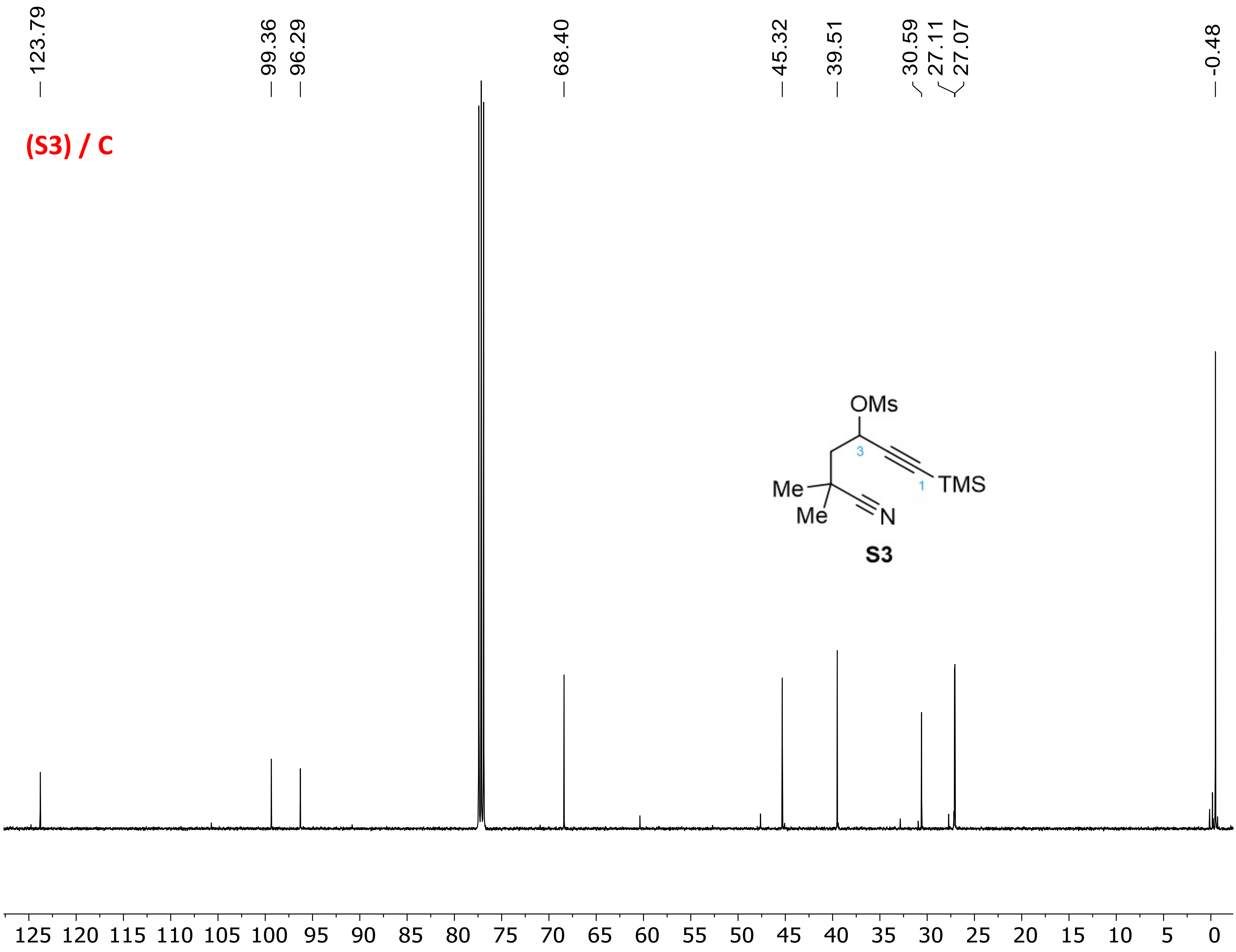
123.79
99.36
96.29
68.40
45.32
39.51
30.59
27.11
27.07
-0.48
(S3) / C
125 120 115 110 105 100 95 90 85 80 75 70 65 60 55 50 45 40 35 30 25 20 15 10 5 0
OMs
3
1
TMS
Me
Me
N

S3

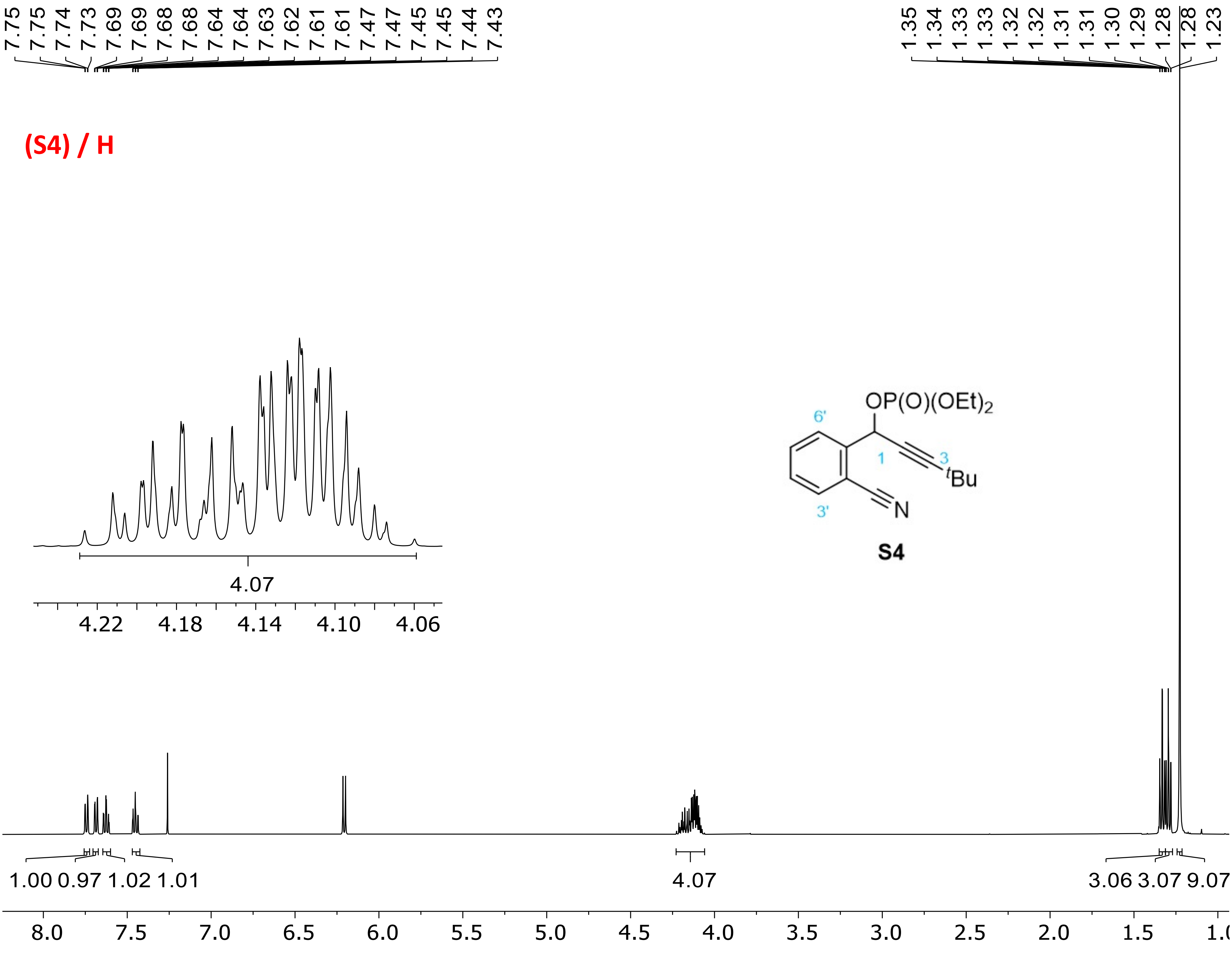
7.75 7.75 7.74 7.73 7.69 7.69 7.68 7.68 7.64 7.64 7.63 7.62 7.61 7.61 7.47 7.47 7.45 7.45 7.44 7.43
1.35 1.34 1.33 1.33 1.32 1.32 1.31 1.31 1.30 1.29 1.28 1.28 1.23
(S4) / H
4.07
4.22 4.18 4.14 4.10 4.06
OP(O)(OEt)2
6'
1
3
tBu
N
3'
S4
1.00 0.97 1.02 1.01
4.07
3.06 3.07 9.07
8.0 7.5 7.0 6.5 6.0 5.5 5.0 4.5 4.0 3.5 3.0 2.5 2.0 1.5

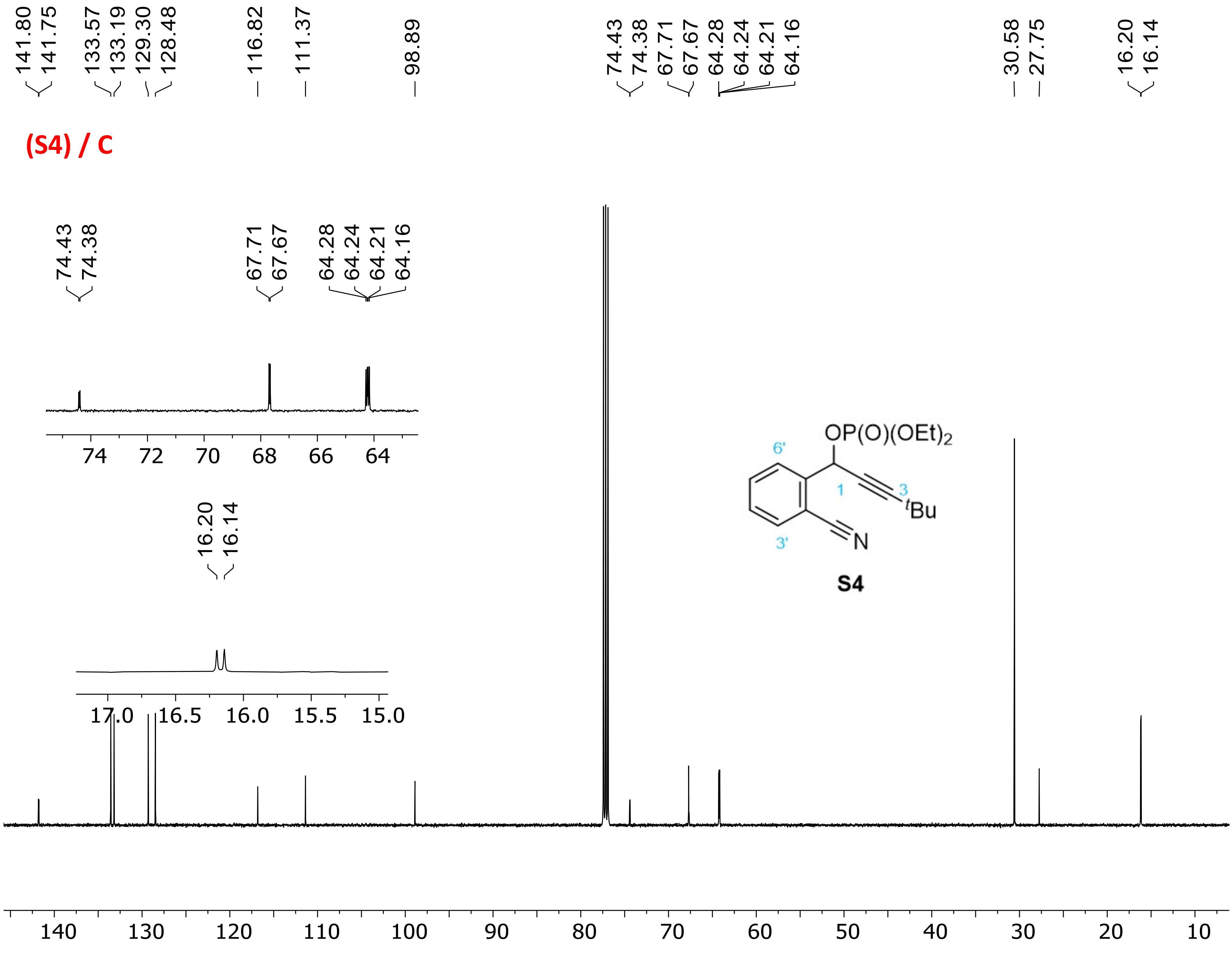
141.80
141.75
133.57
133.19
129.30
128.48
116.82
111.37
98.89
74.43
74.38
67.71
67.67
64.28
64.24
64.21
64.16
30.58
27.75
16.20
16.14
(S4) / C
OP(O)(OEt)2
6'
1
3
tBu
3'
N
S4

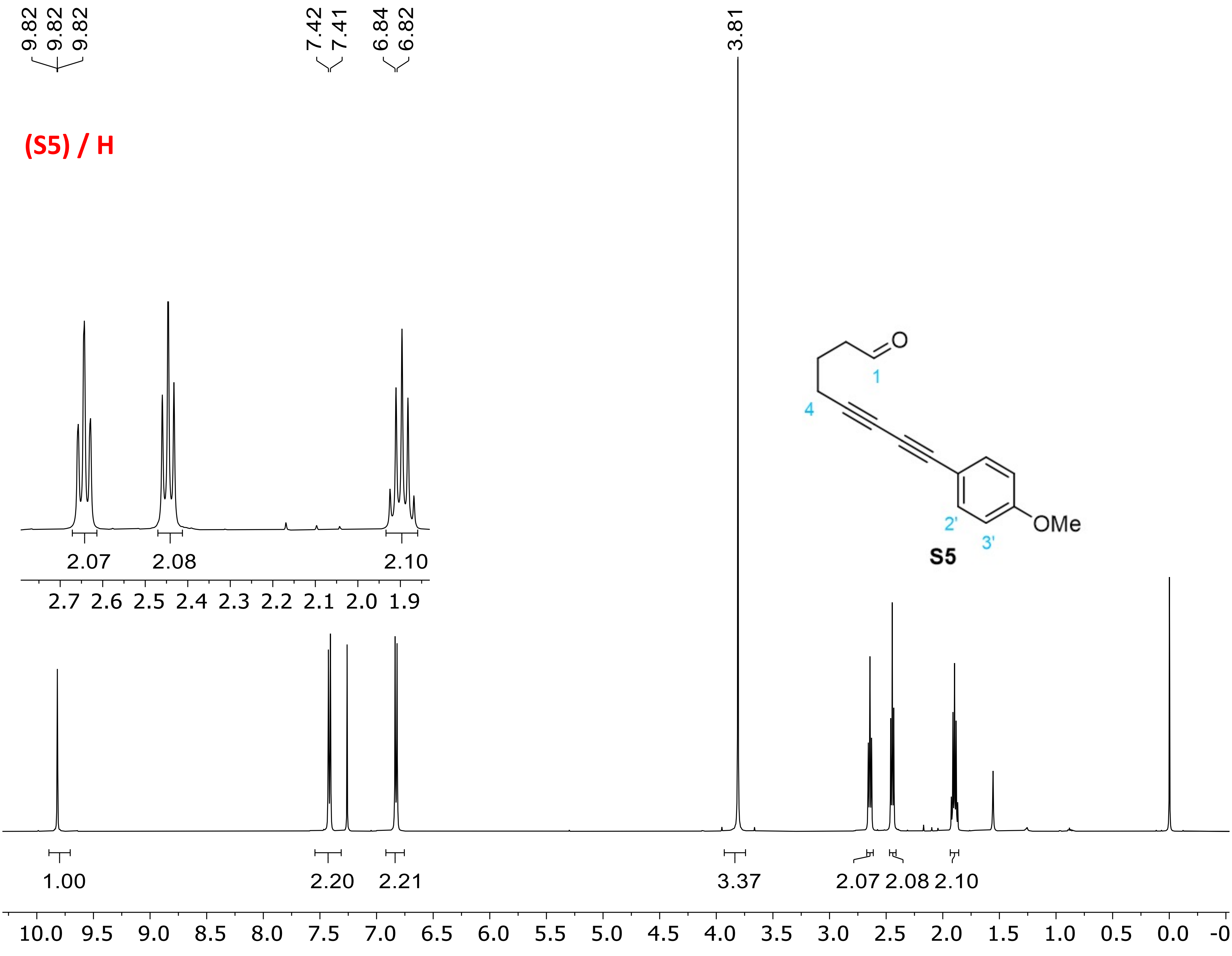
9.82
9.82
9.82
7.42
7.41
6.84
6.82
3.81
(S5) / H
O
1
4
2'
3'
OMe
S5
2.07
2.08
2.10
2.7 2.6 2.5 2.4 2.3 2.2 2.1 2.0 1.9
1.00
2.20
2.21
3.37
2.07
2.08
2.10
10.0 9.5 9.0 8.5 8.0 7.5 7.0 6.5 6.0 5.5 5.0 4.5 4.0 3.5 3.0 2.5 2.0 1.5 1.0 0.5 0.0 -0

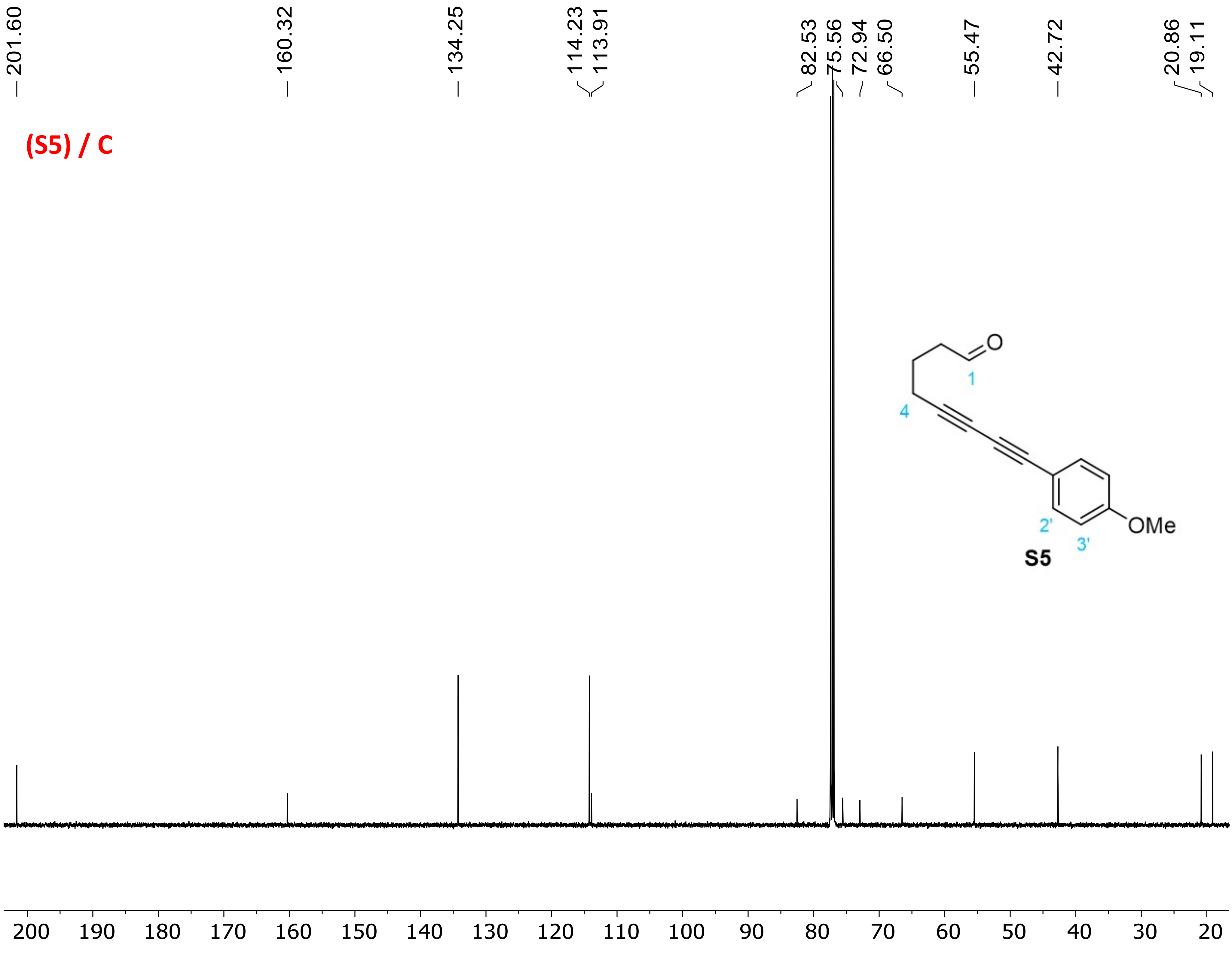
201.60
160.32
134.25
114.23
113.91
82.53
75.56
72.94
66.50
55.47
42.72
20.86
19.11
(S5) / C
O
1
4
2'
3'
OMe
S5
200
190
180
170
160
150
140
130
120
110
100
90
80
70
60
50
40
30
20

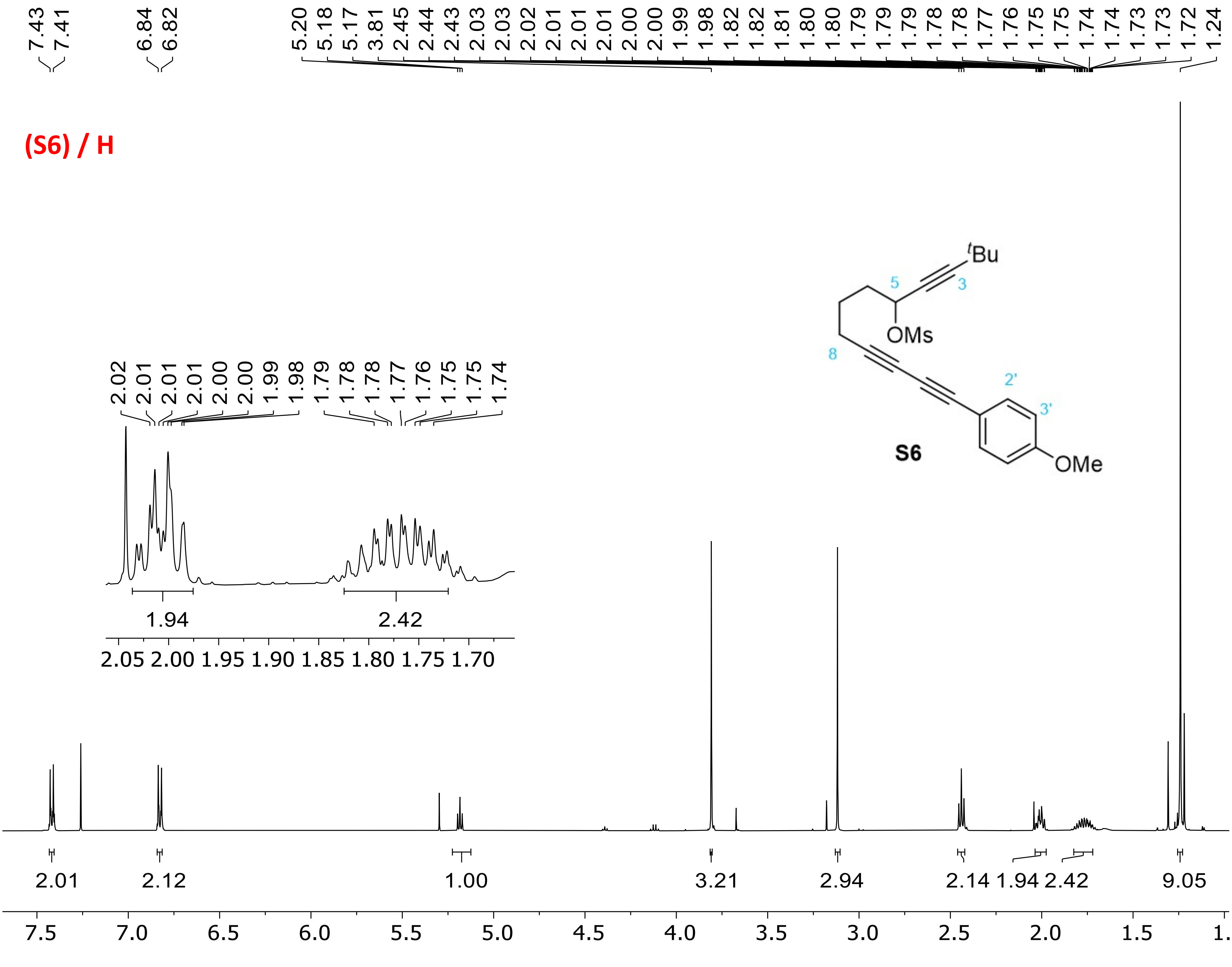
7.43
7.41
6.84
6.82
5.20
5.18
5.17
3.81
2.45
2.44
2.43
2.03
2.03
2.02
2.01
2.01
2.01
2.00
2.00
1.99
1.98
1.82
1.82
1.81
1.80
1.80
1.79
1.79
1.79
1.78
1.78
1.77
1.76
1.75
1.75
1.74
1.74
1.73
1.73
1.72
1.24
(S6) / H
^{t}Bu
5
3
OMs
8
2'
3'
S6
OMe
2.02 2.01 2.01 2.01 2.00 2.00 1.99 1.98 1.79 1.78 1.78 1.77 1.76 1.75 1.75 1.74
1.94
2.42
2.05 2.00 1.95 1.90 1.85 1.80 1.75 1.70
2.01
2.12
1.00
3.21
2.94
2.14 1.94 2.42
9.05
7.5 7.0 6.5 6.0 5.5 5.0 4.5 4.0 3.5 3.0 2.5 2.0 1.5 1.

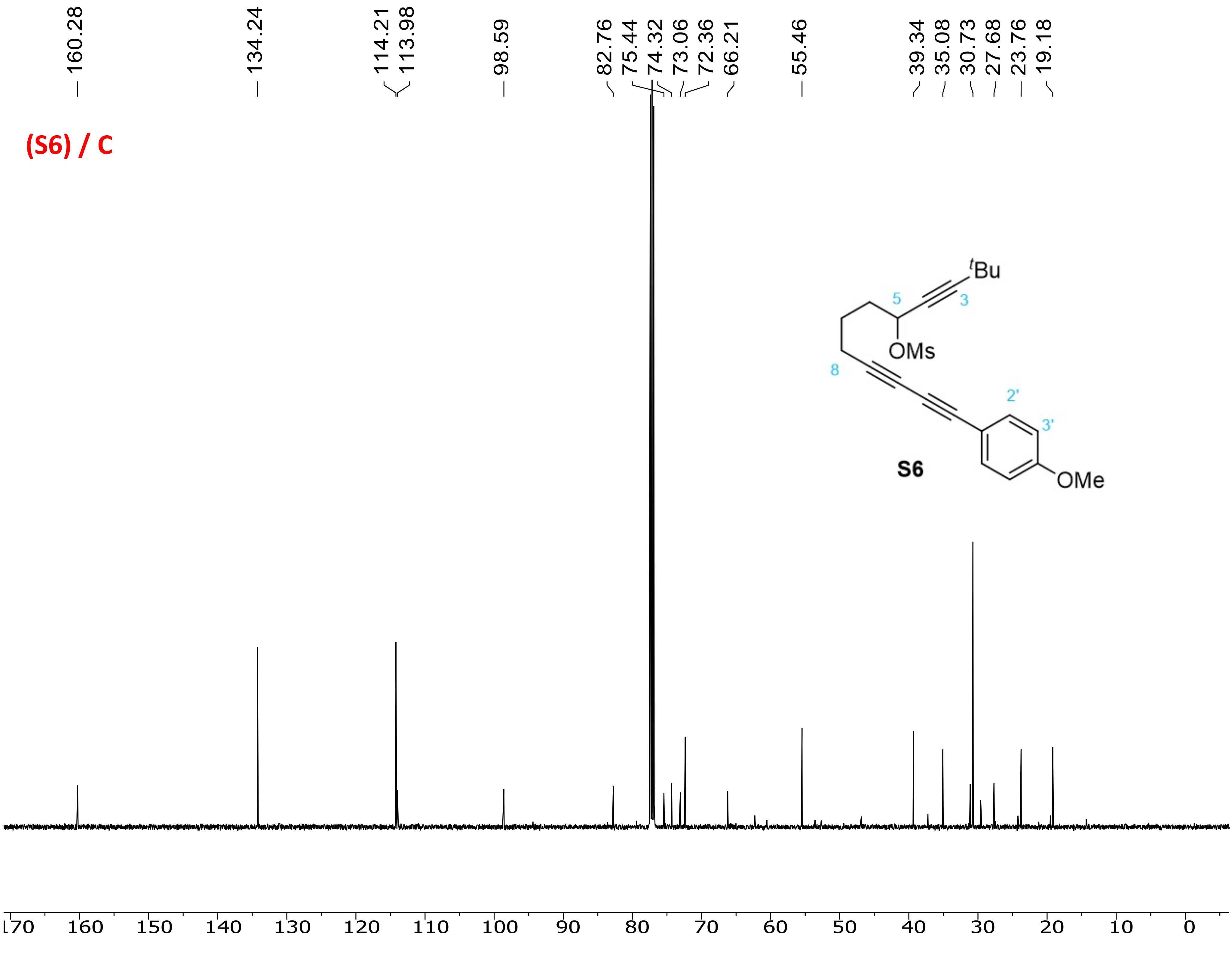
160.28
134.24
114.21
113.98
98.59
82.76
75.44
74.32
73.06
72.36
66.21
55.46
39.34
35.08
30.73
27.68
23.76
19.18
(S6) / C
tBu
5
3
OMs
8
2'
3'
S6
OMe
170
160
150
140
130
120
110
100
90
80
70
60
50
40
30
20
10
0

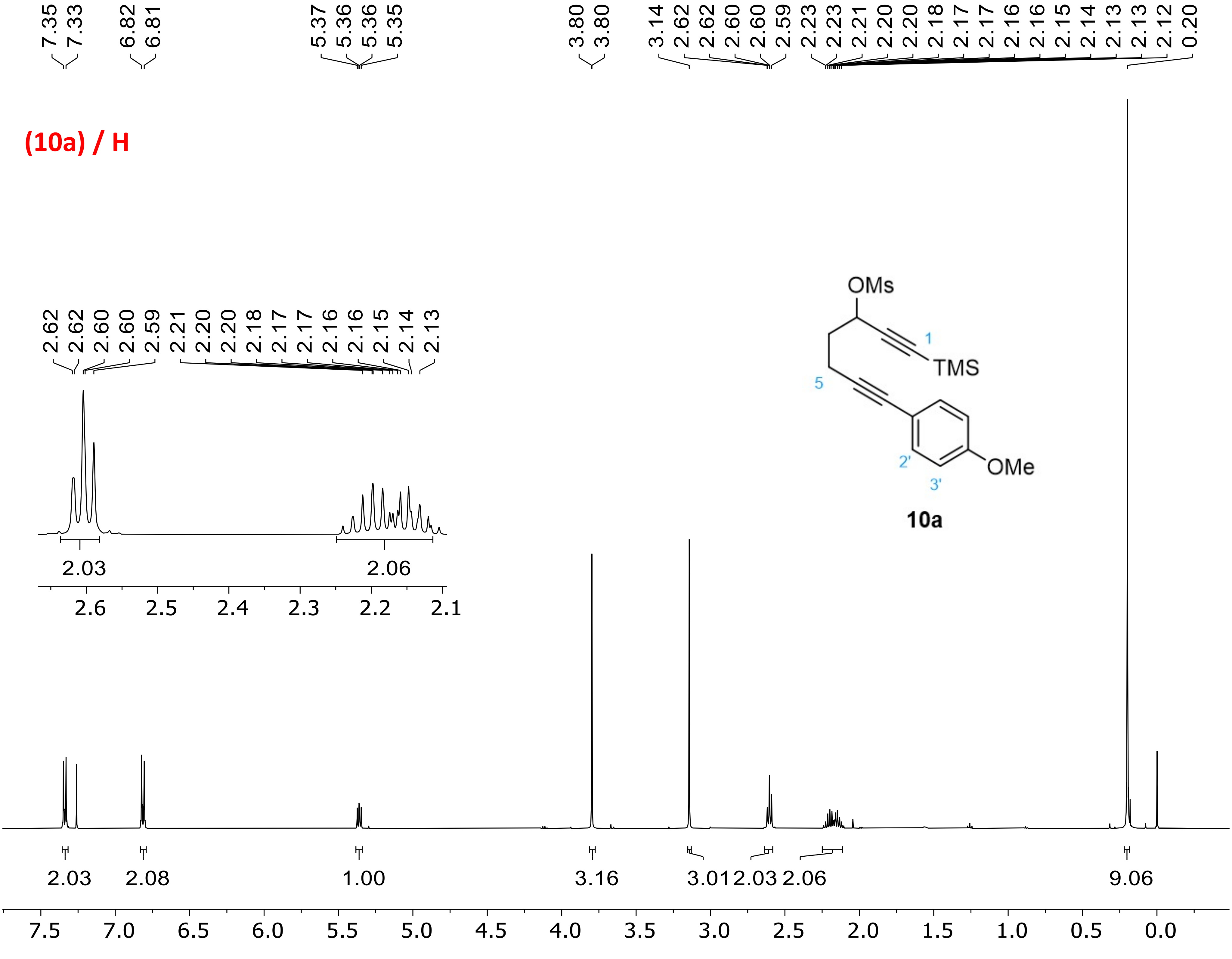
7.35
7.33
6.82
6.81
5.37
5.36
5.36
5.35
3.80
3.80
3.14
2.62
2.62
2.60
2.60
2.59
2.23
2.23
2.21
2.20
2.20
2.18
2.17
2.17
2.16
2.16
2.15
2.14
2.13
2.13
2.12
0.20
(10a) / H
OMs
1
TMS
5
2'
3'
OMe
10a
2.62
2.62
2.60
2.60
2.59
2.21
2.20
2.20
2.18
2.17
2.17
2.16
2.16
2.15
2.14
2.13
2.03
2.06
2.6
2.5
2.4
2.3
2.2
2.1
2.03
2.08
1.00
3.16
3.01
2.03
2.06
9.06
7.5
7.0
6.5
6.0
5.5
5.0
4.5
4.0
3.5
3.0
2.5
2.0
1.5
1.0
0.5
0.0

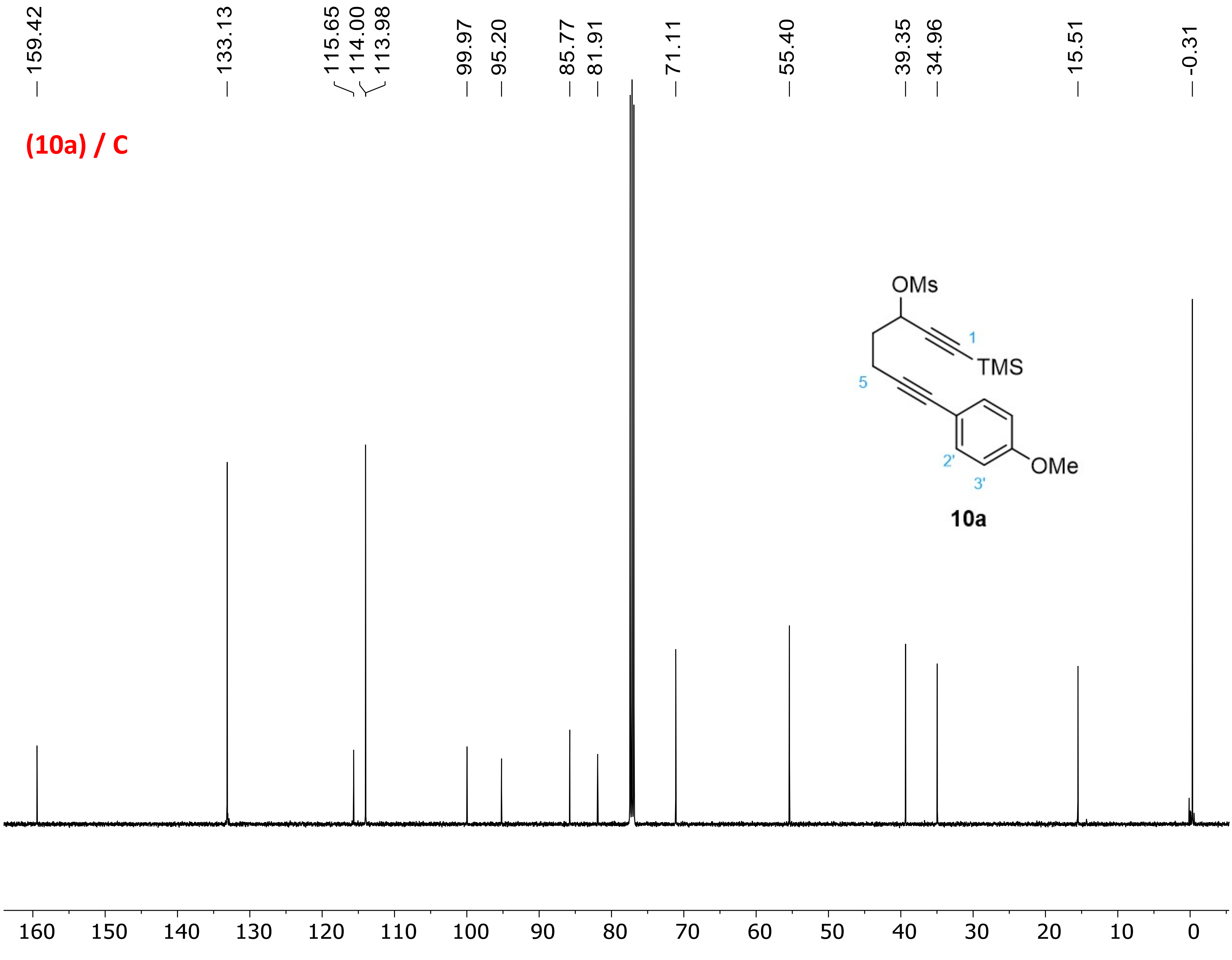

159.42
133.13
115.65
114.00
113.98
99.97
95.20
85.77
81.91
71.11
55.40
39.35
34.96
15.51
-0.31
(10a) / C
OMs
1
TMS
5
2'
3'
OMe
10a
160 150 140 130 120 110 100 90 80 70 60 50 40 30 20 10 0

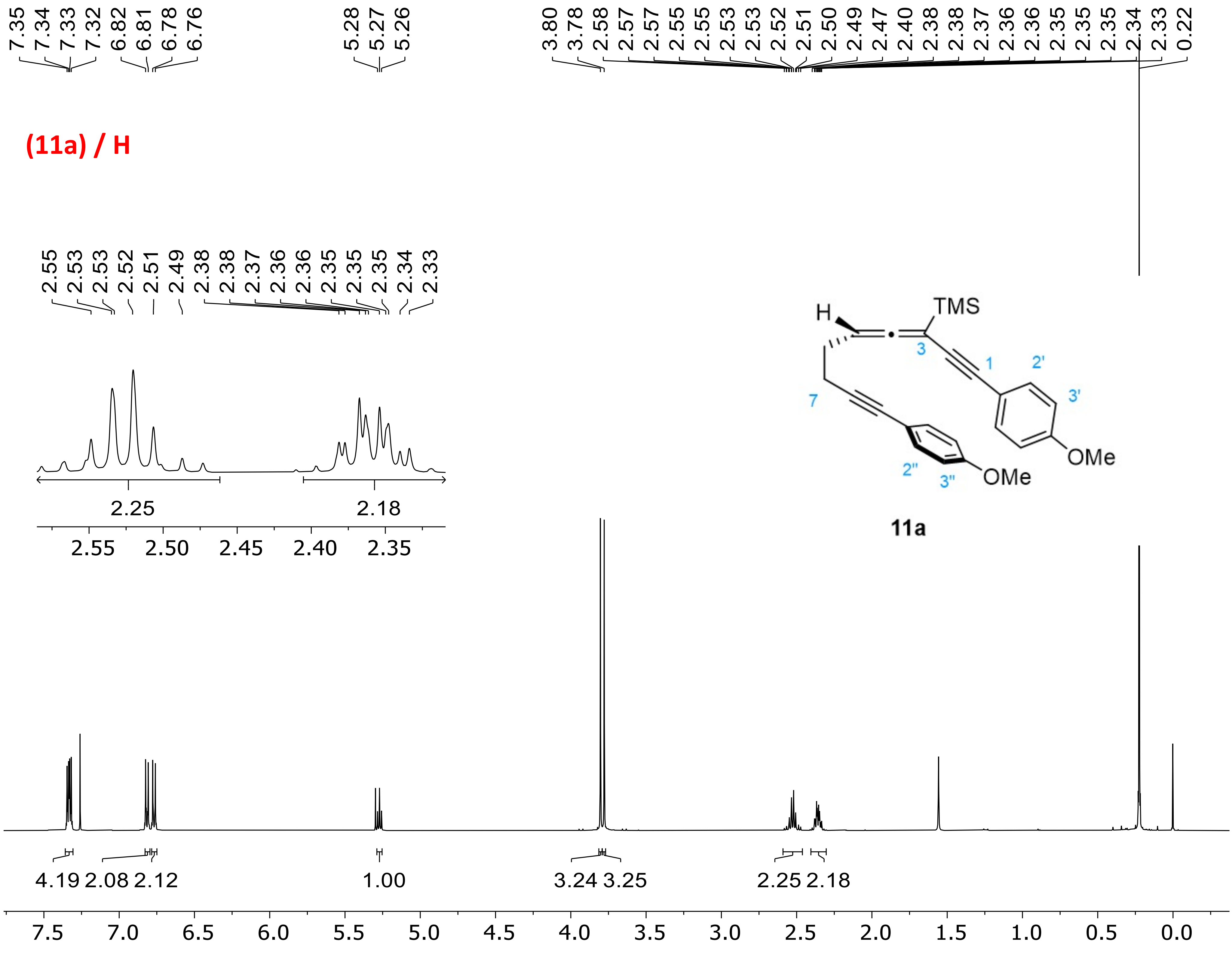

7.35 7.34 7.33 7.32 6.82 6.81 6.78 6.76
5.28 5.27 5.26
3.80 3.78 2.58 2.57 2.57 2.55 2.55 2.53 2.53 2.52 2.51 2.50 2.49 2.47 2.40 2.38 2.38 2.37 2.36 2.36 2.35 2.35 2.35 2.34 2.33 0.22
(11a) / H
2.55 2.53 2.53 2.52 2.51 2.49 2.38 2.38 2.37 2.36 2.36 2.35 2.35 2.35 2.34 2.33
2.25
2.18
2.55 2.50 2.45 2.40 2.35
H
TMS
3
1
2'
3'
7
OMe
2''
3''
OMe
11a
4.19 2.08 2.12
1.00
3.24 3.25
2.25 2.18
7.5 7.0 6.5 6.0 5.5 5.0 4.5 4.0 3.5 3.0 2.5 2.0 1.5 1.0 0.5 0.0

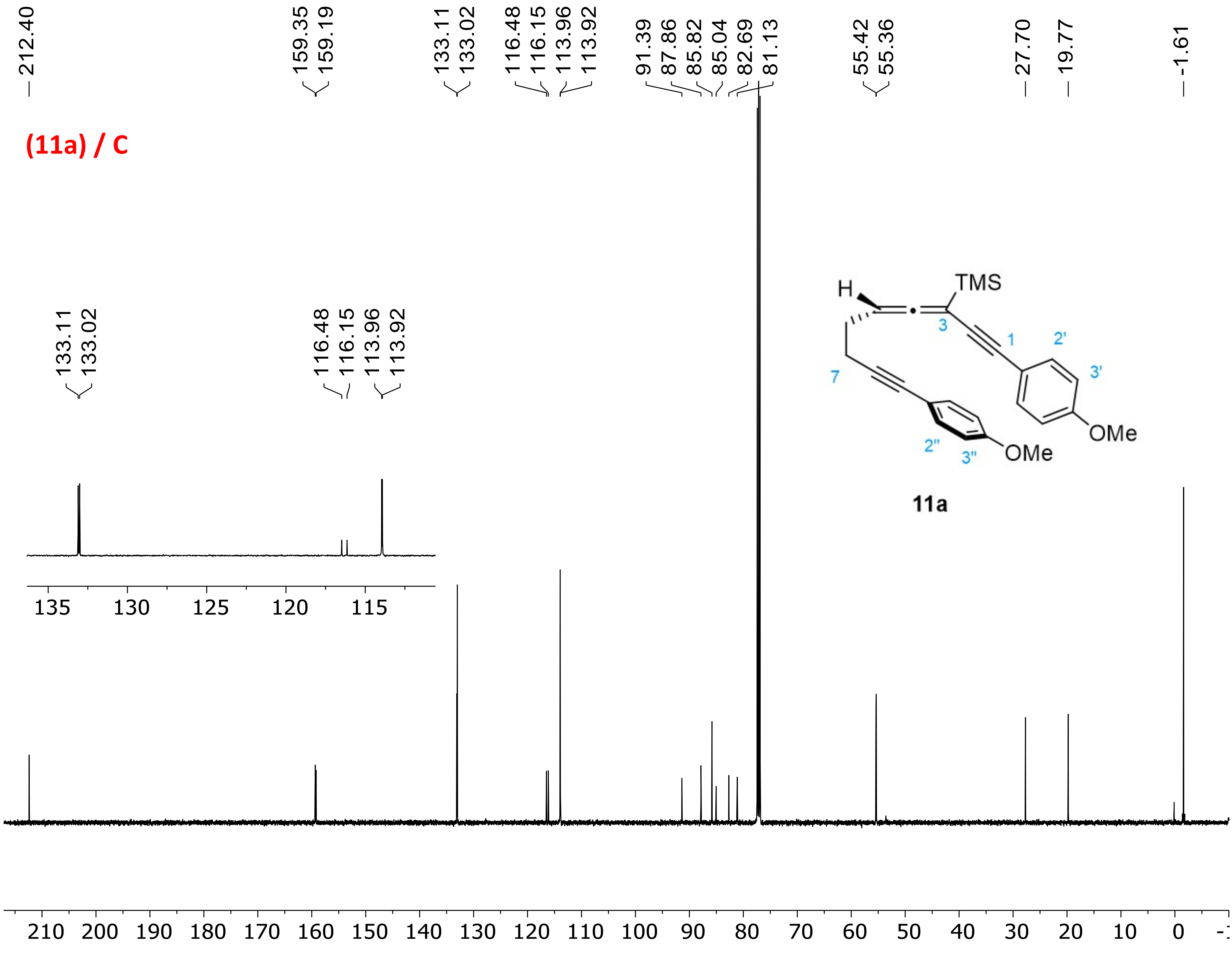
212.40
159.35
159.19
133.11
133.02
116.48
116.15
113.96
113.92
91.39
87.86
85.82
85.04
82.69
81.13
55.42
55.36
27.70
19.77
-1.61
(11a) / C
133.11
133.02
116.48
116.15
113.96
113.92
135 130 125 120 115
H
TMS
3
1
2'
3'
7
OMe
2''
3''
OMe
11a
210 200 190 180 170 160 150 140 130 120 110 100 90 80 70 60 50 40 30 20 10 0

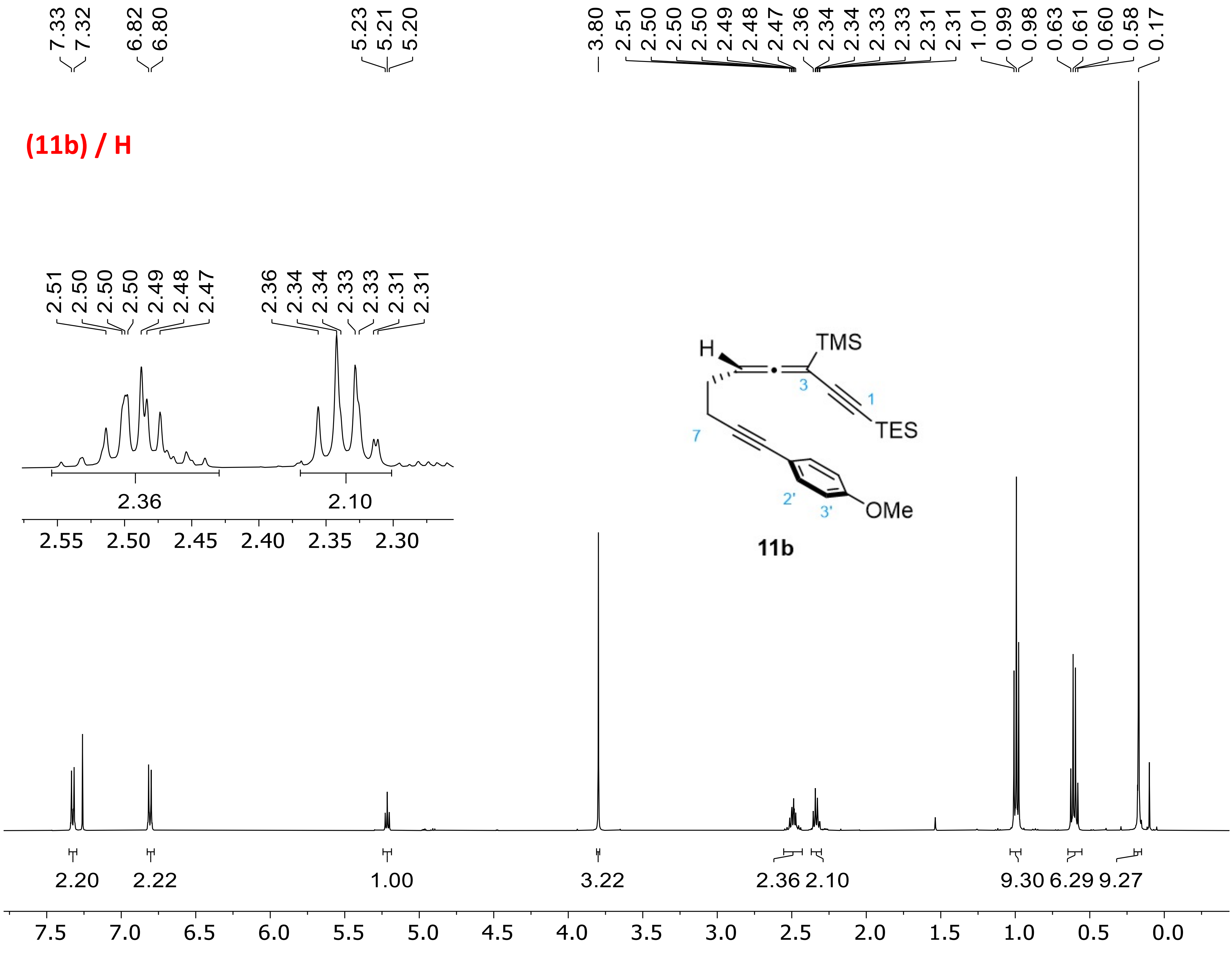
7.33
7.32
6.82
6.80
5.23
5.21
5.20
3.80
2.51
2.50
2.50
2.50
2.49
2.48
2.47
2.36
2.34
2.34
2.33
2.33
2.31
2.31
1.01
0.99
0.98
0.63
0.61
0.60
0.58
0.17
(11b) / H
2.36
2.10
2.55 2.50 2.45 2.40 2.35 2.30
H
TMS
3
1
TES
7
2'
3'
OMe
11b
2.20 2.22 1.00 3.22 2.36 2.10 9.30 6.29 9.27
7.5 7.0 6.5 6.0 5.5 5.0 4.5 4.0 3.5 3.0 2.5 2.0 1.5 1.0 0.5 0.0

(11b) / C

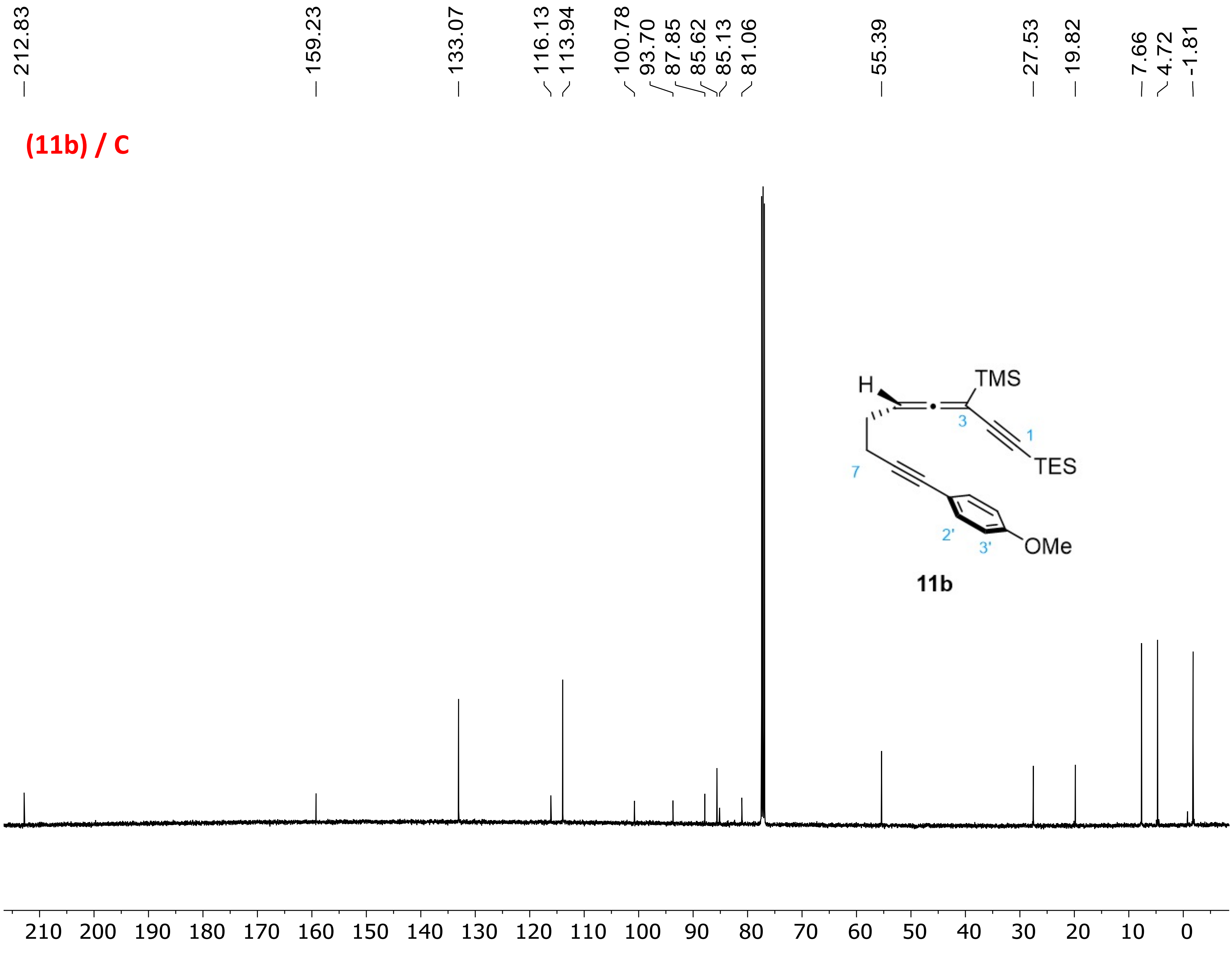
212.83
159.23
133.07
116.13
113.94
100.78
93.70
87.85
85.62
85.13
81.06
55.39
27.53
19.82
7.66
4.72
-1.81
H
TMS
3
1
TES
7
2'
3'
OMe
11b
210
200
190
180
170
160
150
140
130
120
110
100
90
80
70
60
50
40
30
20
10
0

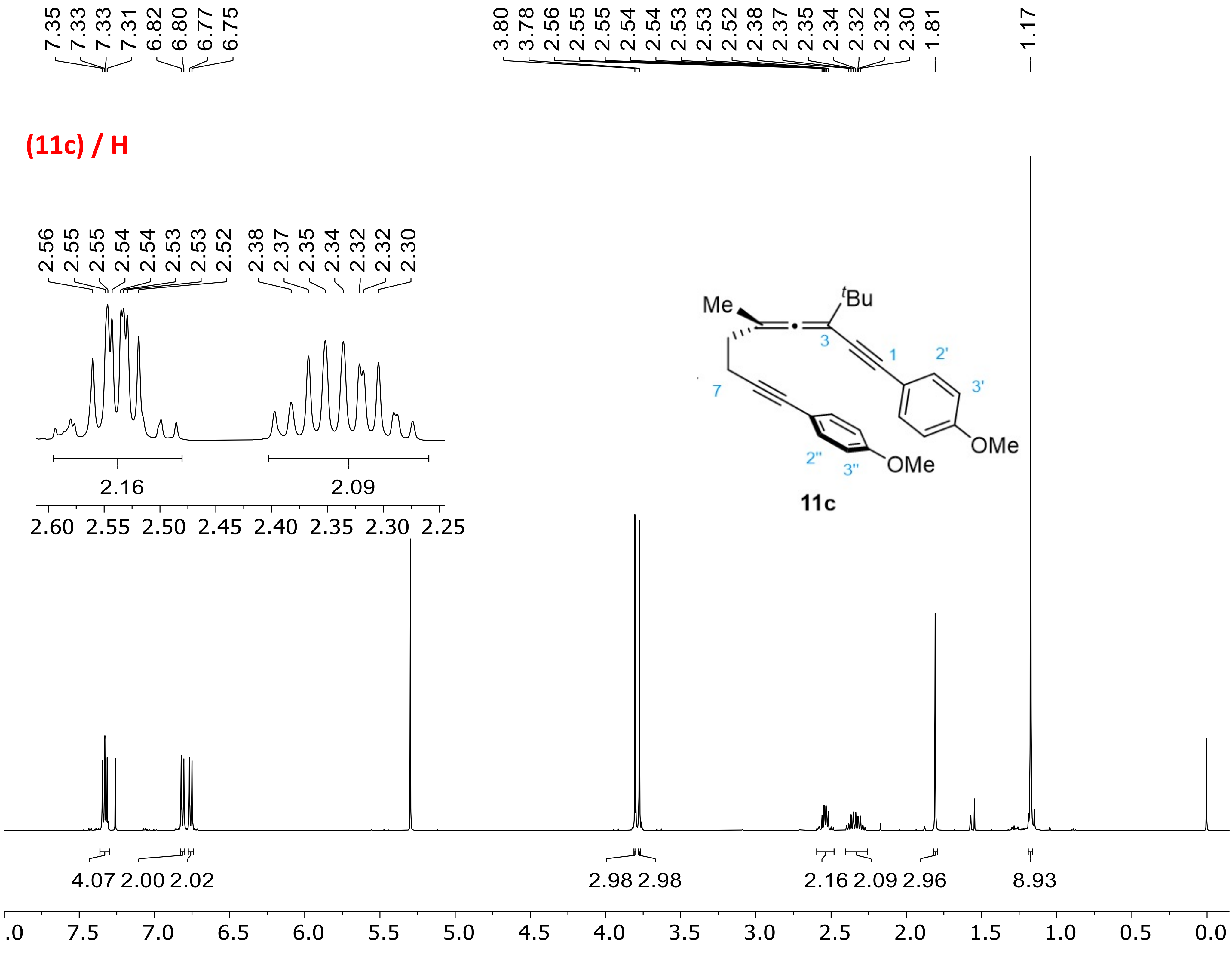
7.35 7.33 7.33 7.31 6.82 6.80 6.77 6.75
3.80 3.78 2.56 2.55 2.55 2.54 2.54 2.53 2.53 2.52 2.38 2.37 2.35 2.34 2.32 2.32 2.30 1.81 1.17
(11c) / H
Me
tBu
3
1
2'
3'
7
OMe
2''
3''
OMe
11c
2.16
2.09
2.60 2.55 2.50 2.45 2.40 2.35 2.30 2.25
4.07 2.00 2.02
2.98 2.98
2.16 2.09 2.96
8.93
7.5 7.0 6.5 6.0 5.5 5.0 4.5 4.0 3.5 3.0 2.5 2.0 1.5 1.0 0.5 0.0

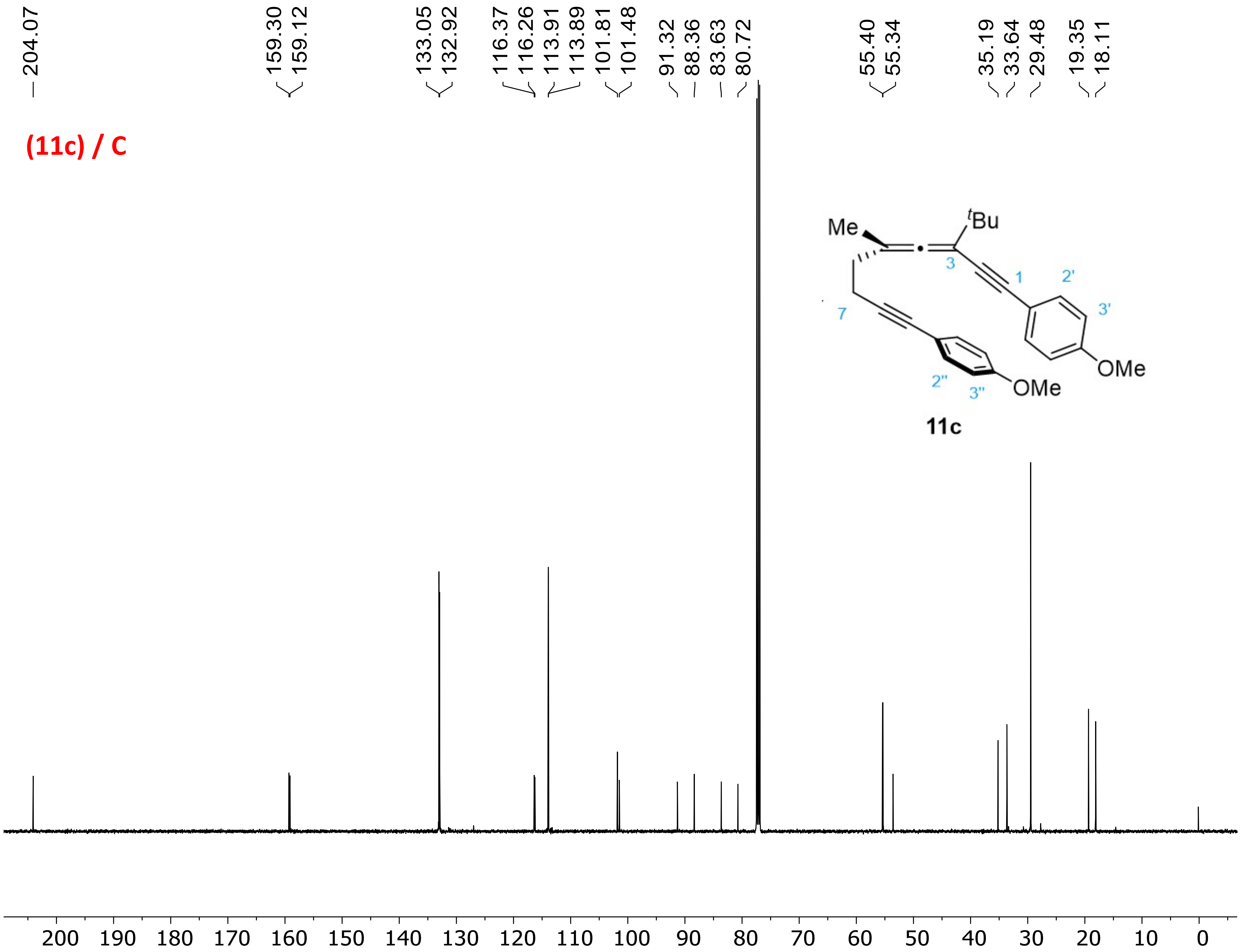
204.07
159.30
159.12
133.05
132.92
116.37
116.26
113.91
113.89
101.81
101.48
91.32
88.36
83.63
80.72
55.40
55.34
35.19
33.64
29.48
19.35
18.11
(11c) / C
Me
tBu
3
1
2'
3'
7
2''
3''
OMe
OMe
11c
200 190 180 170 160 150 140 130 120 110 100 90 80 70 60 50 40 30 20 10 0

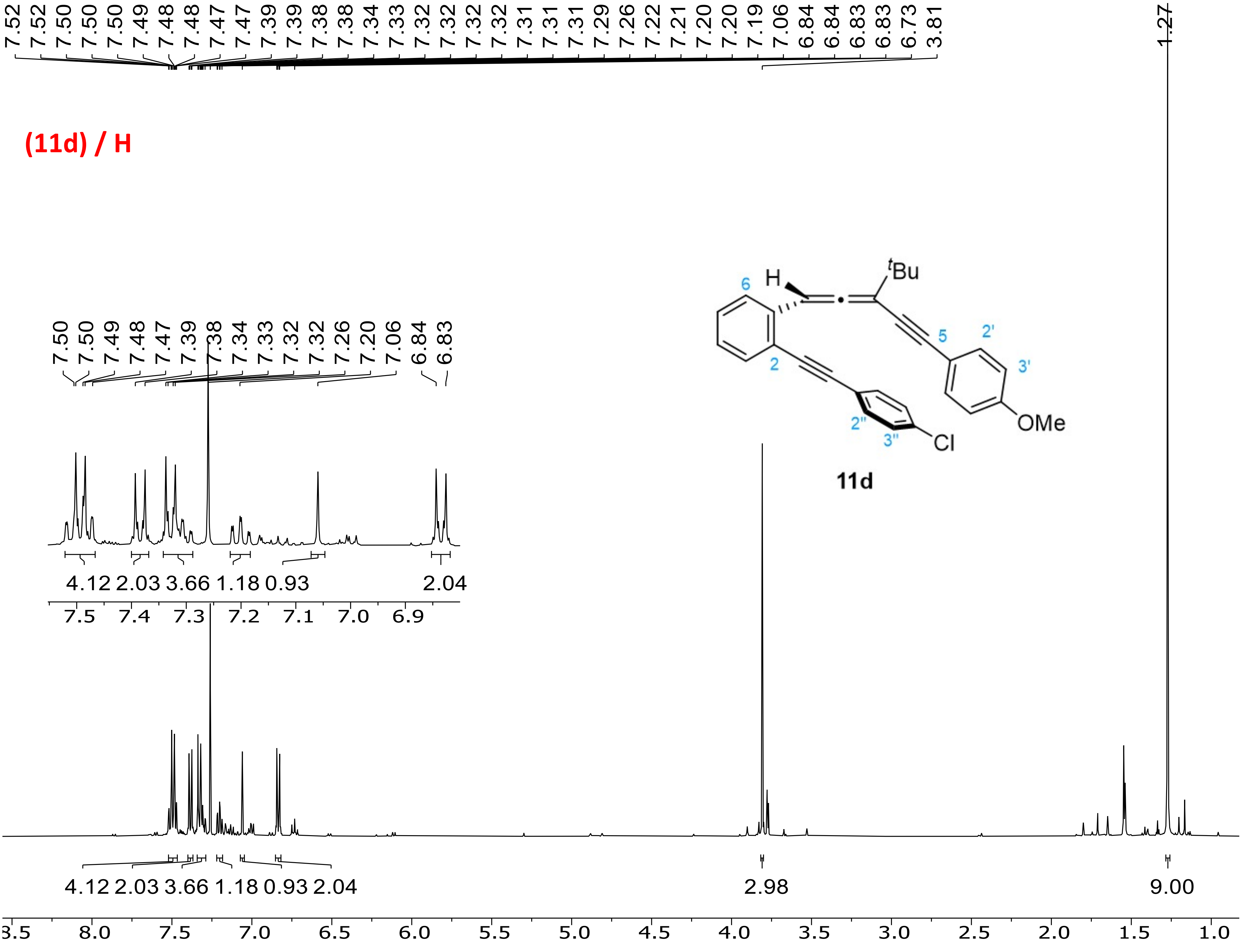
7.52 7.52 7.50 7.50 7.50 7.49 7.48 7.48 7.47 7.47 7.39 7.39 7.38 7.38 7.34 7.33 7.32 7.32 7.32 7.32 7.31 7.31 7.31 7.29 7.26 7.22 7.21 7.20 7.20 7.19 7.06 6.84 6.84 6.83 6.83 6.73 3.81
1.27
(11d) / H
7.50 7.50 7.49 7.48 7.47 7.39 7.38 7.34 7.33 7.32 7.32 7.26 7.20 7.06 6.84 6.83
4.12 2.03 3.66 1.18 0.93
2.04
7.5 7.4 7.3 7.2 7.1 7.0 6.9
H
tBu
6
5
2'
2
3'
2''
3''
Cl
OMe
11d
4.12 2.03 3.66 1.18 0.93 2.04
2.98
9.00
8.5 8.0 7.5 7.0 6.5 6.0 5.5 5.0 4.5 4.0 3.5 3.0 2.5 2.0 1.5 1.0

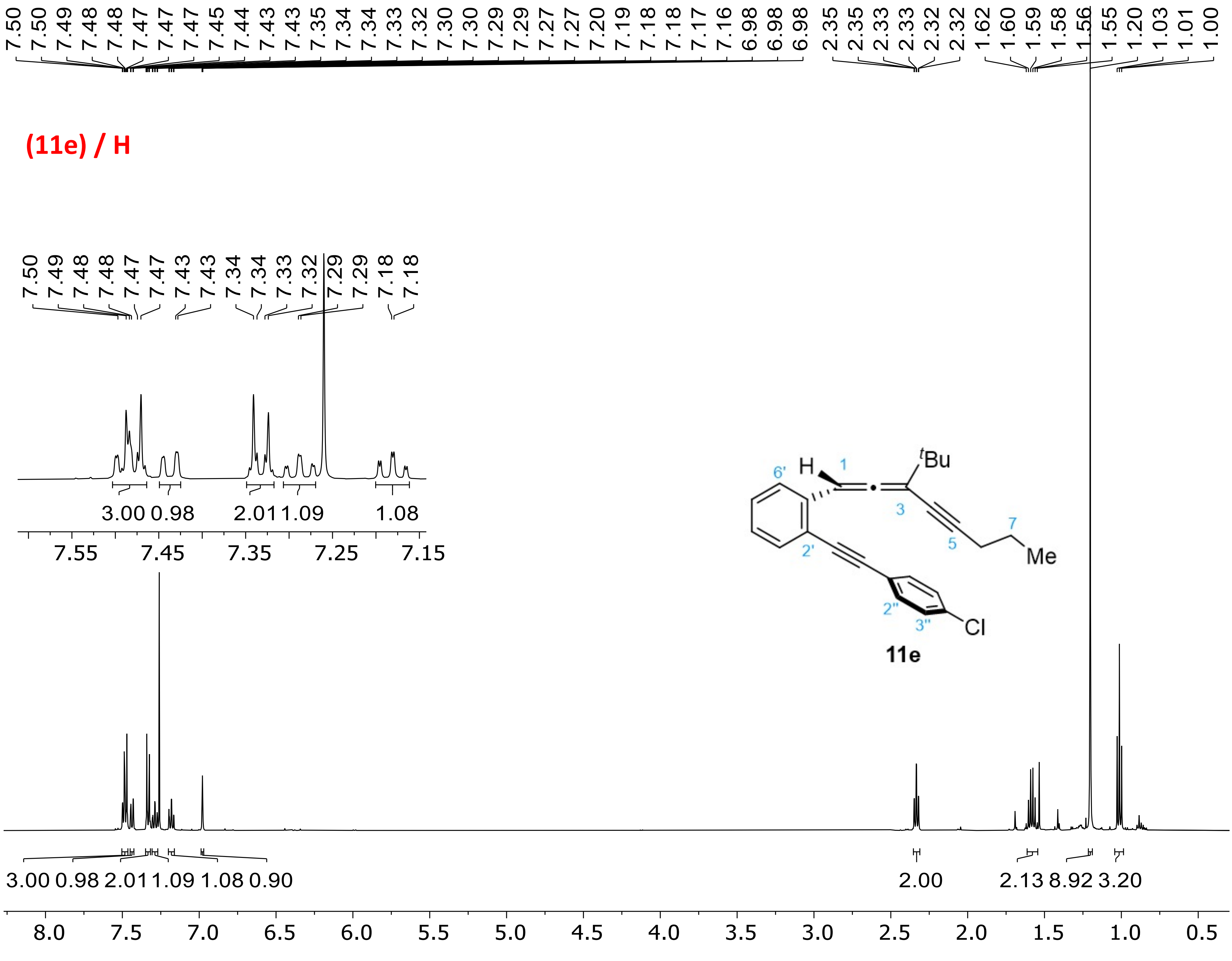
7.50 7.50 7.49 7.48 7.48 7.47 7.47 7.47 7.45 7.44 7.43 7.43 7.35 7.34 7.34 7.33 7.32 7.30 7.30 7.29 7.29 7.27 7.27 7.20 7.19 7.18 7.18 7.17 7.16 6.98 6.98 6.98 2.35 2.35 2.33 2.33 2.32 2.32 1.62 1.60 1.59 1.58 1.56 1.55 1.20 1.03 1.01 1.00
(11e) / H
7.50 7.49 7.48 7.48 7.47 7.47 7.43 7.43 7.34 7.34 7.33 7.32 7.29 7.29 7.18 7.18
3.00 0.98 2.01 1.09 1.08
7.55 7.45 7.35 7.25 7.15
H 1 tBu 6' 3 7 5 2' Me 2'' 3'' Cl
11e
3.00 0.98 2.01 1.09 1.08 0.90 2.00 2.13 8.92 3.20
8.0 7.5 7.0 6.5 6.0 5.5 5.0 4.5 4.0 3.5 3.0 2.5 2.0 1.5 1.0 0.5

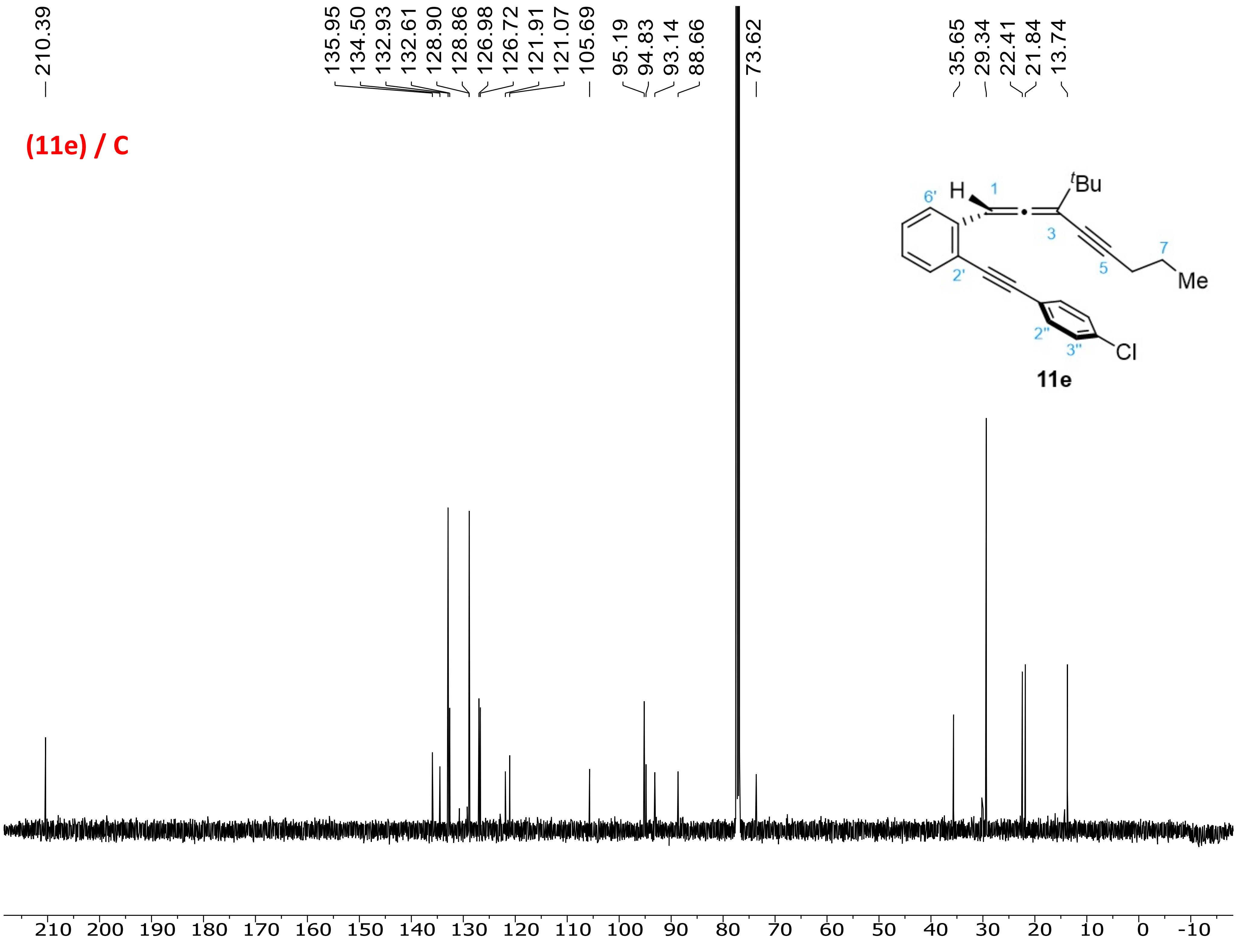
210.39
135.95
134.50
132.93
132.61
128.90
128.86
126.98
126.72
121.91
121.07
105.69
95.19
94.83
93.14
88.66
73.62
35.65
29.34
22.41
21.84
13.74
(11e) / C
H
1
6'
tBu
3
7
5
Me
2'
2''
3''
Cl
11e
210 200 190 180 170 160 150 140 130 120 110 100 90 80 70 60 50 40 30 20 10 0 -10

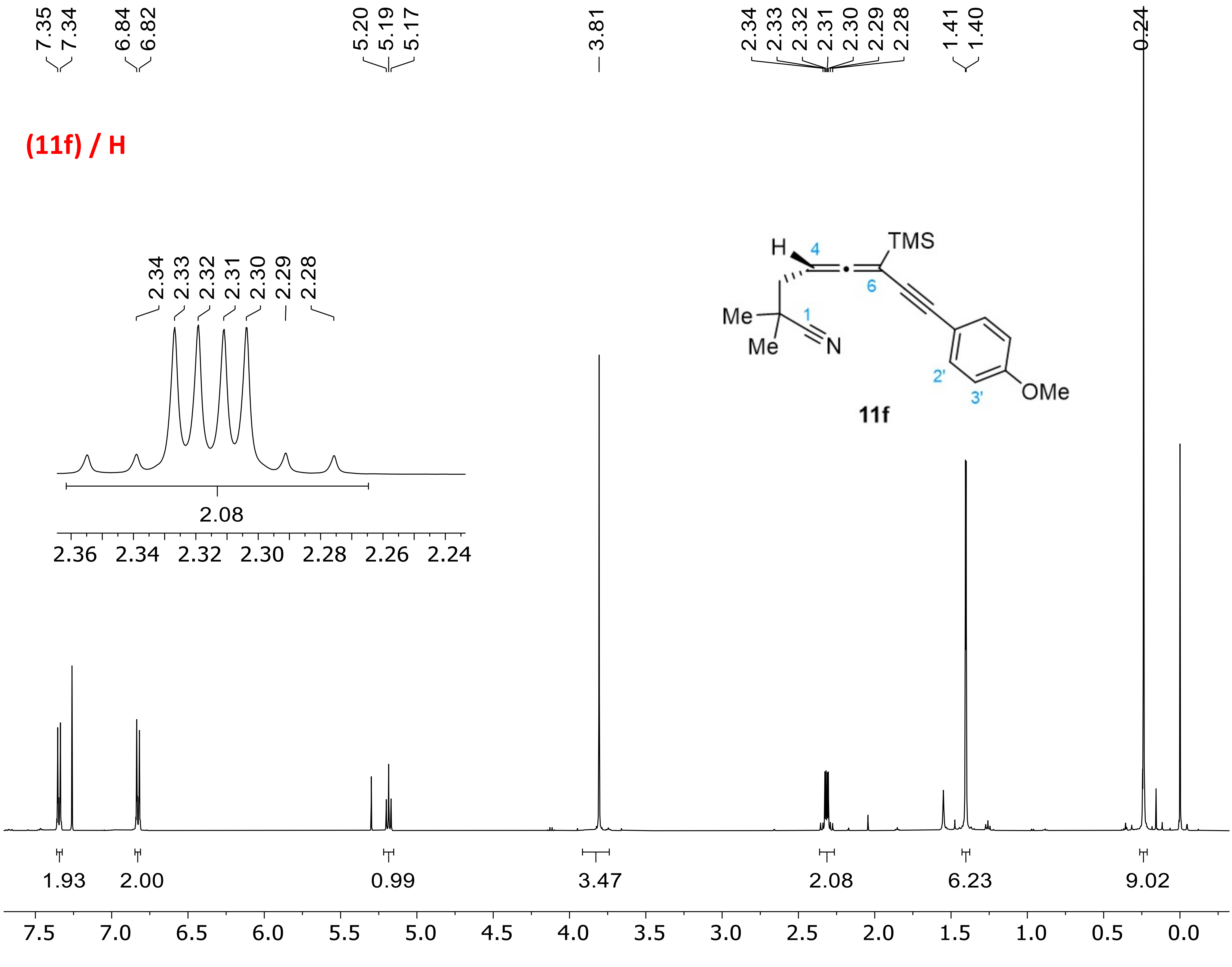

7.35
7.34
6.84
6.82
5.20
5.19
5.17
3.81
2.34
2.33
2.32
2.31
2.30
2.29
2.28
1.41
1.40
0.24
(11f) / H
H
4
TMS
6
1
Me
Me
N
2'
3'
OMe
11f
2.08
2.36 2.34 2.32 2.30 2.28 2.26 2.24
1.93
2.00
0.99
3.47
2.08
6.23
9.02
7.5 7.0 6.5 6.0 5.5 5.0 4.5 4.0 3.5 3.0 2.5 2.0 1.5 1.0 0.5 0.0

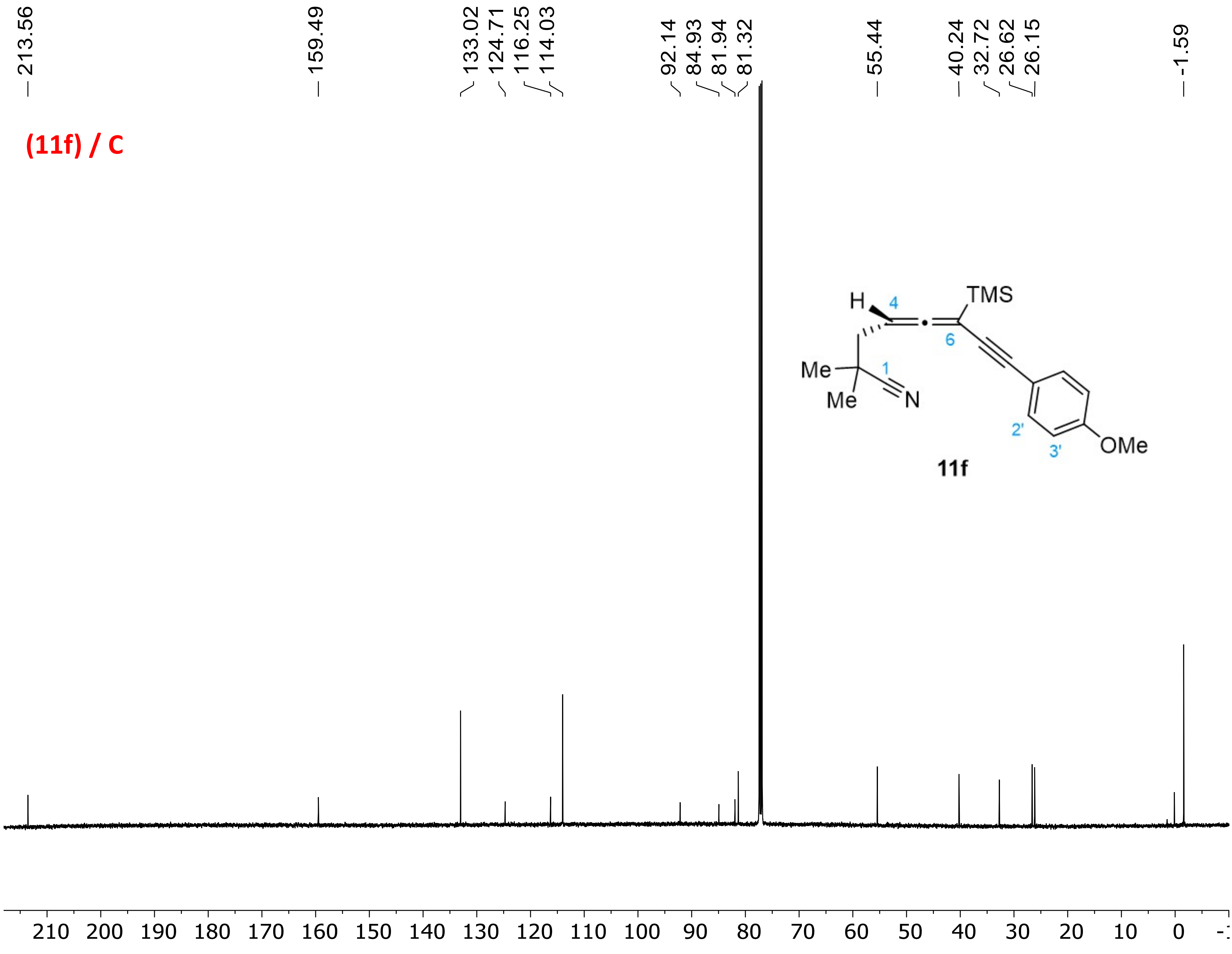
213.56
159.49
133.02
124.71
116.25
114.03
92.14
84.93
81.94
81.32
55.44
40.24
32.72
26.62
26.15
-1.59
(11f) / C
H
4
TMS
6
Me
1
Me
N
2'
3'
OMe
11f
210 200 190 180 170 160 150 140 130 120 110 100 90 80 70 60 50 40 30 20 10 0

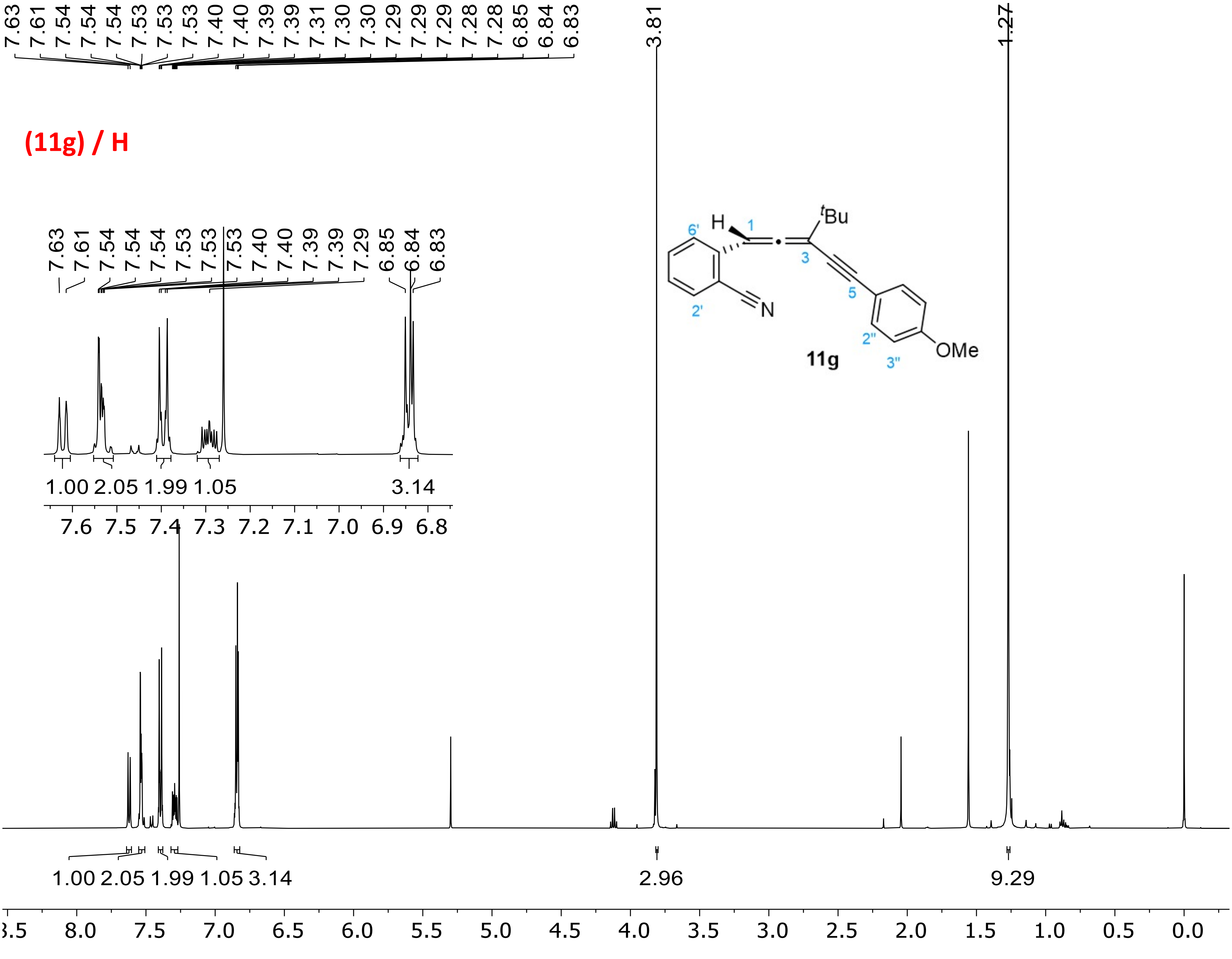
7.63 7.61 7.54 7.54 7.54 7.53 7.53 7.53 7.40 7.40 7.39 7.39 7.31 7.30 7.30 7.29 7.29 7.29 7.28 7.28 6.85 6.84 6.83
3.81
1.27
(11g) / H
7.63 7.61 7.54 7.54 7.54 7.53 7.53 7.53 7.40 7.40 7.39 7.39 7.29 6.85 6.84 6.83
1.00 2.05 1.99 1.05 3.14
7.6 7.5 7.4 7.3 7.2 7.1 7.0 6.9 6.8
H
1
6'
tBu
3
5
2'
N
2''
OMe
11g
3''
1.00 2.05 1.99 1.05 3.14
2.96
9.29
8.5 8.0 7.5 7.0 6.5 6.0 5.5 5.0 4.5 4.0 3.5 3.0 2.5 2.0 1.5 1.0 0.5 0.0

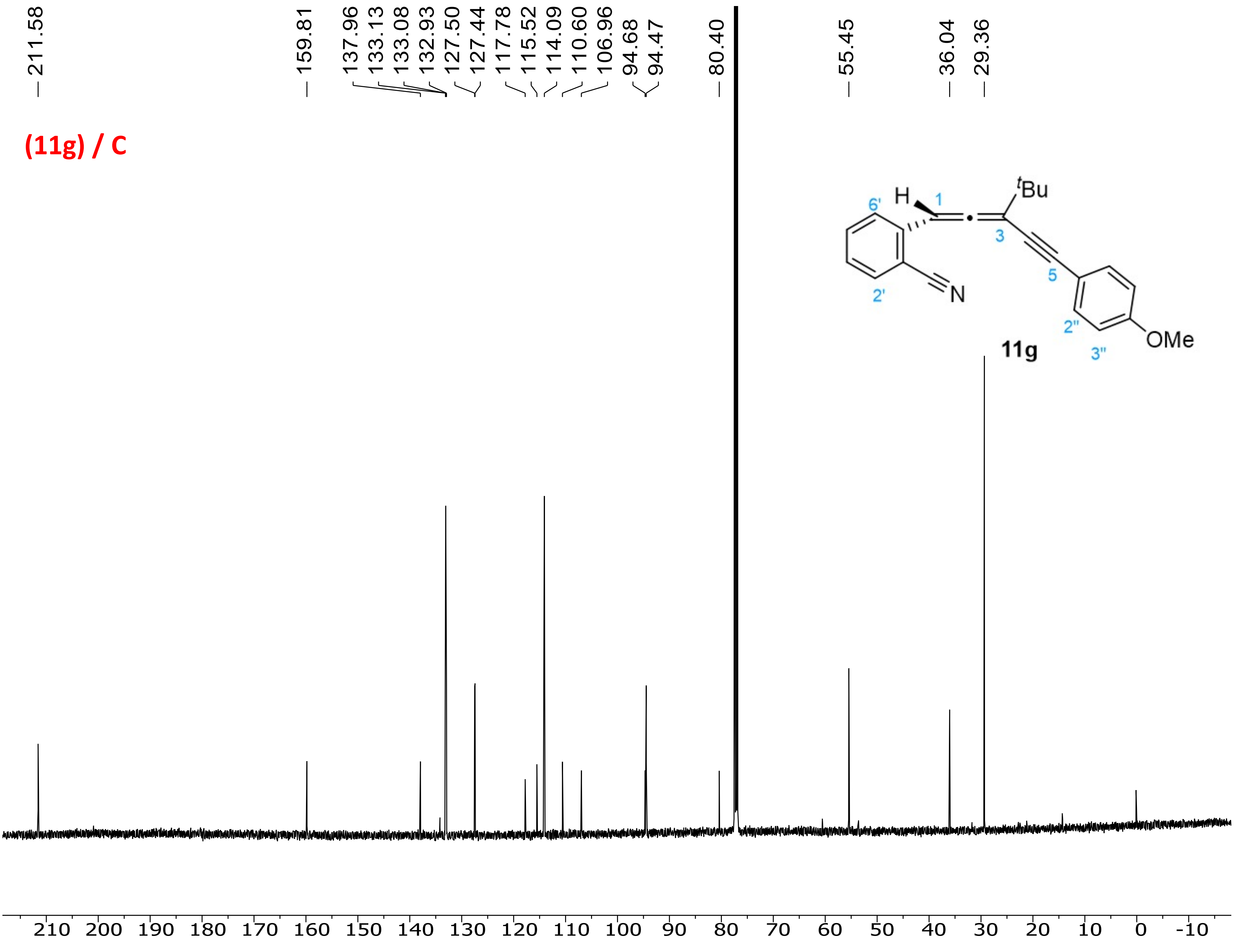
211.58
159.81
137.96
133.13
133.08
132.93
127.50
127.44
117.78
115.52
114.09
110.60
106.96
94.68
94.47
80.40
55.45
36.04
29.36
(11g) / C
H
1
6'
tBu
3
5
2'
N
2''
3''
OMe
11g
210 200 190 180 170 160 150 140 130 120 110 100 90 80 70 60 50 40 30 20 10 0 -10

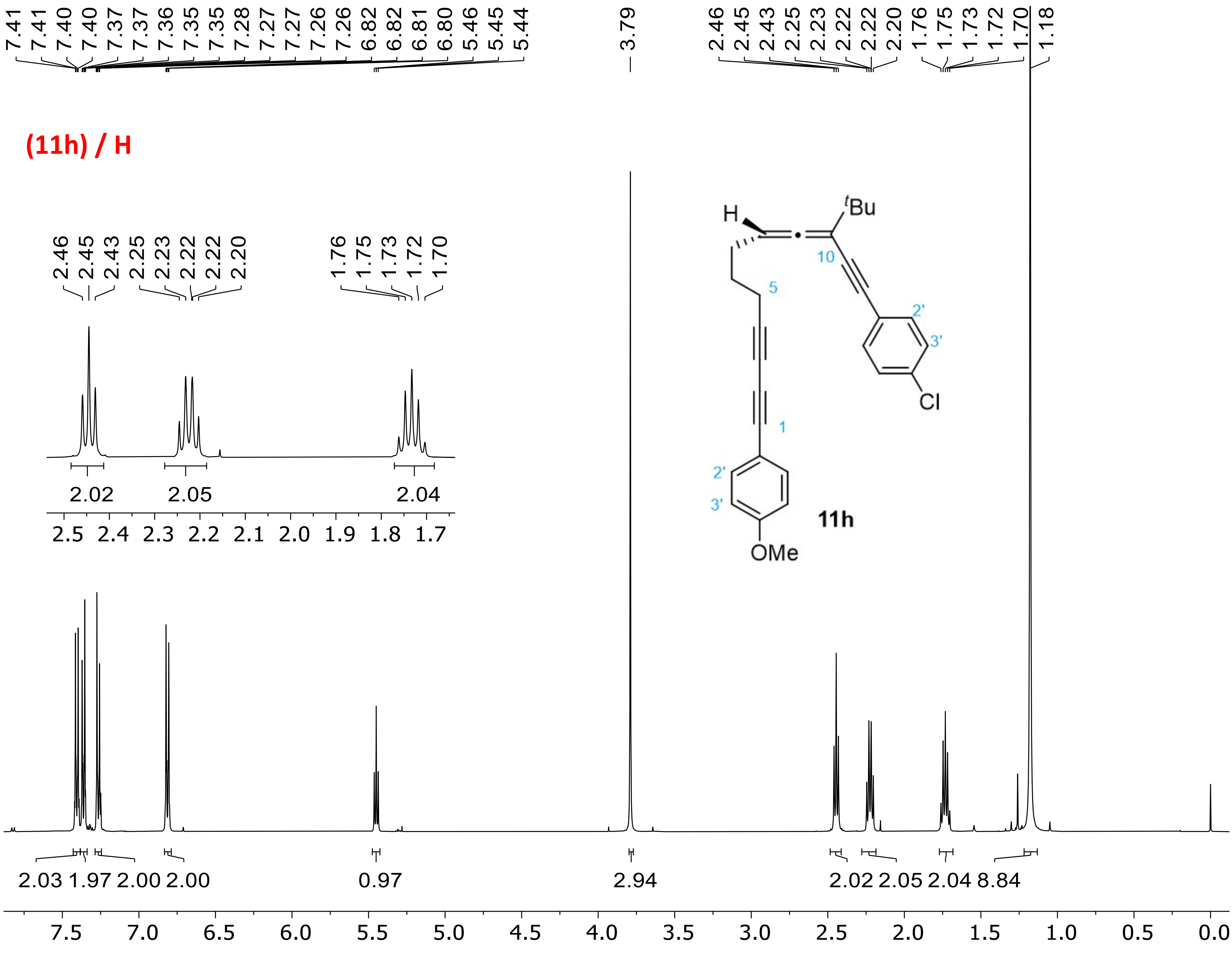
(11h) / H
7.41 7.41 7.40 7.40 7.37 7.37 7.36 7.35 7.35 7.28 7.27 7.27 7.26 7.26 6.82 6.82 6.81 6.80 5.46 5.45 5.44
3.79
2.46 2.45 2.43 2.25 2.23 2.22 2.22 2.20 1.76 1.75 1.73 1.72 1.70 1.18
H
tBu
10
5
2'
3'
Cl
1
2'
3'
OMe
11h
2.46 2.45 2.43 2.25 2.23 2.22 2.22 2.20
1.76 1.75 1.73 1.72 1.70
2.02 2.05 2.04
2.5 2.4 2.3 2.2 2.1 2.0 1.9 1.8 1.7
2.03 1.97 2.00 2.00 0.97 2.94 2.02 2.05 2.04 8.84
7.5 7.0 6.5 6.0 5.5 5.0 4.5 4.0 3.5 3.0 2.5 2.0 1.5 1.0 0.5 0.0

(11h) / C

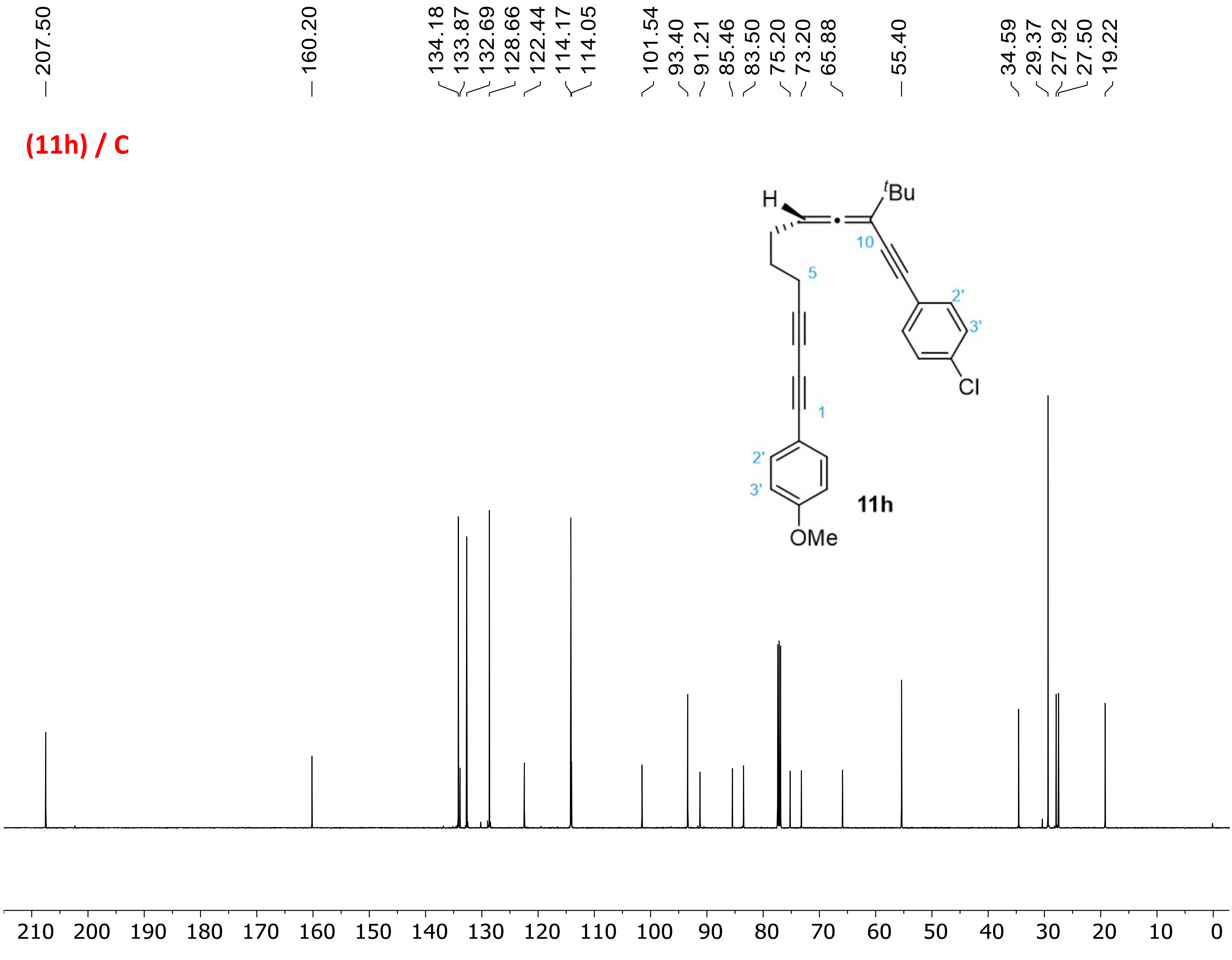
207.50
160.20
134.18
133.87
132.69
128.66
122.44
114.17
114.05
101.54
93.40
91.21
85.46
83.50
75.20
73.20
65.88
55.40
34.59
29.37
27.92
27.50
19.22
H
tBu
10
5
2'
3'
Cl
1
2'
3'
OMe
11h
210 200 190 180 170 160 150 140 130 120 110 100 90 80 70 60 50 40 30 20 10 0

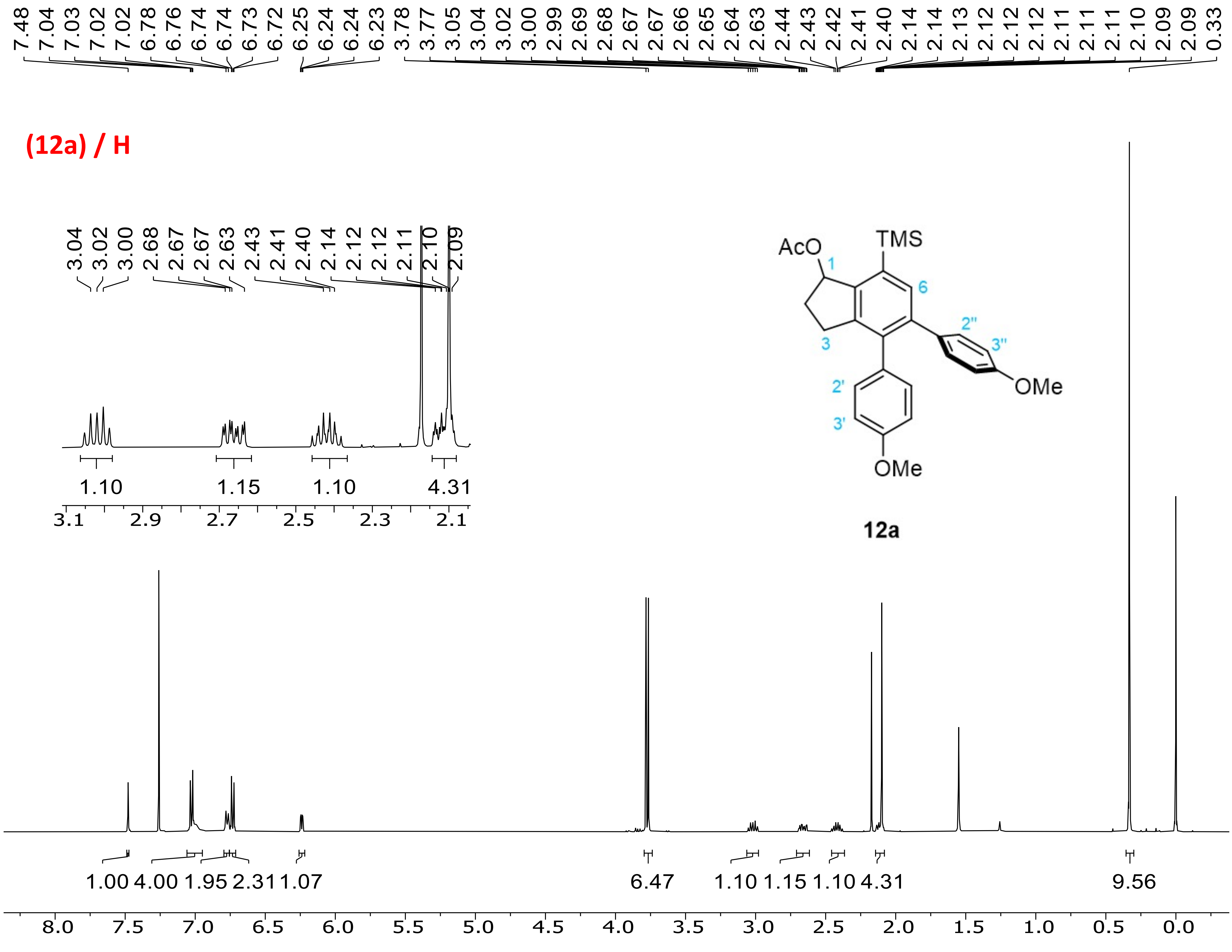
7.48 7.04 7.03 7.02 7.02 6.78 6.76 6.74 6.74 6.73 6.72 6.25 6.24 6.24 6.23 3.78 3.77 3.05 3.04 3.02 3.00 2.99 2.69 2.68 2.67 2.67 2.66 2.65 2.64 2.63 2.44 2.43 2.42 2.41 2.40 2.14 2.14 2.13 2.12 2.12 2.12 2.11 2.11 2.11 2.10 2.09 2.09 0.33
(12a) / H
3.04 3.02 3.00 2.68 2.67 2.67 2.63 2.43 2.41 2.40 2.14 2.12 2.12 2.11 2.10 2.09
1.10
1.15
1.10
4.31
3.1
2.9
2.7
2.5
2.3
2.1
AcO
TMS
1
6
2''
3''
3
OMe
2'
3'
OMe
12a
1.00 4.00 1.95 2.31 1.07
6.47
1.10 1.15 1.10 4.31
9.56
8.0 7.5 7.0 6.5 6.0 5.5 5.0 4.5 4.0 3.5 3.0 2.5 2.0 1.5 1.0 0.5 0.0

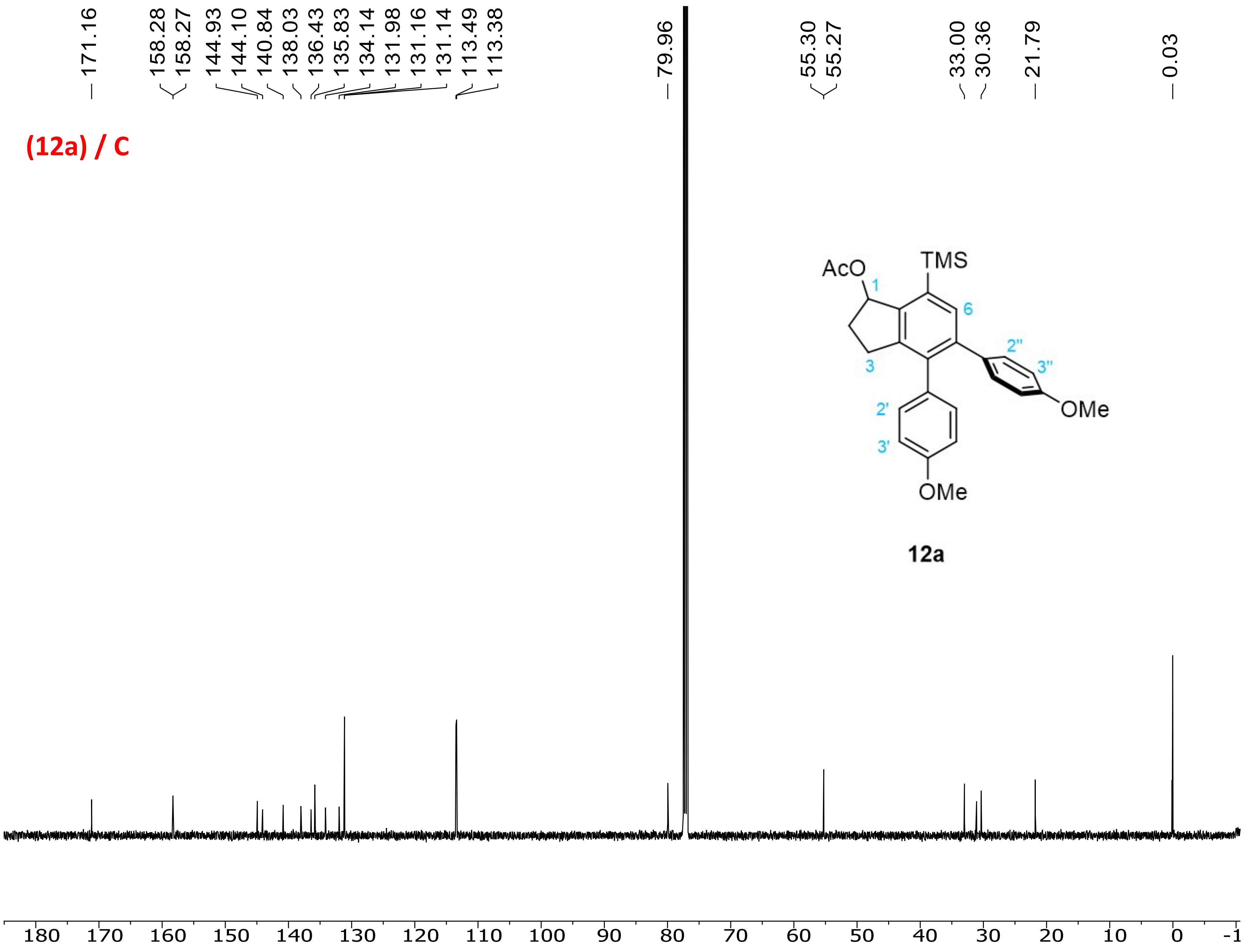
171.16
158.28
158.27
144.93
144.10
140.84
138.03
136.43
135.83
134.14
131.98
131.16
131.14
113.49
113.38
79.96
55.30
55.27
33.00
30.36
21.79
0.03
(12a) / C
AcO
TMS
1
6
2''
3''
3
2'
3'
OMe
OMe
12a
180
170
160
150
140
130
120
110
100
90
80
70
60
50
40
30
20
10
0
-1

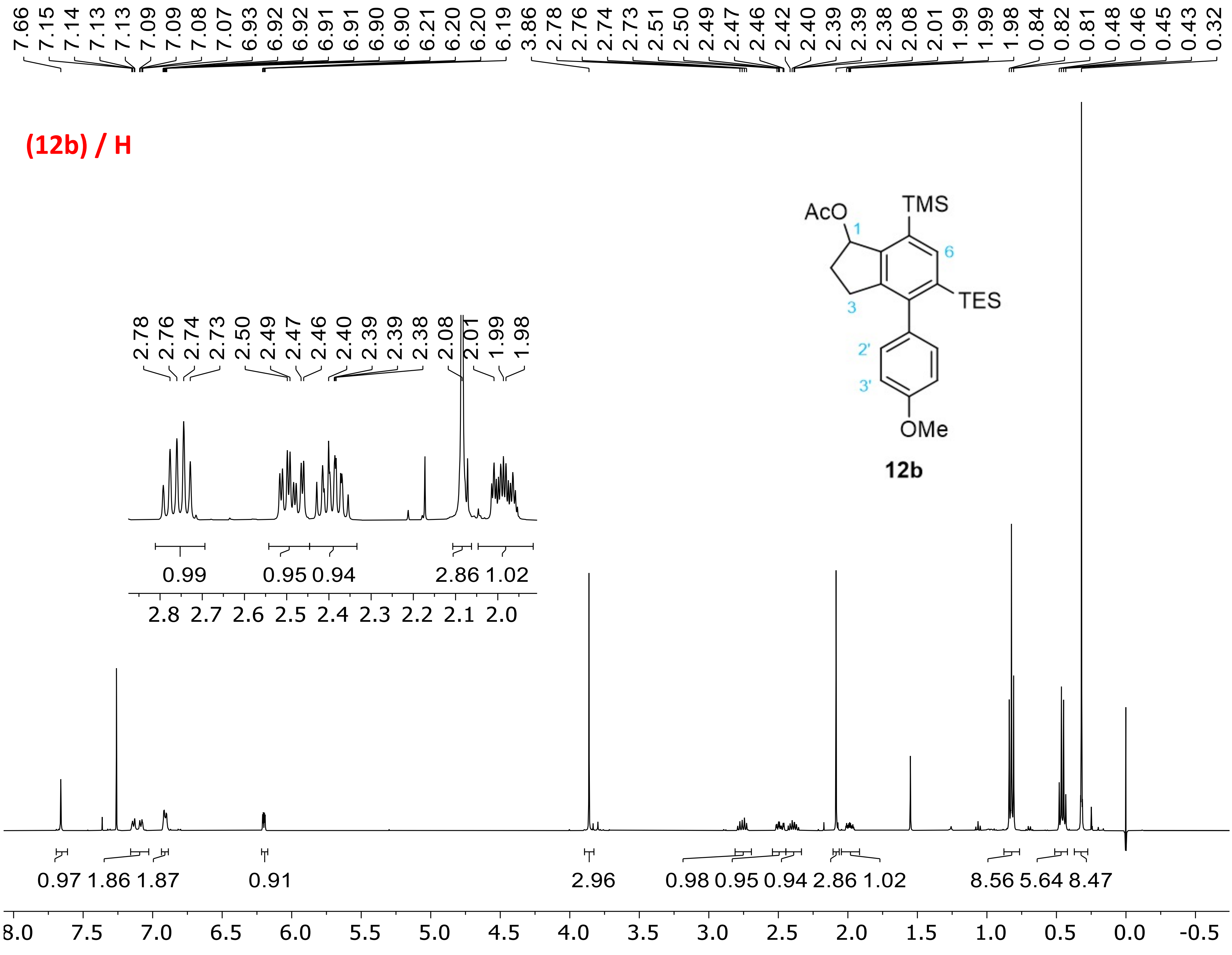
(12b) / H
7.66 7.15 7.14 7.13 7.13 7.09 7.09 7.08 7.07 6.93 6.92 6.92 6.91 6.91 6.90 6.90 6.21 6.20 6.20 6.19 3.86 2.78 2.76 2.74 2.73 2.51 2.50 2.49 2.47 2.46 2.42 2.40 2.39 2.39 2.38 2.08 2.01 1.99 1.99 1.98 0.84 0.82 0.81 0.48 0.46 0.45 0.43 0.32
AcO
TMS
TES
OMe
1
3
6
2'
3'
12b
2.78 2.76 2.74 2.73 2.50 2.49 2.47 2.46 2.40 2.39 2.39 2.38 2.08 2.01 1.99 1.98
0.99 0.95 0.94 2.86 1.02
2.8 2.7 2.6 2.5 2.4 2.3 2.2 2.1 2.0
0.97 1.86 1.87 0.91 2.96 0.98 0.95 0.94 2.86 1.02 8.56 5.64 8.47
8.0 7.5 7.0 6.5 6.0 5.5 5.0 4.5 4.0 3.5 3.0 2.5 2.0 1.5 1.0 0.5 0.0 -0.5

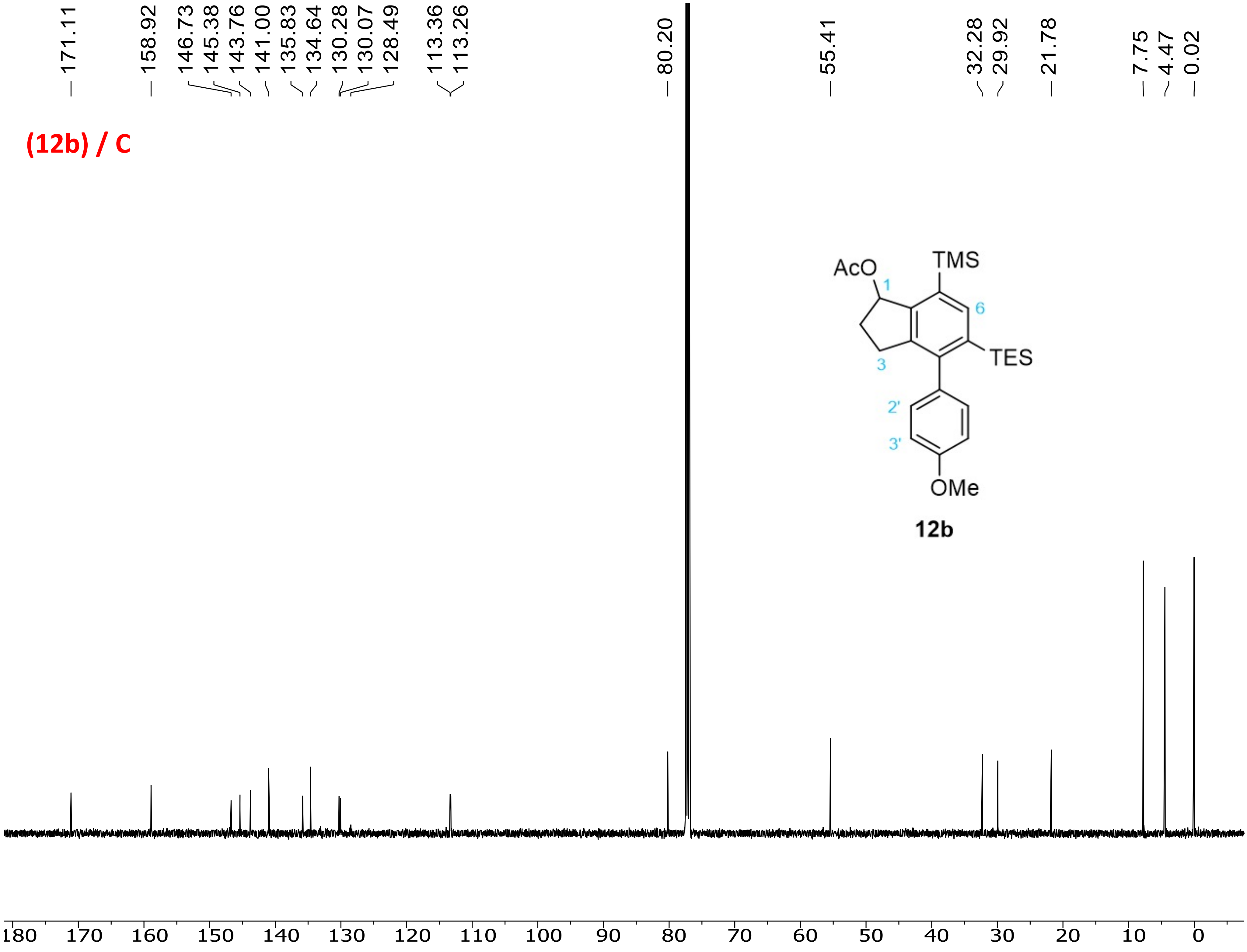
171.11
158.92
146.73
145.38
143.76
141.00
135.83
134.64
130.28
130.07
128.49
113.36
113.26
80.20
55.41
32.28
29.92
21.78
7.75
4.47
0.02
(12b) / C
AcO
TMS
1
6
TES
3
2'
3'
OMe
12b
180 170 160 150 140 130 120 110 100 90 80 70 60 50 40 30 20 10 0

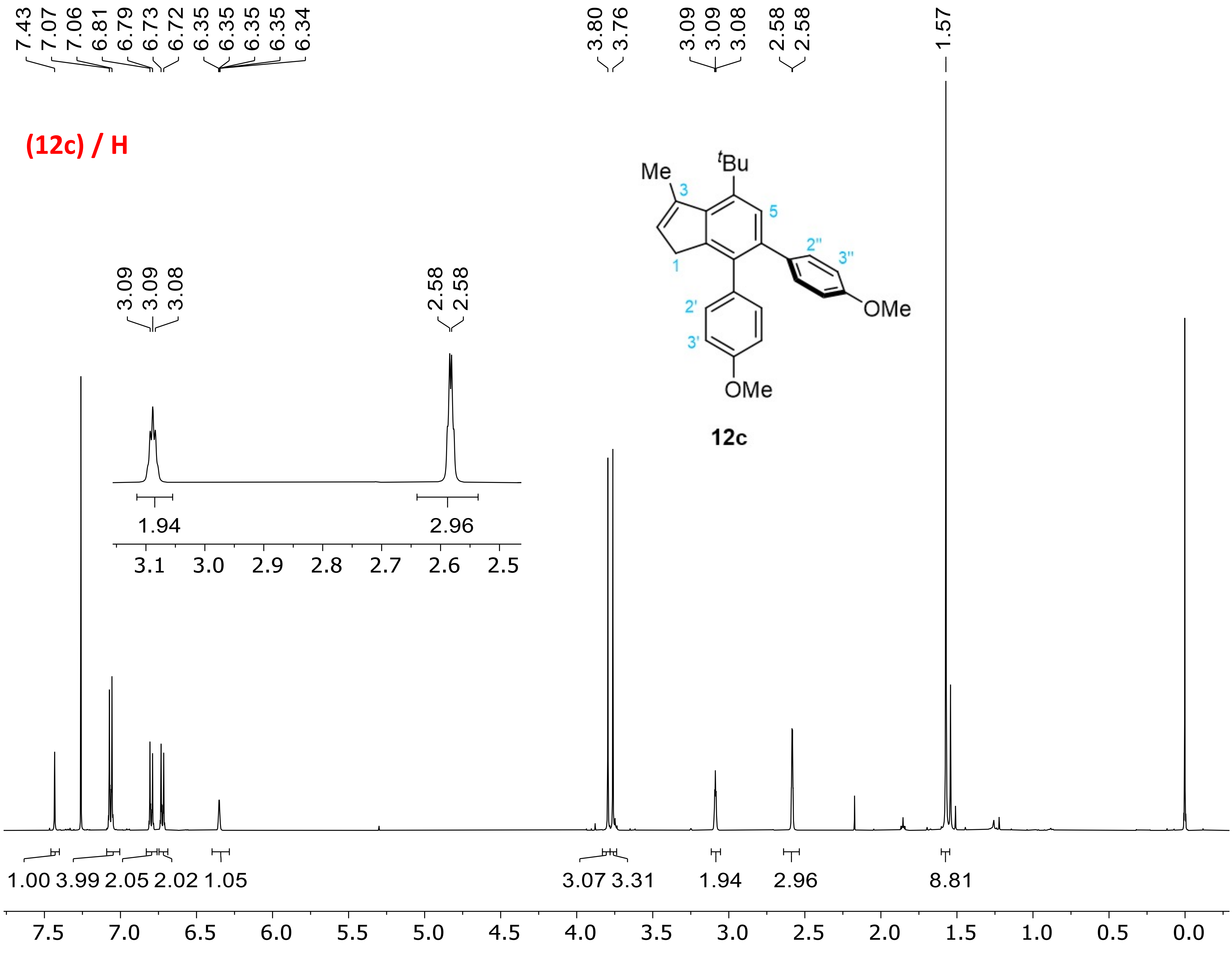
7.43
7.07
7.06
6.81
6.79
6.73
6.72
6.35
6.35
6.35
6.35
6.34
3.80
3.76
3.09
3.09
3.08
2.58
2.58
1.57
(12c) / H
Me
tBu
3
5
2''
3''
1
2'
3'
OMe
OMe
12c
1.94
2.96
3.1
3.0
2.9
2.8
2.7
2.6
2.5
1.00
3.99
2.05
2.02
1.05
3.07
3.31
1.94
2.96
8.81
7.5
7.0
6.5
6.0
5.5
5.0
4.5
4.0
3.5
3.0
2.5
2.0
1.5
1.0
0.5
0.0

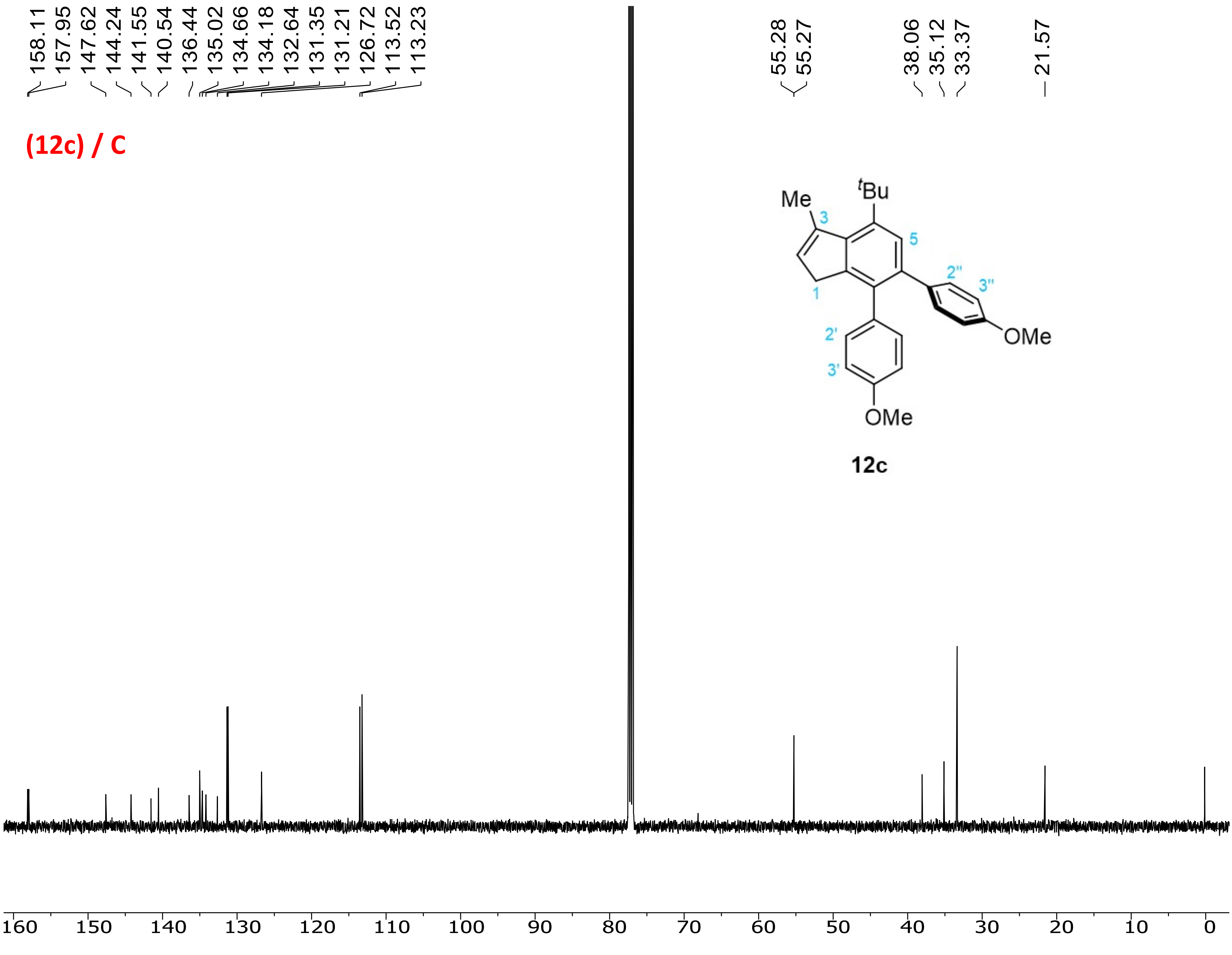
158.11
157.95
147.62
144.24
141.55
140.54
136.44
135.02
134.66
134.18
132.64
131.35
131.21
126.72
113.52
113.23
55.28
55.27
38.06
35.12
33.37
21.57
(12c) / C
Me
tBu
3
5
1
2''
3''
2'
3'
OMe
OMe
12c
160
150
140
130
120
110
100
90
80
70
60
50
40
30
20
10
0

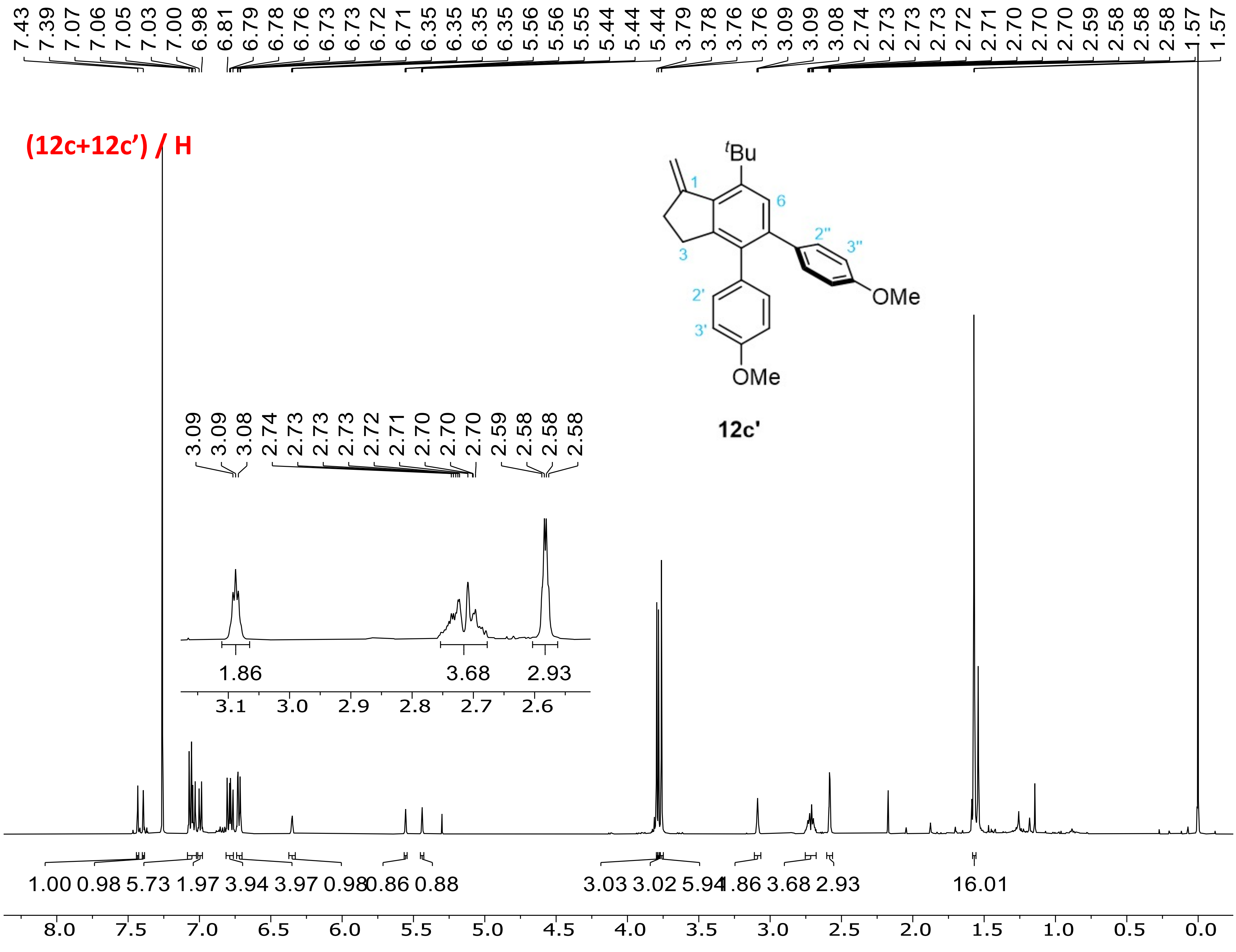

7.43 7.39 7.07 7.06 7.05 7.03 7.00 6.98 6.81 6.79 6.78 6.76 6.73 6.73 6.72 6.71 6.35 6.35 6.35 6.35 5.56 5.56 5.55 5.44 5.44 5.44 3.79 3.78 3.76 3.76 3.09 3.09 3.08 2.74 2.73 2.73 2.73 2.72 2.71 2.70 2.70 2.70 2.59 2.58 2.58 2.58 1.57 1.57
(12c+12c') / H
tBu
1
6
2''
3''
3
2'
3'
OMe
OMe
12c'
3.09 3.09 3.08 2.74 2.73 2.73 2.73 2.72 2.71 2.70 2.70 2.70 2.59 2.58 2.58 2.58
1.86
3.68
2.93
3.1 3.0 2.9 2.8 2.7 2.6
1.00 0.98 5.73 1.97 3.94 3.97 0.98 0.86 0.88
3.03 3.02 5.94 1.86 3.68 2.93
16.01
8.0 7.5 7.0 6.5 6.0 5.5 5.0 4.5 4.0 3.5 3.0 2.5 2.0 1.5 1.0 0.5 0.0

(12c+12c') / C

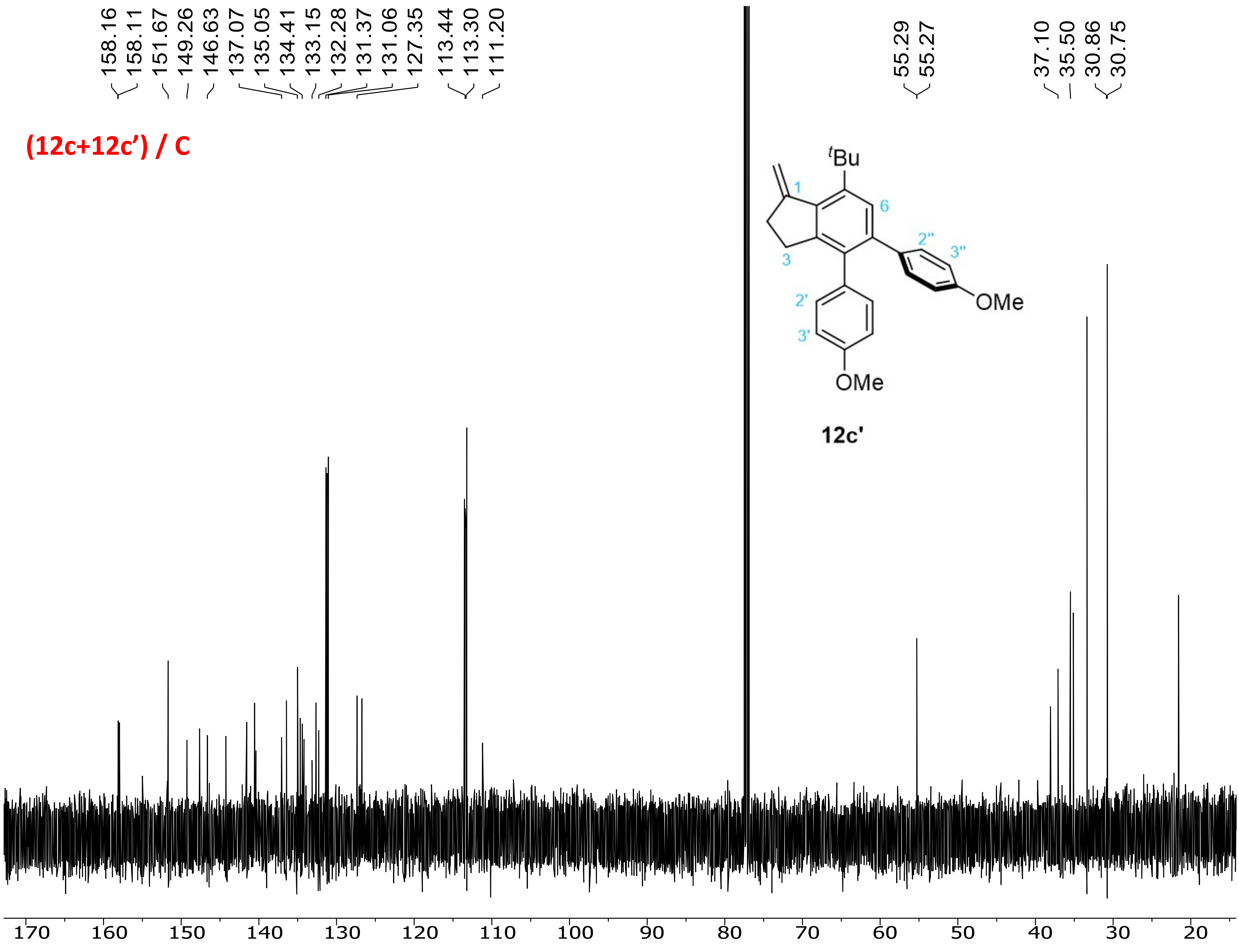
158.16
158.11
151.67
149.26
146.63
137.07
135.05
134.41
133.15
132.28
131.37
131.06
127.35
113.44
113.30
111.20
55.29
55.27
37.10
35.50
30.86
30.75
tBu
1
6
2''
3''
3
2'
3'
OMe
OMe
170
160
150
140
130
120
110
100
90
80
70
60
50
40
30
20

12c'

(12d) / H

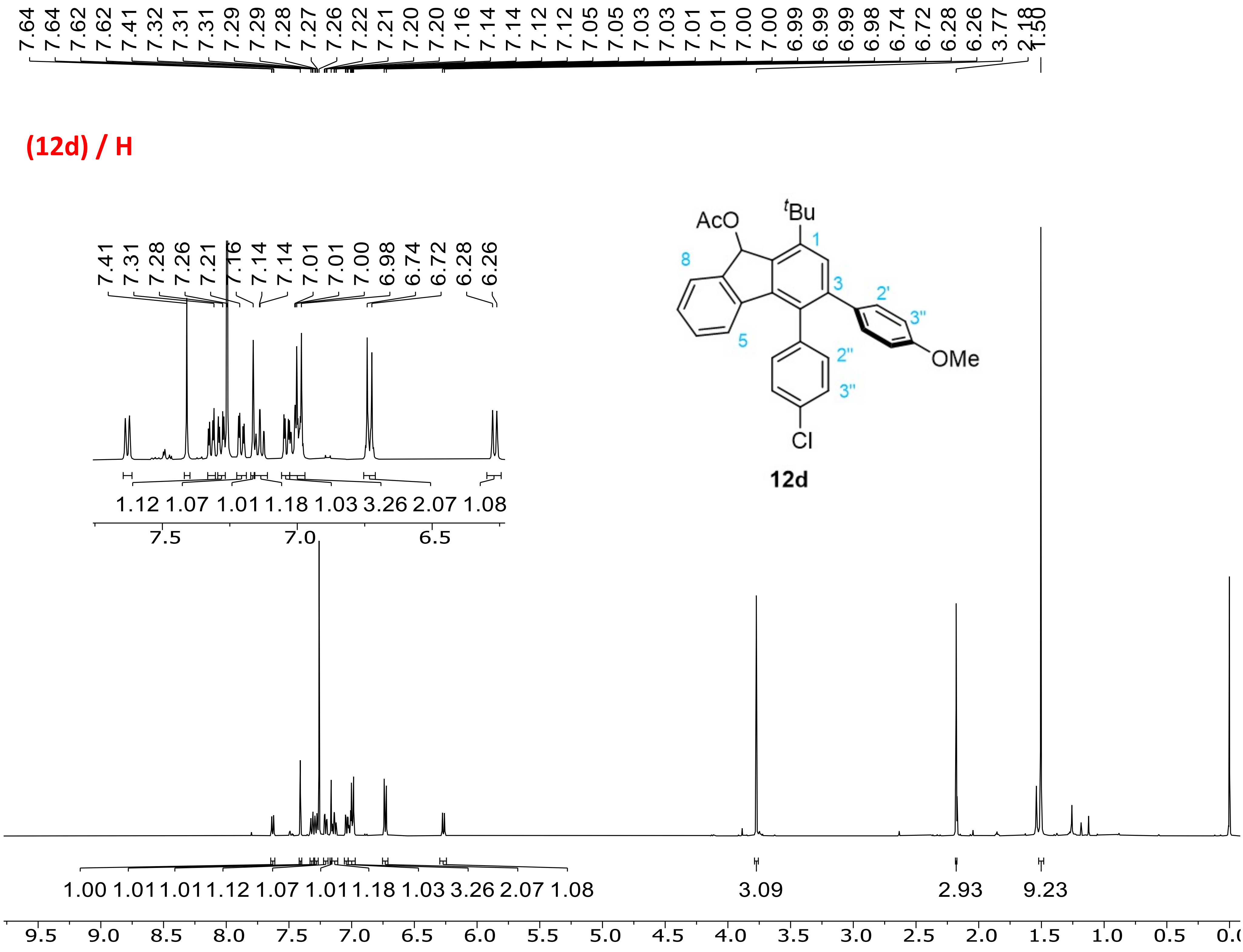
7.64 7.64 7.62 7.62 7.41 7.32 7.31 7.31 7.29 7.29 7.28 7.27 7.26 7.22 7.21 7.20 7.20 7.16 7.14 7.14 7.12 7.12 7.05 7.05 7.03 7.03 7.01 7.01 7.00 7.00 6.99 6.99 6.99 6.98 6.74 6.72 6.28 6.26 3.77 2.18 1.50
7.41 7.31 7.28 7.26 7.21 7.16 7.14 7.14 7.01 7.01 7.00 6.98 6.74 6.72 6.28 6.26
1.12 1.07 1.01 1.18 1.03 3.26 2.07 1.08
7.5 7.0 6.5
1.00 1.01 1.01 1.12 1.07 1.01 1.18 1.03 3.26 2.07 1.08 3.09 2.93 9.23
9.5 9.0 8.5 8.0 7.5 7.0 6.5 6.0 5.5 5.0 4.5 4.0 3.5 3.0 2.5 2.0 1.5 1.0 0.5 0.0
AcO
^{t}Bu
1
8
3
2'
3"
5
2"
OMe
3"
Cl

12d

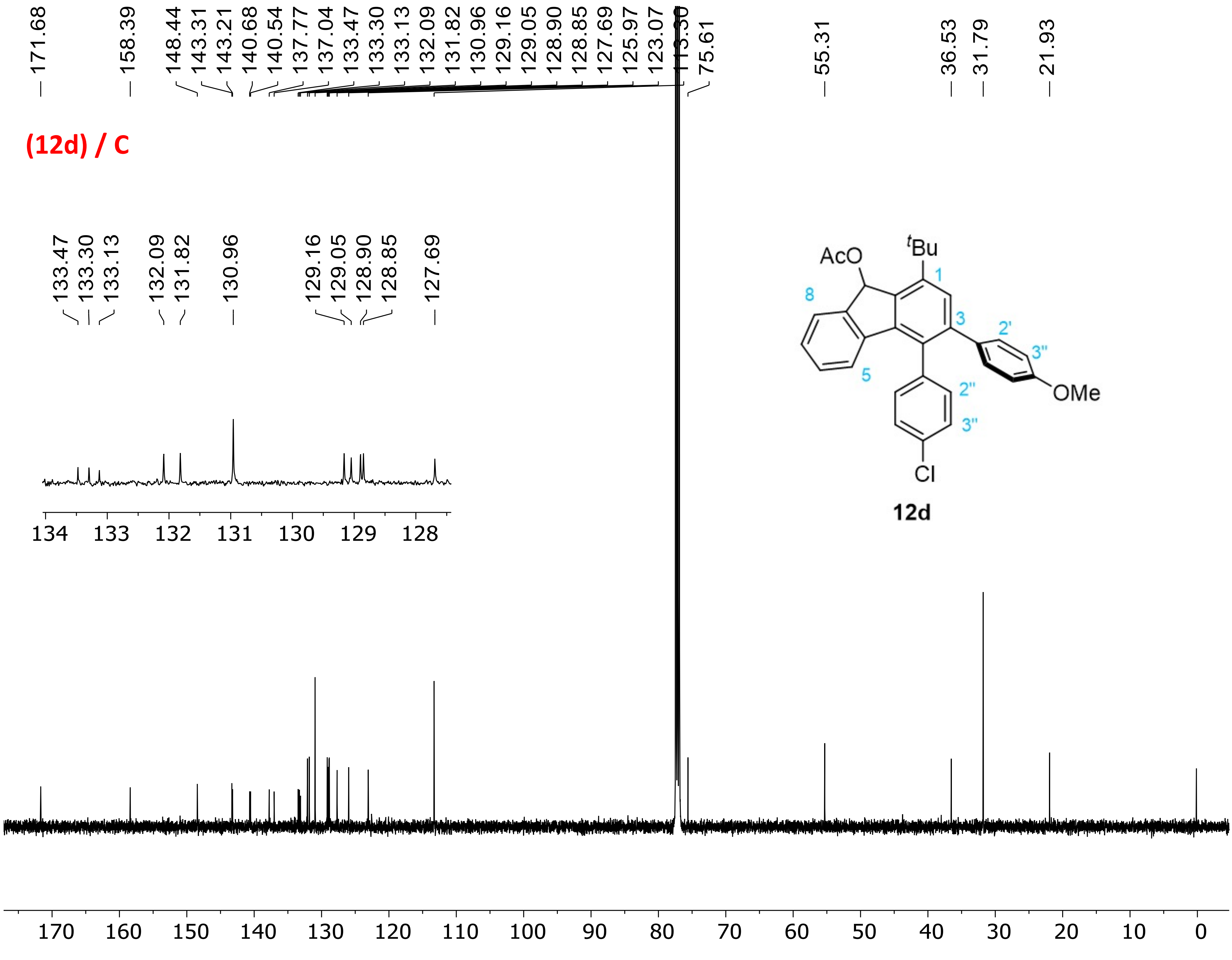
(12d) / C
171.68
158.39
148.44
143.31
143.21
140.68
140.54
137.77
137.04
133.47
133.30
133.13
132.09
131.82
130.96
129.16
129.05
128.90
128.85
127.69
125.97
123.07
113.30
75.61
55.31
36.53
31.79
21.93
AcO
^{t}Bu
OMe
Cl
1
3
5
8
2'
3''
2''
3''
12d
134 133 132 131 130 129 128
170 160 150 140 130 120 110 100 90 80 70 60 50 40 30 20 10 0

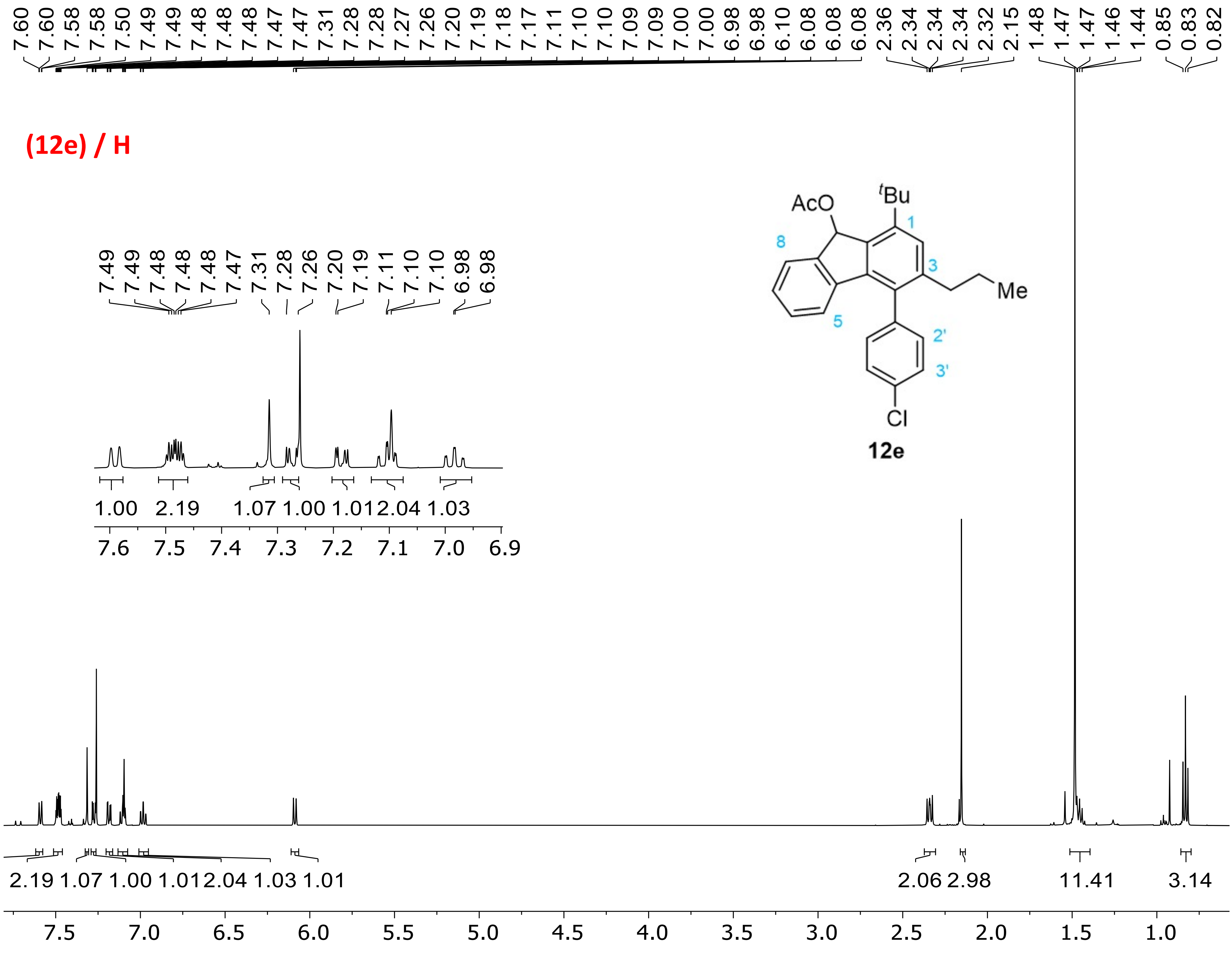
7.60 7.60 7.58 7.58 7.50 7.49 7.49 7.48 7.48 7.48 7.47 7.47 7.31 7.28 7.28 7.27 7.26 7.20 7.19 7.18 7.17 7.11 7.10 7.10 7.09 7.09 7.00 7.00 6.98 6.98 6.10 6.08 6.08 6.08 2.36 2.34 2.34 2.34 2.32 2.15 1.48 1.47 1.47 1.46 1.44 0.85 0.83 0.82
(12e) / H
7.49 7.49 7.48 7.48 7.48 7.47 7.31 7.28 7.26 7.20 7.19 7.11 7.10 7.10 6.98 6.98
1.00 2.19 1.07 1.00 1.01 2.04 1.03
7.6 7.5 7.4 7.3 7.2 7.1 7.0 6.9
AcO
tBu
1
8
3
Me
5
2'
3'
Cl
12e
2.19 1.07 1.00 1.01 2.04 1.03 1.01 2.06 2.98 11.41 3.14
7.5 7.0 6.5 6.0 5.5 5.0 4.5 4.0 3.5 3.0 2.5 2.0 1.5 1.0

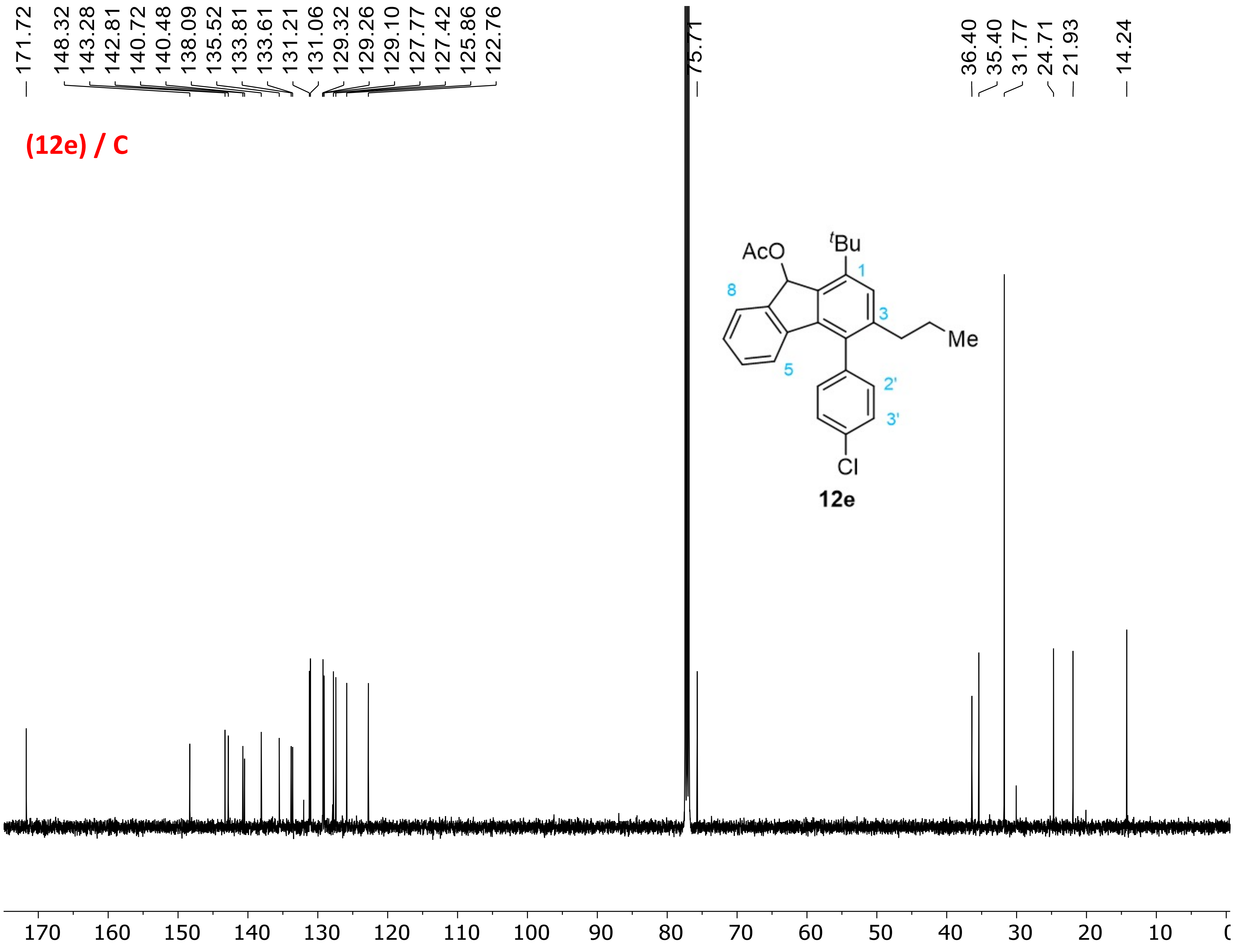
171.72
148.32
143.28
142.81
140.72
140.48
138.09
135.52
133.81
133.61
131.21
131.06
129.32
129.26
129.10
127.77
127.42
125.86
122.76
75.71
36.40
35.40
31.77
24.71
21.93
14.24
(12e) / C
AcO
tBu
1
3
8
5
Me
2'
3'
Cl
12e
170
160
150
140
130
120
110
100
90
80
70
60
50
40
30
20
10

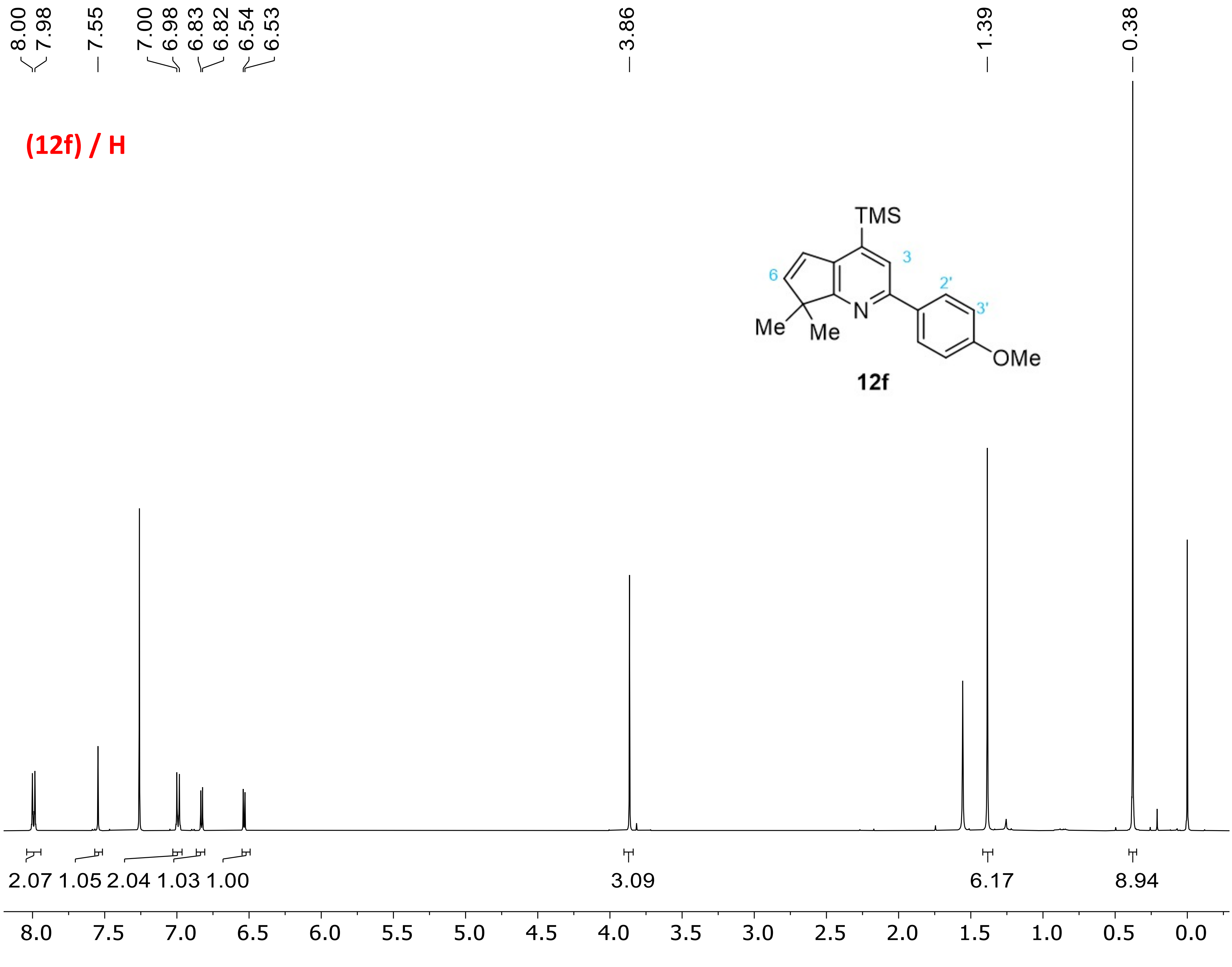
8.00
7.98
7.55
7.00
6.98
6.83
6.82
6.54
6.53
3.86
1.39
0.38
(12f) / H
TMS
3
6
2'
3'
N
Me
Me
OMe
12f
2.07
1.05
2.04
1.03
1.00
3.09
6.17
8.94
8.0
7.5
7.0
6.5
6.0
5.5
5.0
4.5
4.0
3.5
3.0
2.5
2.0
1.5
1.0
0.5
0.0

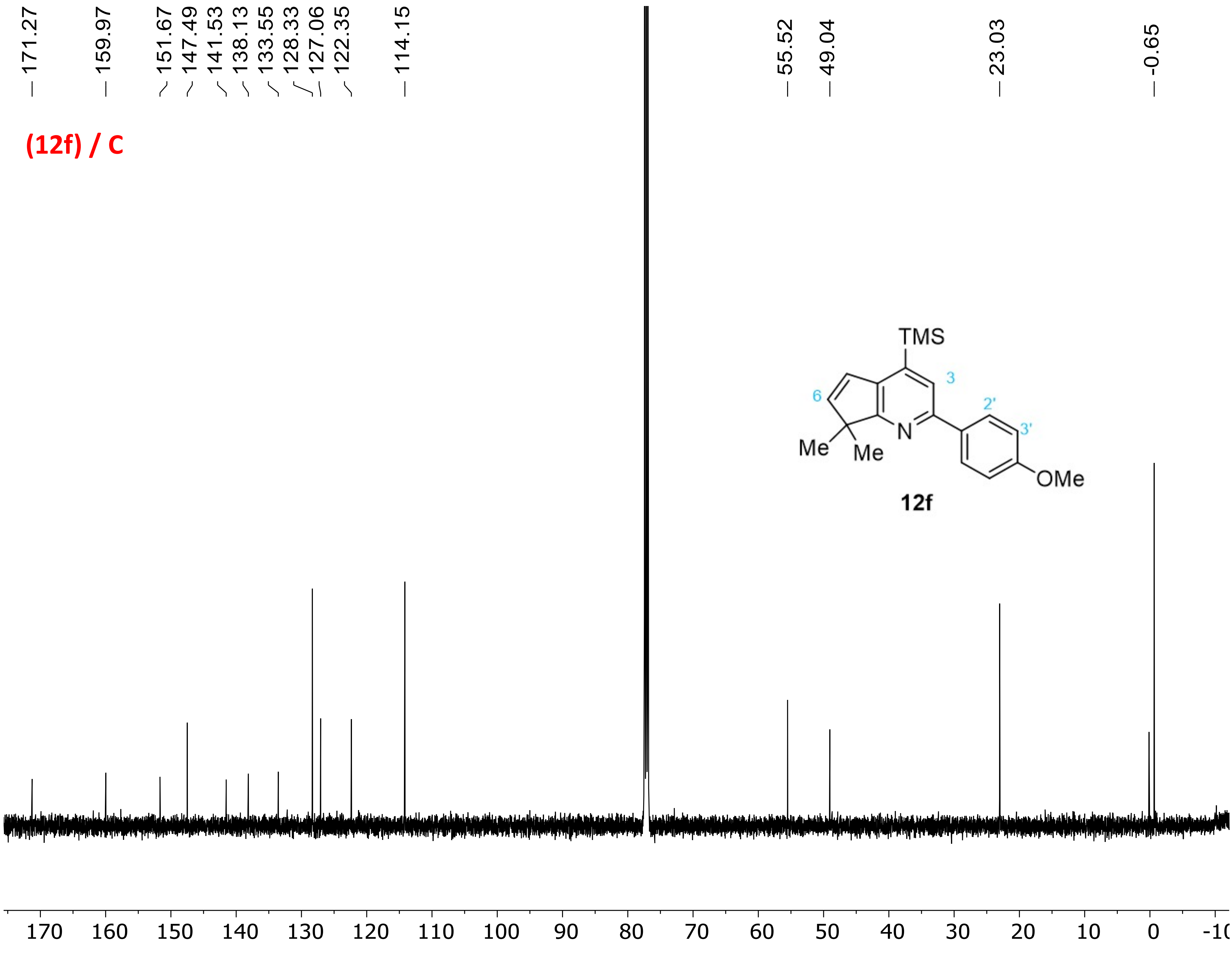
171.27
159.97
151.67
147.49
141.53
138.13
133.55
128.33
127.06
122.35
114.15
55.52
49.04
23.03
-0.65
(12f) / C
TMS
3
6
2'
3'
N
Me
Me
OMe
12f
170 160 150 140 130 120 110 100 90 80 70 60 50 40 30 20 10 0

(12g) / H

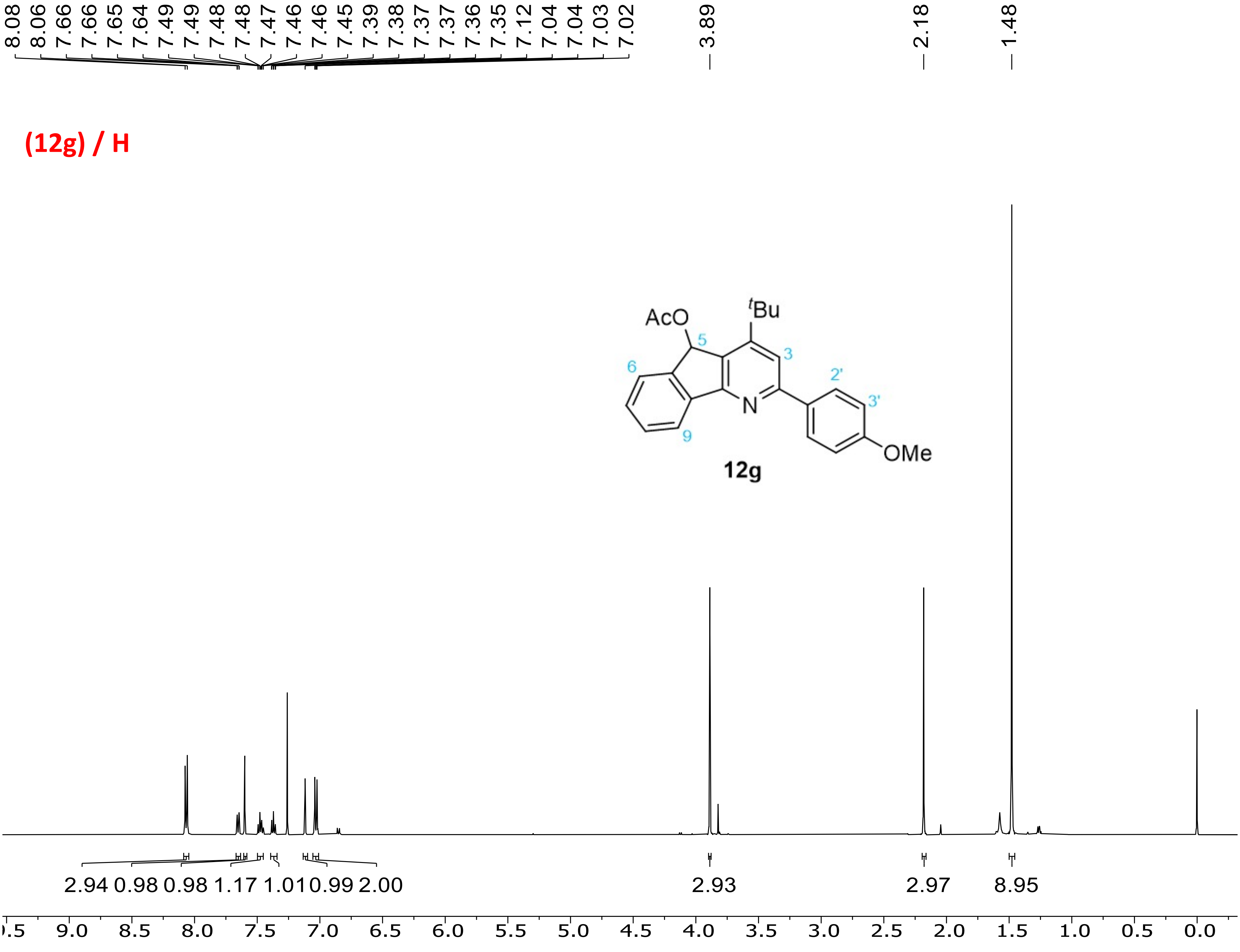
8.08
8.06
7.66
7.66
7.65
7.64
7.49
7.49
7.48
7.48
7.47
7.46
7.46
7.45
7.39
7.38
7.37
7.37
7.36
7.35
7.12
7.04
7.04
7.03
7.02
3.89
2.18
1.48
AcO
tBu
5
3
6
2'
3'
N
9
OMe
12g
2.94
0.98
0.98
1.17
1.01
0.99
2.00
2.93
2.97
8.95
9.0
8.5
8.0
7.5
7.0
6.5
6.0
5.5
5.0
4.5
4.0
3.5
3.0
2.5
2.0
1.5
1.0
0.5
0.0

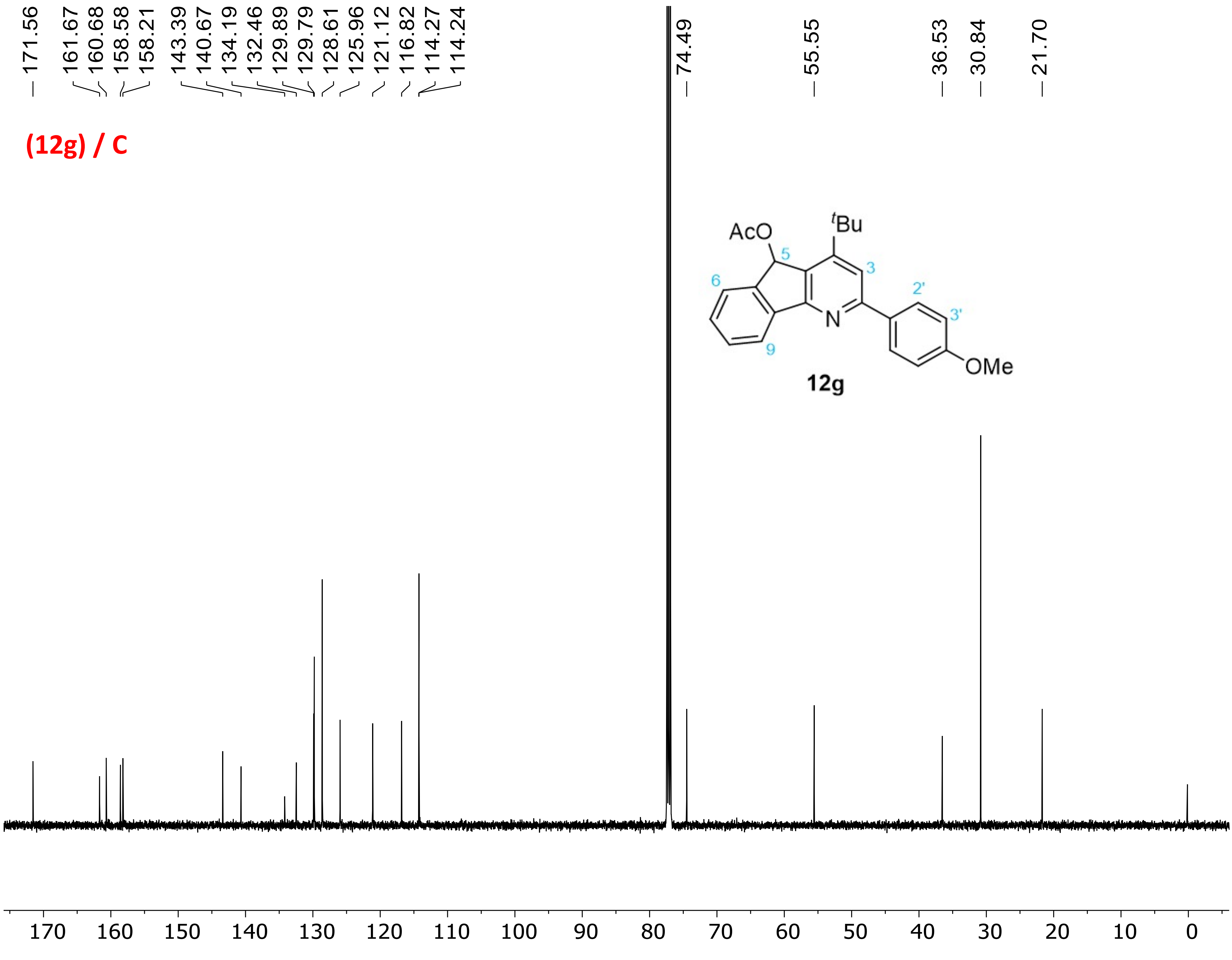
171.56
161.67
160.68
158.58
158.21
143.39
140.67
134.19
132.46
129.89
129.79
128.61
125.96
121.12
116.82
114.27
114.24
74.49
55.55
36.53
30.84
21.70
(12g) / C
AcO
^{t}Bu
5
3
6
2'
3'
N
9
OMe
12g
170 160 150 140 130 120 110 100 90 80 70 60 50 40 30 20 10 0

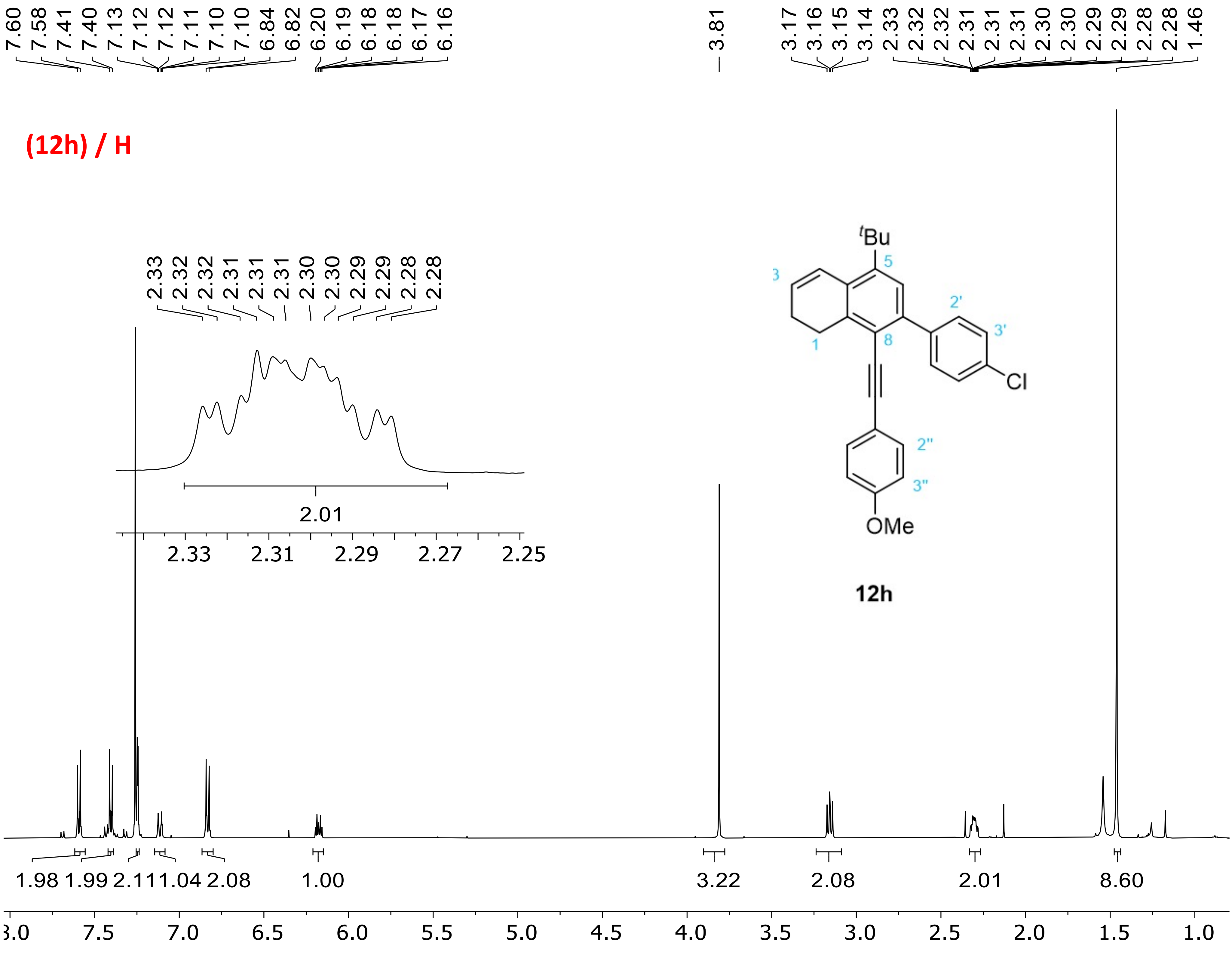
7.60 7.58 7.41 7.40 7.13 7.12 7.12 7.11 7.10 7.10 6.84 6.82 6.20 6.19 6.18 6.18 6.17 6.16
3.81
3.17 3.16 3.15 3.14 2.33 2.32 2.32 2.31 2.31 2.31 2.30 2.30 2.29 2.29 2.28 2.28 1.46
(12h) / H
2.33 2.32 2.32 2.31 2.31 2.31 2.30 2.30 2.29 2.29 2.28 2.28
2.01
2.33 2.31 2.29 2.27 2.25
tBu
3
5
2'
3'
1
8
Cl
2''
3''
OMe
12h
1.98 1.99 2.11 1.04 2.08 1.00 3.22 2.08 2.01 8.60
8.0 7.5 7.0 6.5 6.0 5.5 5.0 4.5 4.0 3.5 3.0 2.5 2.0 1.5 1.0

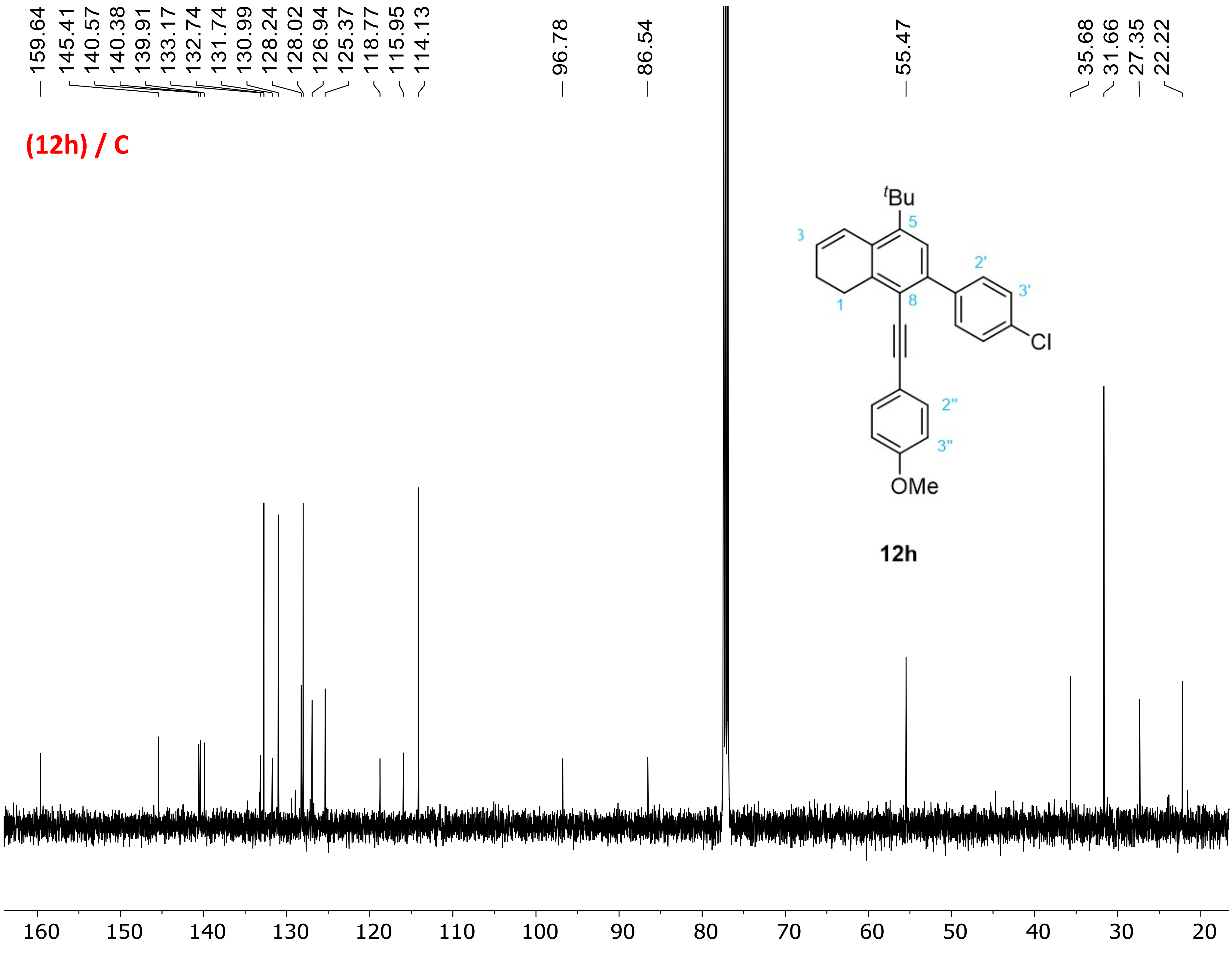
159.64
145.41
140.57
140.38
139.91
133.17
132.74
131.74
130.99
128.24
128.02
126.94
125.37
118.77
115.95
114.13
96.78
86.54
55.47
35.68
31.66
27.35
22.22
(12h) / C
tBu
5
3
2'
3'
8
1
Cl
2''
3''
OMe
12h
160
150
140
130
120
110
100
90
80
70
60
50
40
30
20

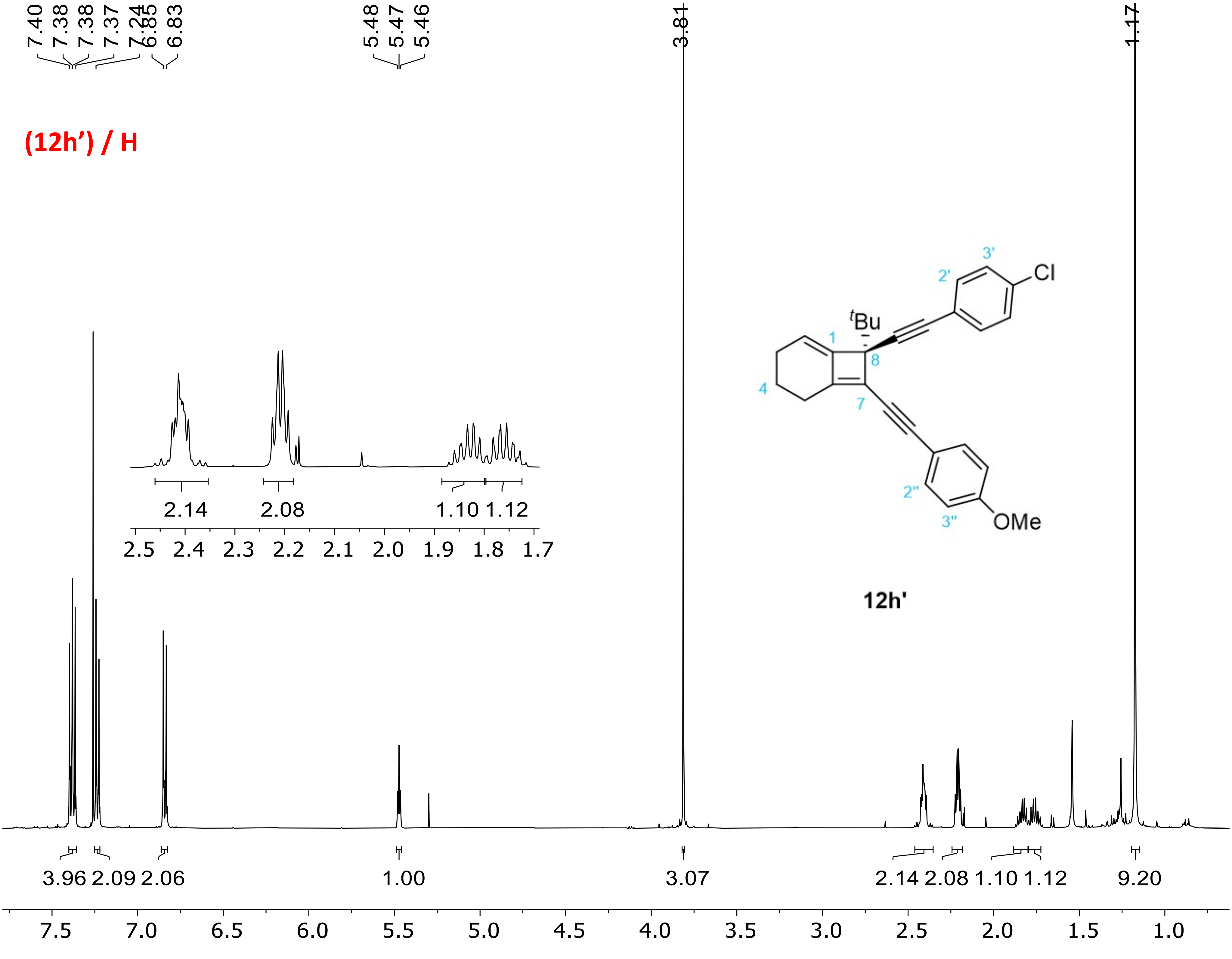
7.40 7.38 7.38 7.37 7.24 6.85 6.83
5.48 5.47 5.46
3.81
1.17
(12h') / H
2.14 2.08 1.10 1.12
2.5 2.4 2.3 2.2 2.1 2.0 1.9 1.8 1.7
tBu
Cl
OMe
1
8
4
7
2'
3'
2''
3''
12h'
3.96 2.09 2.06 1.00 3.07 2.14 2.08 1.10 1.12 9.20
7.5 7.0 6.5 6.0 5.5 5.0 4.5 4.0 3.5 3.0 2.5 2.0 1.5 1.0

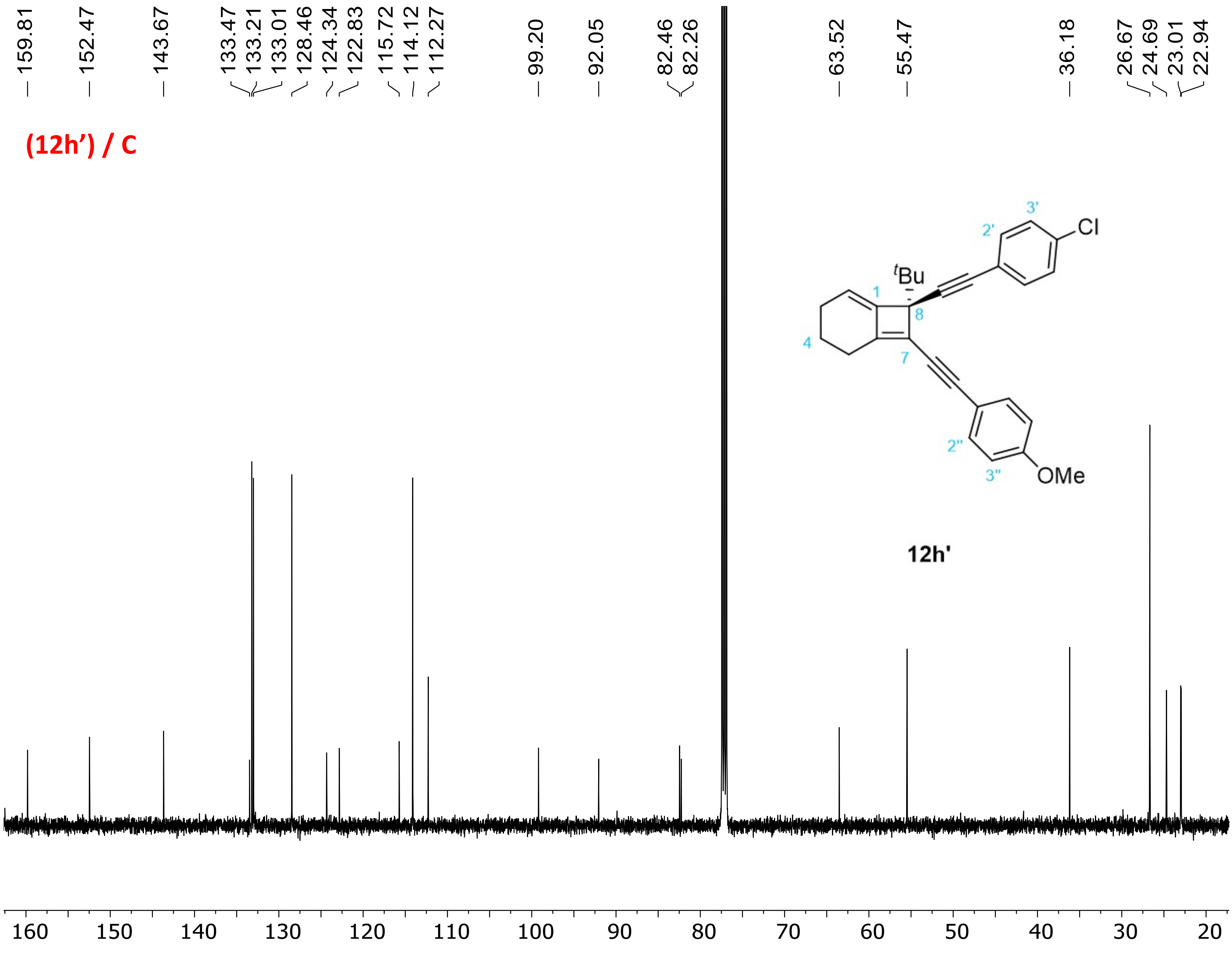
159.81
152.47
143.67
133.47
133.21
133.01
128.46
124.34
122.83
115.72
114.12
112.27
99.20
92.05
82.46
82.26
63.52
55.47
36.18
26.67
24.69
23.01
22.94
(12h') / C
tBu
Cl
OMe
1
2'
3'
4
7
8
2''
3''
12h'
160
150
140
130
120
110
100
90
80
70
60
50
40
30
20

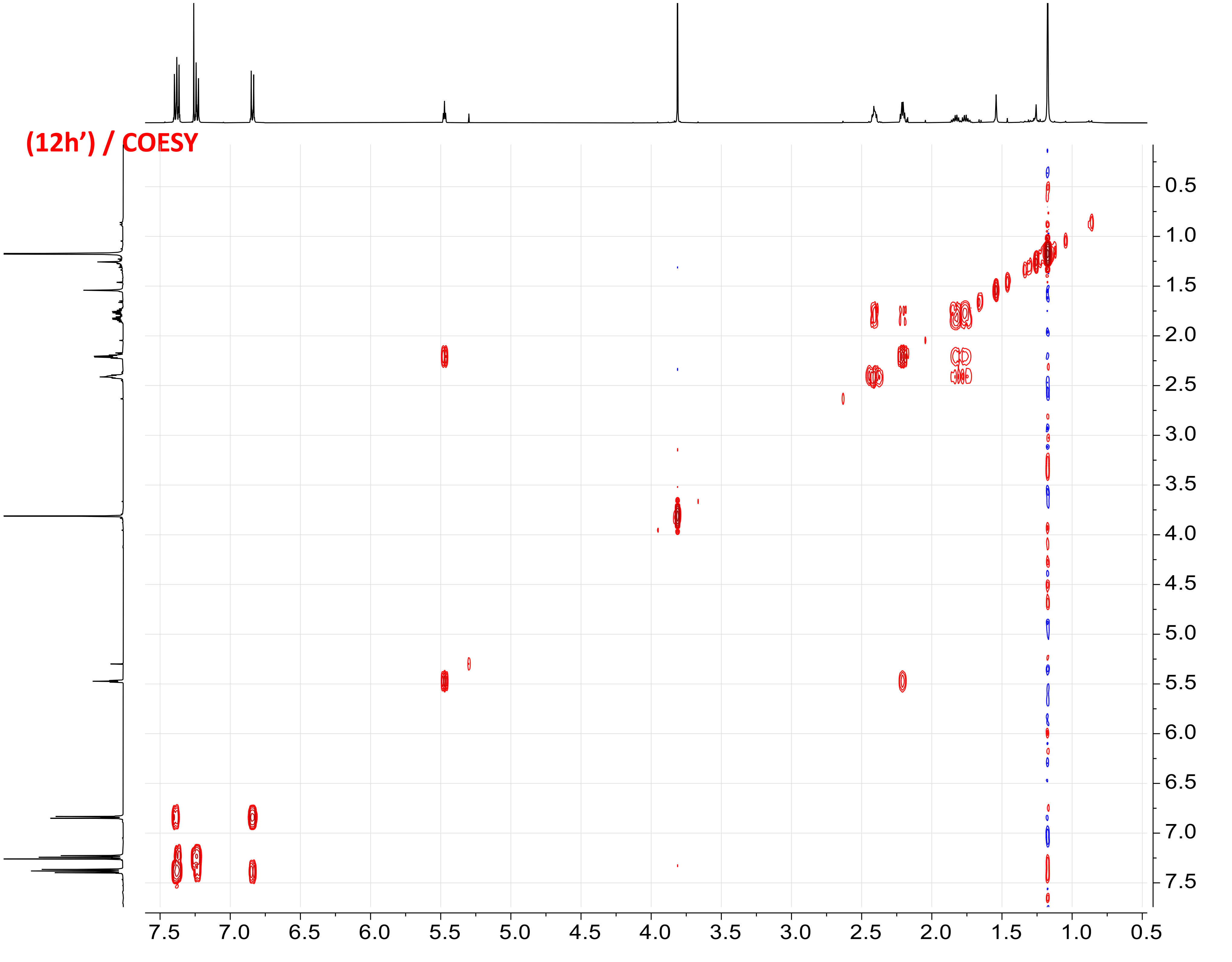
(12h') / COESY
7.5
7.0
6.5
6.0
5.5
5.0
4.5
4.0
3.5
3.0
2.5
2.0
1.5
1.0
0.5

(12h') / HSQC

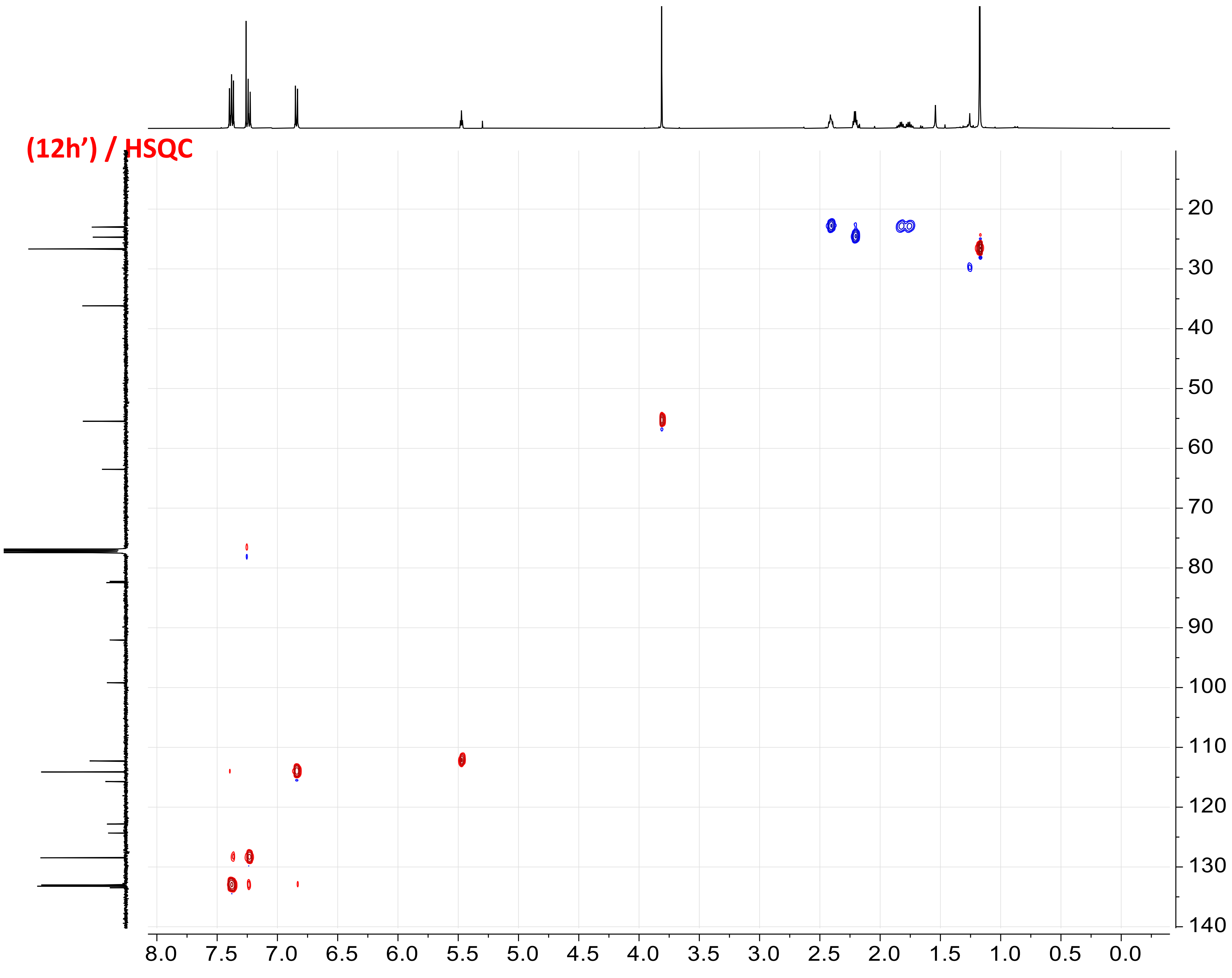
20
30
40
50
60
70
80
90
100
110
120
130
140
8.0 7.5 7.0 6.5 6.0 5.5 5.0 4.5 4.0 3.5 3.0 2.5 2.0 1.5 1.0 0.5 0.0

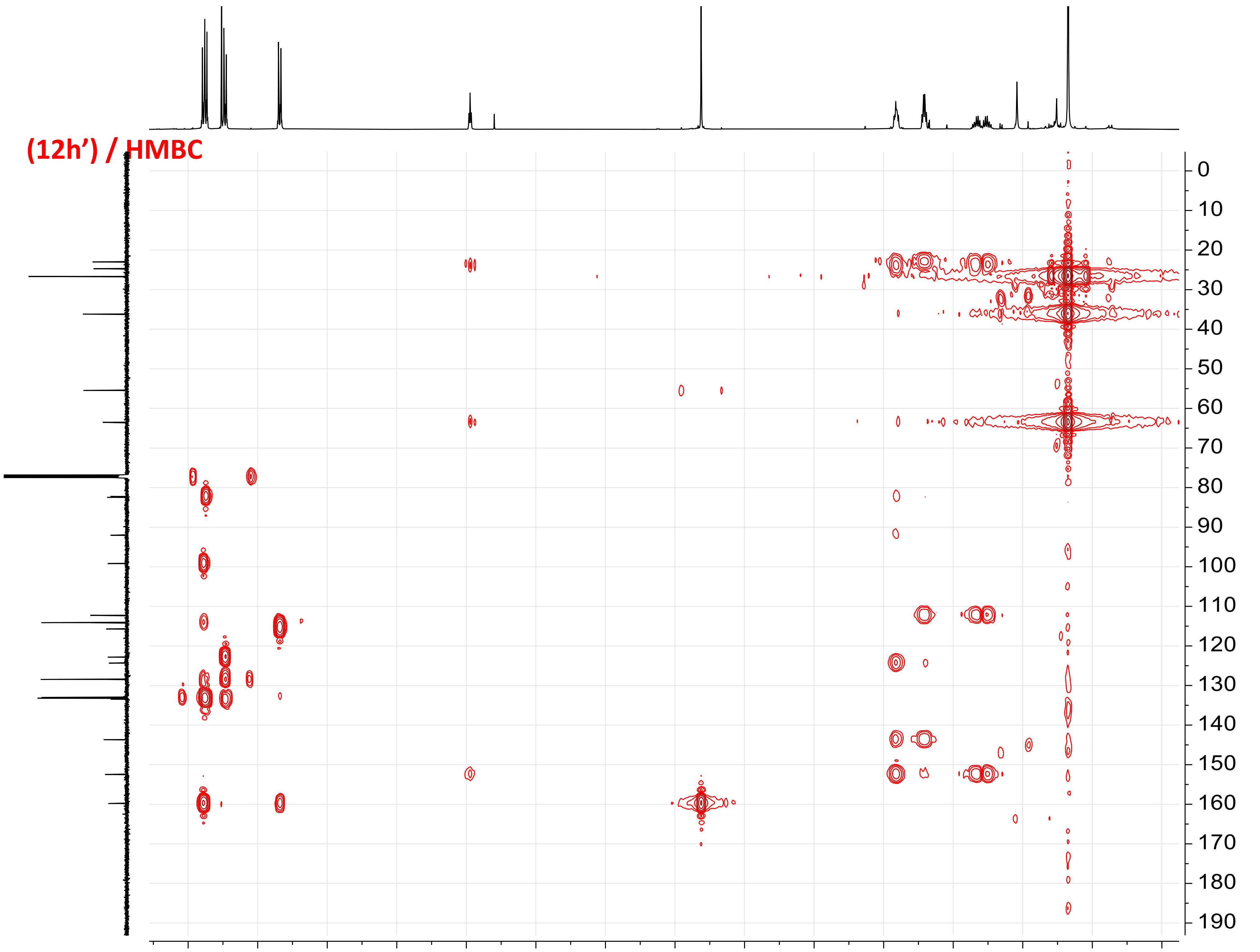
(12h') / HMBC
0
10
20
30
40
50
60
70
80
90
100
110
120
130
140
150
160
170
180
190
7.5
7.0
6.5
6.0
5.5
5.0
4.5
4.0
3.5
3.0
2.5
2.0
1.5
1.0
0.5

(17) / H

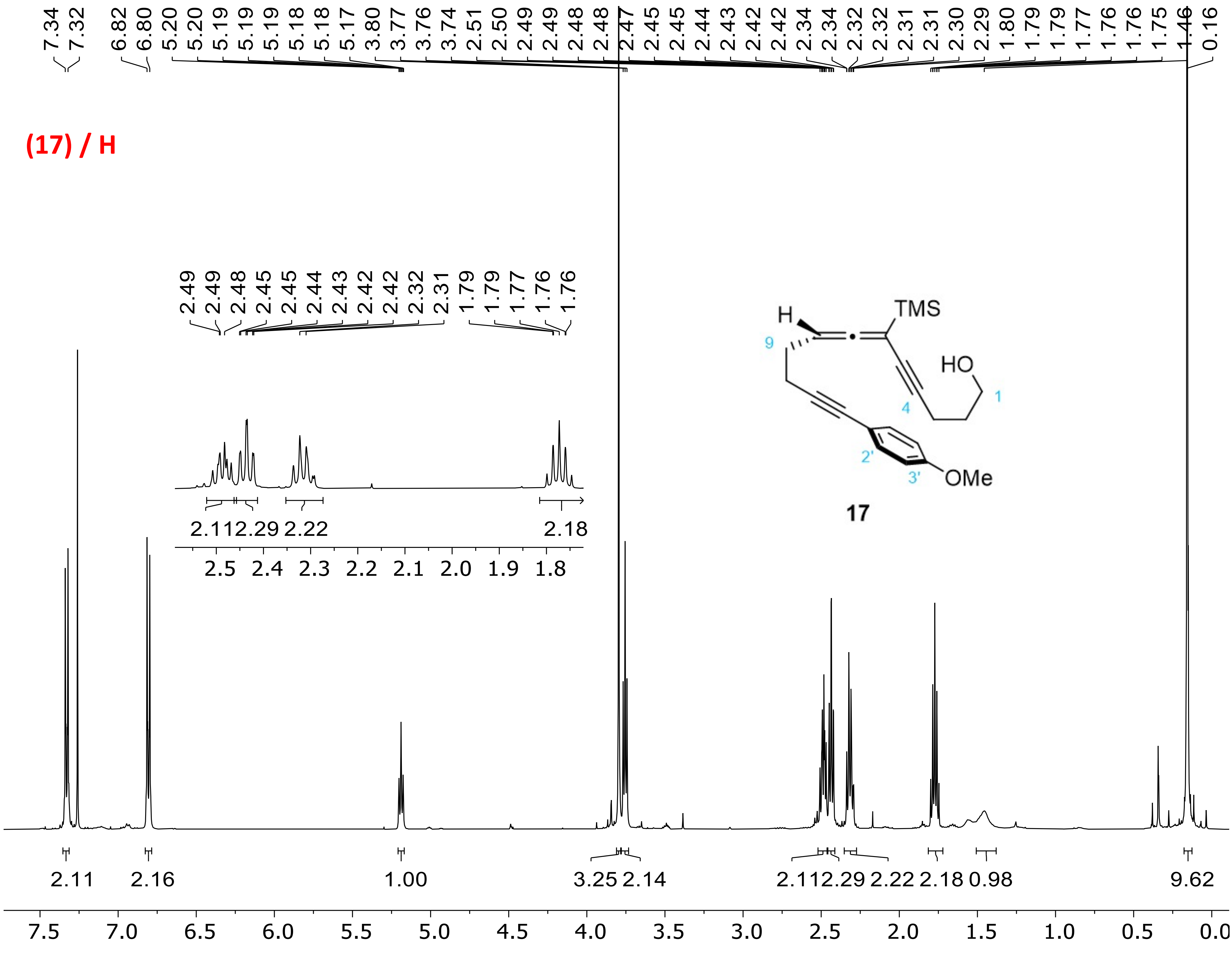

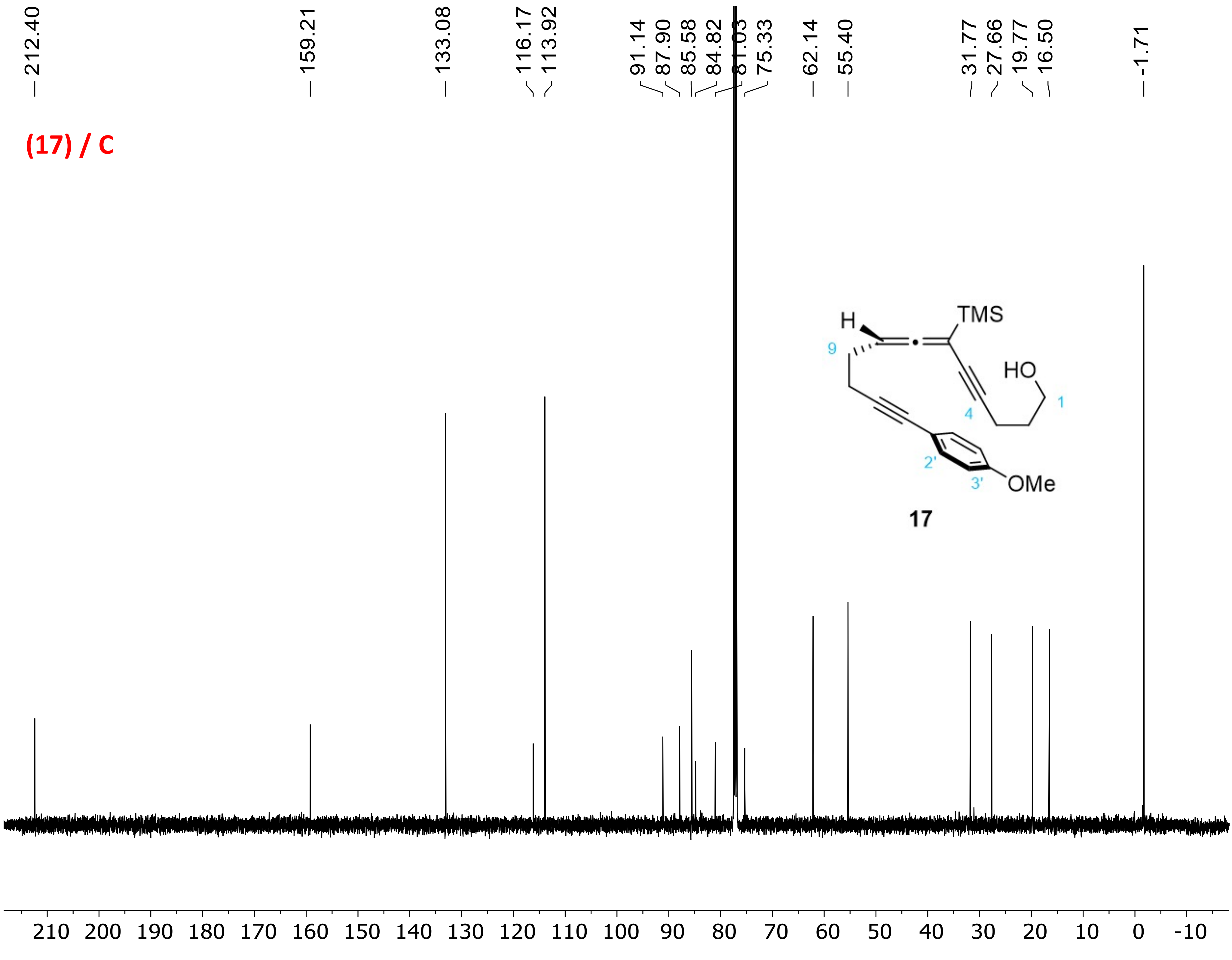
212.40
159.21
133.08
116.17
113.92
91.14
87.90
85.58
84.82
81.03
75.33
62.14
55.40
31.77
27.66
19.77
16.50
-1.71
(17) / C
H
TMS
9
HO
1
4
2'
3'
OMe
17
210 200 190 180 170 160 150 140 130 120 110 100 90 80 70 60 50 40 30 20 10 0 -10

(18) / H

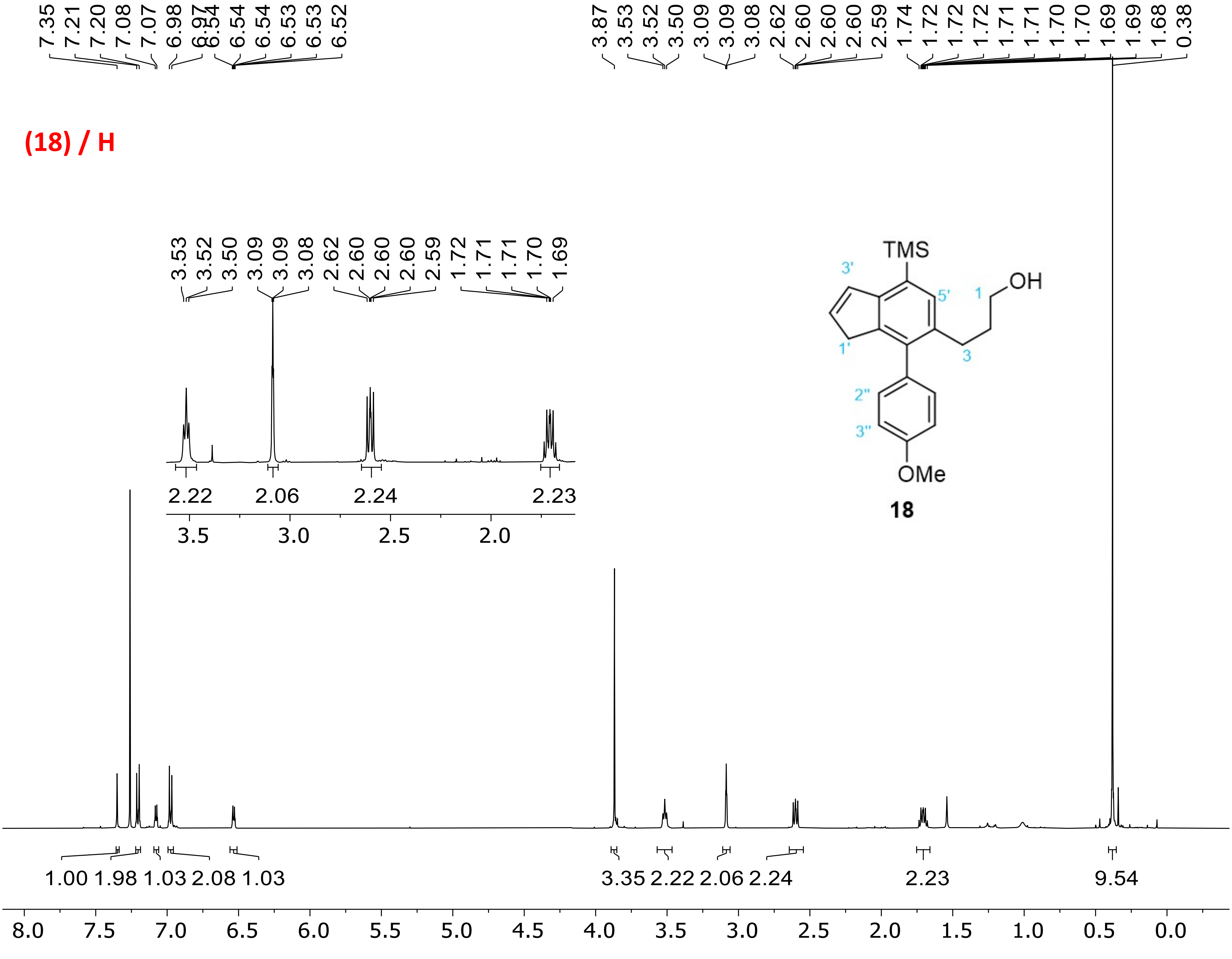
7.35
7.21
7.20
7.08
7.07
6.98
6.97
6.54
6.54
6.54
6.53
6.53
6.52
3.87
3.53
3.52
3.50
3.09
3.09
3.08
2.62
2.60
2.60
2.60
2.59
1.74
1.72
1.72
1.72
1.71
1.71
1.70
1.70
1.69
1.69
1.68
0.38
TMS
OH
OMe
18
1.00 1.98 1.03 2.08 1.03
3.35 2.22 2.06 2.24
2.23
9.54
2.22
2.06
2.24
2.23
8.0 7.5 7.0 6.5 6.0 5.5 5.0 4.5 4.0 3.5 3.0 2.5 2.0 1.5 1.0 0.5 0.0

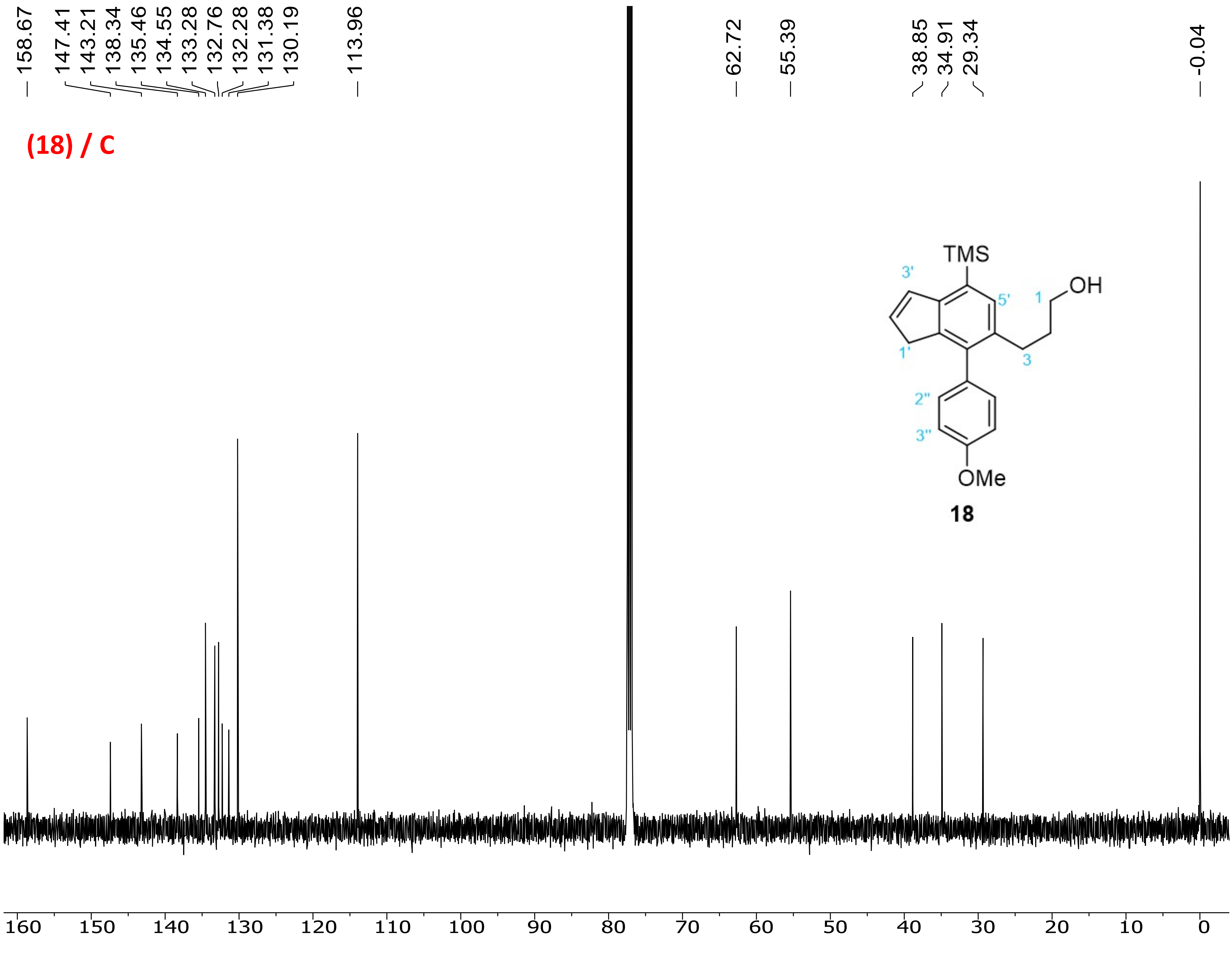
158.67
147.41
143.21
138.34
135.46
134.55
133.28
132.76
132.28
131.38
130.19
113.96
62.72
55.39
38.85
34.91
29.34
-0.04
(18) / C
TMS
3'
5'
1
OH
1'
3
2''
3''
OMe
18
160
150
140
130
120
110
100
90
80
70
60
50
40
30
20
10
0

(18-H$_2$) / H

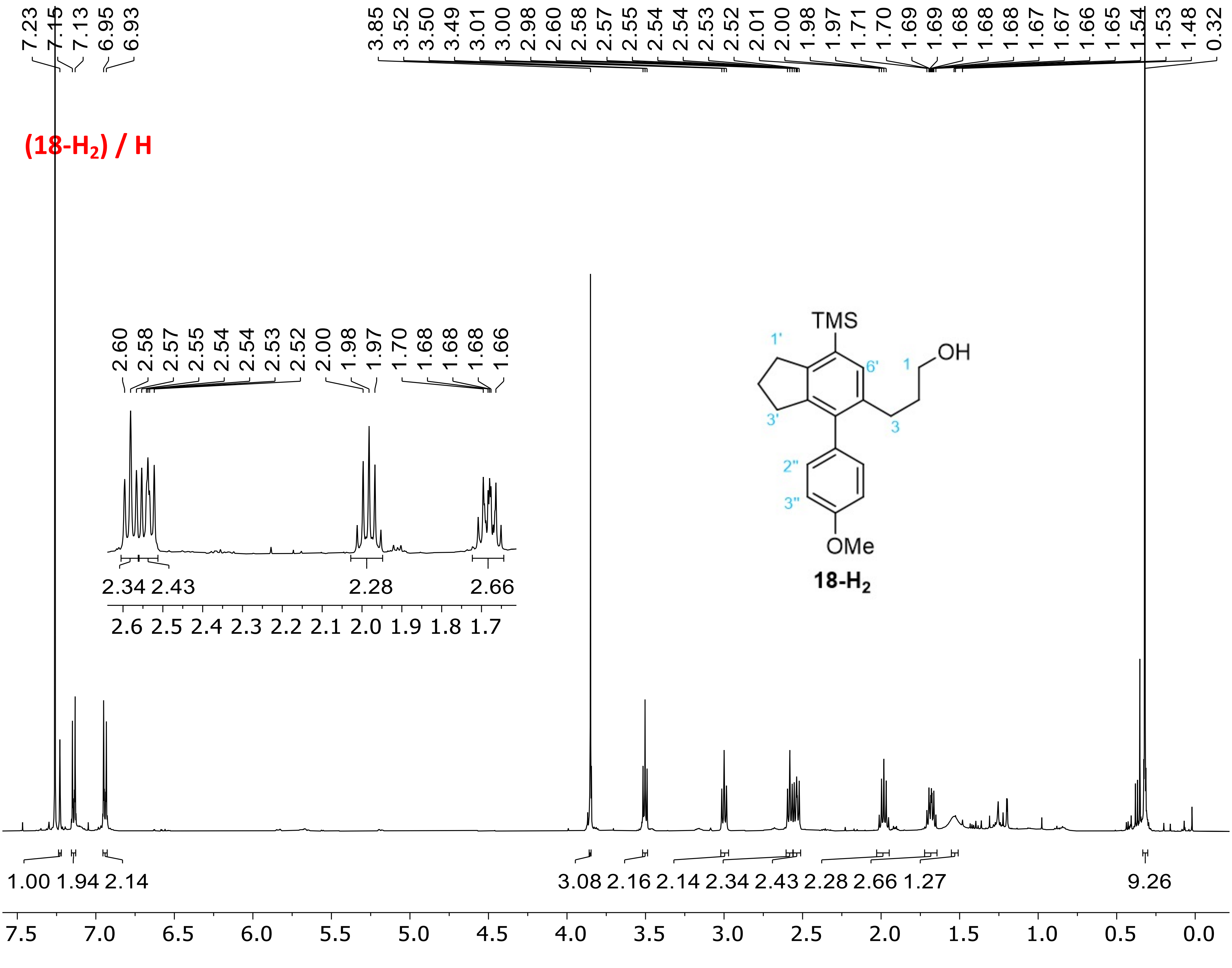
7.23
7.15
7.13
6.95
6.93
3.85
3.52
3.50
3.49
3.01
3.00
2.98
2.60
2.58
2.57
2.55
2.54
2.54
2.53
2.52
2.01
2.00
1.98
1.97
1.71
1.70
1.69
1.69
1.68
1.68
1.68
1.67
1.67
1.66
1.65
1.54
1.53
1.48
0.32
TMS
1'
6'
1
OH
3'
3
2"
3"
OMe
18-H2
2.34 2.43 2.28 2.66
1.00 1.94 2.14 3.08 2.16 2.14 2.34 2.43 2.28 2.66 1.27 9.26

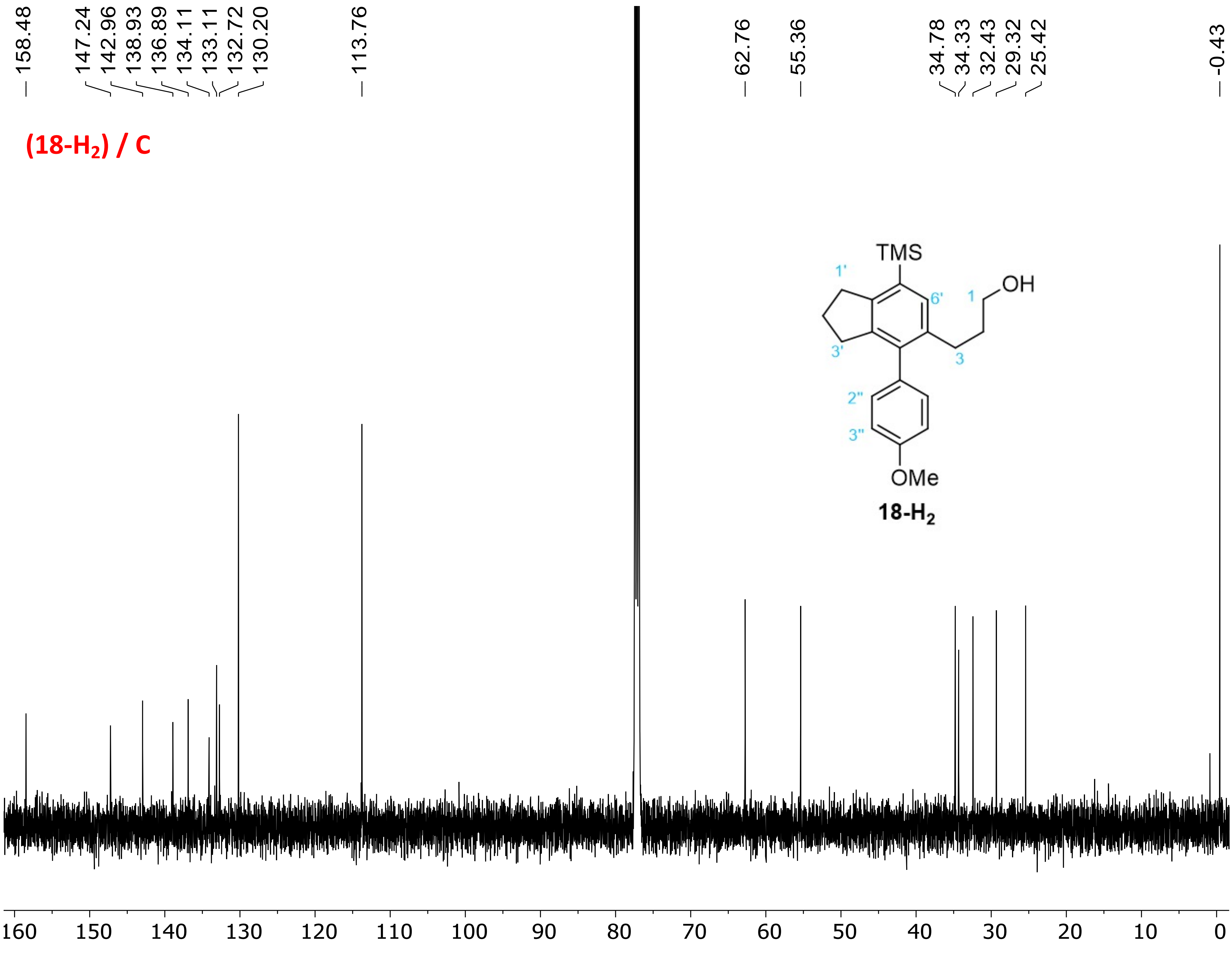
158.48
147.24
142.96
138.93
136.89
134.11
133.11
132.72
130.20
113.76
62.76
55.36
34.78
34.33
32.43
29.32
25.42
-0.43
(18-H2) / C
TMS
1'
6'
1
OH
3'
3
2"
3"
OMe
18-H2
160
150
140
130
120
110
100
90
80
70
60
50
40
30
20
10
0

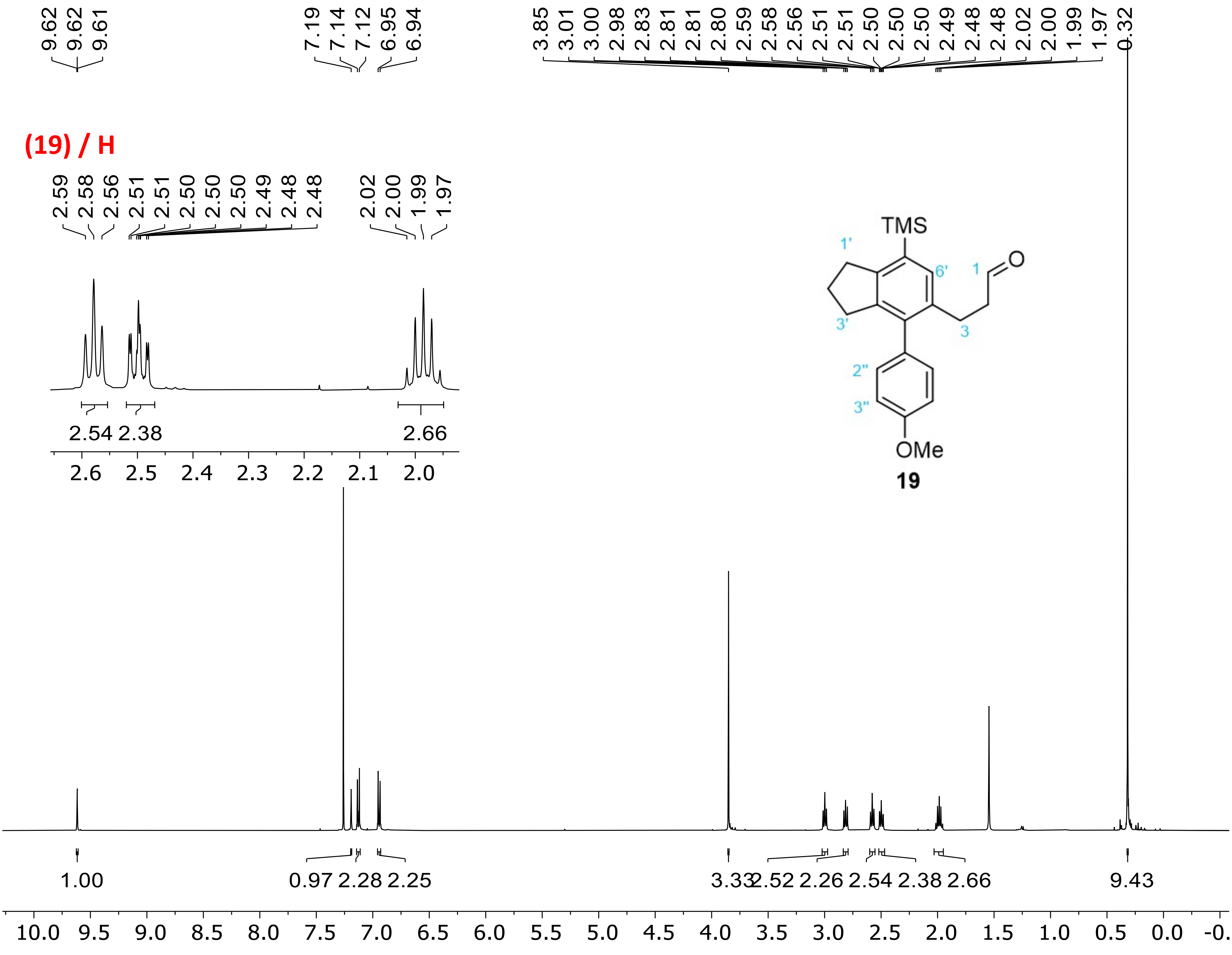
(19) / H
9.62
9.62
9.61
7.19
7.14
7.12
6.95
6.94
3.85
3.01
3.00
2.98
2.83
2.81
2.81
2.80
2.59
2.58
2.56
2.51
2.51
2.50
2.50
2.50
2.49
2.48
2.48
2.02
2.00
1.99
1.97
0.32
TMS
1'
6'
1
O
3'
3
2"
3"
OMe
19
2.54 2.38 2.66
2.6 2.5 2.4 2.3 2.2 2.1 2.0
1.00 0.97 2.28 2.25 3.33 2.52 2.26 2.54 2.38 2.66 9.43
10.0 9.5 9.0 8.5 8.0 7.5 7.0 6.5 6.0 5.5 5.0 4.5 4.0 3.5 3.0 2.5 2.0 1.5 1.0 0.5 0.0 -0.

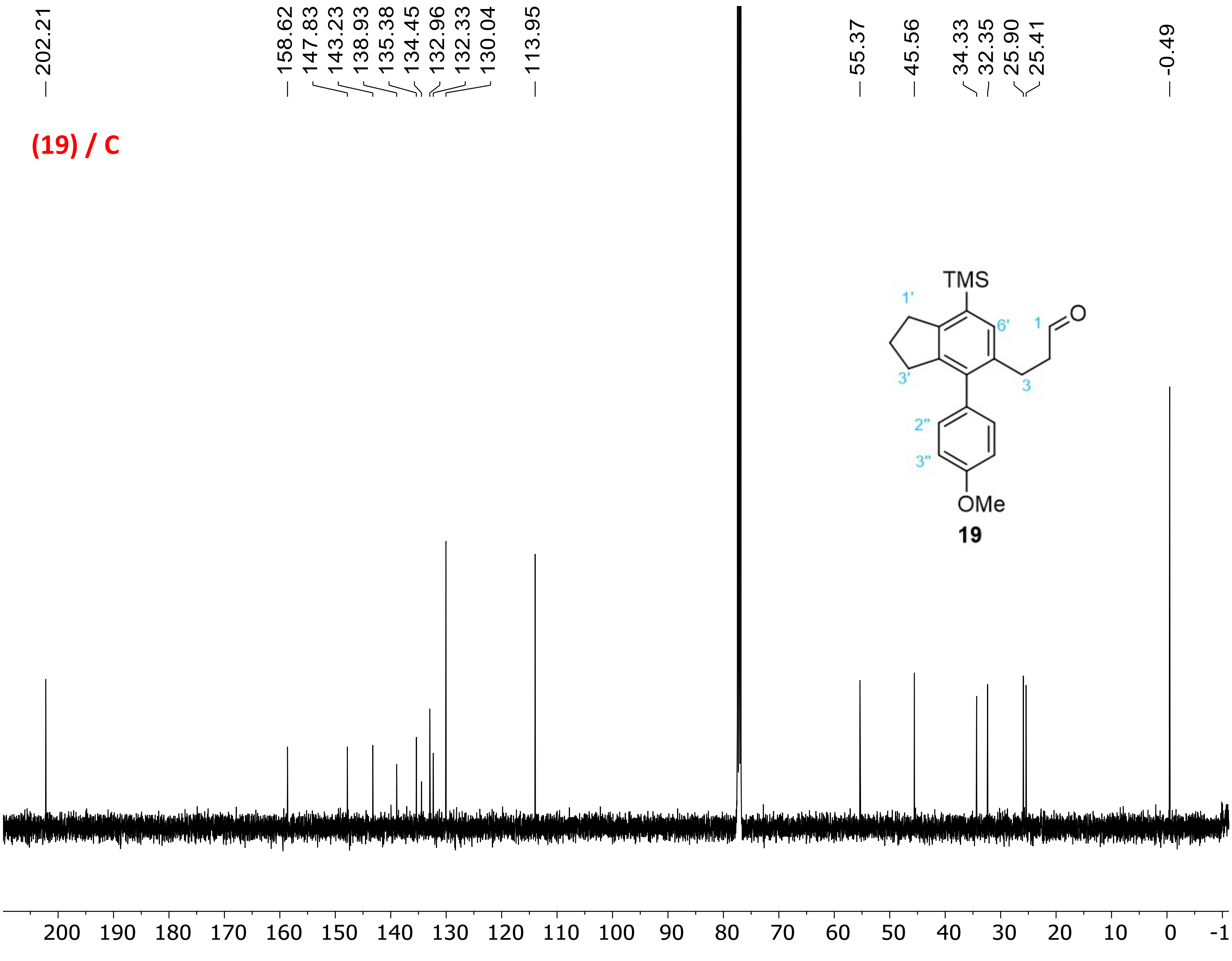

202.21
158.62
147.83
143.23
138.93
135.38
134.45
132.96
132.33
130.04
113.95
55.37
45.56
34.33
32.35
25.90
25.41
-0.49
(19) / C
TMS
1'
6'
1
O
3'
3
2"
3"
OMe
19
200 190 180 170 160 150 140 130 120 110 100 90 80 70 60 50 40 30 20 10 0 -1

(23) / H

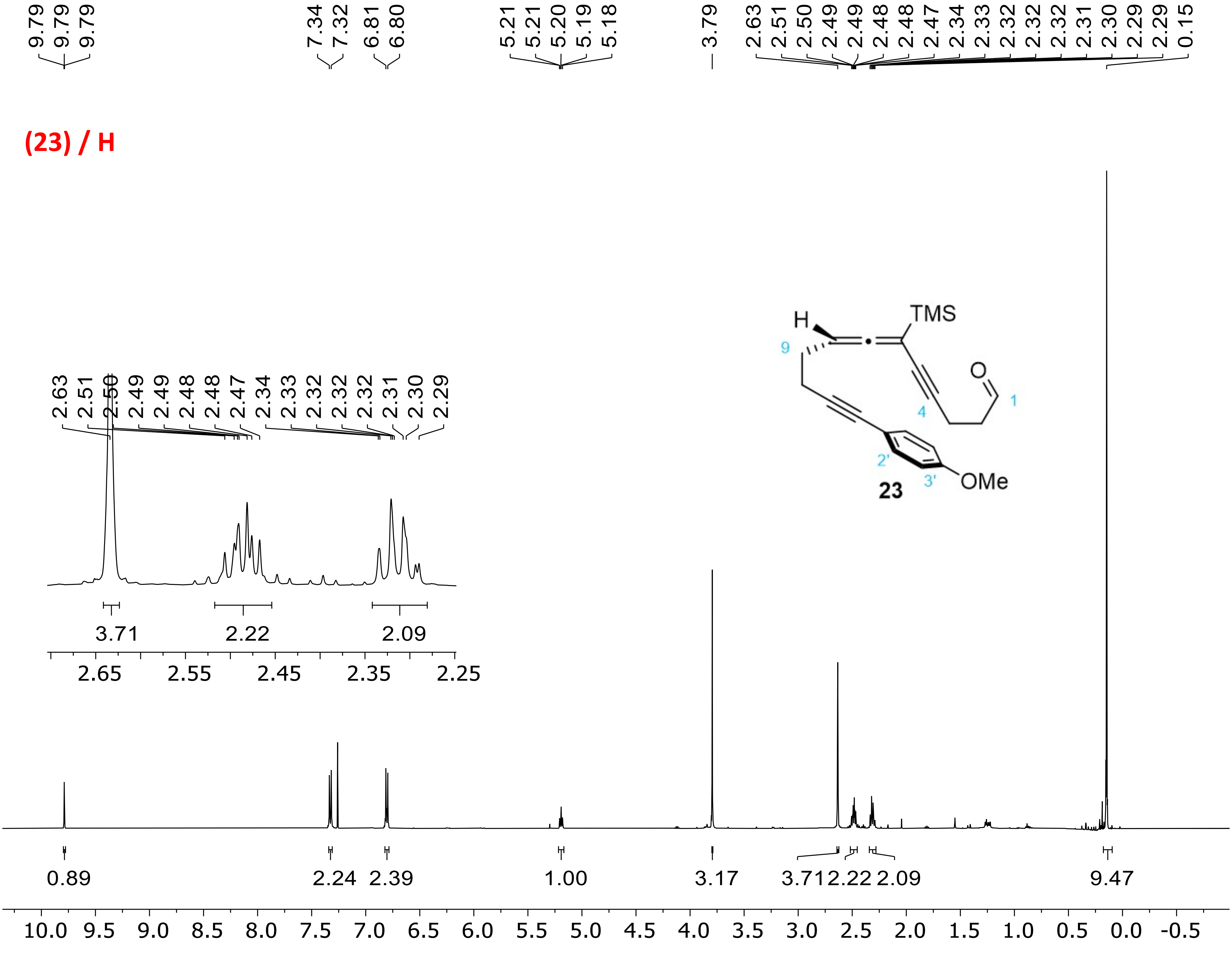
9.79 9.79 9.79
7.34 7.32 6.81 6.80
5.21 5.21 5.20 5.19 5.18
3.79
2.63 2.51 2.50 2.49 2.49 2.48 2.48 2.47 2.34 2.33 2.32 2.32 2.32 2.31 2.30 2.29 2.29 0.15
H
TMS
9
O
1
4
2'
3'
OMe
23
3.71
2.22
2.09
2.65 2.55 2.45 2.35 2.25
0.89 2.24 2.39 1.00 3.17 3.71 2.22 2.09 9.47
10.0 9.5 9.0 8.5 8.0 7.5 7.0 6.5 6.0 5.5 5.0 4.5 4.0 3.5 3.0 2.5 2.0 1.5 1.0 0.5 0.0 -0.5

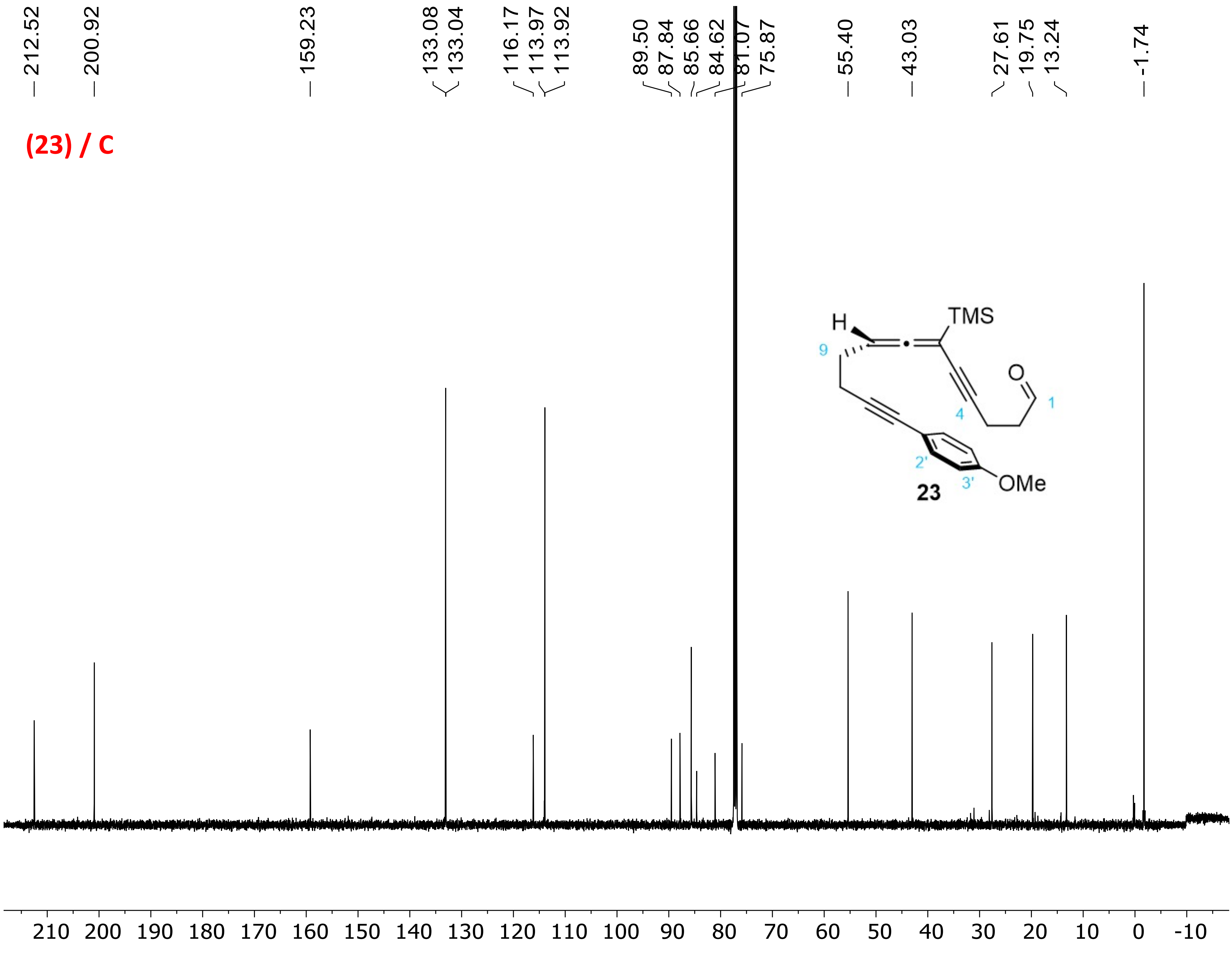
212.52
200.92
159.23
133.08
133.04
116.17
113.97
113.92
89.50
87.84
85.66
84.62
81.07
75.87
55.40
43.03
27.61
19.75
13.24
-1.74
(23) / C
H
TMS
9
O
1
4
2'
3'
OMe
23
210 200 190 180 170 160 150 140 130 120 110 100 90 80 70 60 50 40 30 20 10 0 -10

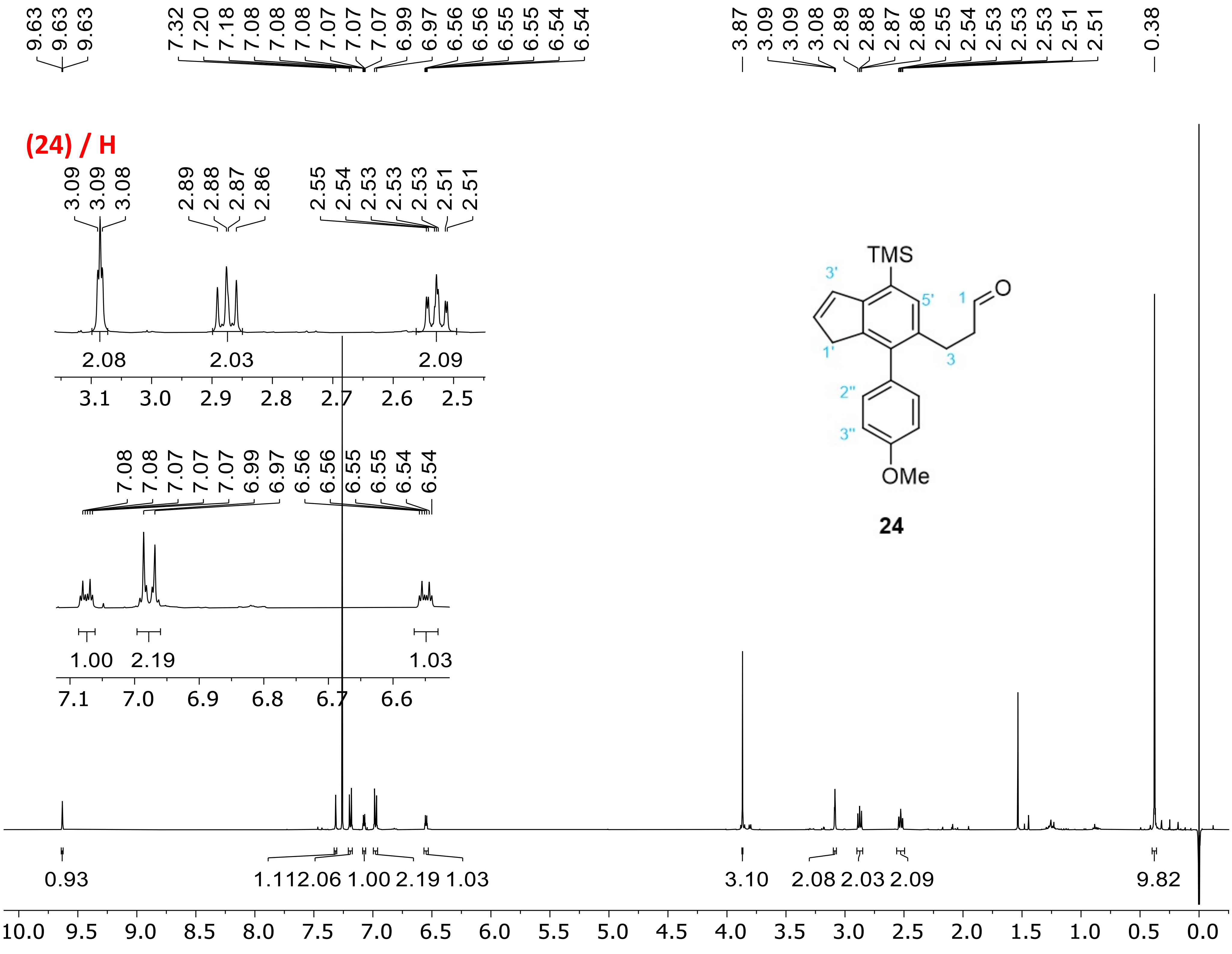
9.63 9.63 9.63
7.32 7.20 7.18 7.08 7.08 7.08 7.07 7.07 7.07 6.99 6.97 6.56 6.56 6.55 6.55 6.54 6.54
3.87 3.09 3.09 3.08 2.89 2.88 2.87 2.86 2.55 2.54 2.53 2.53 2.53 2.51 2.51
0.38
(24) / H
3.09 3.09 3.08
2.89 2.88 2.87 2.86
2.55 2.54 2.53 2.53 2.53 2.51 2.51
2.08 2.03 2.09
3.1 3.0 2.9 2.8 2.7 2.6 2.5
7.08 7.08 7.07 7.07 7.07 6.99 6.97 6.56 6.56 6.55 6.55 6.54 6.54
1.00 2.19 1.03
7.1 7.0 6.9 6.8 6.7 6.6
TMS
3'
5'
1
O
1'
3
2''
3''
OMe
24
0.93
1.11 2.06 1.00 2.19 1.03
3.10 2.08 2.03 2.09
9.82
10.0 9.5 9.0 8.5 8.0 7.5 7.0 6.5 6.0 5.5 5.0 4.5 4.0 3.5 3.0 2.5 2.0 1.5 1.0 0.5 0.0

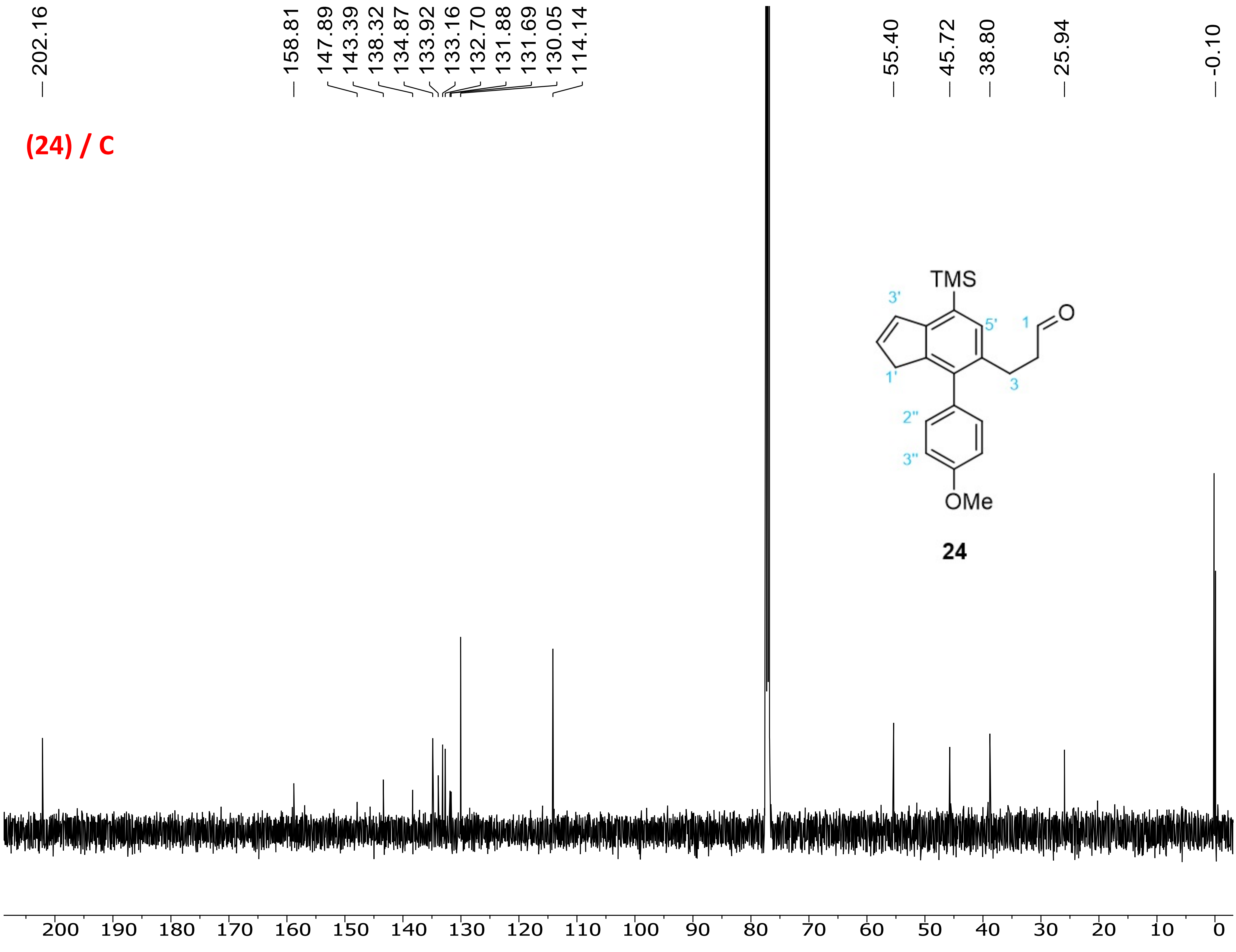
202.16
158.81
147.89
143.39
138.32
134.87
133.92
133.16
132.70
131.88
131.69
130.05
114.14
55.40
45.72
38.80
25.94
-0.10
(24) / C
TMS
3'
5'
1
O
1'
3
2''
3''
OMe
24
200 190 180 170 160 150 140 130 120 110 100 90 80 70 60 50 40 30 20 10 0

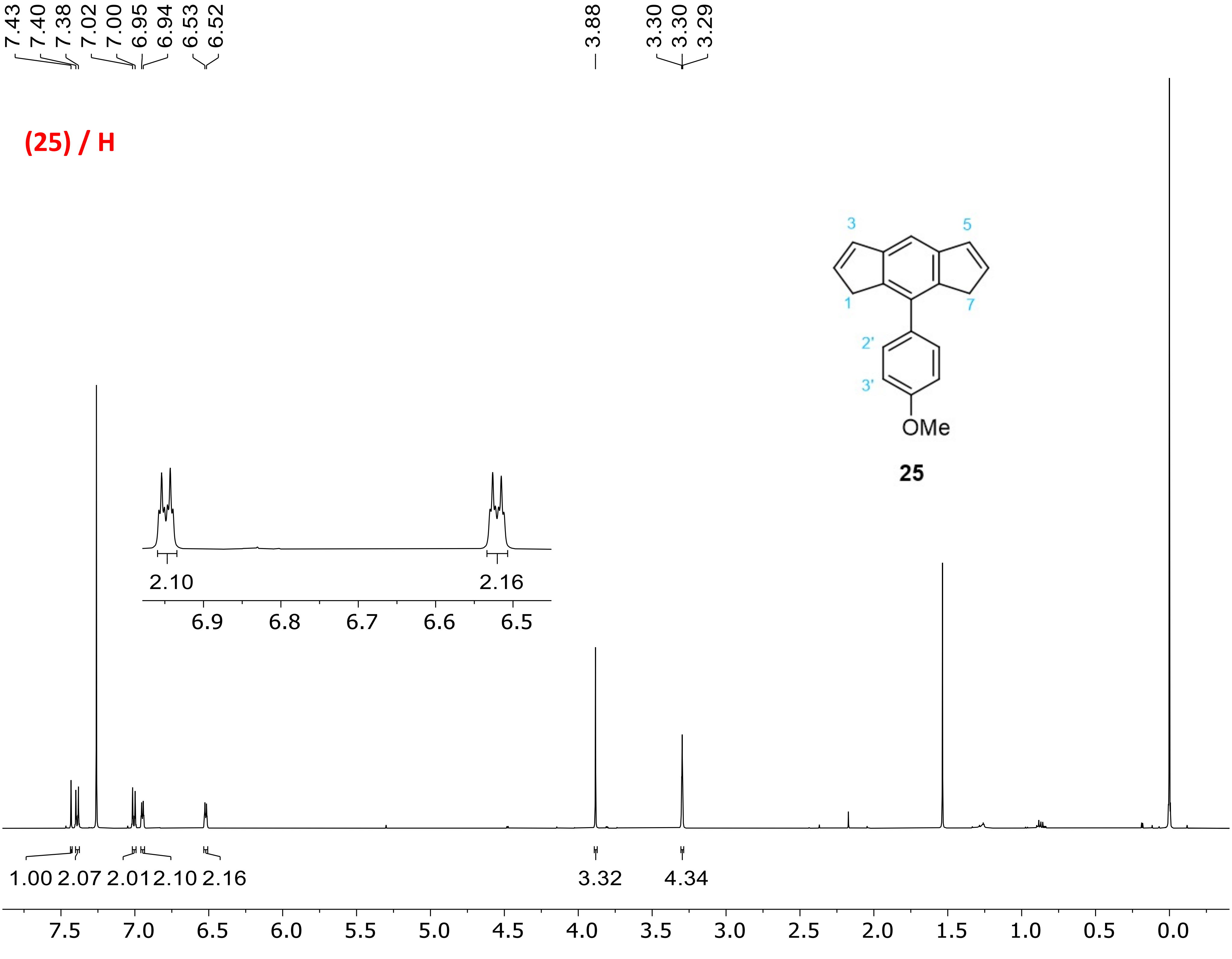
7.43
7.40
7.38
7.02
7.00
6.95
6.94
6.53
6.52
3.88
3.30
3.30
3.29
(25) / H
3
5
1
7
2'
3'
OMe
25
2.10
2.16
6.9
6.8
6.7
6.6
6.5
1.00
2.07
2.01
2.10
2.16
3.32
4.34
7.5
7.0
6.5
6.0
5.5
5.0
4.5
4.0
3.5
3.0
2.5
2.0
1.5
1.0
0.5
0.0

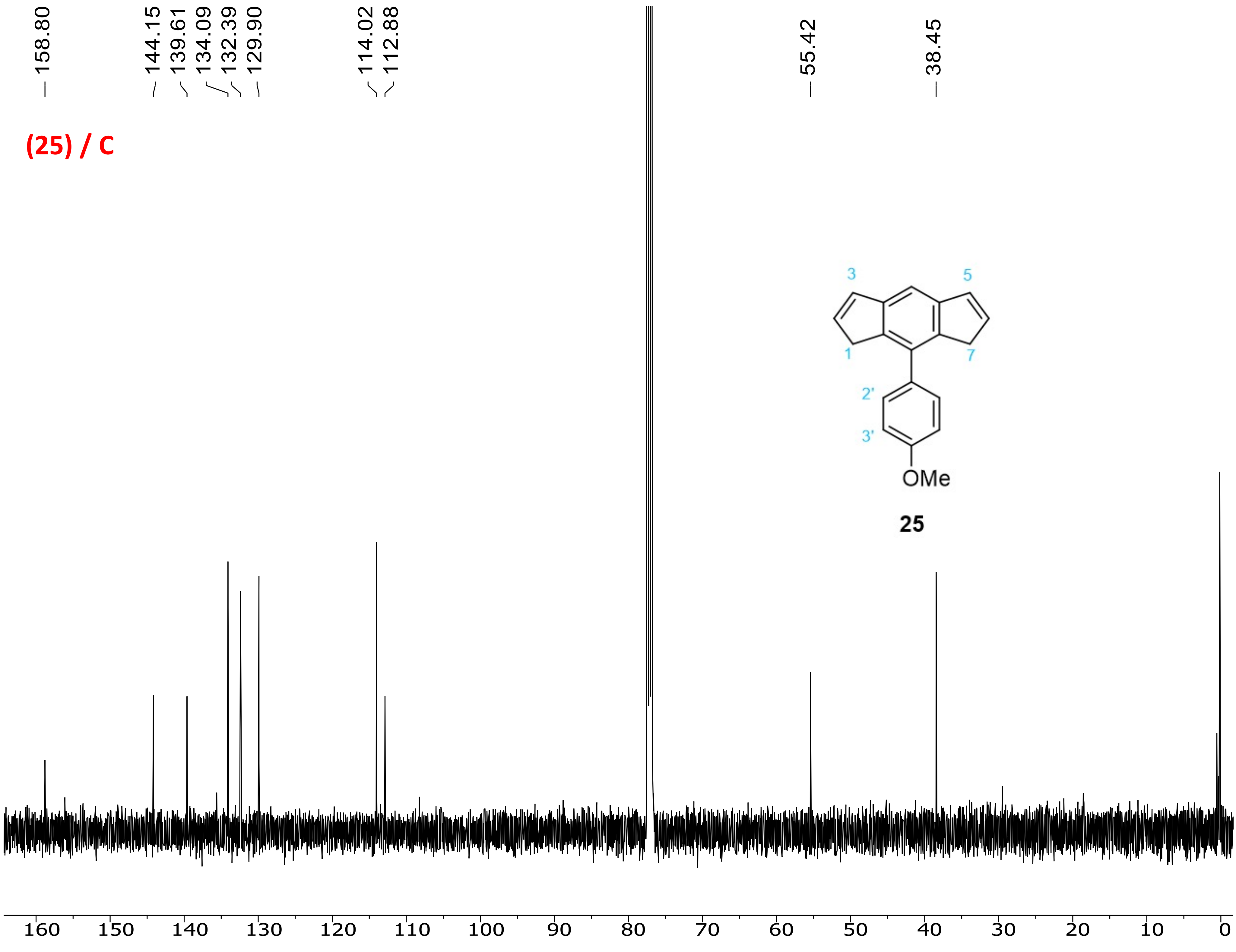
158.80
144.15
139.61
134.09
132.39
129.90
114.02
112.88
55.42
38.45
(25) / C
3
5
1
7
2'
3'
OMe
25
160 150 140 130 120 110 100 90 80 70 60 50 40 30 20 10 0

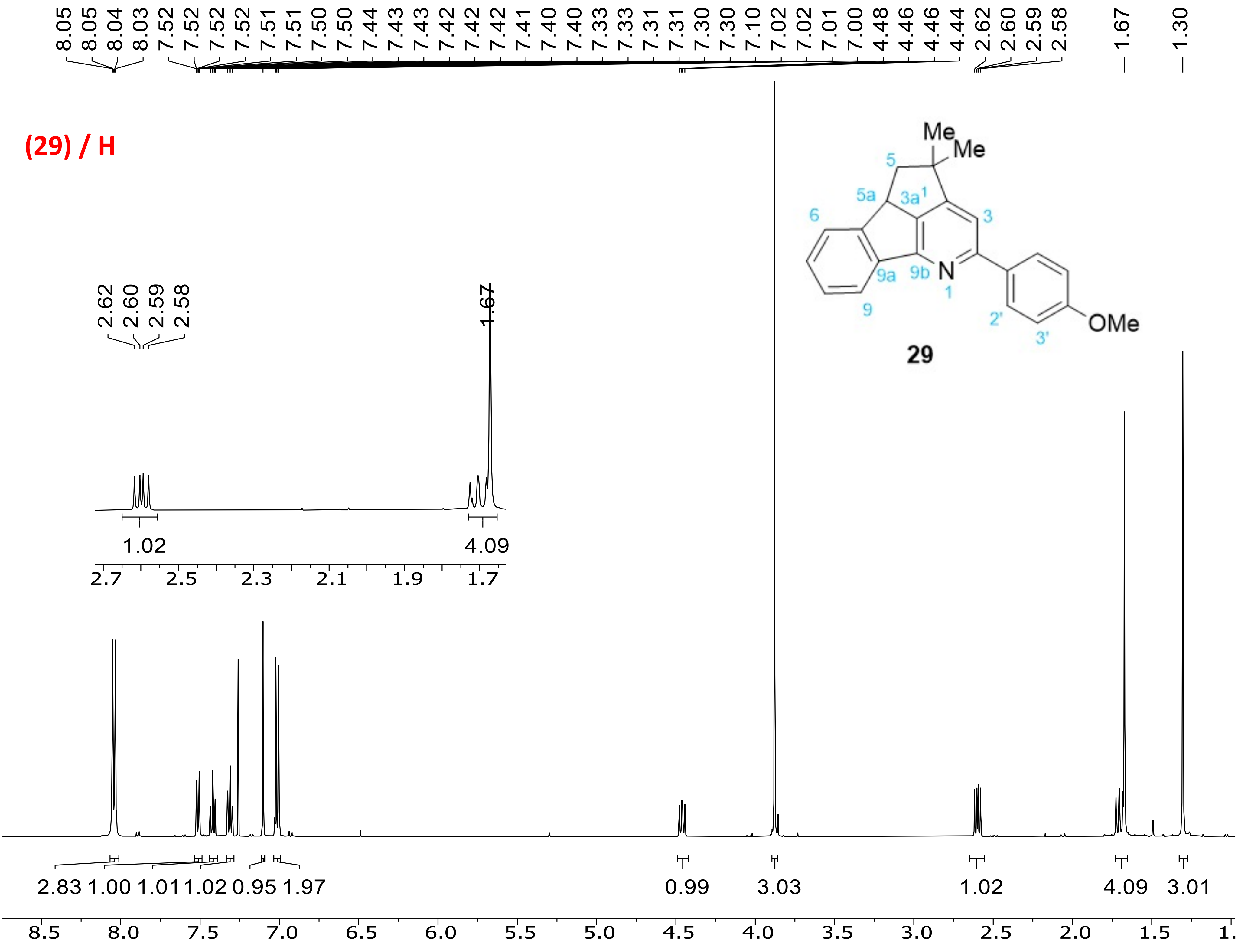
(29) / H
8.05 8.05 8.04 8.03 7.52 7.52 7.52 7.52 7.51 7.51 7.50 7.50 7.44 7.43 7.43 7.42 7.42 7.42 7.41 7.40 7.40 7.33 7.33 7.31 7.31 7.30 7.30 7.10 7.02 7.02 7.01 7.00 4.48 4.46 4.46 4.44 2.62 2.60 2.59 2.58 1.67 1.30
Me
Me
5
5a
3a
1
3
6
9a
9b
N
1
9
2'
3'
OMe
29
2.62 2.60 2.59 2.58
1.67
1.02
4.09
2.7 2.5 2.3 2.1 1.9 1.7
2.83 1.00 1.01 1.02 0.95 1.97
0.99
3.03
1.02
4.09 3.01
8.5 8.0 7.5 7.0 6.5 6.0 5.5 5.0 4.5 4.0 3.5 3.0 2.5 2.0 1.5 1.

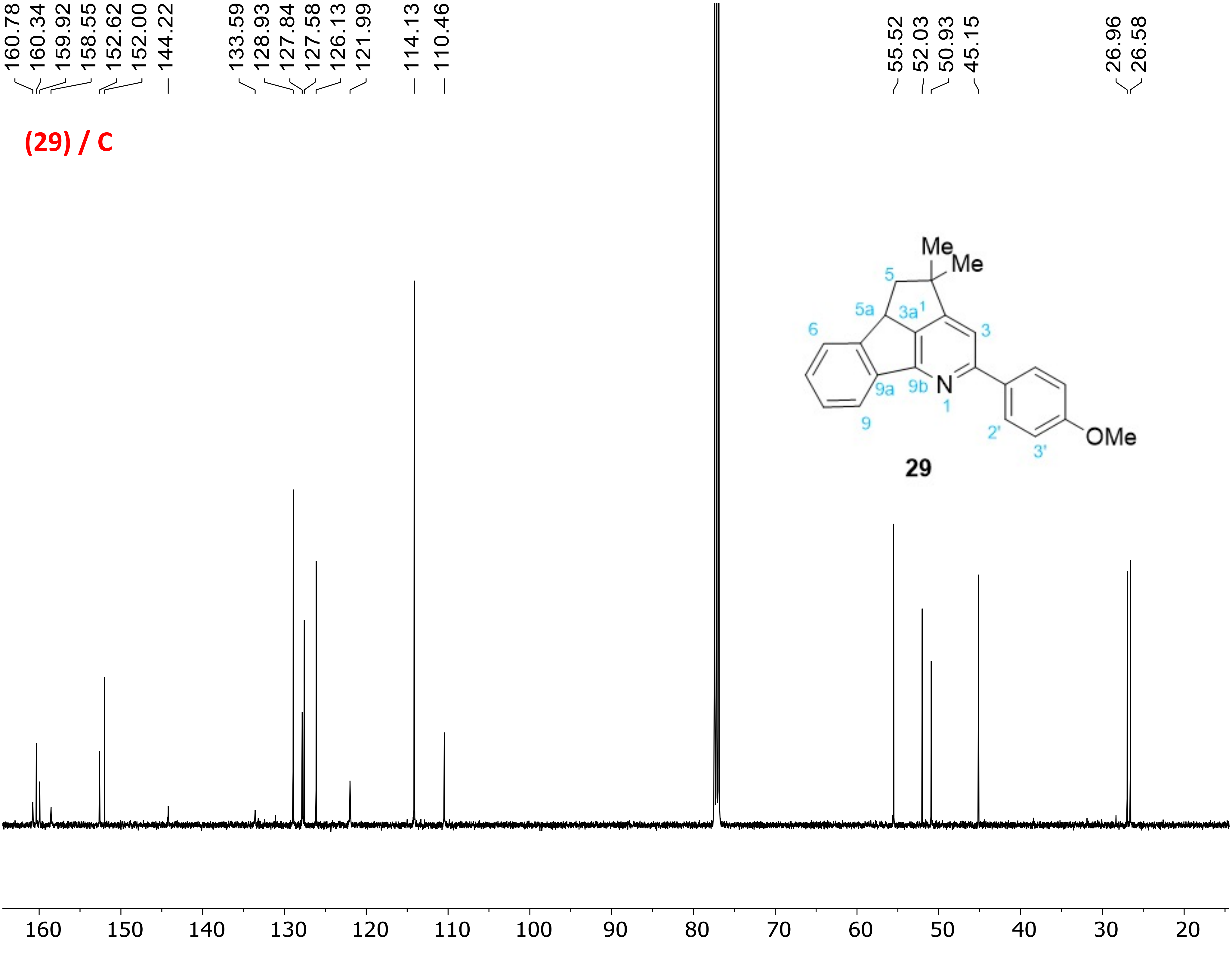
160.78
160.34
159.92
158.55
152.62
152.00
144.22
133.59
128.93
127.84
127.58
126.13
121.99
114.13
110.46
55.52
52.03
50.93
45.15
26.96
26.58
(29) / C
Me
Me
5
5a
3a
1
3
6
9a
9b
N
1
9
2'
3'
OMe
29
160
150
140
130
120
110
100
90
80
70
60
50
40
30
20

(29) / HSQC

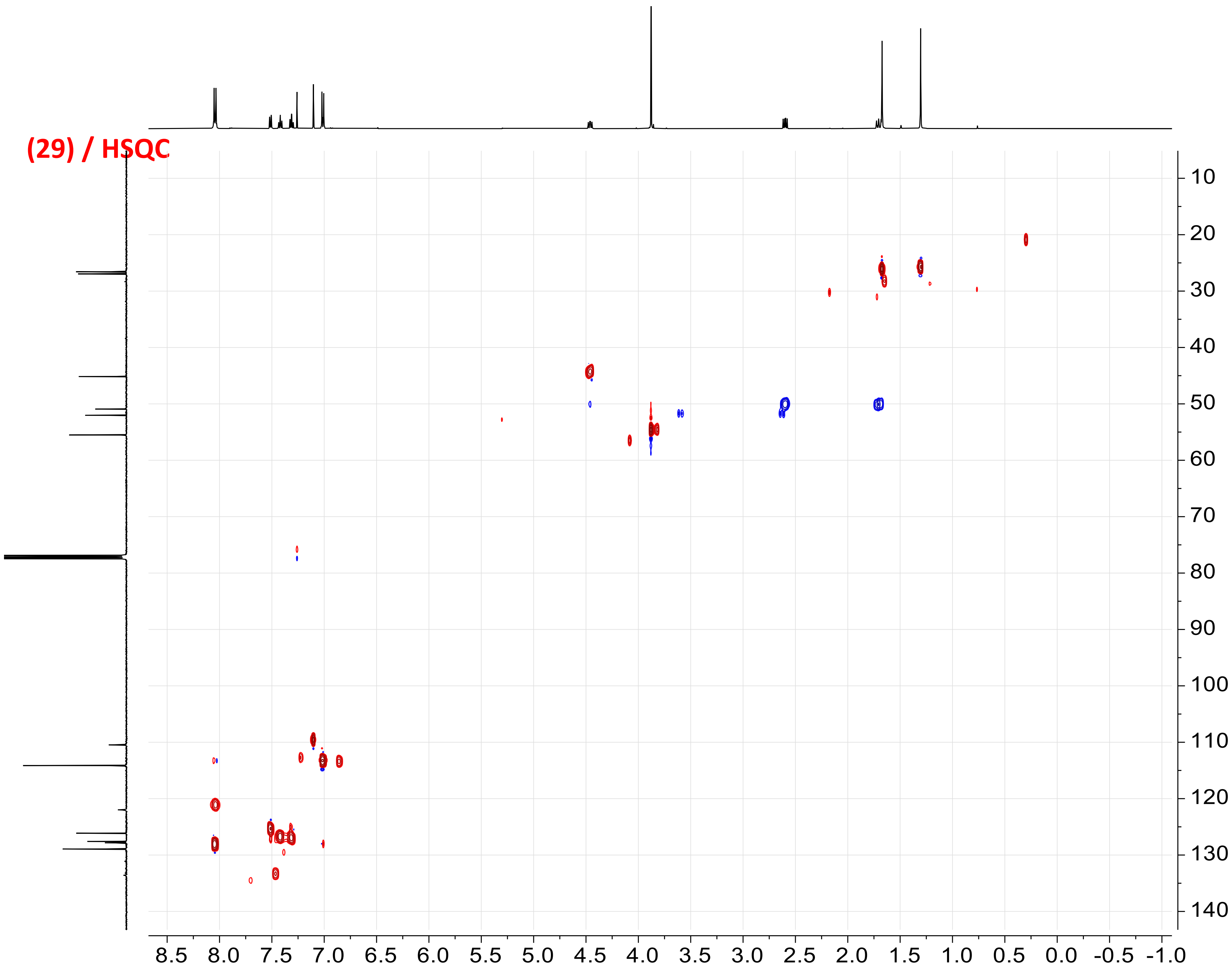
8.5 8.0 7.5 7.0 6.5 6.0 5.5 5.0 4.5 4.0 3.5 3.0 2.5 2.0 1.5 1.0 0.5 0.0 -0.5 -1.0
10 20 30 40 50 60 70 80 90 100 110 120 130 140

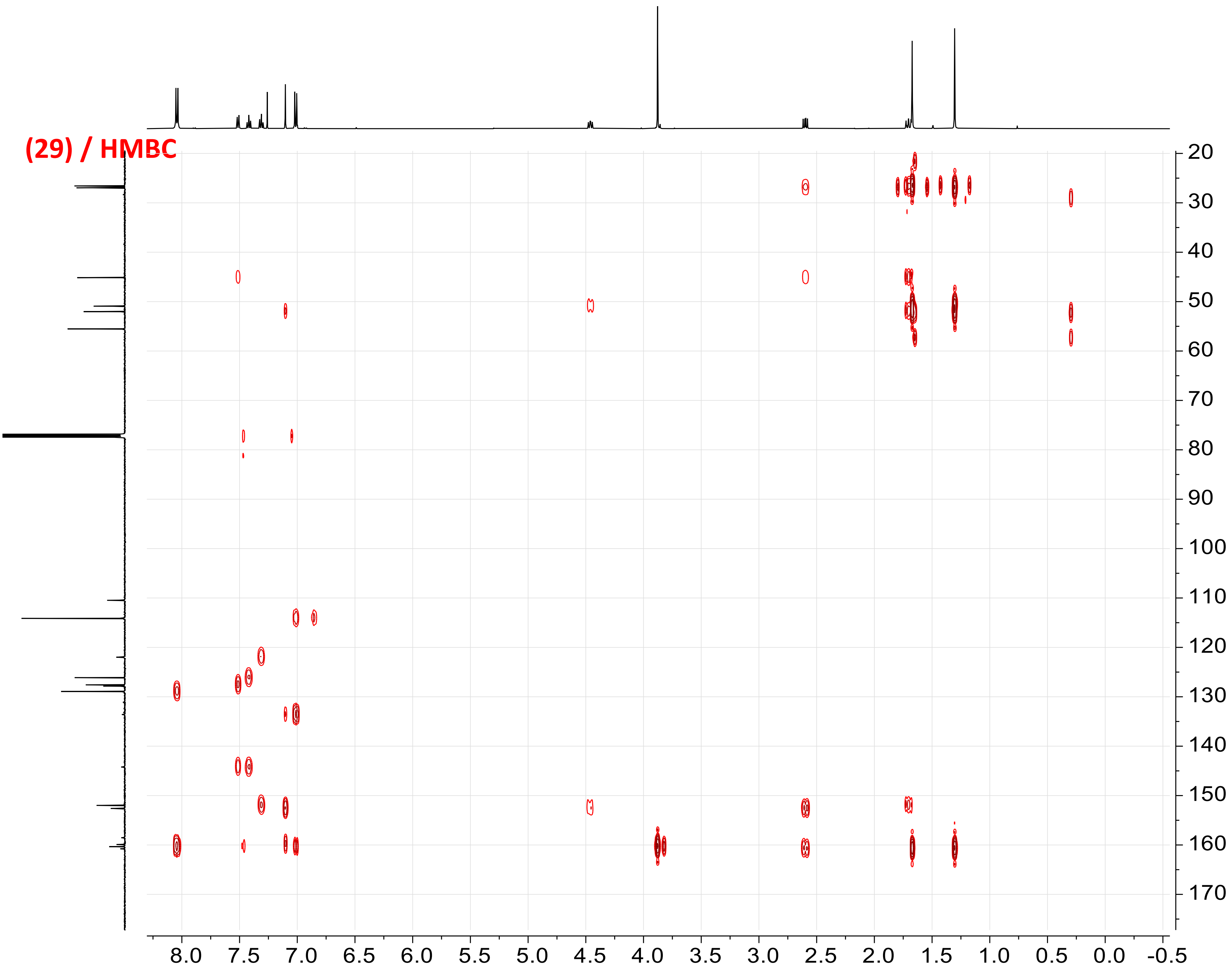
(29) / HMBC
20
30
40
50
60
70
80
90
100
110
120
130
140
150
160
170
8.0
7.5
7.0
6.5
6.0
5.5
5.0
4.5
4.0
3.5
3.0
2.5
2.0
1.5
1.0
0.5
0.0
-0.5

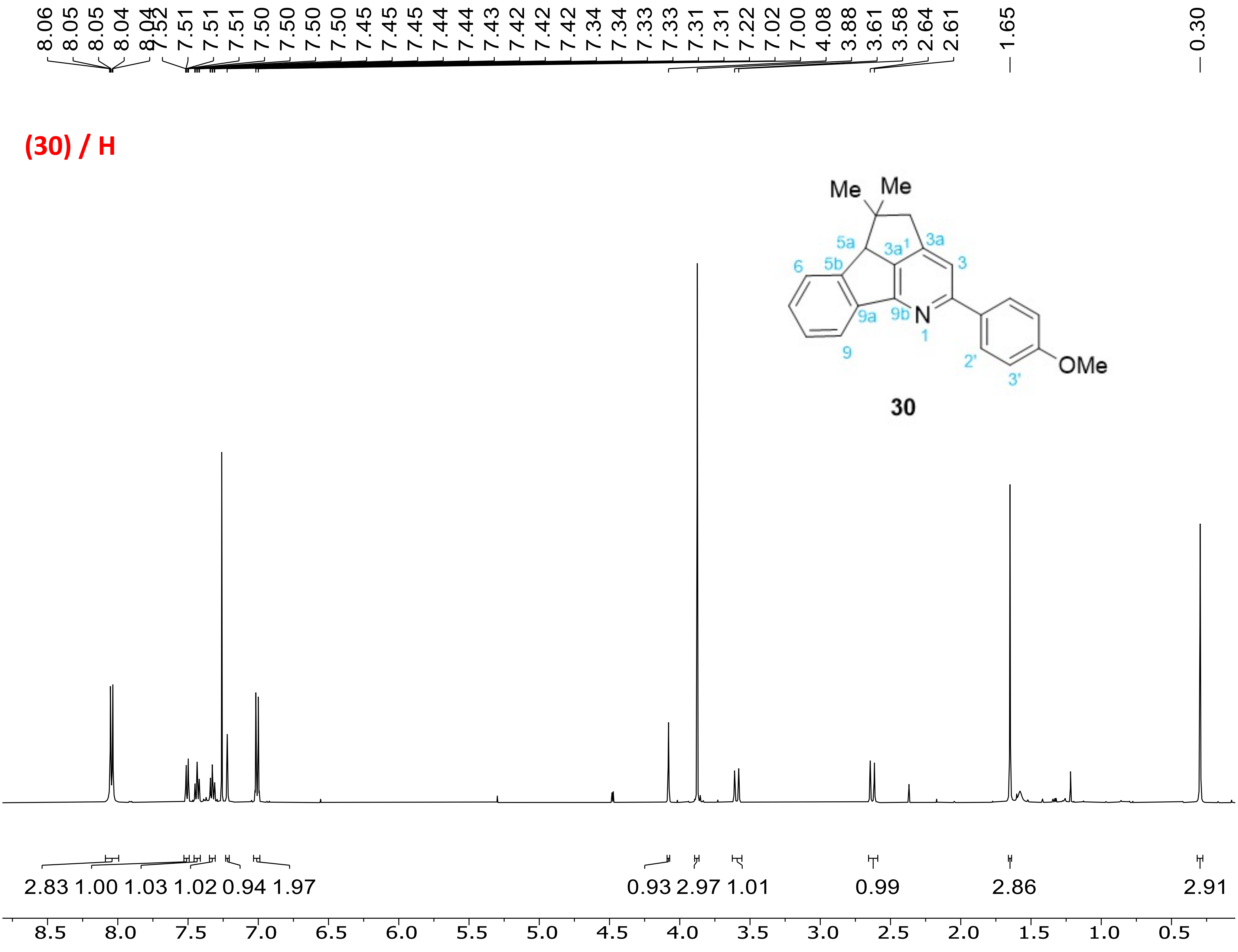
8.06
8.05
8.05
8.04
8.04
7.52
7.51
7.51
7.51
7.50
7.50
7.50
7.50
7.45
7.45
7.45
7.44
7.44
7.43
7.42
7.42
7.42
7.34
7.34
7.33
7.33
7.31
7.31
7.22
7.02
7.00
4.08
3.88
3.61
3.58
2.64
2.61
1.65
0.30
(30) / H
Me
Me
5a
3a1
3a
3
6
5b
9a
9b
N
1
9
2'
3'
OMe
30
2.83 1.00 1.03 1.02 0.94 1.97
0.93 2.97 1.01
0.99
2.86
2.91
8.5 8.0 7.5 7.0 6.5 6.0 5.5 5.0 4.5 4.0 3.5 3.0 2.5 2.0 1.5 1.0 0.5

(30) / C

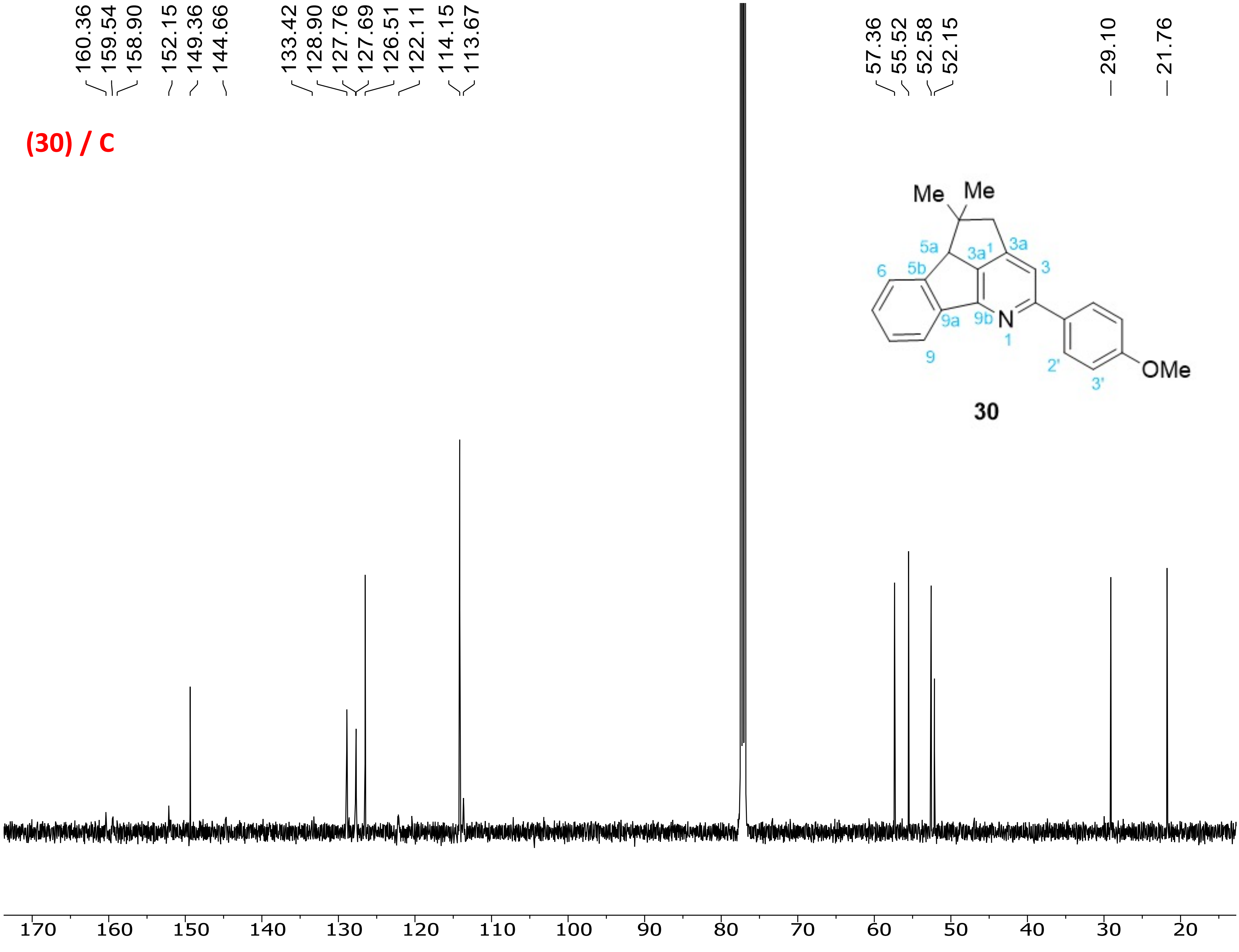
160.36
159.54
158.90
152.15
149.36
144.66
133.42
128.90
127.76
127.69
126.51
122.11
114.15
113.67
57.36
55.52
52.58
52.15
29.10
21.76
170
160
150
140
130
120
110
100
90
80
70
60
50
40
30
20
Me
Me
5a
3a1
3a
3
6
5b
9a
9b
N
1
9
2'
3'
OMe

30

(30) / HSQC

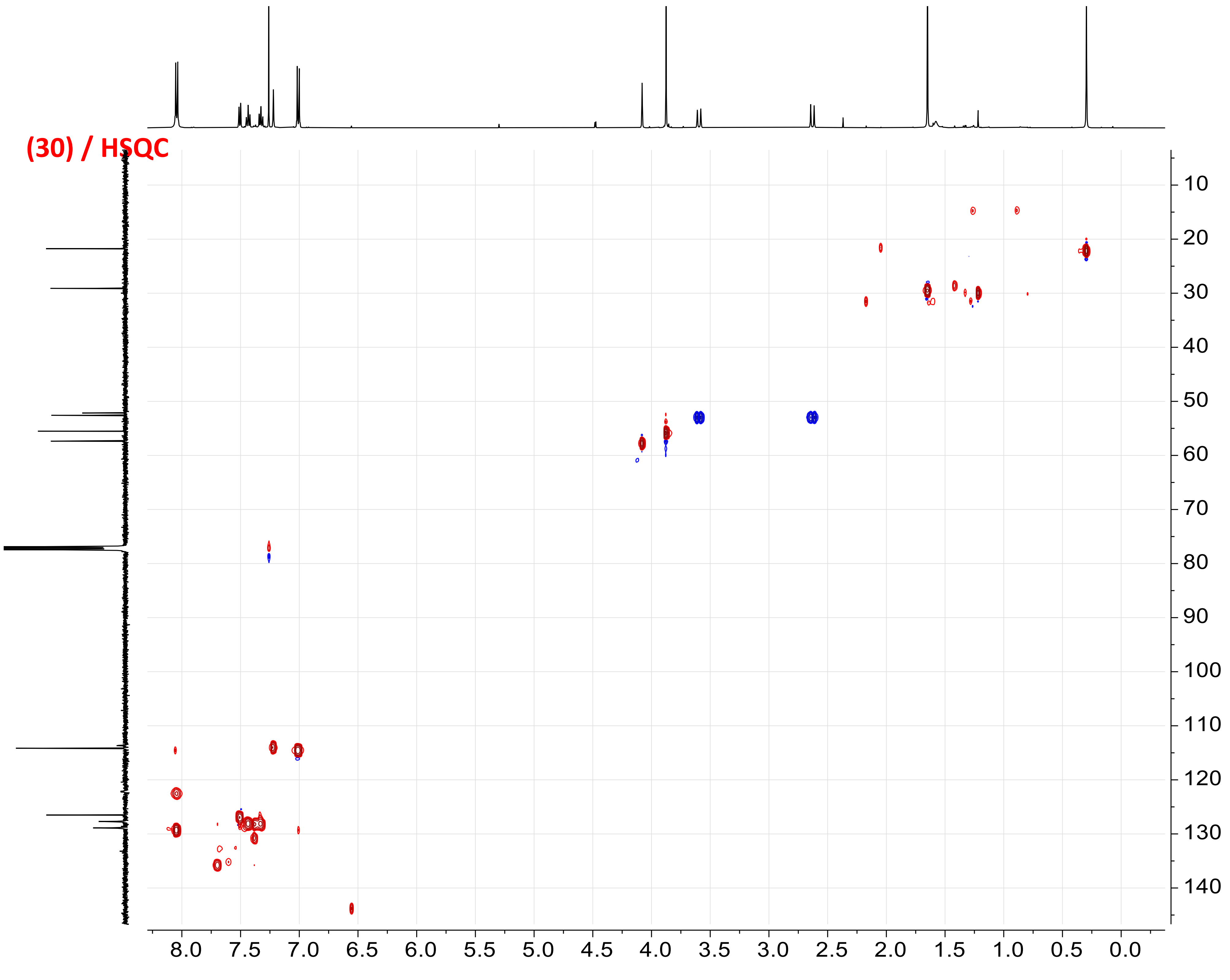
10
20
30
40
50
60
70
80
90
100
110
120
130
140
8.0 7.5 7.0 6.5 6.0 5.5 5.0 4.5 4.0 3.5 3.0 2.5 2.0 1.5 1.0 0.5 0.0

(30) / HMBC

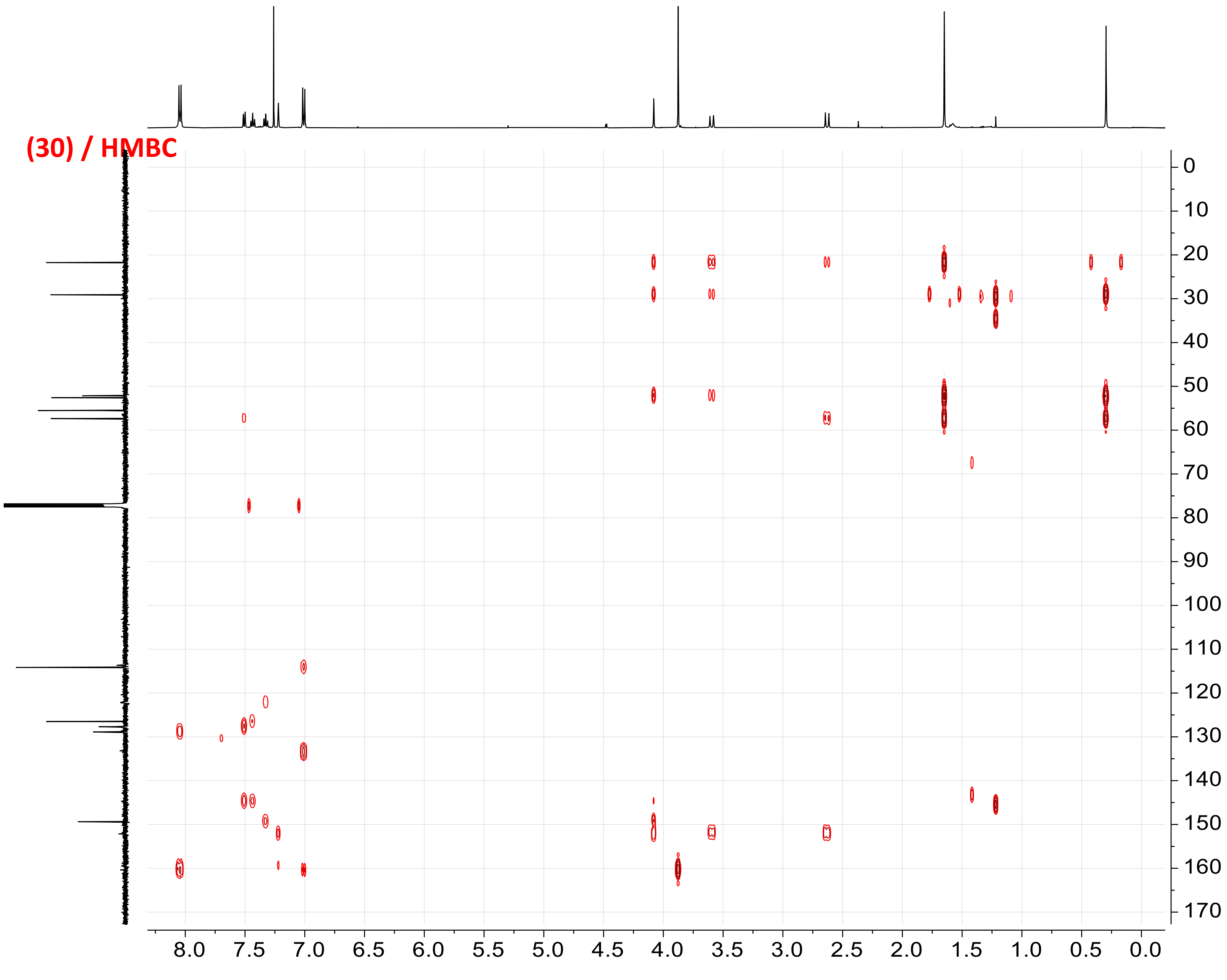
8.0
7.5
7.0
6.5
6.0
5.5
5.0
4.5
4.0
3.5
3.0
2.5
2.0
1.5
1.0
0.5
0.0
0
10
20
30
40
50
60
70
80
90
100
110
120
130
140
150
160
170

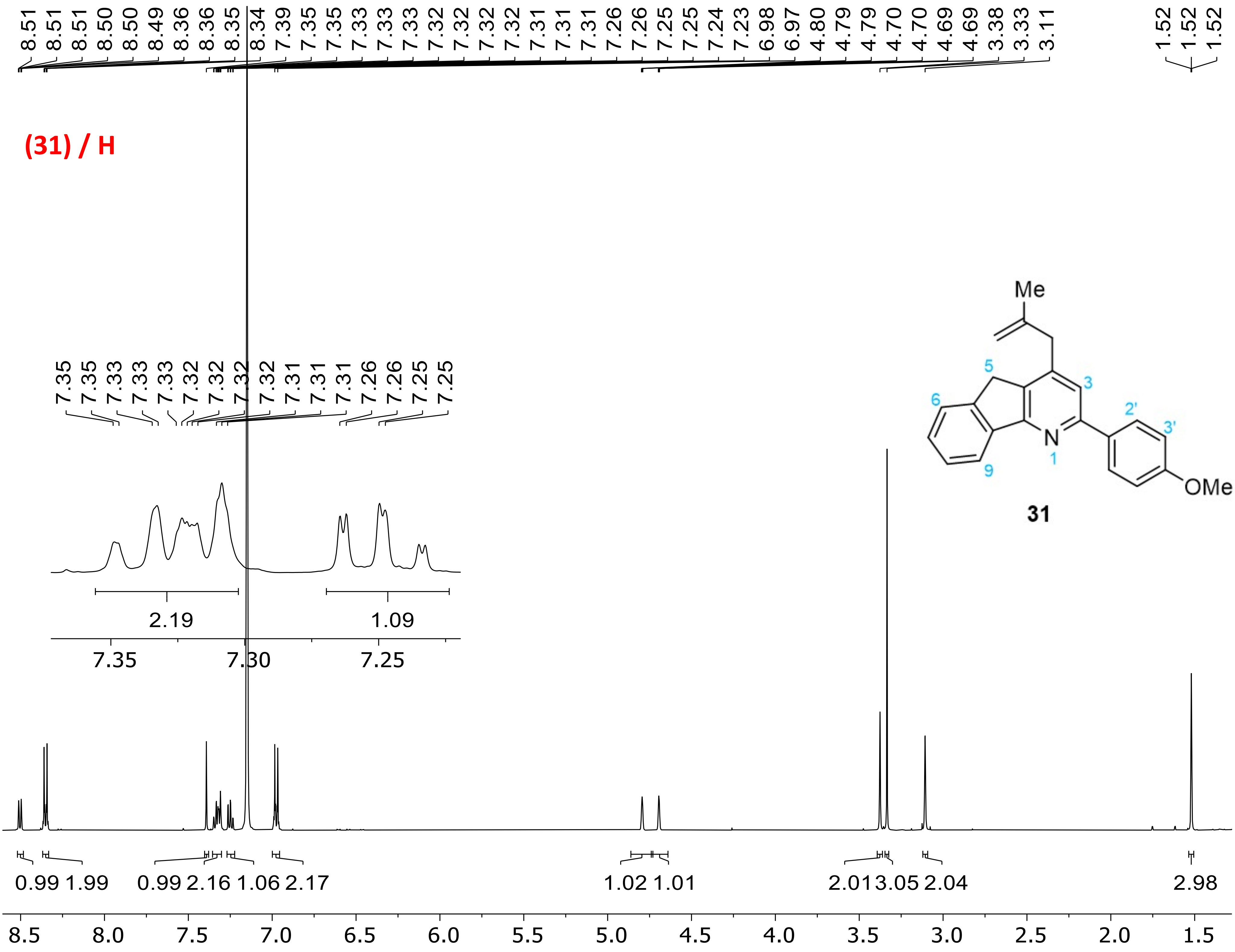
8.51
8.51
8.51
8.50
8.50
8.49
8.36
8.36
8.35
8.34
7.39
7.35
7.35
7.33
7.33
7.33
7.32
7.32
7.32
7.32
7.31
7.31
7.31
7.26
7.26
7.25
7.25
7.24
7.23
6.98
6.97
4.80
4.79
4.79
4.70
4.70
4.69
4.69
3.38
3.33
3.11
1.52
1.52
1.52
(31) / H
7.35
7.35
7.33
7.33
7.33
7.32
7.32
7.32
7.32
7.31
7.31
7.31
7.26
7.26
7.25
7.25
2.19
1.09
7.35
7.30
7.25
0.99 1.99 0.99 2.16 1.06 2.17 1.02 1.01 2.01 3.05 2.04 2.98
8.5 8.0 7.5 7.0 6.5 6.0 5.5 5.0 4.5 4.0 3.5 3.0 2.5 2.0 1.5
Me
5
3
6
2'
3'
N
1
9
OMe

31

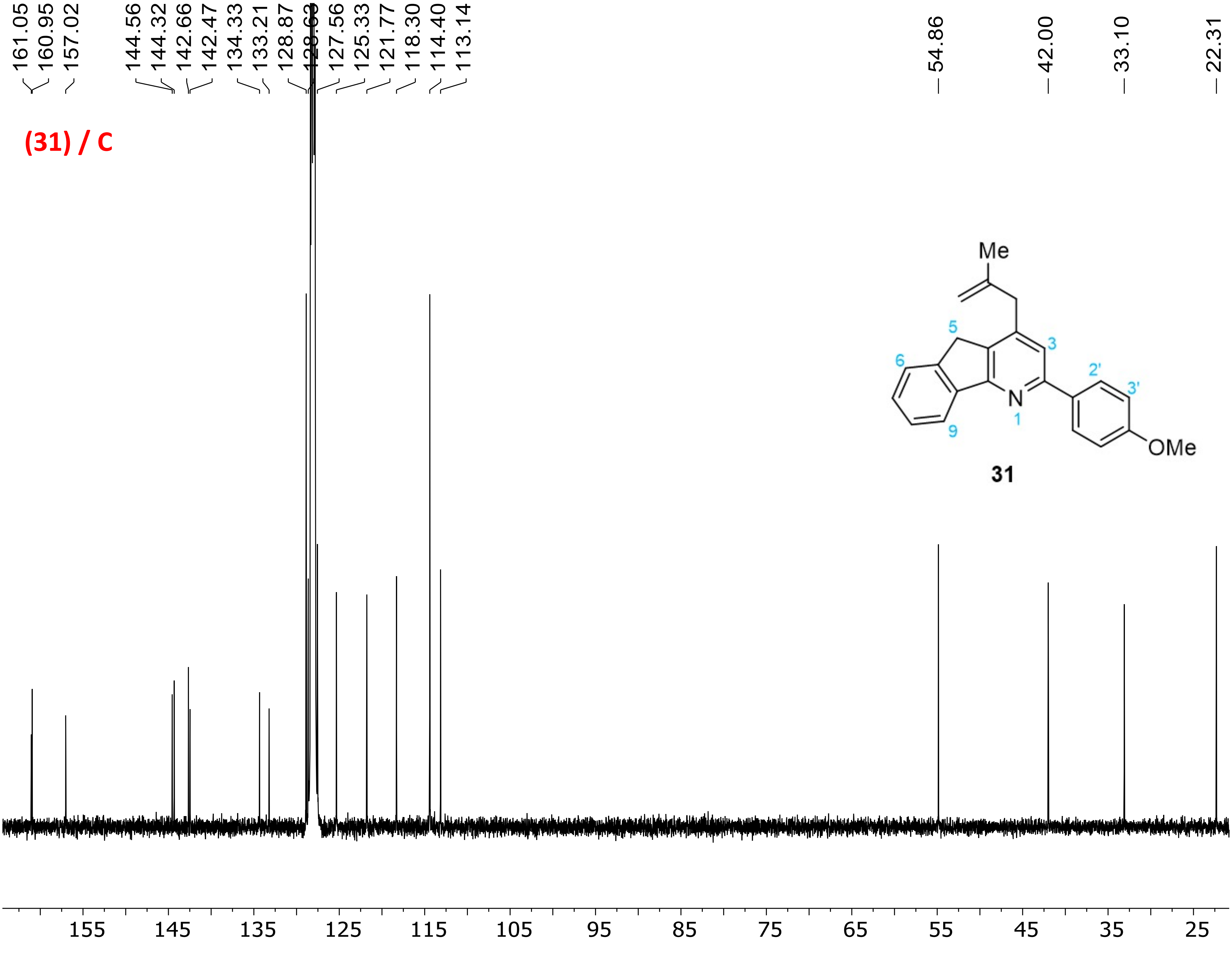
161.05
160.95
157.02
144.56
144.32
142.66
142.47
134.33
133.21
128.87
127.56
125.33
121.77
118.30
114.40
113.14
54.86
42.00
33.10
22.31
(31) / C
Me
5
3
6
2'
3'
N
1
9
OMe
31
155
145
135
125
115
105
95
85
75
65
55
45
35
25

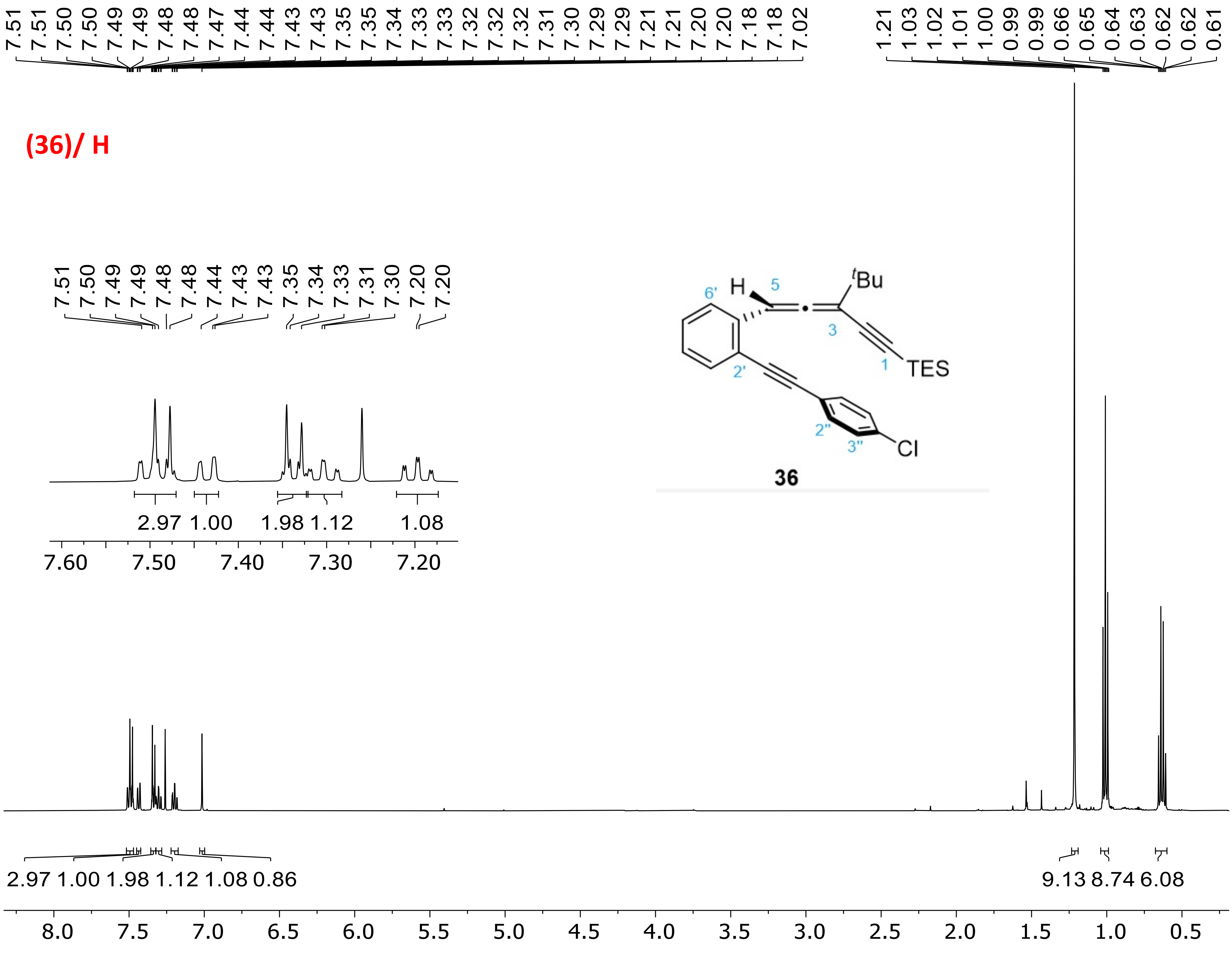
7.51 7.51 7.50 7.50 7.49 7.49 7.48 7.48 7.47 7.44 7.44 7.43 7.43 7.35 7.35 7.34 7.33 7.33 7.32 7.32 7.32 7.31 7.30 7.29 7.29 7.21 7.21 7.20 7.20 7.18 7.18 7.02
1.21 1.03 1.02 1.01 1.00 0.99 0.99 0.66 0.65 0.64 0.63 0.62 0.62 0.61
(36)/ H
7.51 7.50 7.49 7.49 7.48 7.48 7.44 7.43 7.43 7.35 7.34 7.33 7.31 7.30 7.20 7.20
2.97 1.00 1.98 1.12 1.08
7.60 7.50 7.40 7.30 7.20
H
5
6'
tBu
3
1
TES
2'
2''
3''
Cl
36
2.97 1.00 1.98 1.12 1.08 0.86
9.13 8.74 6.08
8.0 7.5 7.0 6.5 6.0 5.5 5.0 4.5 4.0 3.5 3.0 2.5 2.0 1.5 1.0 0.5

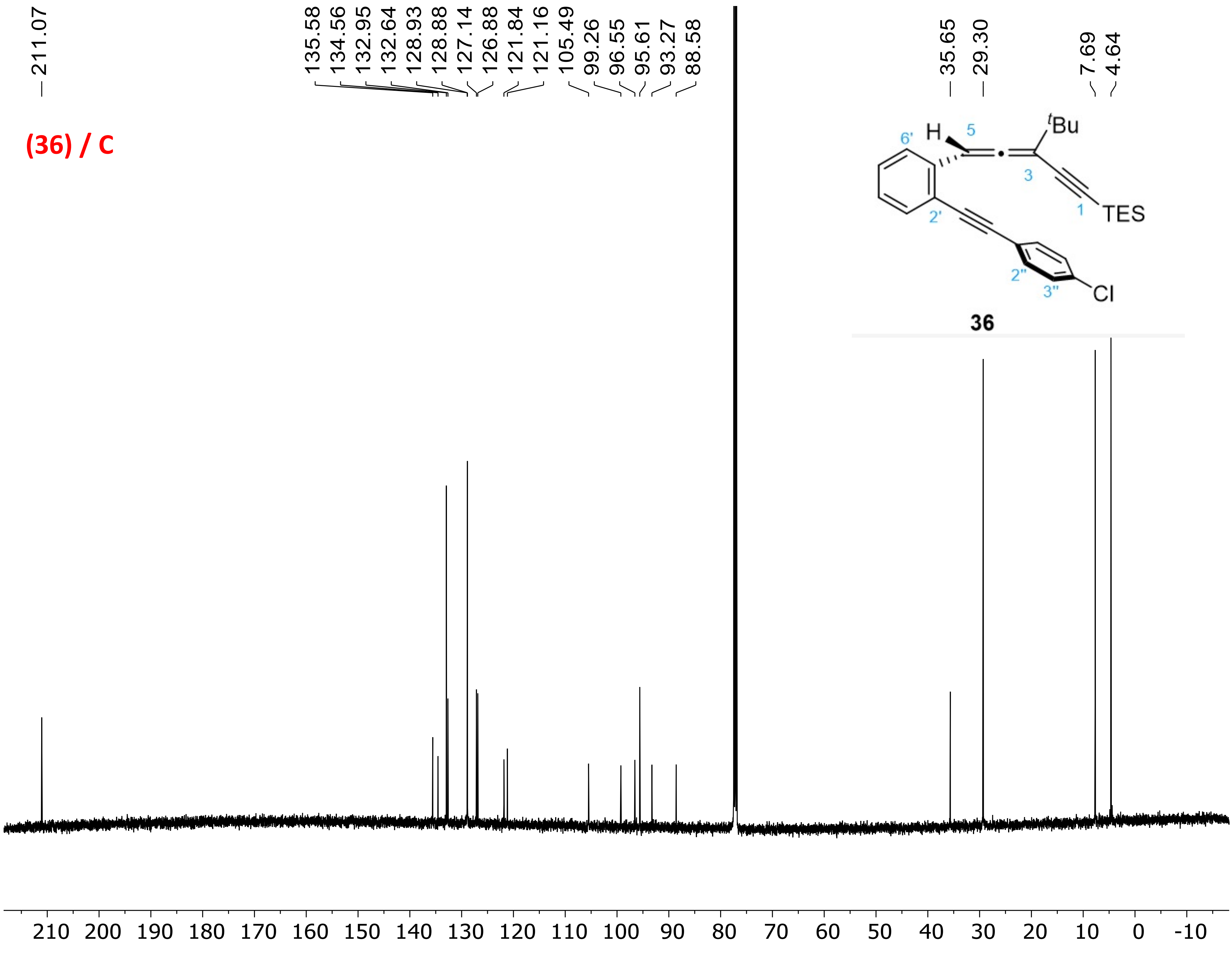

(36) / C
211.07
135.58
134.56
132.95
132.64
128.93
128.88
127.14
126.88
121.84
121.16
105.49
99.26
96.55
95.61
93.27
88.58
35.65
29.30
7.69
4.64
H
5
6'
tBu
3
1
TES
2'
2''
3''
Cl
36
210 200 190 180 170 160 150 140 130 120 110 100 90 80 70 60 50 40 30 20 10 0 -10

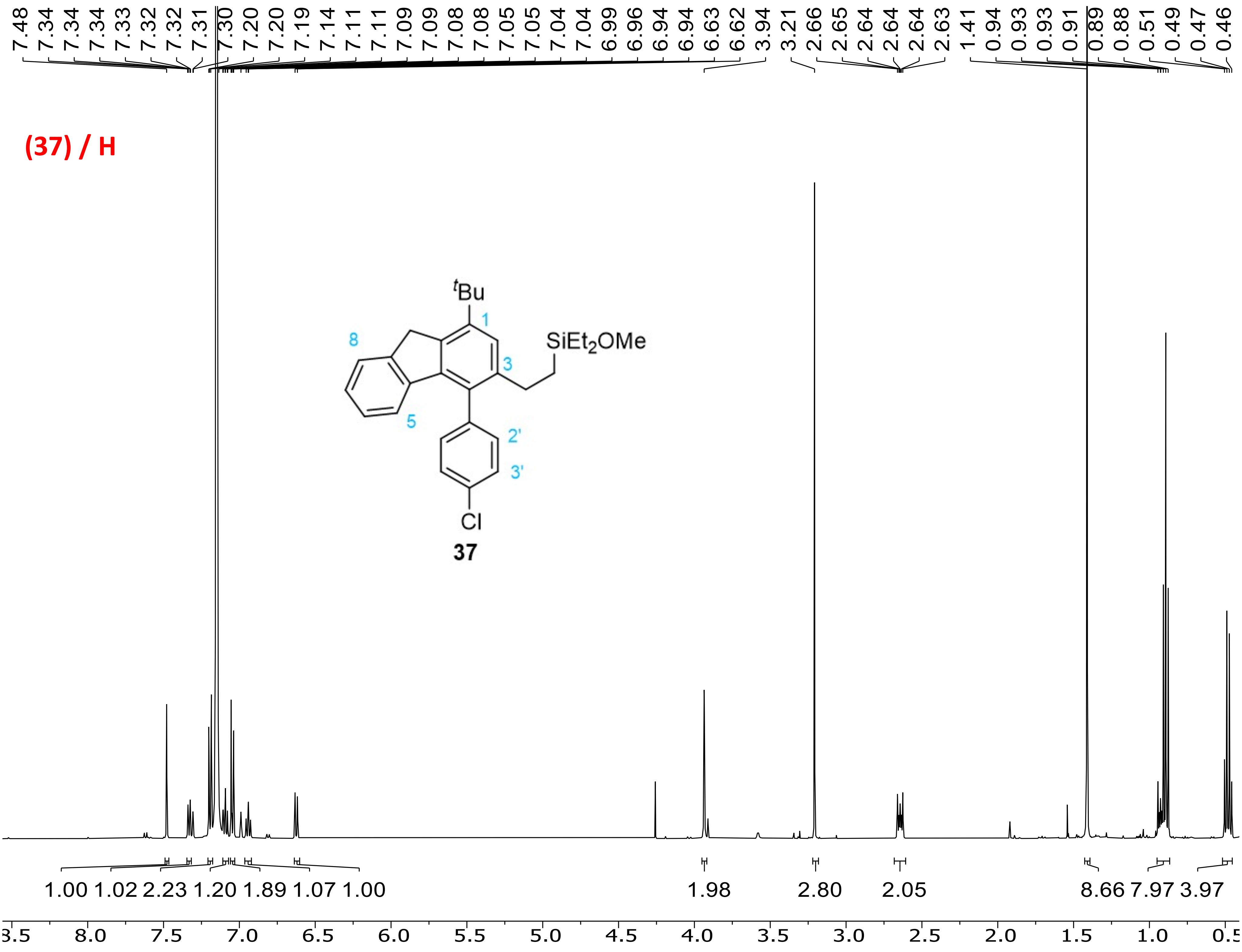
7.48
7.34
7.34
7.34
7.33
7.32
7.32
7.31
7.30
7.20
7.20
7.19
7.14
7.11
7.11
7.09
7.09
7.08
7.08
7.05
7.05
7.04
7.04
6.99
6.96
6.94
6.94
6.63
6.62
3.94
3.21
2.66
2.65
2.64
2.64
2.64
2.63
1.41
0.94
0.93
0.93
0.91
0.89
0.88
0.51
0.49
0.47
0.46
(37) / H
tBu
1
8
3
SiEt2OMe
5
2'
3'
Cl
37
1.00 1.02 2.23 1.20 1.89 1.07 1.00
1.98
2.80
2.05
8.66 7.97 3.97
8.5
8.0
7.5
7.0
6.5
6.0
5.5
5.0
4.5
4.0
3.5
3.0
2.5
2.0
1.5
1.0
0.5

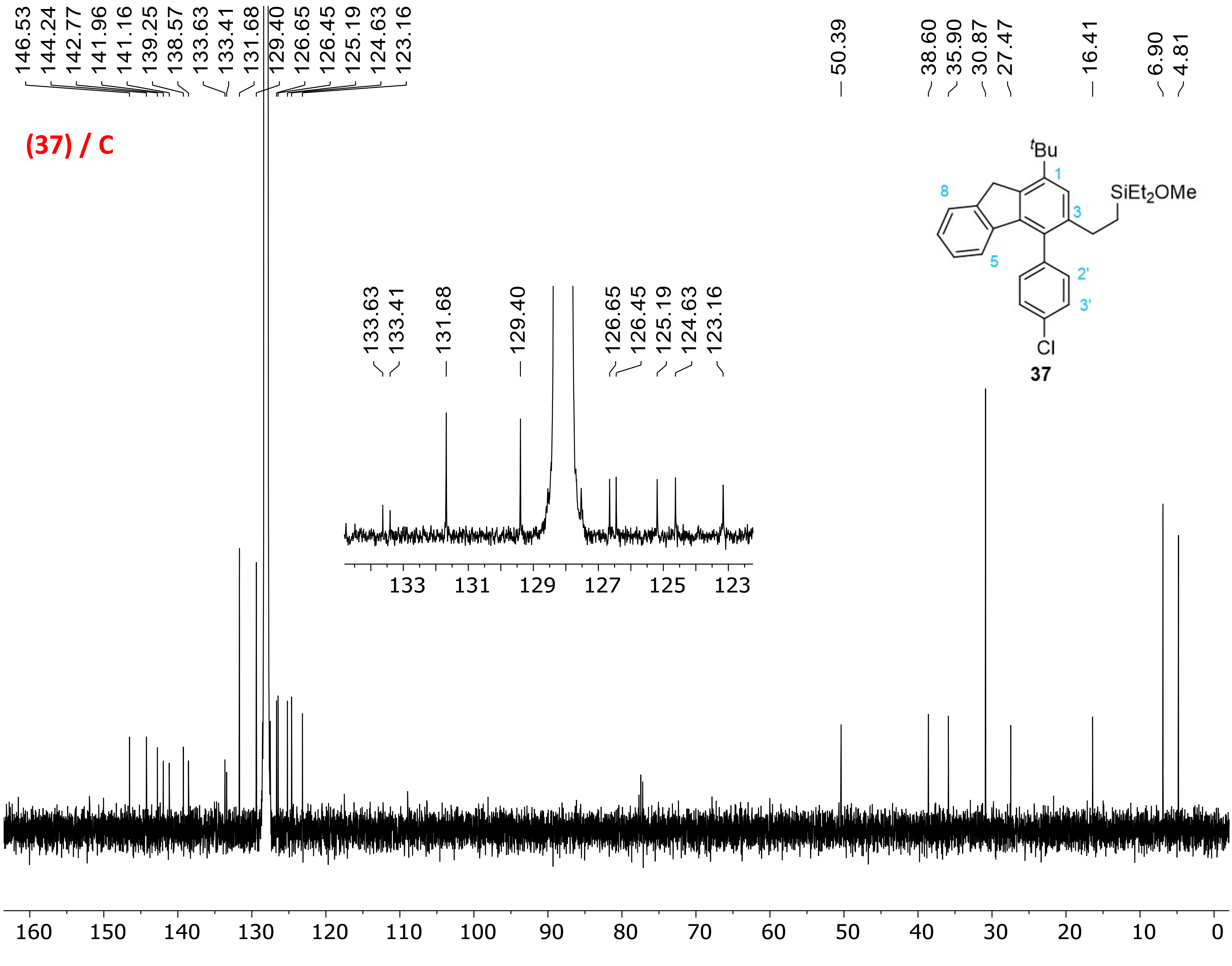
146.53
144.24
142.77
141.96
141.16
139.25
138.57
133.63
133.41
131.68
129.40
126.65
126.45
125.19
124.63
123.16
50.39
38.60
35.90
30.87
27.47
16.41
6.90
4.81
(37) / C
tBu
SiEt2OMe
Cl
37
133 131 129 127 125 123
160 150 140 130 120 110 100 90 80 70 60 50 40 30 20 10 0

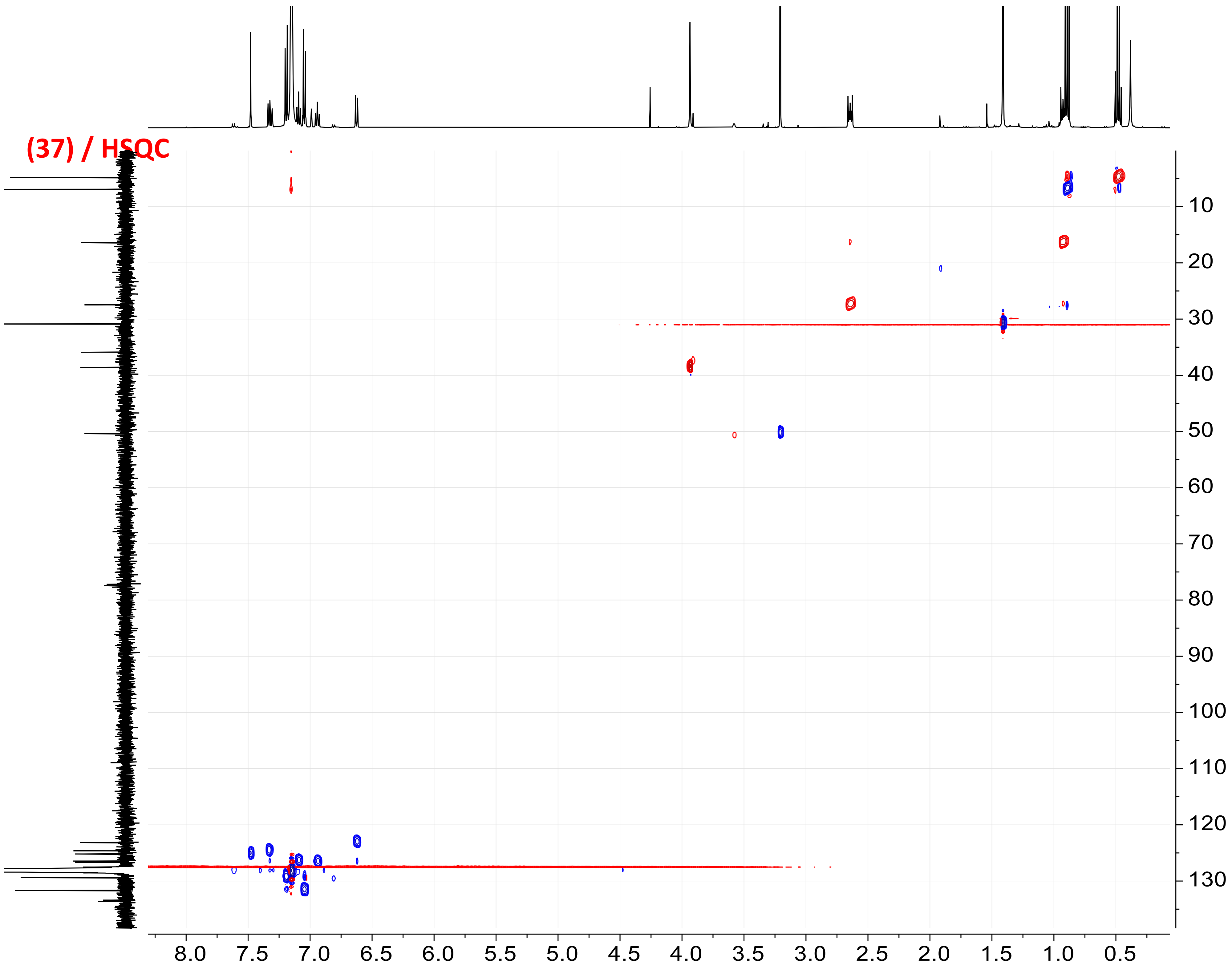
(37) / HSQC
10
20
30
40
50
60
70
80
90
100
110
120
130
8.0
7.5
7.0
6.5
6.0
5.5
5.0
4.5
4.0
3.5
3.0
2.5
2.0
1.5
1.0
0.5

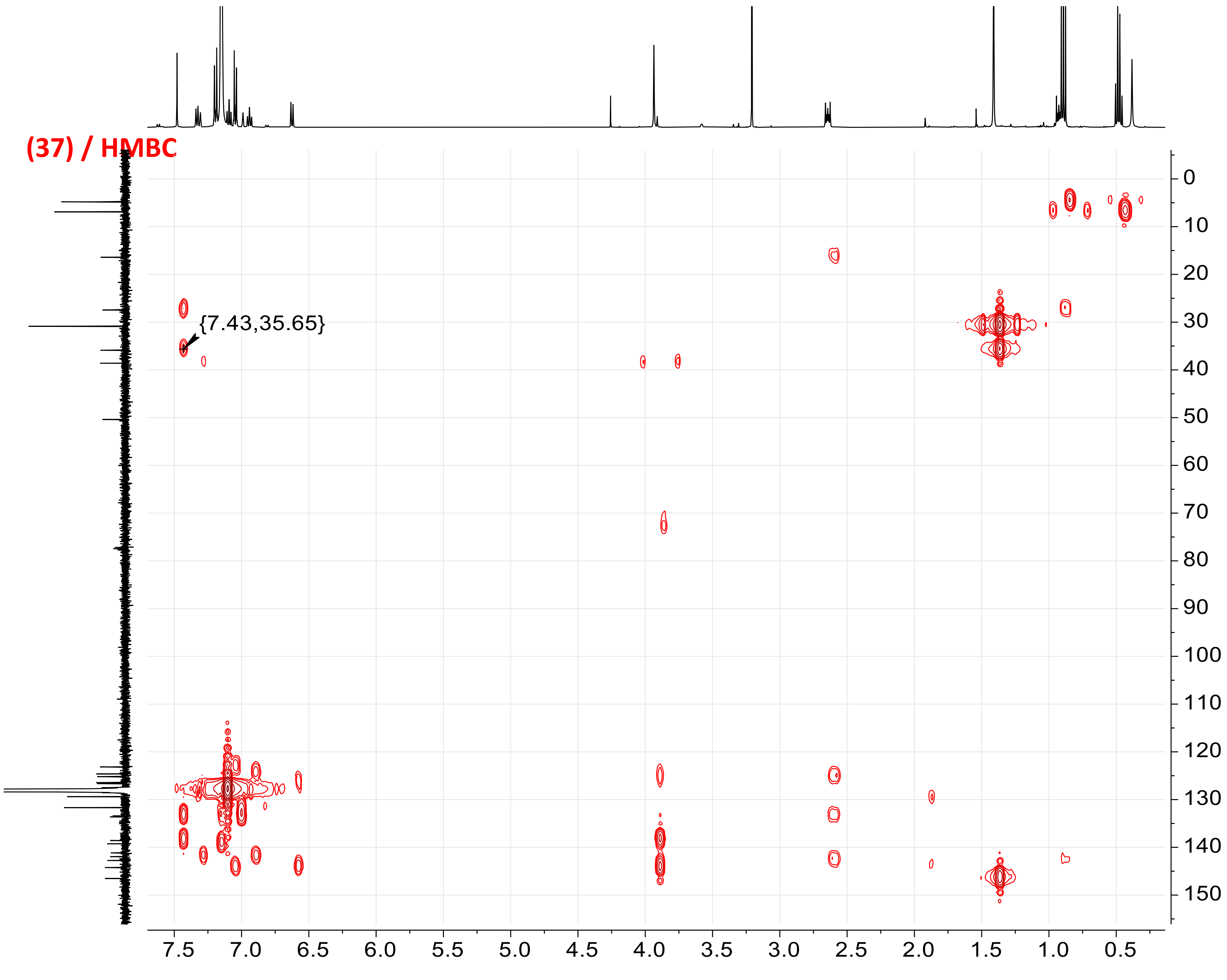
(37) / HMBC
{7.43,35.65}
0
10
20
30
40
50
60
70
80
90
100
110
120
130
140
150
7.5 7.0 6.5 6.0 5.5 5.0 4.5 4.0 3.5 3.0 2.5 2.0 1.5 1.0 0.5

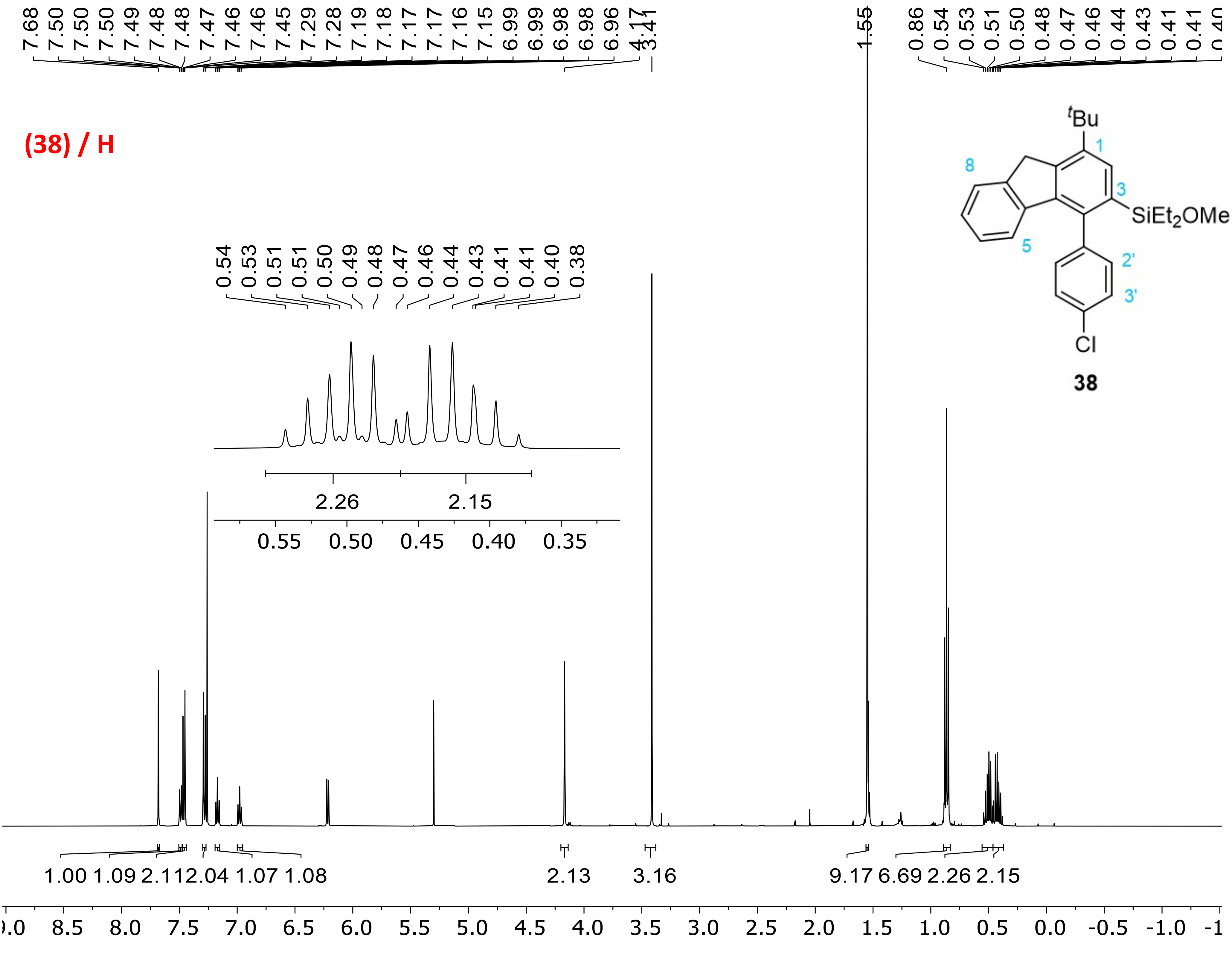
(38) / H
7.68 7.50 7.50 7.50 7.49 7.48 7.48 7.47 7.46 7.46 7.45 7.29 7.28 7.19 7.18 7.17 7.17 7.16 7.15 6.99 6.99 6.98 6.98 6.96 4.17 3.41
1.55
0.86 0.54 0.53 0.51 0.50 0.48 0.47 0.46 0.44 0.43 0.41 0.41
0.54 0.53 0.51 0.51 0.50 0.49 0.48 0.47 0.46 0.44 0.43 0.41 0.41 0.40 0.38
2.26 2.15
0.55 0.50 0.45 0.40 0.35
tBu
SiEt2OMe
Cl
38
1.00 1.09 2.11 2.04 1.07 1.08 2.13 3.16 9.17 6.69 2.26 2.15
8.5 8.0 7.5 7.0 6.5 6.0 5.5 5.0 4.5 4.0 3.5 3.0 2.5 2.0 1.5 1.0 0.5 0.0 -0.5 -1.0

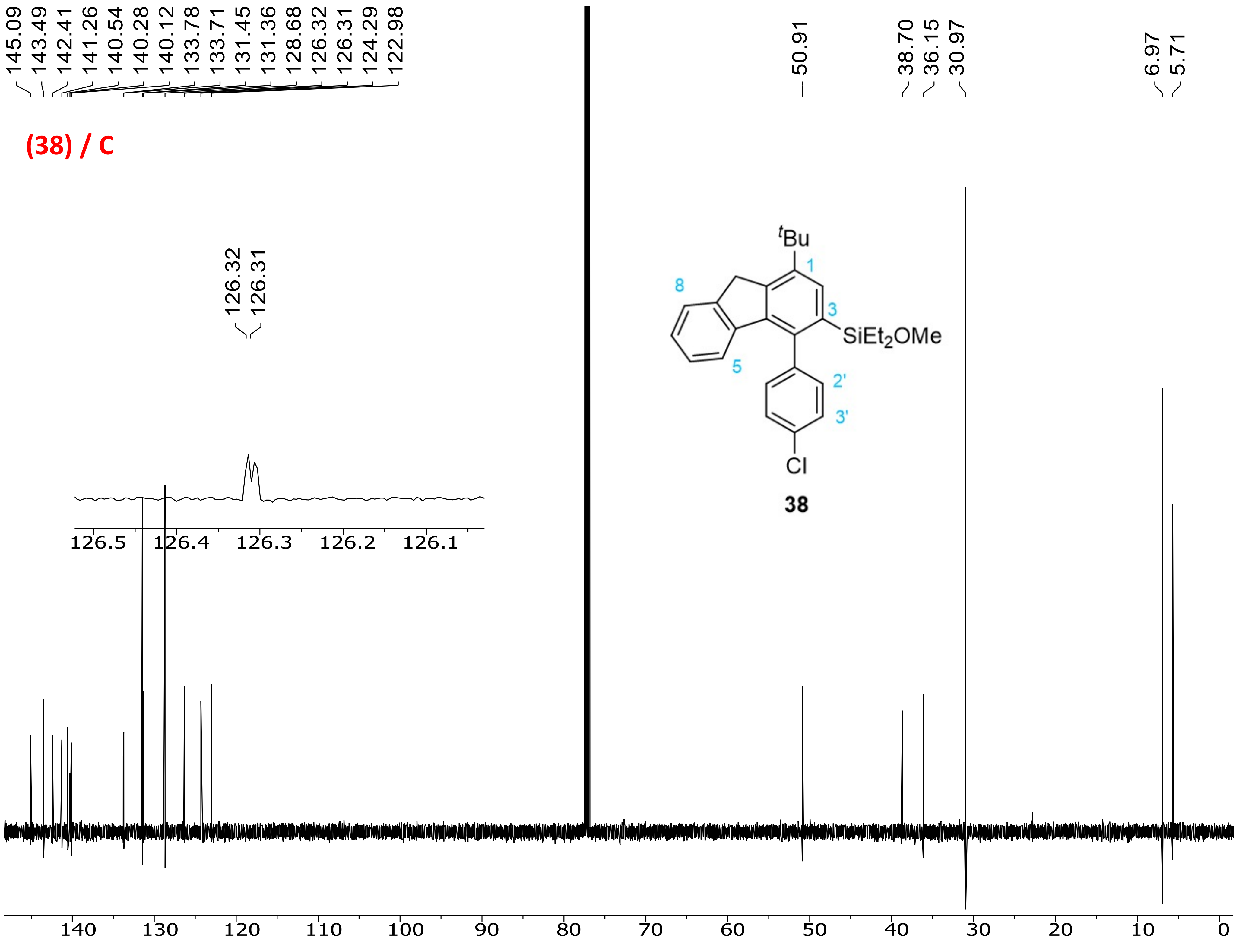

145.09
143.49
142.41
141.26
140.54
140.28
140.12
133.78
133.71
131.45
131.36
128.68
126.32
126.31
124.29
122.98
50.91
38.70
36.15
30.97
6.97
5.71
(38) / C
126.32
126.31
126.5
126.4
126.3
126.2
126.1
tBu
1
3
8
5
SiEt2OMe
2'
3'
Cl
38
140
130
120
110
100
90
80
70
60
50
40
30
20
10
0

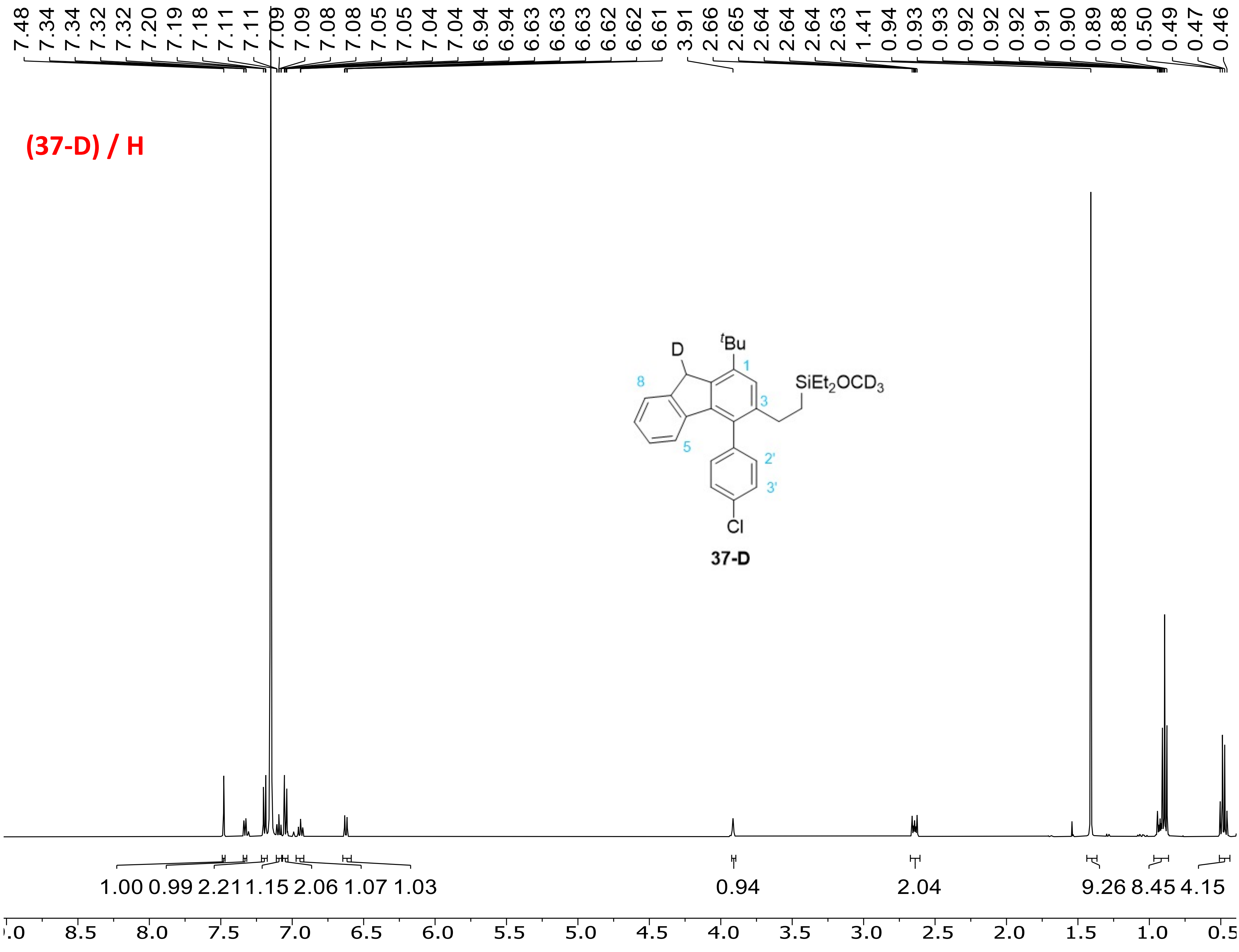
7.48 7.34 7.34 7.32 7.32 7.20 7.19 7.18 7.11 7.11 7.09 7.09 7.08 7.08 7.05 7.05 7.04 7.04 6.94 6.94 6.63 6.63 6.63 6.62 6.62 6.61 3.91 2.66 2.65 2.64 2.64 2.64 2.63 1.41 0.94 0.93 0.93 0.92 0.92 0.92 0.91 0.90 0.89 0.88 0.50 0.49 0.47 0.46
(37-D) / H
D
tBu
SiEt2OCD3
1
3
8
5
2'
3'
Cl
37-D
1.00 0.99 2.21 1.15 2.06 1.07 1.03
0.94
2.04
9.26 8.45 4.15
8.5 8.0 7.5 7.0 6.5 6.0 5.5 5.0 4.5 4.0 3.5 3.0 2.5 2.0 1.5 1.0 0.5

(37-D) / C

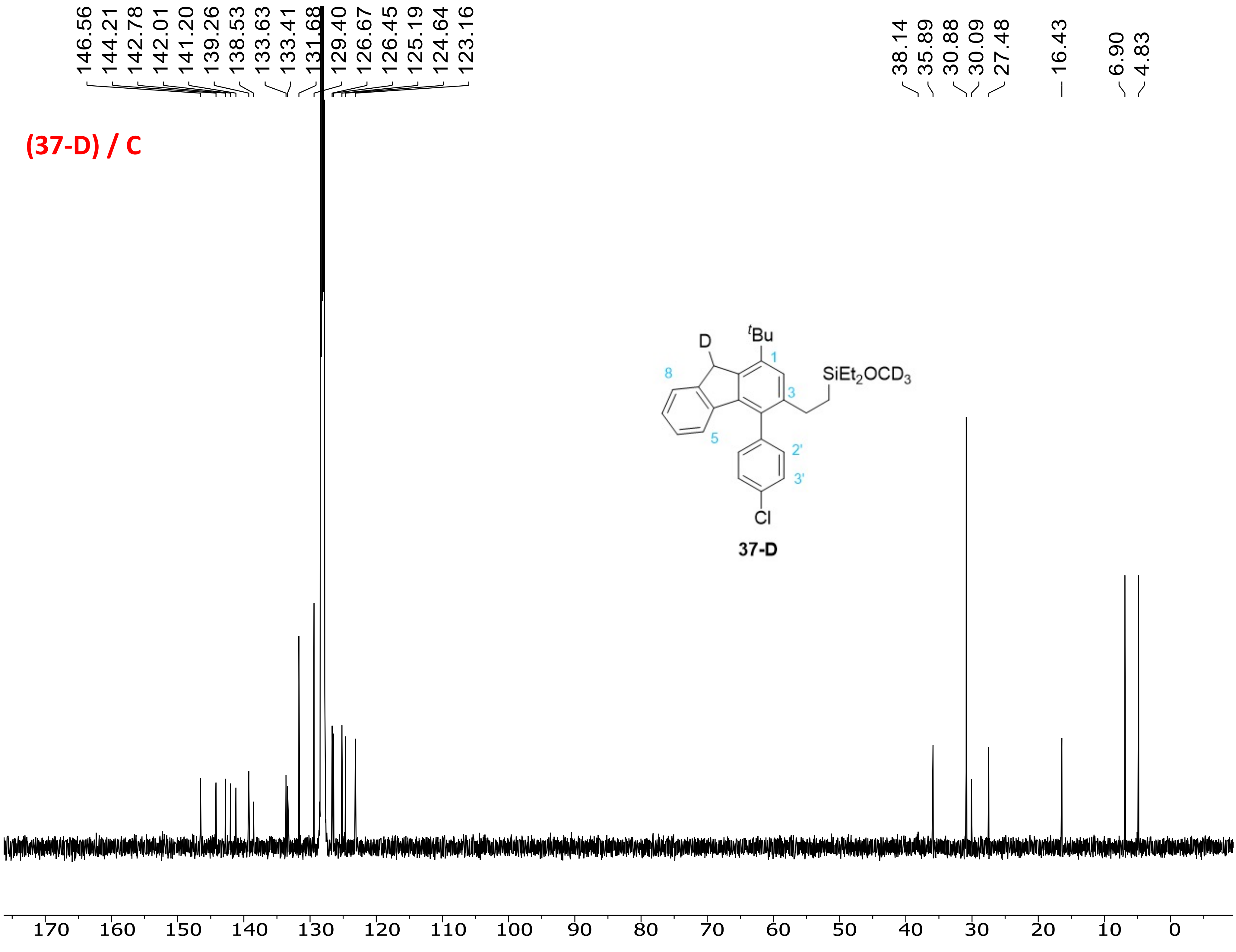
146.56
144.21
142.78
142.01
141.20
139.26
138.53
133.63
133.41
131.68
129.40
126.67
126.45
125.19
124.64
123.16
38.14
35.89
30.88
30.09
27.48
16.43
6.90
4.83
D
^{t}Bu
SiEt$_2$OCD$_3$
Cl
1
3
5
8
2'
3'
37-D
170 160 150 140 130 120 110 100 90 80 70 60 50 40 30 20 10 0

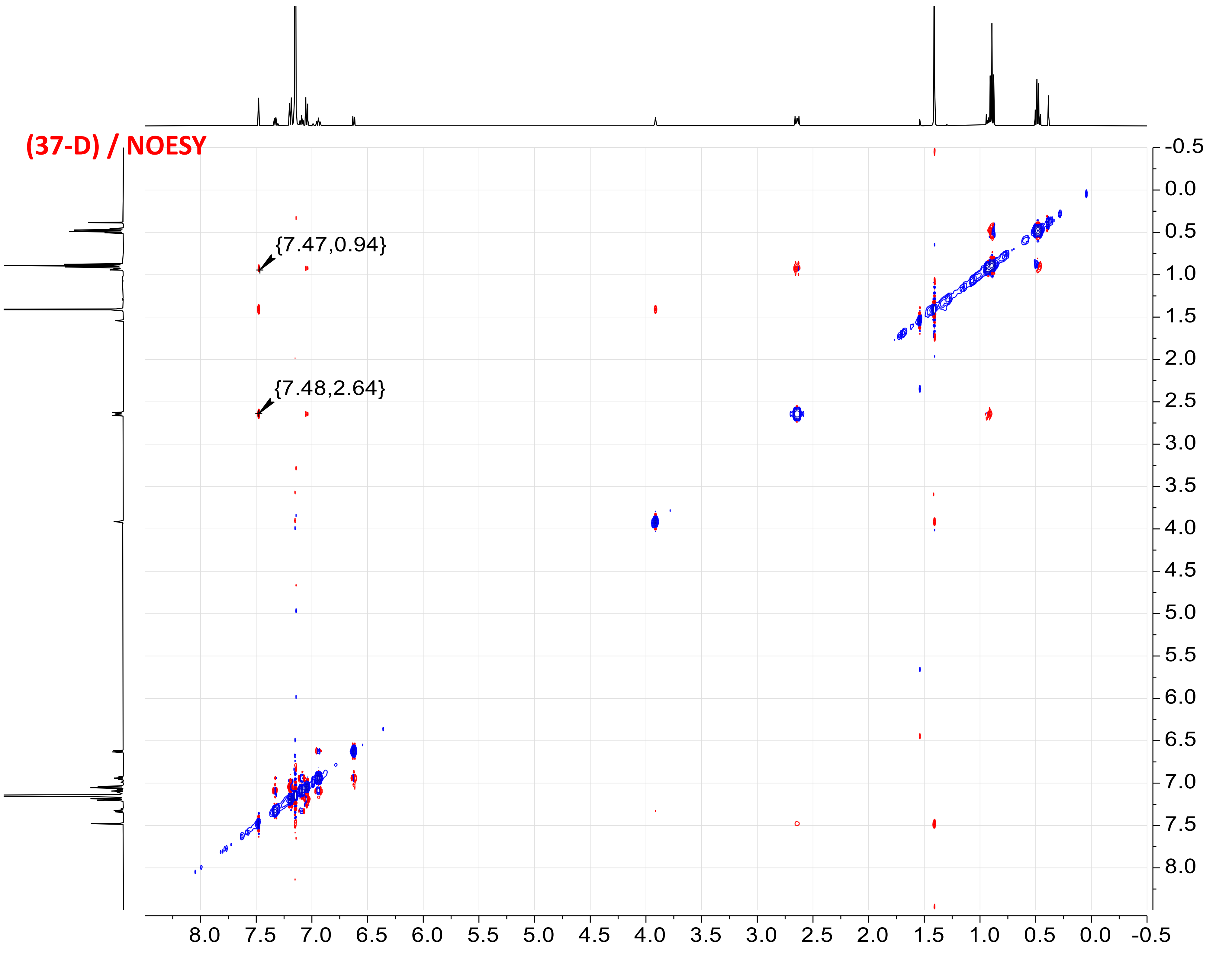
(37-D) / NOESY
{7.47,0.94}
{7.48,2.64}
8.0 7.5 7.0 6.5 6.0 5.5 5.0 4.5 4.0 3.5 3.0 2.5 2.0 1.5 1.0 0.5 0.0 -0.5
-0.5 0.0 0.5 1.0 1.5 2.0 2.5 3.0 3.5 4.0 4.5 5.0 5.5 6.0 6.5 7.0 7.5 8.0

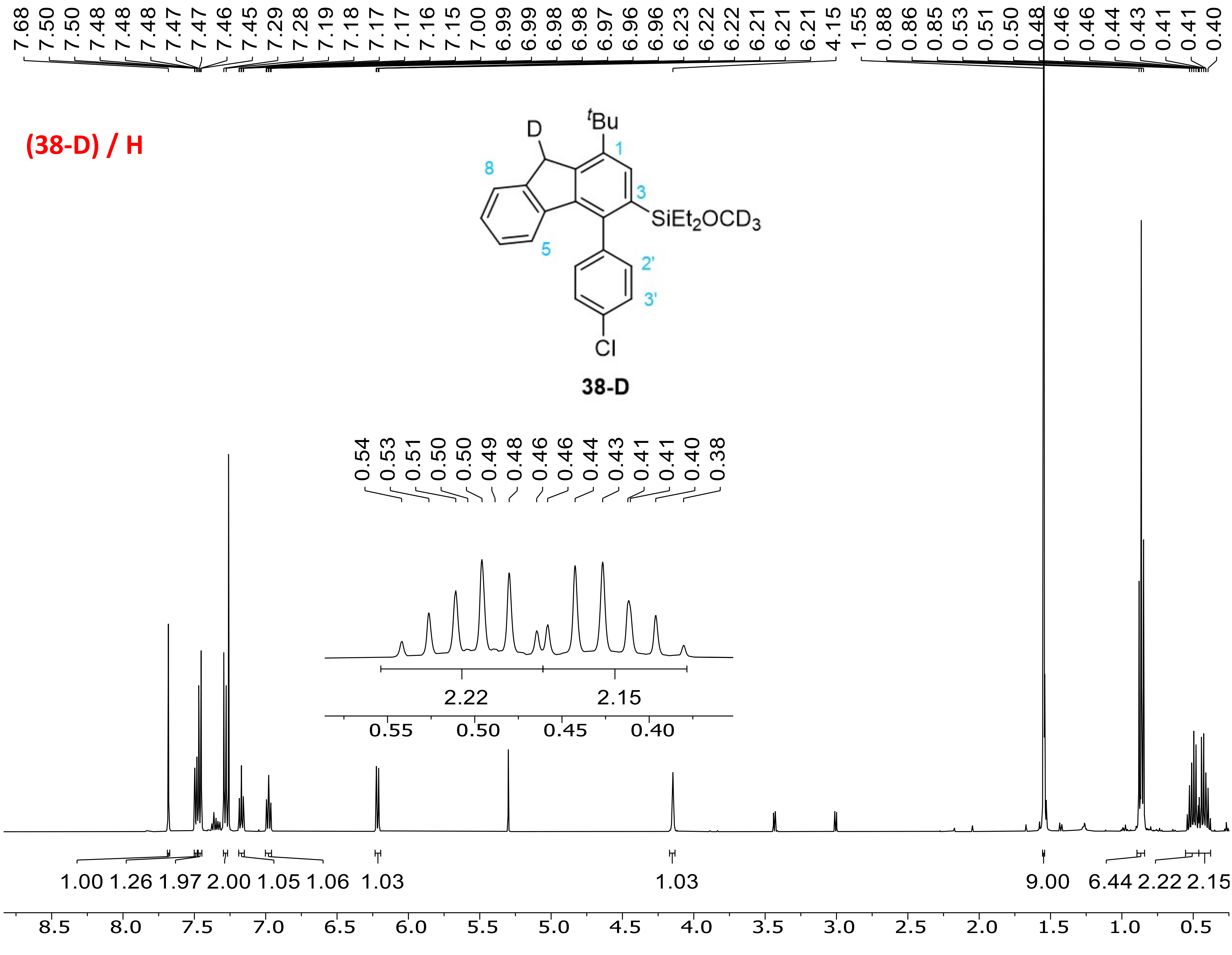
(38-D) / H
D
tBu
SiEt2OCD3
Cl
38-D
1.00 1.26 1.97 2.00 1.05 1.06 1.03
1.03
9.00 6.44 2.22 2.15
2.22
2.15

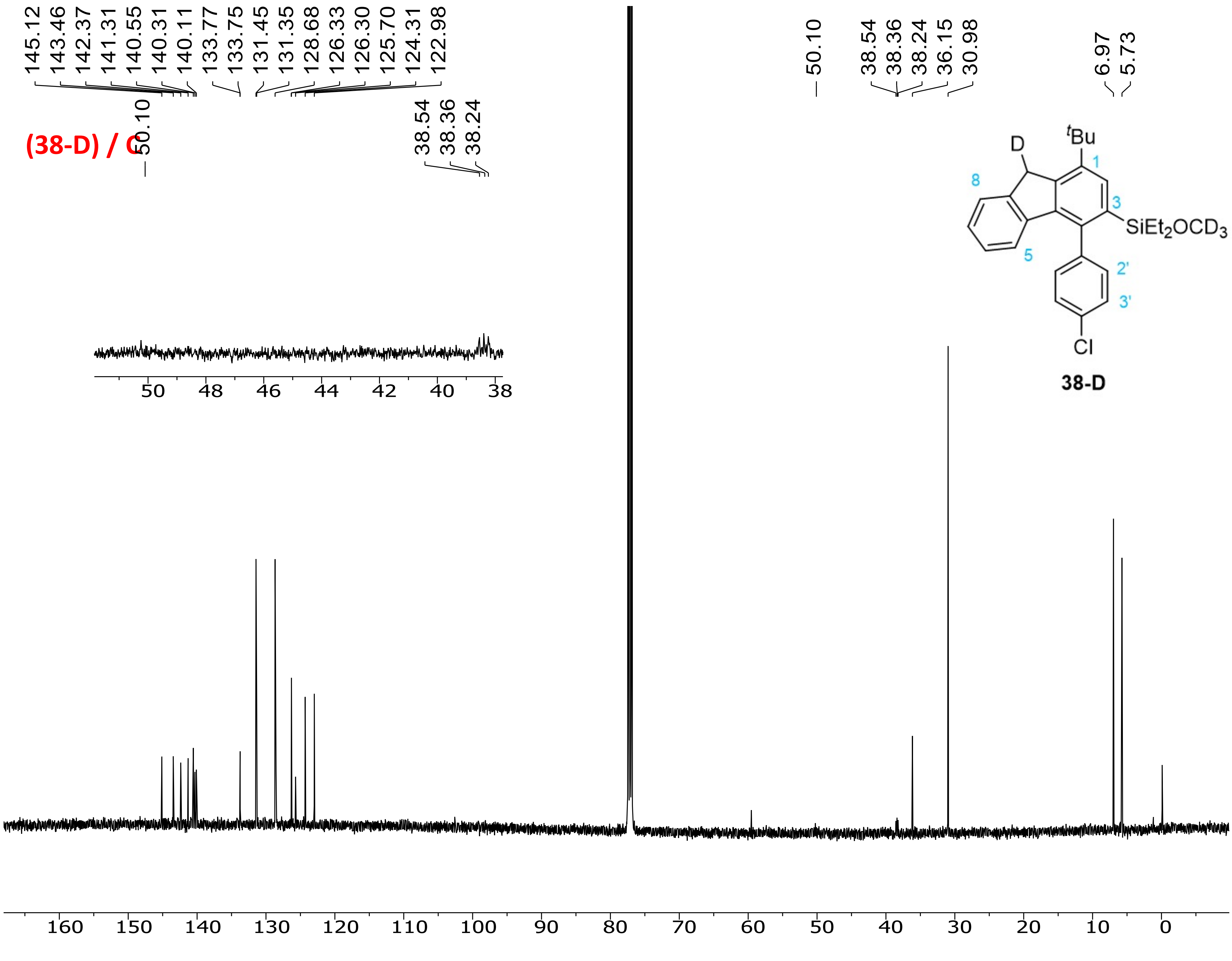
145.12
143.46
142.37
141.31
140.55
140.31
140.11
133.77
133.75
131.45
131.35
128.68
126.33
126.30
125.70
124.31
122.98
50.10
38.54
38.36
38.24
36.15
30.98
6.97
5.73
(38-D) / C
D
tBu
SiEt2OCD3
Cl
38-D

(38-D) / HSQC

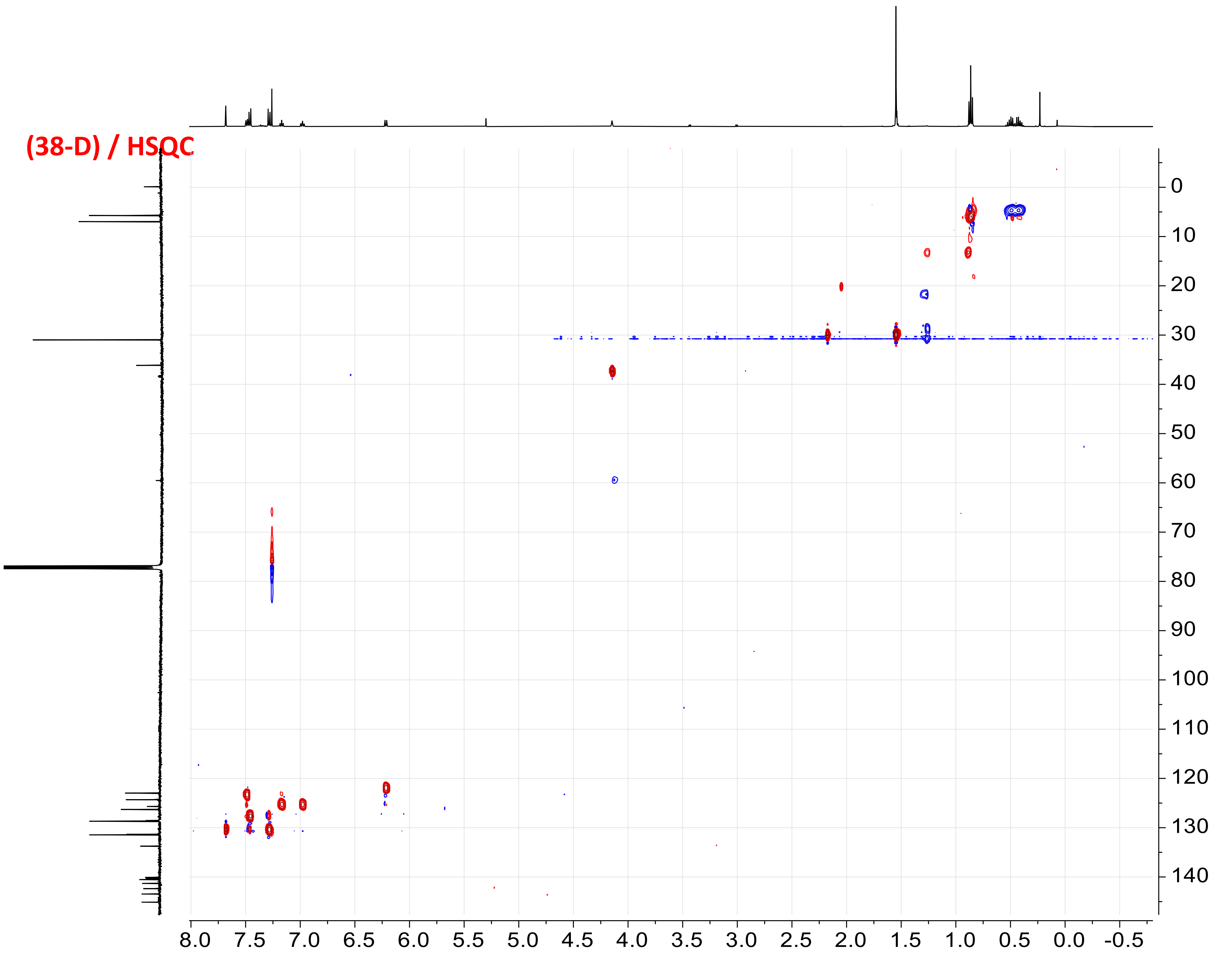
8.0 7.5 7.0 6.5 6.0 5.5 5.0 4.5 4.0 3.5 3.0 2.5 2.0 1.5 1.0 0.5 0.0 -0.5
0 10 20 30 40 50 60 70 80 90 100 110 120 130 140

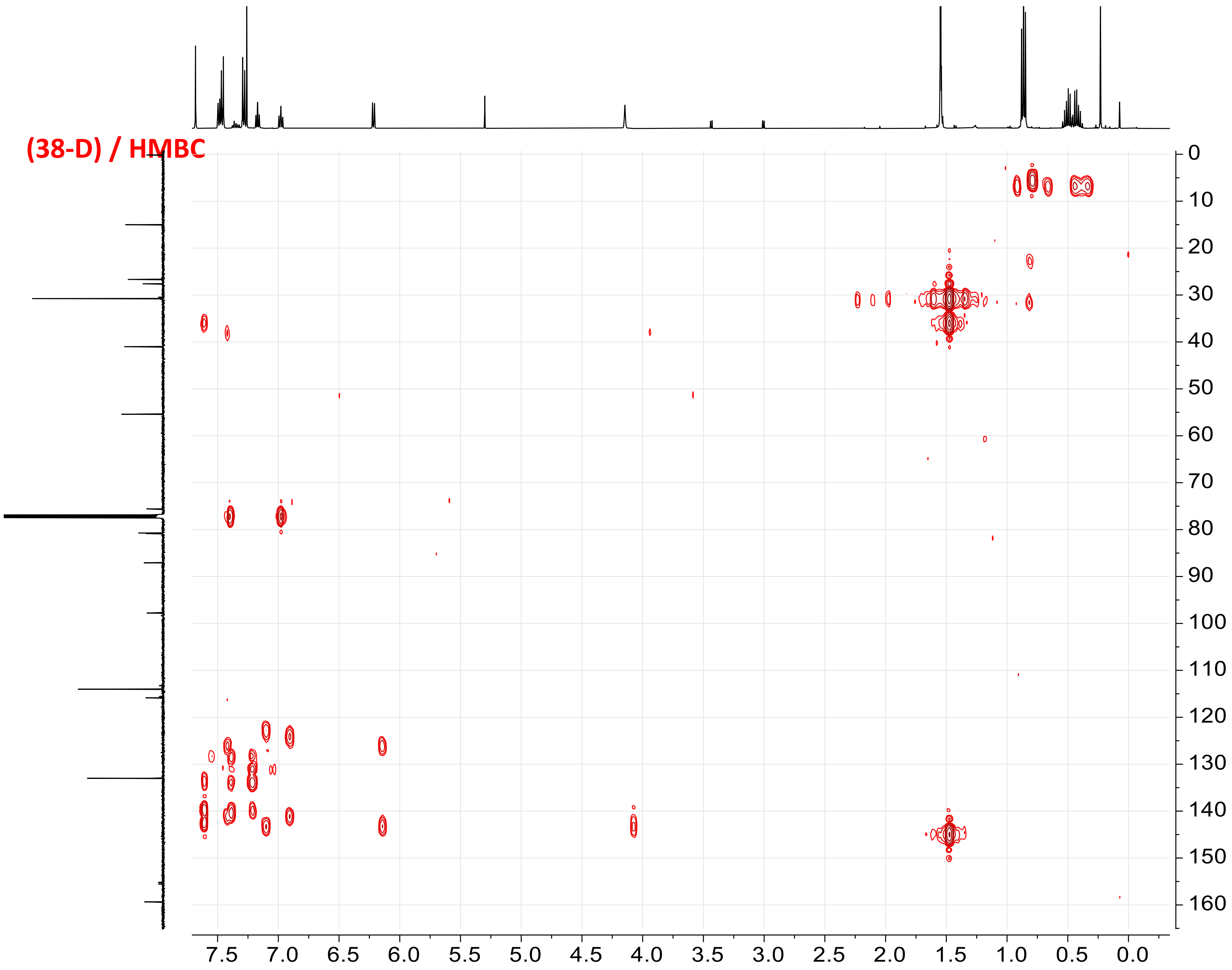
(38-D) / HMBC
7.5
7.0
6.5
6.0
5.5
5.0
4.5
4.0
3.5
3.0
2.5
2.0
1.5
1.0
0.5
0.0
0
10
20
30
40
50
60
70
80
90
100
110
120
130
140
150
160

(38-D) / NOESY

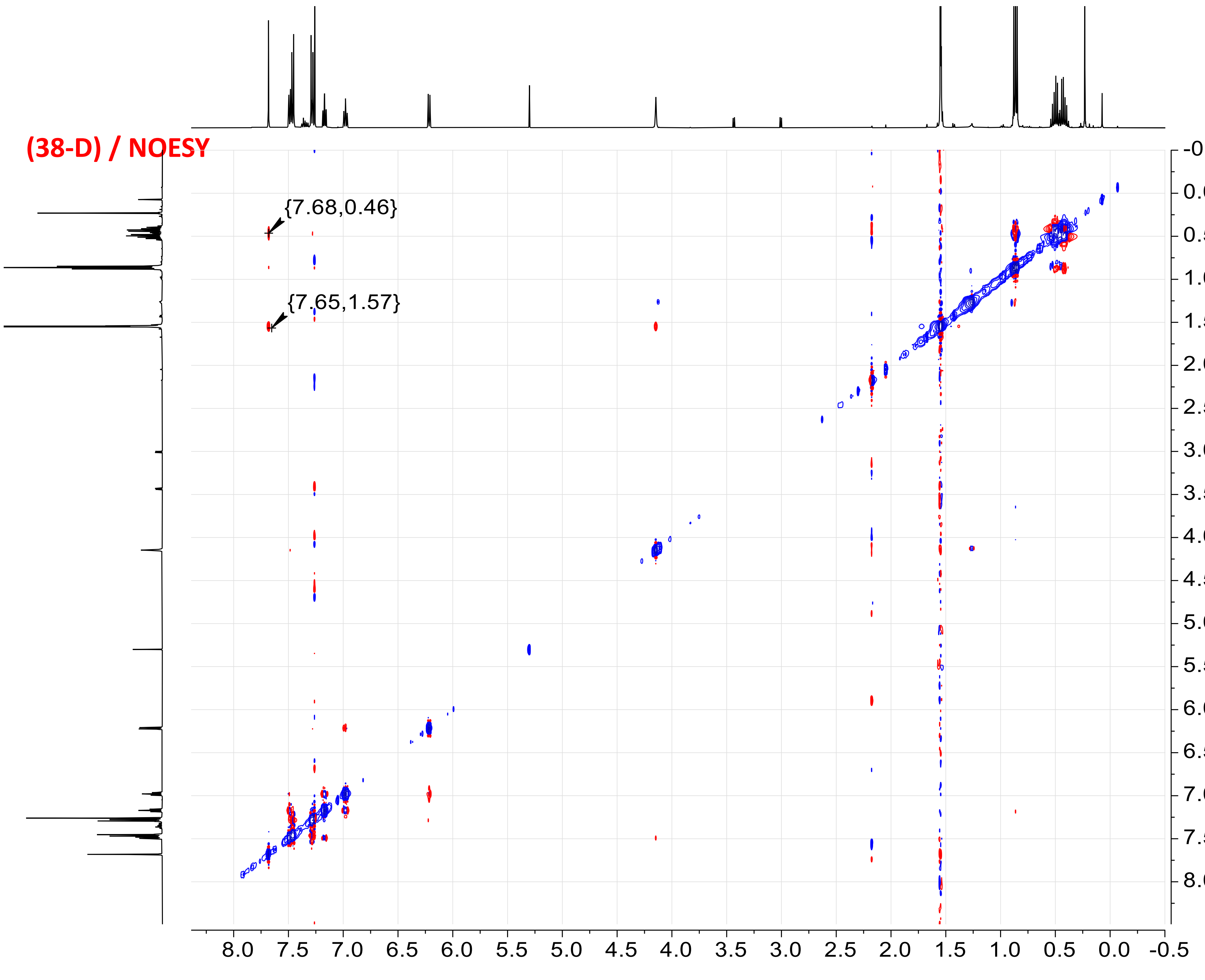
{7.68,0.46}
{7.65,1.57}
-0.5
0.0
0.5
1.0
1.5
2.0
2.5
3.0
3.5
4.0
4.5
5.0
5.5
6.0
6.5
7.0
7.5
8.0
8.0 7.5 7.0 6.5 6.0 5.5 5.0 4.5 4.0 3.5 3.0 2.5 2.0 1.5 1.0 0.5 0.0 -0.5
